# Variability in Corneal and Epithelial Pachymetry: A Comparison of Optopol Revo 130 and Optovue RTV XR Avanti in Healthy Patients

**DOI:** 10.3390/jcm14041295

**Published:** 2025-02-15

**Authors:** Edward Wylęgała, Katarzyna Kryszan, Kamila Rogacz, Katarzyna Bujała, Bogdan Dugiełło, Przemysław Woźniak, Adam Wylęgała

**Affiliations:** 1 Ophthalmology Department, Railway Hospital, 40-760 Katowice, Poland; ewylegala@sum.edu.pl (E.W.); rogaczkamila@gmail.com (K.R.); katarzynabujalaa@gmail.com (K.B.); bogdan.dugiello@gmail.com (B.D.); przemyslaw.z.wozniak@gmail.com (P.W.); 2 Chair and Clinical Department of Ophthalmology, Faculty of Medical Sciences in Zabrze, Medical University of Silesia, Okręgowy Szpital Kolejowy, Panewnicka 65, 40-760 Katowice, Poland; 3 Experimental Ophthalmology Unit, Department of Biophysics, II School of Medicine with the Division of Dentistry in Zabrze Medical University of Silesia, 40-752 Katowice, Poland; adam.wylegala@gmail.com

**Keywords:** corneal epithelial thickness, pachymetry, optical coherence tomography, device agreement, repeatability, reproducibility, OCT REVO FC 130, Optovue RTV XR Avanti, Bland–Altman analysis, intraclass correlation coefficient

## Abstract

**Background/Objectives:** The accurate measurement of corneal epithelial thickness (CET) and pachymetry is essential for diagnosing and managing corneal conditions such as keratoconus and for optimizing outcomes in refractive surgery. This study aimed to evaluate the agreement and repeatability of measurements between two anterior segment optical coherence tomography (AS-OCT) devices—OCT REVO FC 130 and Optovue RTV XR Avanti—in healthy individuals. **Methods:** This prospective, cross-sectional, observational study included 53 healthy participants. High-quality scans were performed using both devices by two trained operators. Agreement between devices was assessed using Bland–Altman analysis and Deming regression, while repeatability and reproducibility were evaluated through intraclass correlation coefficients (ICCs) and test–retest variability (TRT). **Results:** Both devices demonstrated high repeatability and reproducibility across central and peripheral zones, with ICCs exceeding 0.98 for central pachymetry and stroma measurements. Repeatability and reproducibility were slightly higher for the OCT REVO FC 130 compared to the Optovue RTV XR Avanti, particularly for central measurements. Bland–Altman analysis revealed a mean difference near zero with narrow limits of agreement for most parameters, indicating strong inter-device agreement. Variability in CET measurements was higher, potentially due to the inclusion of tear film in OCT segmentation. **Conclusions:** The OCT REVO FC 130 and Optovue RTV XR Avanti offer comparable reliability and precision for corneal thickness measurements in healthy eyes, with the REVO demonstrating slightly better repeatability and reproducibility. These findings support the use of either device in clinical practice and research for corneal assessment.

## 1. Introduction

Pachymetry and epithelial thickness measurements are critical in diagnosing and managing various corneal conditions, including keratoconus, corneal peripheral thinning disorders, infections, and dystrophies. These measurements help clinicians assess the severity of corneal thinning, monitor disease progression, and evaluate the effectiveness of treatment strategies. For example, in keratoconus, the regular monitoring of corneal thickness is essential to detect early changes that may necessitate intervention, such as corneal cross-linking. Similarly, epithelial thickness mapping can reveal early signs of corneal dystrophies or infections, guiding timely therapeutic decisions.

Furthermore, corneal thickness is a crucial parameter in refractive surgery. Accurate corneal thickness measurements help identify patients who may be at increased risk for complications after refractive surgery. Corneal thickness is essential for calculating the appropriate refractive correction and ensuring optimal visual outcomes. Monitoring changes in corneal thickness post-operatively can help detect complications and assess healing [1]. In refractive laser ablations, such as trans-epithelial photorefractive keratectomy (Trans-PRK) and phototherapeutic keratectomy, surgeons should account for the vertical variability in epithelial zone mapping to ensure precise refractive outcomes and prevent the introduction of unintended corneal aberrations [2].

Imaging technologies have played a key role in the development of corneal epithelial thickness mapping, enabling a more accurate and detailed assessment of this important component of the eye.

Moreover, the consistency of these measurements over time is crucial, particularly when using the same instrument. Even though different devices may yield slightly different absolute values, the relative changes observed with the same machine can reliably indicate a patient’s response to treatment. Therefore, pachymetry and epithelial thickness measurements not only serve as diagnostic tools but also play a significant role in customizing treatment plans to the specific needs of patients with corneal conditions.

Earlier methods of measuring corneal epithelial thickness (CET) were based on point measurements, which provided a limited picture of its structure. Confocal microscopy, despite its excellent axial resolution, was unable to provide the holistic view of the cornea necessary for mapping. Similarly, hand-held ultrasound pachymeters were of limited use in CET mapping.

A breakthrough came with the introduction of very high-frequency ultrasound and optical coherence tomography (OCT) technology. These devices, by analyzing data from a wider area of the cornea, made it possible to create maps from larger diameters [3].

In this study, we assessed the agreement and repeatability both between and within instruments and compared the test–retest variabilities of epithelium, stroma, and pachymetry measurements taken from the OCT REVO FC 130 (Optopol Technology; Zawiercie, Poland) and Optovue RTV XR Avanti (Optovue Inc., Fremont, CA, USA) in healthy individuals.

## 2. Materials and Methods

### 2.1. Study Procedures

This is a prospective, cross-sectional, observational study performed in accordance with the Declaration of Helsinki and was reviewed and approved by the bioethics committee of the Silesian Medical University (Commission approval number KNW/0022/KB1/128/18/19, dated 8 January 2019).

Informed consent was obtained from all patients before any study procedure or examinations were performed. The study population was composed of healthy participants. Healthy participants were defined as individuals with no history of ocular disease, surgery, or contact lens wear. Additionally, all participants underwent a comprehensive ophthalmic examination, including slit lamp examination, tonometry, and corneal topography, to confirm the absence of any corneal abnormalities.

We included in the study 53 participants (28 females and 25 males) aged 36.82 median 35.5 (19–71 years old), and only one eye per subject was included (29 right eyes and 24 left).

Two operators (Operator1: K.K, and Operator2: K.R.) performed scanning acquisition for both OCT devices. REVO FC 130 examinations were acquired in version 11.5.1. Avanti XR RTVue examinations were acquired with version 2018.1. For the agreement study, the operators took at least 1 excellent-quality examination for the Anterior Radial 8 mm program (REVO FC 130, Optopol Technology; Zawiercie, Poland) and 1 excellent-quality examination for the Pachymetry Wide program (Avanti XR RTVue, Optovue Inc., Fremont, CA, USA). For each patient, the examination repeats were taken during one visit, on the same day with a short time interval. Before the scan acquisition, the patient was asked to blink and keep the examined eye open during the scan.

### 2.2. Quality Assessment and Segmentation Correction

Post-examination quality verification was performed for the whole dataset. The following were checked and served as the exclusion criteria for the examinations: eyelid occupying the area above 0.5 mm, missing scans, eye blinks, lack of central vertex, and examination not performed centrally.

If more than one eye was scanned for the patient, only one randomly selected eye was used for the agreement or precision calculation.

For the agreement study, each patient’s data from both devices were reviewed for quality and excluded if either met any exclusion criteria. For precision, the patient’s data were excluded if three acceptable (or six for reproducibility) scans were not obtained.

### 2.3. Statistical Analyses

Statistical analyses were performed using R version 4.3. [4] and RStudio version 23.12.1 [5] to ensure robust and reproducible computation. Various specialized R packages were utilized to generate visualizations and perform advanced statistical modeling.

The ggplot2(Version 0.1.2), ggtext(Version 0.1.2), gridExtra(Version 2.3), and ggpubr(Version 0.6.0) packages were employed for generating publication-quality plots [6,7,8,9]. These tools allowed for the creation of figures, including scatter plots, histograms, and overlays, enhancing the presentation of results. Annotations and custom formatting were applied to figures using ggtext(Version 0.1.2), while gridExtra(Version 2.3) facilitated the alignment and combination of multiple plots for comparative analysis.

For Bland–Altman analyses, the SimplyAgree(Version 0.0.2) package was used [10] to evaluate agreement between devices. This method provided key statistical outputs, such as mean differences (bias), limits of agreement (LOAs), and confidence intervals for LOAs. Bland–Altman plots, generated through this package, visually depicted the agreement by plotting measurement differences against their averages, revealing any systematic bias or proportional discrepancies.

The mcr(Version 1.3.2) package was utilized for performing Deming regression [11], a technique that accounts for measurement errors in both variables. This regression method was chosen over ordinary least squares regression due to its suitability for evaluating the relationship between two devices without assuming a reference standard. Outputs included slope and intercept estimates with confidence intervals, providing insights into the proportional and systematic agreement between devices.

For precision calculations, the lme4(Version 1.1-10) and performance(Version 3.0) packages were applied [12,13]. These tools allowed for the modeling of random and fixed effects to assess repeatability and reproducibility. Key metrics included within-subject standard deviation (SW), test–retest variability (TRT), and intraclass correlation coefficients (ICCs). The random-effects models effectively captured operator variability and subject-specific differences, while the performance (Version 3.0)package streamlined the evaluation of model fit and reliability of the measurements.

### 2.4. Agreement

The distribution of the mean differences between the two devices (Avanti XR RTVue—REVO FC 130) for each measured parameter was evaluated for normality using the Shapiro–Wilk test. Parameters demonstrating a non-normal distribution would have necessitated non-parametric methods; however, normality allowed the application of parametric tests.

To assess whether the observed mean differences were statistically significant, a paired *t*-test was conducted for each parameter. This test compared the paired measurements from the two devices while accounting for the within-subject variability. The significance level was set at α = 0.05. Agreement between the REVO FC 130 and Avanti XR RTVue OCT devices was analyzed using the Bland–Altman method, a robust statistical tool for comparing two measurement techniques [14,15,16,17]. For each parameter, the Bland–Altman analysis provided the following:

- Mean difference (bias): Representing the systematic difference between the two devices.
- Limits of Agreement (LOAs): Defined as the mean difference ±1.96 standard deviations (SD), providing the range within which 95% of differences between measurements are expected to lie.
- Confidence Intervals (CI): Calculated for the LOAs to provide an estimate of their precision and reliability.

The Bland–Altman plots visually represented the agreement, displaying the differences between the two devices’ measurements against their average. This approach highlighted potential trends or proportional biases that could indicate systematic discrepancies between the devices.

In addition to Bland–Altman analysis, Deming regression was employed to further evaluate the relationship between the two devices. Unlike ordinary least squares regression, Deming regression accounts for measurement error in both variables, making it more suitable for this comparison. Each parameter was modeled independently under the assumption of equal error variance in both devices, reflecting the expectation that neither device serves as a perfect reference standard.

Deming regression provided the following:

- Slope and intercept estimates: Indicating the proportional and systematic agreement between devices.
- Confidence intervals for regression coefficients: Allowing for statistical inference on the consistency of measurement relationships [18,19].

### 2.5. Precision

To establish the precision of the REVO FC 130, both intra-examiner repeatability and inter-examiner reproducibility were evaluated systematically. The study included two trained operators, each performing a minimum of three acceptable scans per patient to ensure consistency and data quality.

For the precision study, repeatability and reproducibility were assessed using high-quality scans conducted by both operators. Repeatability was independently determined for each operator based on three repeated scans, allowing for the evaluation of consistency within a single examiner. Reproducibility was assessed by incorporating six scans from the two operators, enabling a comprehensive analysis of variability between different examiners. These metrics were calculated to quantify precision:

- Overall means (Ῡ) with standard deviation (SD): Provided a baseline measure of the central tendency and dispersion of thickness values.
- Within-subject standard deviation (SW): Quantified the variability in measurements for the same patient under identical conditions, reflecting the reliability of repeated scans.
- Test–retest variability (TRT): Calculated as √2 × 1.96 × SW, this metric offered a direct estimate of the range within which repeated measurements could be expected to fall.
- Coefficient of variation (COV): Expressed as a percentage, calculated as (100 × SW)/Ῡ, it indicated relative variability in relation to the mean thickness values.
- Intraclass correlation coefficient (ICC): Provided a statistical measure of the reliability of measurements, with values closer to 1.0 indicating stronger agreement between scans.

Reproducibility metrics were further analyzed using a random effects ANOVA model. This model incorporated random effects for the operator and patient–operator interaction while including the fixed effect of the eye. This approach allowed for a robust evaluation of reproducibility, estimating SW, TRT, COV, and ICC accordingly.

For the agreement study, a total of 53 eyes were scanned to evaluate the consistency of results across devices and conditions. Specific inclusion and exclusion criteria were applied to maintain the quality of the dataset. For the REVO FC 130, nine patients were excluded due to eyelid interference (*n* = 6) and image shifting (*n* = 3). Similarly, for the Avanti XR RTVue, exclusions included eyelid interference (*n* = 1, 0.7%), image shifting (*n* = 1, 0.7%), incorrect scan centralization (*n* = 13, 9%), and extensive tear-film irregularities (*n* = 1, 0.7%). These exclusions highlighted common technical challenges encountered during imaging procedures and underscored the importance of addressing these issues to improve data reliability.

## 3. Results

The Avanti device tends to produce slightly higher standard deviations, particularly for epithelial and stromal thickness measurements, indicating higher variability compared to the REVO device. Pachymetric measurements showed minimal differences in variability between the two devices (Table 1).

**Figure 1:**
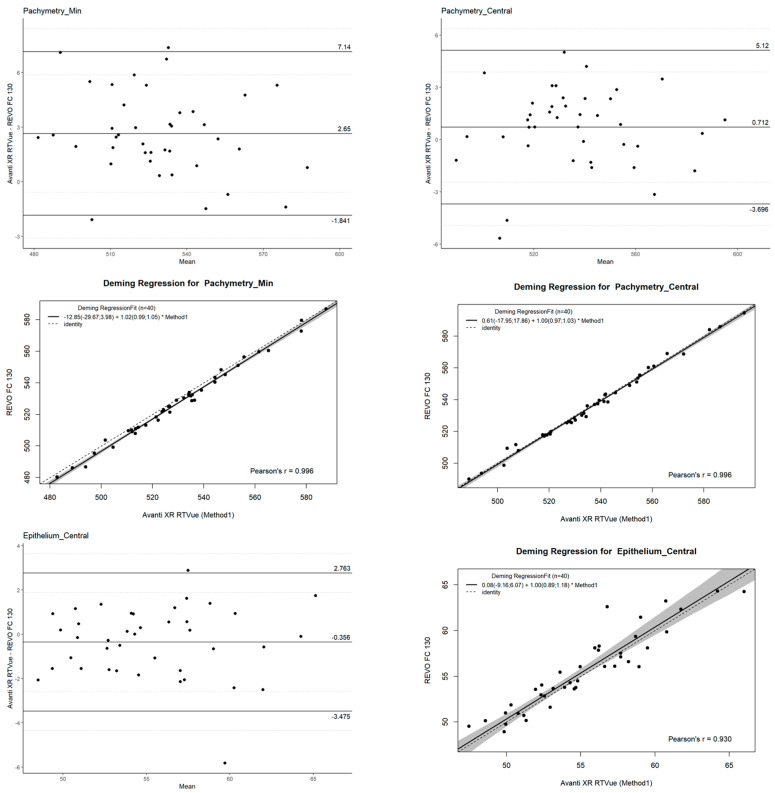
Bland–Altman plot and Deming regression for central epithelium and corneal thickness.

The central pachymetry values (Pachymetry_Min, Pachymetry_Median, and Pachymetry_Central) demonstrated high repeatability and reproducibility (Figure 1). For instance, Pachymetry_Min had mean values of 531.14 μm and 531.01 μm for Operators 1 and 2, respectively, with corresponding SDs of 31.97 μm and 31.88 μm. The reproducibility mean was 531.08 μm with an SD of 31.85 μm. The ICC values were exceptionally high (0.9968 to 0.9985), indicating excellent reliability. The COV% remained low, between 0.24% and 0.36%, suggesting minimal variability (Table 2).

Measurements in the 2–5 mm and 5–7 mm zones showed slightly higher variability compared to central pachymetry. For example, Pachymetry_S_2–5 mm had mean values of 570.20 μm and 569.68 μm for Operators 1 and 2, with SDs of 33.88 μm and 33.55 μm, respectively. The reproducibility mean was 569.94 μm with an SD of 33.64 μm. Despite the higher variability, the ICC values remained high (0.9911 to 0.9913), indicating robust consistency. The COV% ranged from 0.56% to 0.74%, slightly higher than central measurements.

The central epithelium thickness showed high repeatability and reproducibility. The mean values for the central epithelium were 55.83 μm for both operators, with SDs of 4.28 μm and 4.24 μm. The reproducibility mean was 55.83 μm with an SD of 4.25 μm. The ICC values ranged from 0.9247 to 0.9455, indicating very good reliability. The COV% values were higher compared to pachymetry, between 1.76% and 2.30%.

Peripheral epithelium measurements showed increased variability compared to the central epithelium. For example, Epithelium_S_2–5 mm had mean values of 54.15 μm for both operators, with SDs of 4.12 μm and 3.98 μm, respectively. The reproducibility mean was 54.15 μm with an SD of 4.04 μm. ICC values ranged from 0.9127 to 0.9156, indicating good reliability. The COV% values ranged from 2.18% to 2.77.

The central stroma measurements (Stroma_Min, Stroma_Median, Stroma_Central) demonstrated high repeatability and reproducibility. For instance, Stroma_Min had mean values of 475.78 μm and 475.55 μm for Operators 1 and 2, with corresponding SDs of 30.83 μm and 30.81 μm. The reproducibility mean was 475.66 μm with an SD of 30.75 μm. The ICC values were extremely high (0.9986 to 0.9997). The COV% remained very low, between 0.12% and 0.25%.

Measurements in the 2–5 mm and 5–7 mm zones of the stroma showed slightly higher variability compared to central measurements. For example, Stroma_S_2–5 mm had mean values of 516.06 μm and 515.53 μm for Operators 1 and 2, with SDs of 33.03 μm and 32.70 μm, respectively. The reproducibility mean was 515.79 μm with an SD of 32.79 μm. The ICC values remained high (0.9919 to 0.9925), indicating robust consistency. The COV% values ranged from 0.56% to 0.65%.

## 4. Discussion

In this study, we evaluated the intra-examiner repeatability, inter-examiner reproducibility, and inter-device agreement of corneal surface measurements among healthy patients using two AS-OCT devices: the OCT REVO 130 and the Optovue XR Avanti. Overall, the results showed high repeatability and reproducibility across all measurement parameters for both operators, with ICC values consistently exceeding 0.98 for most metrics, indicating excellent reliability in the measurements.

The superior axial resolution of OCT devices enhances image quality, allowing for greater detail in visualizing structures and enabling clear distinction of the corneal layers [20]. High image quality can facilitate more reliable and precise automated image processing, including the segmentation of relevant layers. In high-resolution OCT images, the Bowman’s layer is distinctly visible, and the clarity of this layer may serve as an indicator of image quality, potentially linked to the axial resolution of the OCT device [21].

Central measurements of epithelium, stroma, and pachymetry exhibited less variation compared to their peripheral measurements—the highest ICC values were 0.9247, 0.9997, 0.9985 and 0.9156, 0.9925, 0.9913, respectively. This was also reflected in the COV% values, which, in central zones were 2.30%, 0.25%, and 0.36%, compared to 2.77%, 0.65%, and 0.74%, respectively in peripheral zones.

The Avanti device tends to produce slightly higher standard deviations, particularly for epithelial and stromal thickness measurements, indicating higher variability compared to the REVO device. The main reason is the way Optovue and Revo segment (detect) corneal boundaries. It seems that Optovue interpolates boundaries (often visible on the edge difference between the segmentation line and the pixels representing the corneal boundaries) versus the more precise pixel-by-pixel detection in REVO. Additional conditions might be image resolution and scan speed. Avanti composes images with 1020 A-scan, whereas REVO has 2560 A-scan. REVO FC 130 has a scan speed of 130,000 A-scan/sec, whereas Optovue has a speed of 70,000.

Mansoori et al. [22] observed similar results in RTX Avanti pachymetry measurements, noting lower ICC values in the peripheral parts of the cornea. They proposed unique properties of the cornea, the number of measurement points in each zone, and the spacing of radial lines may be the reason for more variable results in the periphery. The OCT may be more accurate in central measurements because of its greater eye movement artifact compensation in this area. Fixational eye movements continue to be a significant source of artifacts in optical coherence tomography (OCT) images, even with advancements in acquisition speeds [23]. The number of measured points decreases from the center to the periphery of the cornea, where the radial lines are more spaced, and more points are missed.

Similar problems with measurements in the peripheral zone (>7 mm diameter) were observed in [24], where the thickness measurement was not reliable due to the weaker device signal.

Significant alterations are observed in the shape of the peripheral cornea. Generally, the peripheral cornea becomes noticeably flatter and slightly less astigmatic compared to the central cornea [25]. The corneal stromal thickness increases at the periphery due to a thickening of the peripheral posterior lamellae [26].

The epithelium layer measurement seemed to be the biggest challenge, which is reflected in the high COV% values (range 2.30% to 2.77%). Current evidence consistently supports a thickness of approximately 3 μm for the human precorneal tear film [27], and it is even the greatest deficiency among patients with corneal disease, e.g., keratoconus [28]. Both devices have a tissue resolution of 5 μm, which may explain the false inclusion of the tear film layer in the measured thickness of the corneal epithelium. The accumulation of tear film in the folds of the conjunctival sac, along with incomplete eyelid opening, leads to abnormal image segmentation at the corneal periphery [29]. Optical coherence tomography (OCT) has emerged as a valuable tool in ophthalmology, offering non-invasive, high-resolution imaging of ocular structures. In particular, OCT has proven effective in mapping corneal thickness, a critical parameter in diagnosing and managing various corneal diseases. While traditional methods like VHF-US (very high-frequency ultrasound) have been used for corneal thickness measurements, OCT offers several advantages.

The accurate measurement of central corneal thickness (CCT) is a crucial component of ophthalmic practice. It plays a significant role in the diagnosis, management, and surgical planning of various corneal diseases.

Irregularities in corneal thickness and structure can be indicative of underlying pathologies. Keratoconus, a progressive corneal disorder, is characterized by a thinning of the cornea, often accompanied by a conical protrusion. CCT mapping can reveal the characteristic “bull’s eye” pattern associated with keratoconus, aiding in early diagnosis. Subclinical keratoconus and forme fruste keratoconus, early stages of the disease, can be difficult to detect with traditional methods. CCT mapping can help identify subtle changes, such as localized thinning, which may suggest the presence of these conditions [30].

Several external factors can influence corneal thickness. The long-term wear of contact lenses, especially orthokeratology lenses, can alter corneal curvature and thickness. Certain eye drops can affect CCT. Ocular diseases, such as dry eye and allergic conjunctivitis, can influence corneal thickness. Systemic diseases, including diabetes, lupus, and Graves’ disease, may also affect CCT.

OCT provides higher-resolution images, allowing for more precise measurements of corneal layers, especially the epithelium. It is also a non-contact technique, making it more comfortable for patients. This study [31] examined morphological differences in conjunctival lesions using high-resolution AS-OCT, focusing on epithelial thickness and the extent of lesion spread on the corneal surface. These parameters were compared between two clinically suspected groups: pterygium and corneo-conjunctival intraepithelial neoplasia (CIN). The findings demonstrated that OCT is an effective tool for differentiating between pterygium and CIN, providing distinctive morphological features. An epithelial thickness exceeding 141 μm on AS-OCT was associated with 100% sensitivity and specificity for diagnosing CIN.

However, OCT may have limitations, such as a potential variability in image analysis algorithms [3].

A key limitation of this study is that all participants were healthy individuals with no known corneal abnormalities. As a result, the findings cannot be directly extrapolated to individuals with corneal pathologies such as keratoconus, peripheral thinning disorders, infections, or dystrophies. The repeatability and agreement of pachymetry and epithelial thickness measurements may differ in eyes with pathological changes due to altered corneal structure and potential segmentation challenges. Future research should focus on evaluating the reliability of these measurements in patients with corneal diseases to determine the clinical applicability of these devices in such cases.

The next limitation of this study is that it only considers ophthalmic conditions when classifying a patient as healthy, without accounting for general systemic diseases. It has been demonstrated that systemic conditions can influence corneal thickness and the characteristics of its various layers. In diabetes mellitus [32], the central corneal epithelial thickness seemed to be thinner compared to healthy patients (*p* = 0.003). Patients with hyperthyroidism were found to have thicker corneas than individuals with normal thyroid function [33].

**Figure 2:**
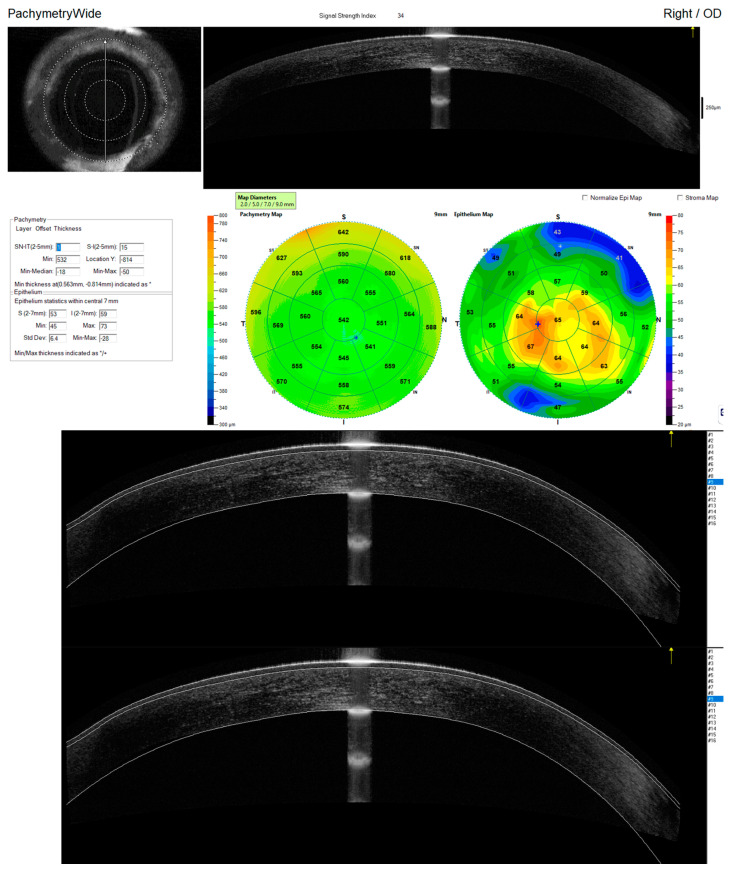
An anterior segment OCT from Optovue Avanti showed artefactual increase of the epithelial thickness, possibly due to a reflectivity of a tear film.

The repeatability of epithelial thickness mapping (ETM) is critical for the accurate assessment of corneal epithelial conditions. Our findings align with existing literature highlighting that repeatability is influenced by multiple interdependent factors, categorized as device-related, patient-related, and procedural. The precision of corneal measurement is heavily influenced by the technology utilized (Figure 2). High-resolution devices, such as very high-frequency ultrasound (VHF-US), achieve an axial resolution of approximately 1 μm, enabling more detailed assessments of epithelial thickness compared to spectral-domain OCT (SD-OCT), which offers 3.6–5 μm resolution. However, VHF-US requires prolonged acquisition times and immersion techniques, which may introduce motion artifacts and patient discomfort [34].

Scan diameter also affects repeatability, with larger scan areas (e.g., 10 mm) providing more comprehensive corneal assessments but potentially reducing measurement consistency in the peripheral zones. Furthermore, algorithmic variations between devices, such as those used by PachymetryWide and Pachymetry + Cpwr scan patterns, contribute to variability, emphasizing the importance of standardizing imaging protocols. Patient-specific characteristics, including ocular stability and tear film integrity, play a significant role in ETM repeatability. For instance, involuntary eye movements during imaging can lead to errors, particularly in devices lacking advanced tracking systems. Moreover, the inclusion of tear film thickness in CET measurements by OCT devices introduces variability, especially in patients with dry eye or other tear film abnormalities.

Diseases such as keratoconus and post-surgical states like LASIK or PRK further complicate measurements. In these cases, epithelial remodeling leads to heterogeneous thickness profiles, reducing repeatability. This underscores the need for tailored imaging strategies in diseased and post-surgical corneas.

Repeatability is also influenced by procedural elements, including patient positioning and operator expertise. Proper alignment ensures consistent imaging, particularly in peripheral zones where variability is highest. Operator training and standardization of procedures are critical to minimizing inconsistencies.

Our findings highlight the need for zone-specific considerations when interpreting ETM data. Central corneal measurements exhibit higher repeatability than peripheral zones, which are more susceptible to artifacts and variability. This underscores the importance of integrating advanced tracking systems and optimized algorithms in future device development [35]. Additionally, environmental conditions such as temperature and humidity, while not extensively studied, may influence tear film dynamics and thereby affect ETM. Standardizing imaging environments may further enhance reliability [36].

By understanding these factors, clinicians can make more informed decisions, improving diagnostic accuracy and monitoring in conditions such as keratoconus, post-refractive surgery irregularities, and dry eye syndrome. Future studies should aim to refine imaging techniques, explore the impact of environmental conditions, and validate the clinical applicability of emerging technologies.

A promising area of research would be to examine corneal parameters in patients who are ophthalmically healthy but suffer from systemic conditions such as diabetes or hypertension.

## 5. Conclusions

This study demonstrated that both the OCT REVO FC 130 and Optovue RTV XR Avanti provide high reliability and precision for corneal thickness measurements, with superior performance in central zones compared to peripheral ones. The REVO exhibited slightly better repeatability and reproducibility, particularly for central pachymetry and stromal thickness, while the Optovue showed marginally higher variability in peripheral and epithelial measurements. Despite these differences, the devices showed strong agreement. However, caution is advised when using these devices interchangeably due to possible segmentation errors. Peripheral and epithelial measurements remain more variable due to anatomical and segmentation challenges, highlighting areas for future improvement. These findings affirm the value of these devices for consistent corneal assessment and emphasize the importance of standardized protocols and careful data interpretation. Further research is necessary to establish the repeatability and reproducibility of measurements in healthy individuals.

## Figures and Tables

**Table 1:** Mean measurements of corneal layers by device and location.

	Mean Measurement					Mean Measurement					Mean Measurement			
	Avanti Mean	Avanti SD	REVO Mean	REVO SD		Avanti Mean	Avanti SD	REVO Mean	REVO SD		Avanti Mean	Avanti SD	REVO Mean	REVO SD
2 mm Epi_2 Central	55.2	4.26	55.55	4.28	2 mm Pachy_2 Central	536.56	24.19	535.85	24.13	2 mm Stroma_2 Central	481.36	23.72	480.29	23.56
2–5 mm Epi_I_2_5	56.43	4.35	55.99	4.2	2–5 mm Pachy_I_2_5	556.54	24.38	548.89	25.09	2–5 mm Stroma_I_2_5	500.11	23.88	492.9	24.42
2–5 mm Epi_IN_2_5	55.87	3.96	55.69	4.08	2–5 mm Pachy_IN_2_5	563.43	23.55	557.42	24.67	2–5 mm Stroma_IN_2_5	507.56	23.39	501.72	24.14
2–5 mm Epi_IT_2_5	55.67	4.54	55.3	4.41	2–5 mm Pachy_IT_2_5	547.15	24.89	539.15	25.23	2–5 mm Stroma_IT_2_5	491.48	24.26	483.85	24.37
2–5 mm Epi_S_2_5	54.02	3.47	53.96	3.61	2–5 mm Pachy_N_2_5	567.9	24.01	563.87	24.52	2–5 mm Stroma_N_2_5	512.92	23.65	508.92	24.12
2–5 mm Epi_SN_2_5	54.54	3.62	54.43	3.58	2–5 mm Pachy_S_2_5	570.9	27.15	566.36	25.83	2–5 mm Stroma_S_2_5	516.88	26.56	512.4	25.22
2–5 mm Epi_ST_2_5	53.87	3.66	53.99	3.72	2–5 mm Pachy_SN_2_5	572.9	25.78	569.59	25.06	2–5 mm Stroma_SN_2_5	518.36	25.29	515.16	24.72
2–5 mm Epi_T_2_5	54.67	4.06	54.28	4.05	2–5 mm Pachy_ST_2_5	557.11	27.3	551.5	25.82	2–5 mm Stroma_ST_2_5	503.24	26.76	497.51	25.06
2–5 mmEpi_N_2_5	54.98	3.73	54.96	3.98	2–5 mm Pachy_T_2_5	545.37	25.96	538.02	25.27	2–5 mm Stroma_T_2_5	490.69	25.45	483.74	24.49
5–7 mm Epi_I_5_7	56.44	3.67	55.93	3.15	5–7 mm Pachy_I_5_7	592.19	26.14	586.41	26.86	5–7 mm Stroma_I_5_7	535.75	25.63	530.48	26.23
5–7 mm Epi_IN_5_7	55.58	3.13	55.74	2.96	5–7 mm Pachy_IN_5_7	600.8	26.07	598.77	26.43	5–7 mm Stroma_IN_5_7	545.21	25.78	543.03	25.95
5–7 mm Epi_IT_5_7	56.15	3.88	55.45	3.55	5–7 mm Pachy_IT_5_7	575.17	26.47	568.25	26.39	5–7 mm Stroma_IT_5_7	519.01	25.86	512.8	25.75
5–7 mm Epi_N_5_7	55.14	3.45	55.6	3.23	5–7 mm Pachy_N_5_7	605.71	25.63	606.3	25.9	5–7 mm Stroma_N_5_7	550.57	25.43	550.69	25.44
5–7 mm Epi_S_5_7	51.82	3.32	52.15	2.64	5–7 mm Pachy_S_5_7	615.73	29.77	614.67	27.68	5–7 mm Stroma_S_5_7	563.91	29.65	562.52	27.64
5–7 mm Epi_SN_5_7	54.05	3.25	54.31	2.94	5–7 mm Pachy_SN_5_7	615.9	27.4	617.31	26.31	5–7 mm Stroma_SN_5_7	561.86	27.45	563	26.28
5–7 mm Epi_ST_5_7	52.47	3.2	53.05	2.54	5–7 mm Pachy_ST_5_7	591.68	30.16	588.45	26.7	5–7 mm Stroma_ST_5_7	539.21	29.83	535.4	26.68
5–7 mm Epi_T_5_7	54.05	3.34	54.01	3.12	5–7 mm Pachy_T_5_7	568.76	27.6	562.59	25.79	5–7 mm Stroma_T_5_7	514.72	27.31	508.58	25.44
Epi_Median	53.13	3.6	54.69	3.09	Pachy_Median	554.92	24.55	578.96	25.4	Stroma_Median	519.16	24.88	524.23	25.08
Epi_Min	48.85	2.93	48.15	2.42	Pachy_Min	530.18	24.09	527.53	24.55	Stroma_Min	474.96	23.69	472.47	23.79

**Table 2:** Corneal biometric measurements: repeatability and reproducibility.

Parameter Name	Repeatability Operator 1						Repeatability Operator 2						Reproducibility					
	Mean	SD	SW	TRT	COV%	ICC	Mean	SD	SW	TRT	COV%	ICC	Mean_Rep	SD_Rep	SW_Rep	TRT_Rep	COV_Rep	ICC_Rep
Pachymetry																		
Pachymetry_Min	531.14	31.97	1.84	5.1	0.35	0.9968	531.01	31.88	1.25	3.47	0.24	0.9985	531.08	31.85	1.92	5.32	0.36	0.9976
Pachymetry_Median	581.15	31.85	2.39	6.63	0.41	0.9945	580.77	31.88	1.9	5.27	0.33	0.9965	580.96	31.8	2.52	7	0.43	0.9955
Pachymetry_Central	539.45	31.99	1.8	4.98	0.33	0.9969	539.3	31.77	1.55	4.31	0.29	0.9977	539.38	31.81	1.87	5.17	0.35	0.9973
Pachymetry 2–5 mm																		
Pachymetry_S_2–5 mm	570.2	33.88	3.2	8.88	0.56	0.9913	569.68	33.55	3.2	8.86	0.56	0.9911	569.94	33.64	3.69	10.24	0.65	0.9912
Pachymetry_SN_2–5 mm	573.92	33.26	3.69	10.24	0.64	0.9878	573.62	32.7	3.92	10.85	0.68	0.986	573.77	32.91	4.24	11.75	0.74	0.9869
Pachymetry_N_2–5 mm	567.73	32.14	3.3	9.15	0.58	0.9897	567.76	31.9	3.58	9.93	0.63	0.9877	567.74	31.95	3.87	10.72	0.68	0.9887
Pachymetry_IN_2–5 mm	560.87	31.65	2.6	7.2	0.46	0.9935	561.31	31.51	2.96	8.22	0.53	0.9914	561.09	31.51	3.1	8.6	0.55	0.9924
Pachymetry_I_2–5 mm	552.4	31.53	2.19	6.06	0.4	0.9953	552.65	31.49	1.99	5.53	0.36	0.9961	552.52	31.44	2.42	6.71	0.44	0.9957
Pachymetry_IT_2–5 mm	542.86	31.6	2.42	6.7	0.45	0.9943	542.67	31.56	1.69	4.7	0.31	0.9972	542.76	31.51	2.53	7	0.47	0.9958
Pachymetry_T_2–5 mm	541.58	32.42	2.41	6.67	0.44	0.9947	541.02	32.71	1.77	4.92	0.33	0.9971	541.3	32.5	2.53	7.01	0.47	0.9959
Pachymetry_ST_2–5 mm	555.08	33.6	2.74	7.6	0.49	0.9935	554.42	33.73	2.09	5.79	0.38	0.9962	554.75	33.59	2.86	7.94	0.52	0.9949
Pachymetry 5–7 mm																		
Pachymetry_S_5–7 mm	616.33	35.17	4.36	12.09	0.71	0.9852	615.72	33.98	4.48	12.42	0.73	0.9833	616.02	34.49	5.48	15.19	0.89	0.9837
Pachymetry_SN_5–7 mm	619.12	34.12	4.48	12.41	0.72	0.9838	620.87	34.26	5.41	15	0.87	0.9757	620.01	34.13	6.08	16.84	0.98	0.9792
Pachymetry_N_5–7 mm	608.87	33.73	4.33	12.01	0.71	0.984	609.05	33.16	5.23	14.5	0.86	0.9759	608.96	33.37	5.49	15.22	0.9	0.98
Pachymetry_IN_5–7 mm	600.19	33.34	3.56	9.87	0.59	0.989	600.84	32.77	4.4	12.19	0.73	0.9827	600.52	32.98	4.55	12.62	0.76	0.9859
Pachymetry_I_5–7 mm	587.91	32.43	2.83	7.85	0.48	0.9926	587.64	32.47	2.98	8.27	0.51	0.9919	587.78	32.38	3.36	9.3	0.57	0.9923
Pachymetry_IT_5–7 mm	570.73	31.13	3.45	9.56	0.6	0.988	570.78	31.72	3.54	9.82	0.62	0.9877	570.76	31.36	4.2	11.64	0.74	0.9879
Pachymetry_T_5–7 mm	565.94	32.37	3.67	10.17	0.65	0.9874	565.44	32.94	3.51	9.73	0.62	0.9888	565.69	32.59	4.17	11.55	0.74	0.9881
Pachymetry_ST_5–7 mm	590.2	33.09	3.74	10.36	0.63	0.9879	590.05	34.16	3.41	9.44	0.58	0.9901	590.13	33.56	4.17	11.57	0.71	0.989
Epithelium																		
Epithelium_Central	55.83	4.28	1.18	3.26	2.11	0.9247	55.84	4.24	0.98	2.72	1.76	0.9455	55.83	4.25	1.29	3.56	2.3	0.9348
Epithelium 2–5 mm																		
Epithelium_S_2–5 mm	54.15	4.12	1.21	3.37	2.24	0.9156	54.15	3.98	1.18	3.28	2.18	0.9127	54.15	4.04	1.5	4.16	2.77	0.9141
Epithelium_SN_2–5 mm	55.05	4.11	1.3	3.59	2.35	0.9024	54.87	3.91	1.09	3.03	1.99	0.9221	54.96	4.01	1.47	4.08	2.68	0.9119
Epithelium_N_2–5 mm	55.45	4.19	1.32	3.65	2.37	0.9026	55.28	4.13	1.03	2.87	1.87	0.9368	55.37	4.15	1.4	3.88	2.53	0.9192
Epithelium_IN_2–5 mm	56.14	4.45	1.3	3.6	2.31	0.9174	56.12	4.37	1.23	3.41	2.19	0.9206	56.13	4.4	1.52	4.21	2.7	0.919
Epithelium_I_2–5 mm	56.34	4.62	1.31	3.62	2.32	0.9233	56.34	4.4	1.12	3.09	1.98	0.9376	56.34	4.5	1.47	4.06	2.6	0.93
Epithelium_IT_2–5 mm	55.61	4.63	1.23	3.41	2.21	0.932	55.59	4.43	1.02	2.83	1.84	0.9484	55.6	4.52	1.42	3.94	2.56	0.9398
Epithelium_T_2–5 mm	54.33	4.17	1.14	3.15	2.09	0.9285	54.25	4.1	0.99	2.75	1.83	0.9429	54.29	4.13	1.44	3.98	2.64	0.9355
Epithelium_ST_2–5 mm	53.93	4.09	1.2	3.33	2.23	0.917	53.85	3.95	1.09	3.01	2.02	0.9251	53.89	4.02	1.52	4.2	2.81	0.9208
Epithelium 5–7 mm																		
Epithelium_S_5–7 mm	52.47	3.65	1.15	3.18	2.18	0.9047	52.9	3.28	1.09	3.03	2.07	0.8871	52.69	3.47	1.37	3.8	2.6	0.894
Epithelium_SN_5–7 mm	54.42	3.45	1.06	2.93	1.94	0.9082	54.64	3.1	0.87	2.41	1.59	0.924	54.53	3.27	1.12	3.1	2.05	0.9149
Epithelium_N_5–7 mm	55.48	3.27	0.87	2.4	1.56	0.9325	55.28	3.24	0.77	2.13	1.39	0.9454	55.38	3.25	0.99	2.75	1.79	0.9389
Epithelium_IN_5–7 mm	55.7	3.4	0.97	2.69	1.75	0.9216	55.57	3.28	0.76	2.1	1.36	0.9486	55.63	3.33	0.98	2.71	1.76	0.9347
Epithelium_I_5–7 mm	55.83	3.71	1.05	2.91	1.88	0.9229	55.73	3.31	0.63	1.75	1.14	0.965	55.78	3.51	0.97	2.68	1.74	0.9416
Epithelium_IT_5–7 mm	55.48	3.39	0.78	2.16	1.41	0.9493	55.29	3.47	0.75	2.09	1.36	0.9546	55.39	3.42	0.86	2.39	1.56	0.952
Epithelium_T_5–7 mm	53.87	3.21	0.64	1.77	1.18	0.9619	53.69	3.4	0.8	2.22	1.49	0.9461	53.78	3.3	0.93	2.59	1.74	0.9537
Epithelium_ST_5–7 mm	53.11	3.59	0.78	2.15	1.46	0.9529	53.18	3.51	0.9	2.5	1.69	0.9321	53.14	3.54	0.98	2.71	1.84	0.9419
Stroma																		
Stroma_Min	475.78	30.83	1.18	3.28	0.25	0.9986	475.55	30.81	0.57	1.57	0.12	0.9997	475.66	30.75	1.2	3.32	0.25	0.9991
Stroma_Median	526.33	31.53	2.1	5.82	0.4	0.9957	526.07	31.6	1.75	4.84	0.33	0.997	526.2	31.5	2.27	6.3	0.43	0.9964
Stroma_Central	483.63	30.89	1.34	3.73	0.28	0.9982	483.46	30.73	1.07	2.97	0.22	0.9988	483.54	30.74	1.41	3.91	0.29	0.9985
Stroma 2–5 mm																		
Stroma_S_2–5 mm	516.06	33.03	3	8.31	0.58	0.9919	515.53	32.7	2.86	7.93	0.56	0.9925	515.79	32.79	3.34	9.27	0.65	0.9922
Stroma_SN_2–5 mm	518.87	32.43	3.5	9.7	0.67	0.9885	518.75	31.99	3.66	10.13	0.7	0.9873	518.81	32.13	3.83	10.63	0.74	0.9879
Stroma_N_2–5 mm	512.27	31.65	3.19	8.84	0.62	0.9902	512.48	31.37	3.35	9.29	0.65	0.9889	512.38	31.44	3.51	9.73	0.69	0.9896
Stroma_IN_2–5 mm	504.73	31.38	2.3	6.38	0.46	0.9948	505.19	31.22	2.63	7.3	0.52	0.9931	504.96	31.23	2.74	7.59	0.54	0.994
Stroma_I_2–5 mm	496.06	31.24	1.61	4.45	0.32	0.9974	496.3	31.17	1.73	4.81	0.35	0.997	496.18	31.14	1.99	5.52	0.4	0.9972
Stroma_IT_2–5 mm	487.25	31.02	1.78	4.94	0.37	0.9968	487.08	30.84	1.47	4.07	0.3	0.9978	487.16	30.86	2.11	5.84	0.43	0.9973
Stroma_T_2–5 mm	487.25	31.47	1.98	5.5	0.41	0.9962	486.77	31.67	1.68	4.65	0.34	0.9973	487.01	31.5	2.16	5.98	0.44	0.9967
Stroma_ST_2–5 mm	501.15	32.56	2.41	6.68	0.48	0.9947	500.57	32.67	1.95	5.42	0.39	0.9965	500.86	32.54	2.46	6.81	0.49	0.9956
Stroma 5–7 mm																		
Stroma_S_5–7 mm	563.86	34.49	4.14	11.49	0.74	0.9862	562.81	33.37	4.36	12.07	0.77	0.9838	563.33	33.85	5.24	14.52	0.93	0.9845
Stroma_SN_5–7 mm	564.7	33.66	4.34	12.04	0.77	0.9843	566.23	34.04	5.13	14.21	0.91	0.9779	565.48	33.78	5.78	16.02	1.02	0.9806
Stroma_N_5–7 mm	553.38	33.78	4.33	11.99	0.78	0.9841	553.77	33.27	5.05	14.01	0.91	0.9776	553.58	33.45	5.3	14.7	0.96	0.9809
Stroma_IN_5–7 mm	544.5	33.42	3.47	9.61	0.64	0.9896	545.27	32.88	4.31	11.94	0.79	0.9835	544.89	33.08	4.46	12.36	0.82	0.9866
Stroma_I_5–7 mm	532.09	32.48	2.48	6.89	0.47	0.9943	531.91	32.44	2.88	7.99	0.54	0.9924	532	32.39	3.21	8.9	0.6	0.9934
Stroma_IT_5–7 mm	515.25	31.18	3.29	9.12	0.64	0.9891	515.48	31.59	3.5	9.71	0.68	0.9879	515.36	31.32	4.09	11.33	0.79	0.9885
Stroma_T_5–7 mm	512.07	31.79	3.53	9.79	0.69	0.9879	511.75	32.38	3.57	9.89	0.7	0.988	511.91	32.01	4.02	11.13	0.78	0.988
Stroma_ST_5–7 mm	537.1	32.38	3.63	10.07	0.68	0.9881	536.87	33.36	3.39	9.4	0.63	0.9897	536.98	32.8	4.08	11.3	0.76	0.9889

## Data Availability

The original contributions presented in this study are included in the article/Supplementary Materials. Further inquiries can be directed to the corresponding author.

## Supplementary Materials

The following supporting information can be downloaded at: https://www.mdpi.com/article/10.3390/jcm14041295/s1, Supplementary file: Agreement Study between Anterior Radial (REVO FC130) and (Avanti RTVue XR).
