# Supplementary material for: Variability in Corneal and Epithelial Pachymetry: A Comparison of Optopol Revo 130 and Optovue RTV XR Avanti in Healthy Patients
Source: J Clin Med. 2025 Feb 15;14(4):1295. doi: 10.3390/jcm14041295 (PMC11856664; doi:10.3390/jcm14041295)
Supplement: Supplementary file 1 [file jcm-14-01295-s001.zip › jcm-3373806-supplementary.pdf]

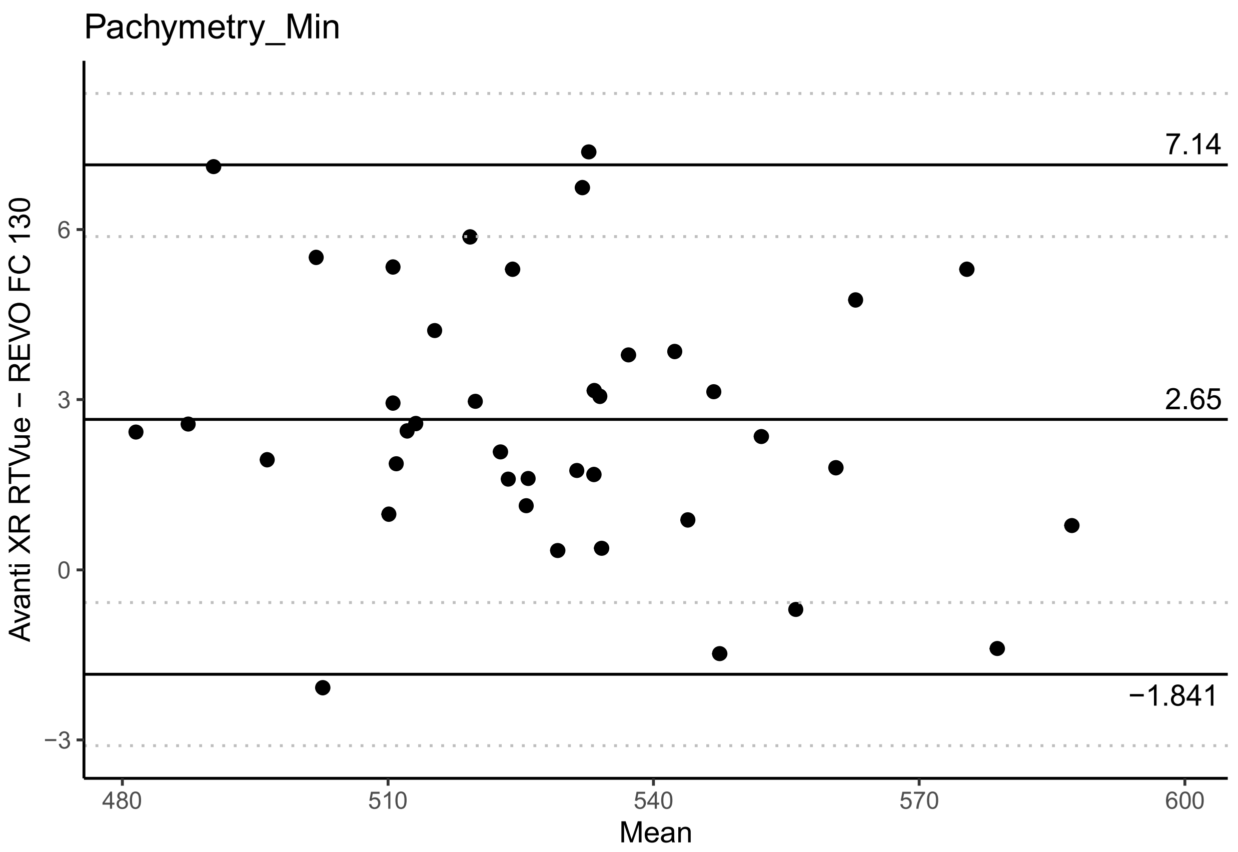

Table 1: Shapiro-Wilk Test Results

Test	W_statistic	p_value
Avanti RTVue	0.9810411	0.7281025
REVO FC 130	0.9793200	0.6642992
Differences	0.9763378	0.5559603

Table 2: Paired T-test results

	Test	t_statistic	p_value
t	Paired t-test for mean differences	7.313937	0

Table 3: Bland Altman Statistics

Sector	Mean_Diff	SD	upperLOA	lower_CI	upper_CI	lowerLOA	lower_CI_	upper_CI_
Pachymetry_Min	2.6495	2.2911	7.14	5.8773	8.4028	-1.841	-3.1038	-0.5783

Table 4: Basic Statistics

Mean_Avanti	SD1	Min1	Max1	Mean_REVO	SD2	Min2	Max2
530.1842	24.0887	482.76	587.62	527.5348	24.5502	480.33	586.84

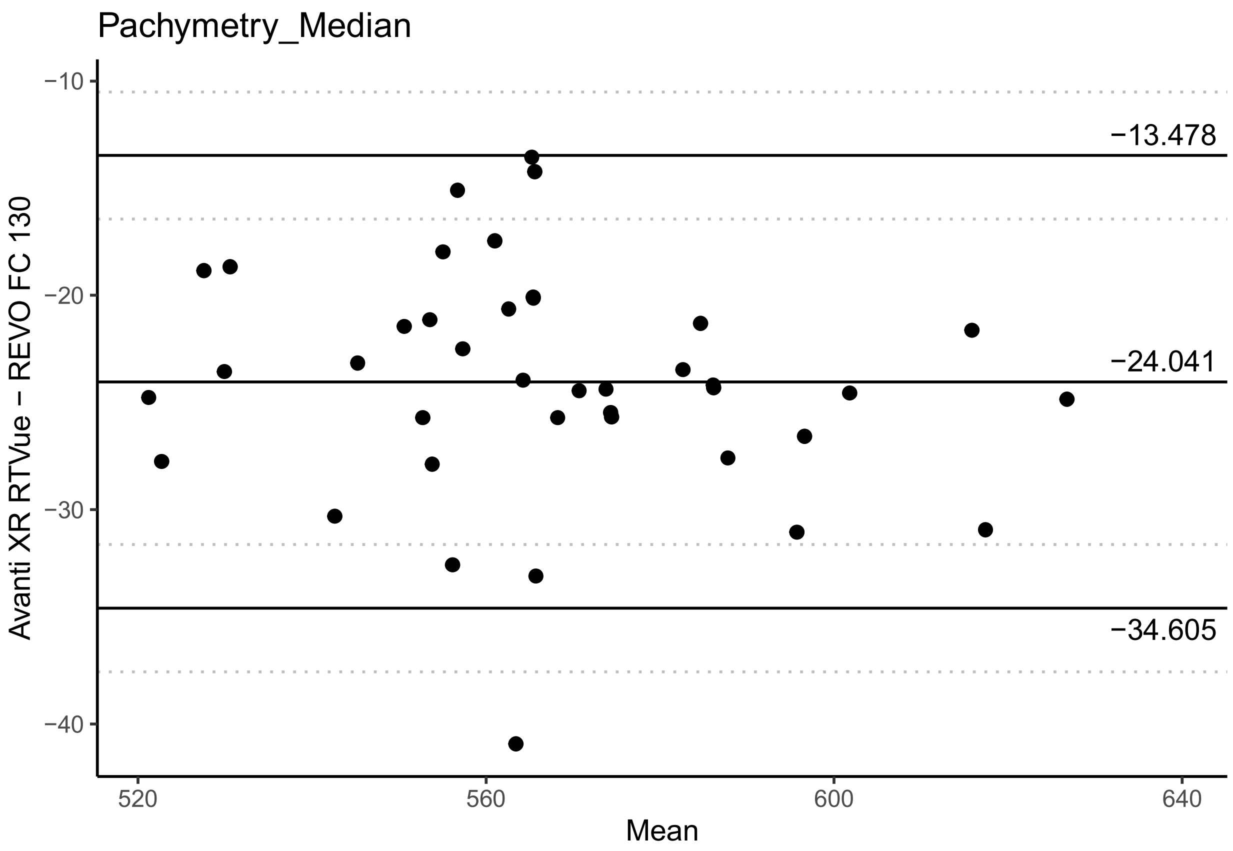

Table 5: Shapiro-Wilk Test Results

Test	W_statistic	p_value
Avanti RTVue	0.9746191	0.4972771
REVO FC 130	0.9690201	0.3349348
Differences	0.9644403	0.2368479

Table 6: Paired T-test results

	Test	t_statistic	p_value
t	Paired t-test for mean differences	-28.21263	0

Table 7: Bland Altman Statistics

Sector	Mean_Diff	SD	upperLOA	lower_CI	upper_CI	lowerLOA	lower_CI_	upper_CI_
Pachymetry_Median	-24.0413	5.3894	-13.478	-16.4484	-10.5075	-34.6045	-37.575	-31.6341

Table 8: Basic Statistics

Mean_Avanti	SD1	Min1	Max1	Mean_REVO	SD2	Min2	Max2
554.916	24.5467	508.84	614.33	578.9573	25.4027	533.61	639.18

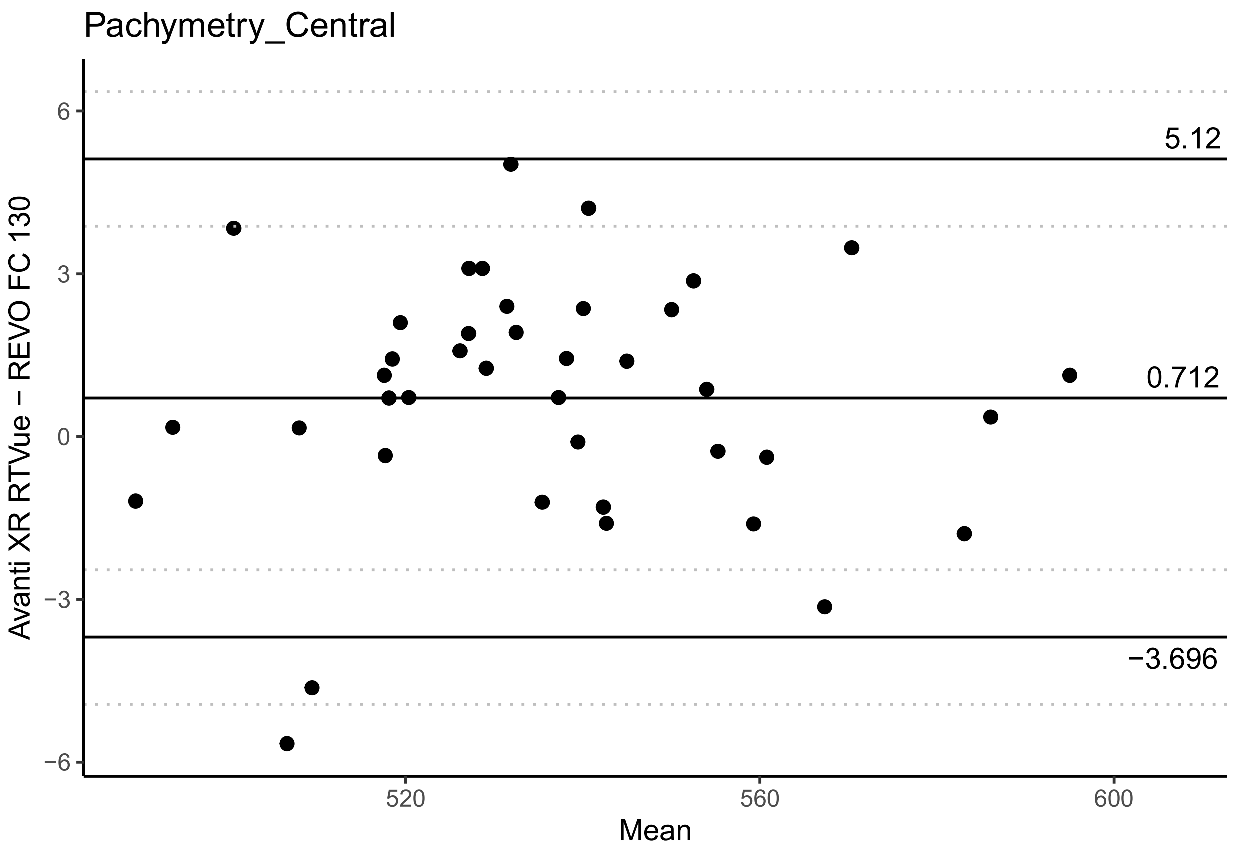

Table 9: Shapiro-Wilk Test Results

Test	W_statistic	p_value
Avanti RTVue	0.9818155	0.7564239
REVO FC 130	0.9750173	0.5105523
Differences	0.9661433	0.2698725

Table 10: Paired T-test results

	Test	t_statistic	p_value
t	Paired t-test for mean differences	2.002322	0.0522384

Table 11: Bland Altman Statistics

Sector	Mean_Diff	SD	upperLOA	lower_CI	upper_CI	lowerLOA	lower_CI_	upper_CI_
Pachymetry_Central	0.712	2.2489	5.1199	3.8804	6.3594	-3.6959	-4.9354	-2.4564

Table 12: Basic Statistics

Mean_Avanti	SD1	Min1	Max1	Mean_REVO	SD2	Min2	Max2
536.5575	24.1866	488.9	595.58	535.8455	24.1273	490.09	594.45

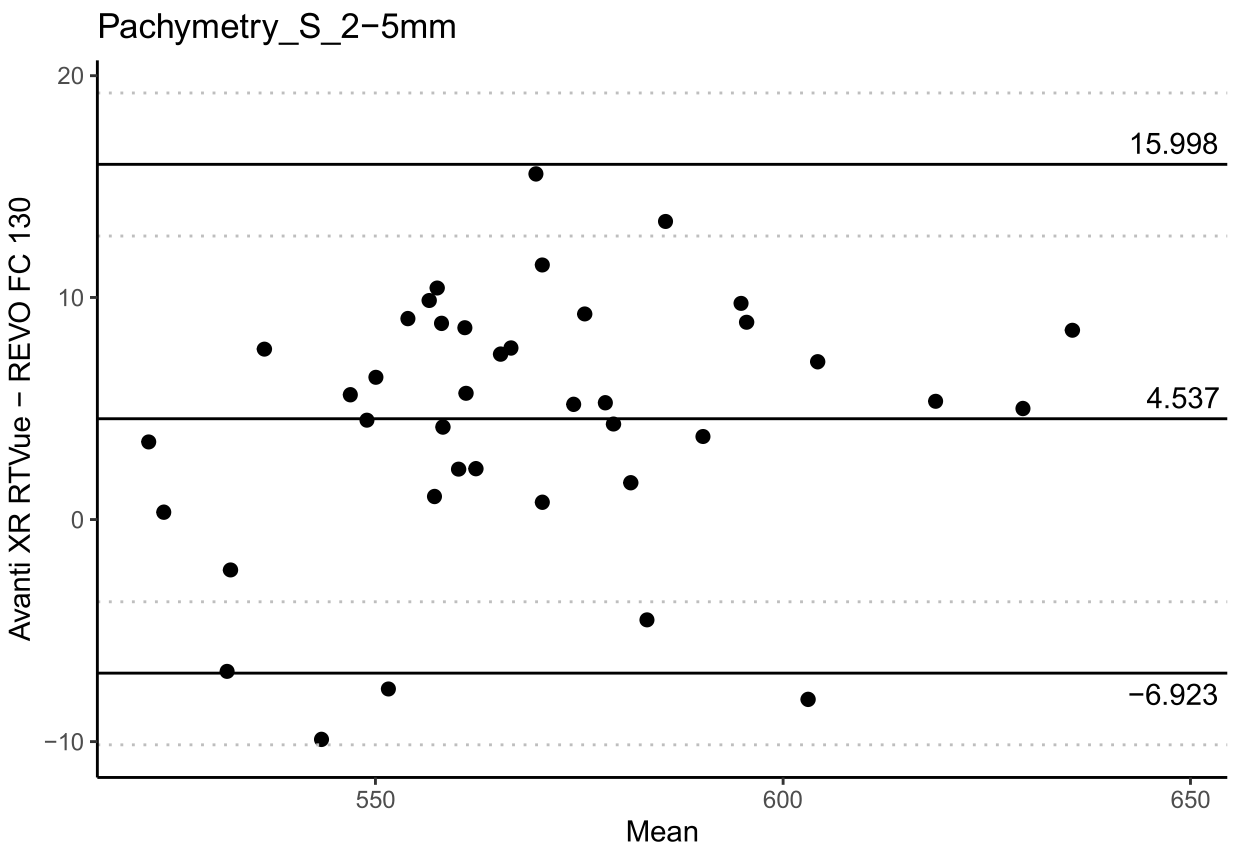

Table 13: Shapiro-Wilk Test Results

Test	W_statistic	p_value
Avanti RTVue	0.9679980	0.3104279
REVO FC 130	0.9560781	0.1228339
Differences	0.9359589	0.0253185

Table 14: Paired T-test results

	Test	t_statistic	p_value
t	Paired t-test for mean differences	4.907685	1.68e-05

Table 15: Bland Altman Statistics

Sector	Mean_Diff	SD	upperLOA	lower_CI	upper_CI	lowerLOA	lower_CI_	upper_CI_
Pachymetry_S_2-5mm	4.5373	5.8472	15.9977	12.775	19.2204	-6.9232	-10.1459	-3.7005

Table 16: Basic Statistics

Mean_Avanti	SD1	Min1	Max1	Mean_REVO	SD2	Min2	Max2
570.8978	27.1457	523.91	639.76	566.3605	25.8252	520.41	631.23

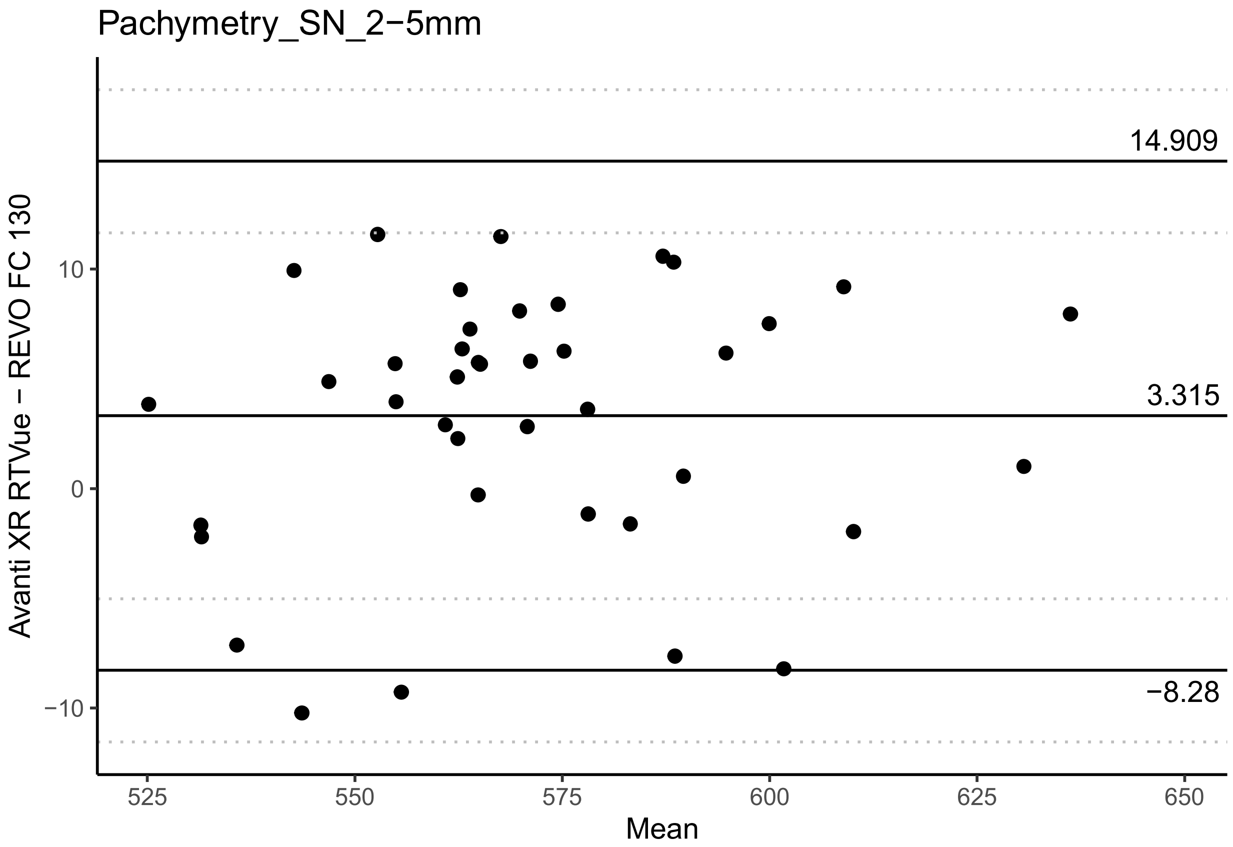

Table 17: Shapiro-Wilk Test Results

Test	W_statistic	p_value
Avanti RTVue	0.9703012	0.3678863
REVO FC 130	0.9620196	0.1962565
Differences	0.9260101	0.0119595

Table 18: Paired T-test results

	Test	t_statistic	p_value
t	Paired t-test for mean differences	3.543746	0.001042

Table 19: Bland Altman Statistics

Sector	Mean_Diff	SD	upperLOA	lower_CI	upper_CI	lowerLOA	lower_CI_	upper_CI_
Pachymetry_SN_2-5mm	3.3145	5.9154	14.9087	11.6484	18.169	-8.2797	-11.54	-5.0194

Table 20: Basic Statistics

Mean_Avanti	SD1	Min1	Max1	Mean_REVO	SD2	Min2	Max2
572.9005	25.7757	527.07	640.21	569.586	25.0596	523.23	632.26

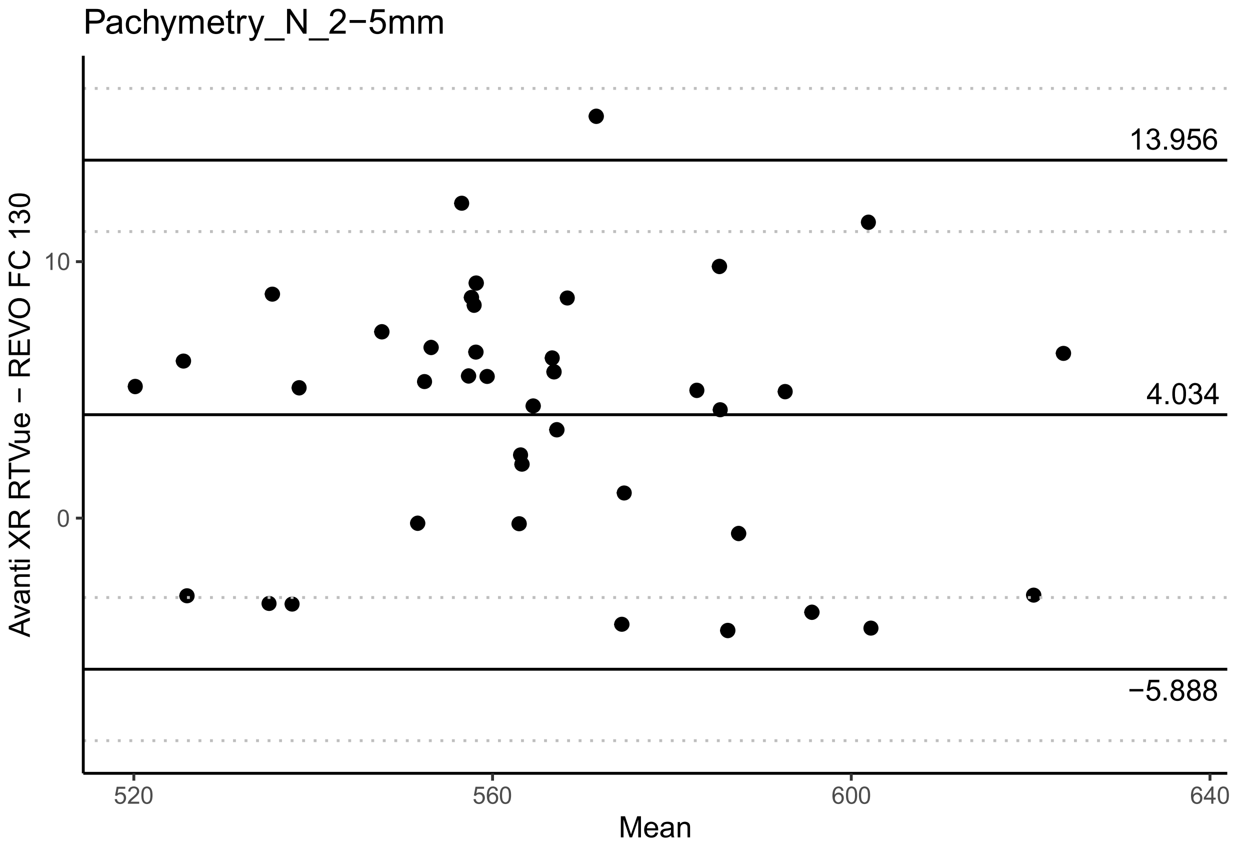

Table 21: Shapiro-Wilk Test Results

Test	W_statistic	p_value
Avanti RTVue	0.9746874	0.4995388
REVO FC 130	0.9707744	0.3806958

Test	W_statistic	p_value
Differences	0.9484874	0.0672736

Table 22: Paired T-test results

	Test	t_statistic	p_value
t	Paired t-test for mean differences	5.039712	1.1e-05

Table 23: Bland Altman Statistics

Sector	Mean_Diff	SD	upperLOA	lower_CI	upper_CI	lowerLOA	lower_CI_	upper_CI_
Pachymetry_N_2-5mm	4.034	5.0624	13.9564	11.1662	16.7466	-5.8884	-8.6786	-3.0982

Table 24: Basic Statistics

Mean_Avanti	SD1	Min1	Max1	Mean_REVO	SD2	Min2	Max2
567.9043	24.0095	522.74	626.86	563.8703	24.5209	517.61	621.83

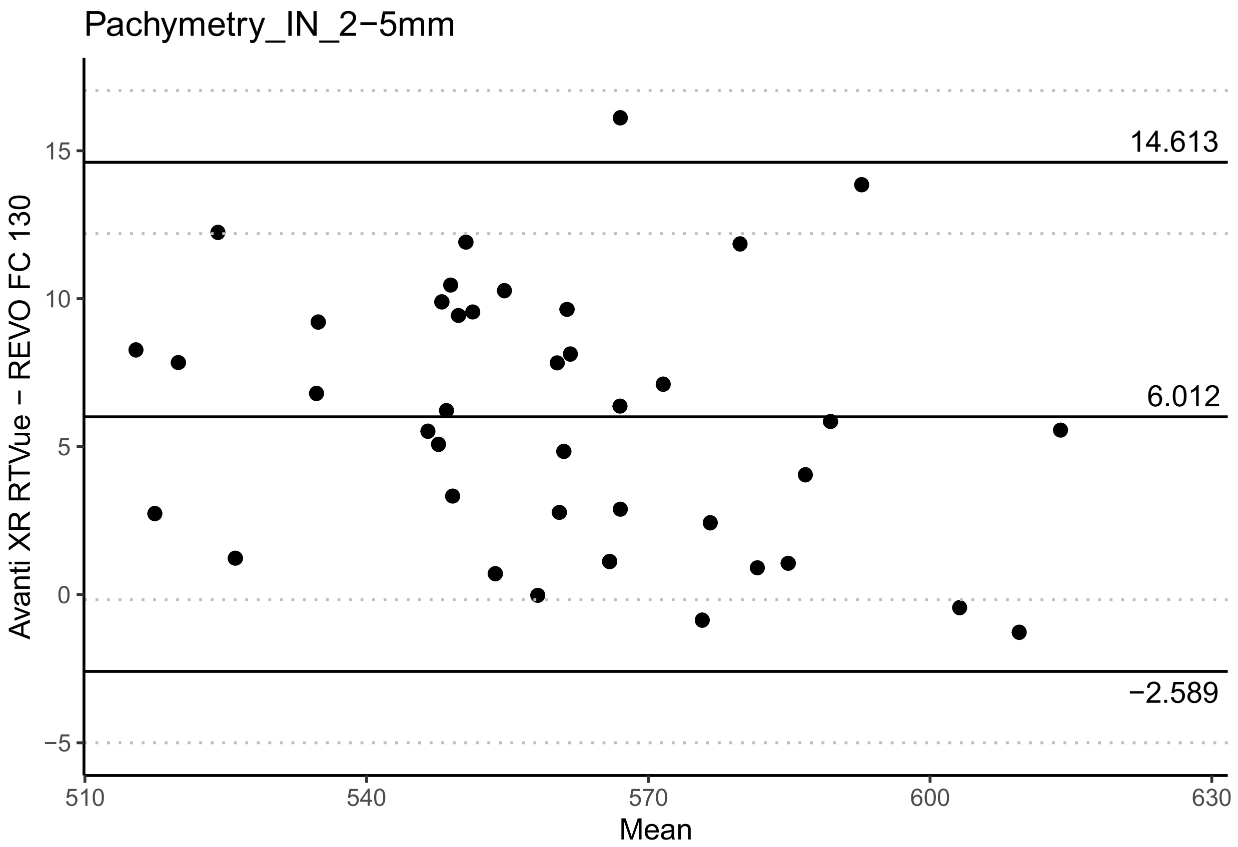

Table 25: Shapiro-Wilk Test Results

Test	W_statistic	p_value
Avanti RTVue	0.9798155	0.6826989
REVO FC 130	0.9722629	0.4232266
Differences	0.9741682	0.4824989

Table 26: Paired T-test results

	Test	t_statistic	p_value
t	Paired t-test for mean differences	8.664934	0

Table 27: Bland Altman Statistics

Sector	Mean_Diff	SD	upperLOA	lower_CI	upper_CI	lowerLOA	lower_CI_	upper_CI_
Pachymetry_IN_2-5mm	6.0122	4.3884	14.6134	12.1947	17.0321	-2.5889	-5.0076	-0.1702

Table 28: Basic Statistics

Mean_Avanti	SD1	Min1	Max1	Mean_REVO	SD2	Min2	Max2
563.4285	23.5451	518.81	616.68	557.4162	24.6669	511.3	611.12

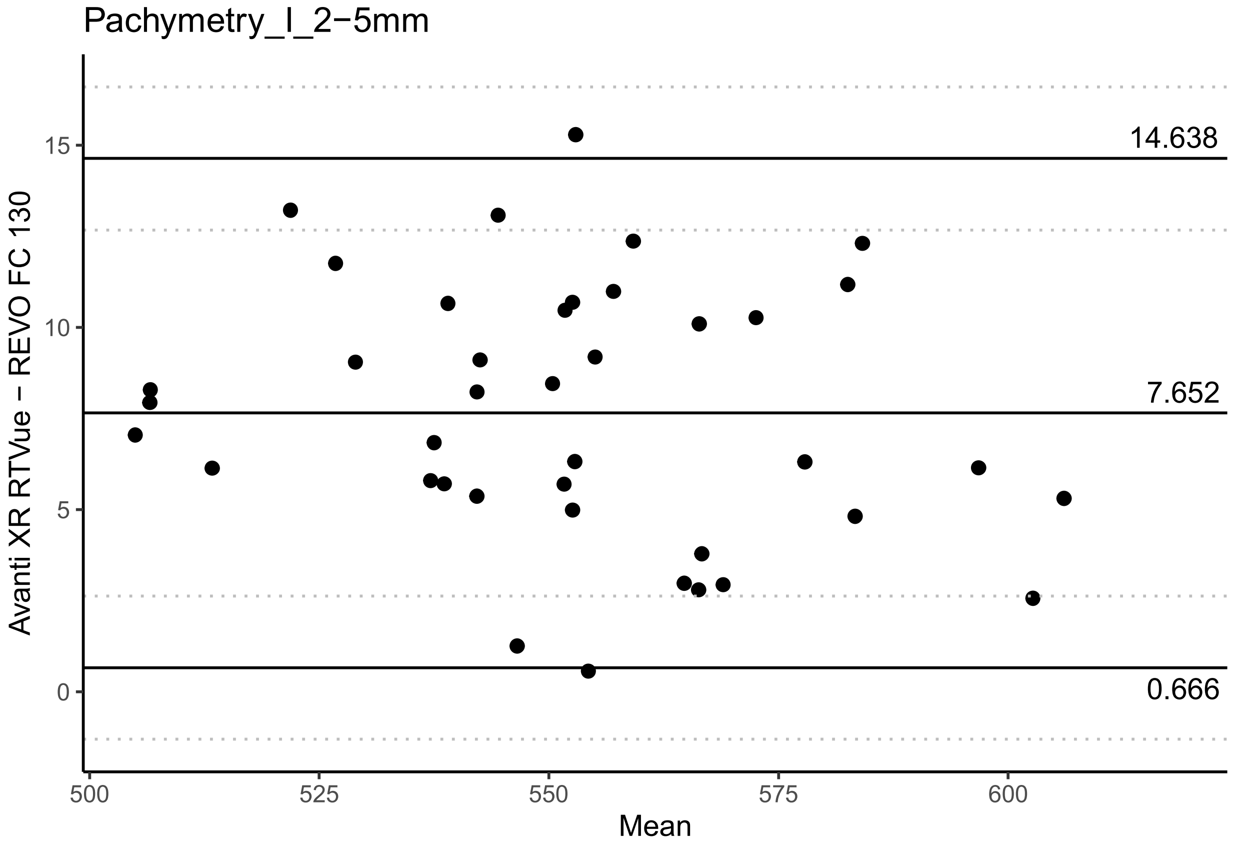

Table 29: Shapiro-Wilk Test Results

Test	W_statistic	p_value
Avanti RTVue	0.9777724	0.6073234
REVO FC 130	0.9741676	0.4824782
Differences	0.9832170	0.8061047

Table 30: Paired T-test results

	Test	t_statistic	p_value
t	Paired t-test for mean differences	13.5775	0

Table 31: Bland Altman Statistics

Sector	Mean_Diff	SD	upperLOA	lower_CI	upper_CI	lowerLOA	lower_CI_	upper_CI_
Pachymetry_I_2-5mm	7.652	3.5644	14.6382	12.6737	16.6027	0.6658	-1.2987	2.6303

Table 32: Basic Statistics

Mean_Avanti	SD1	Min1	Max1	Mean_REVO	SD2	Min2	Max2
556.542	24.3807	508.5	608.74	548.89	25.0916	501.45	603.43

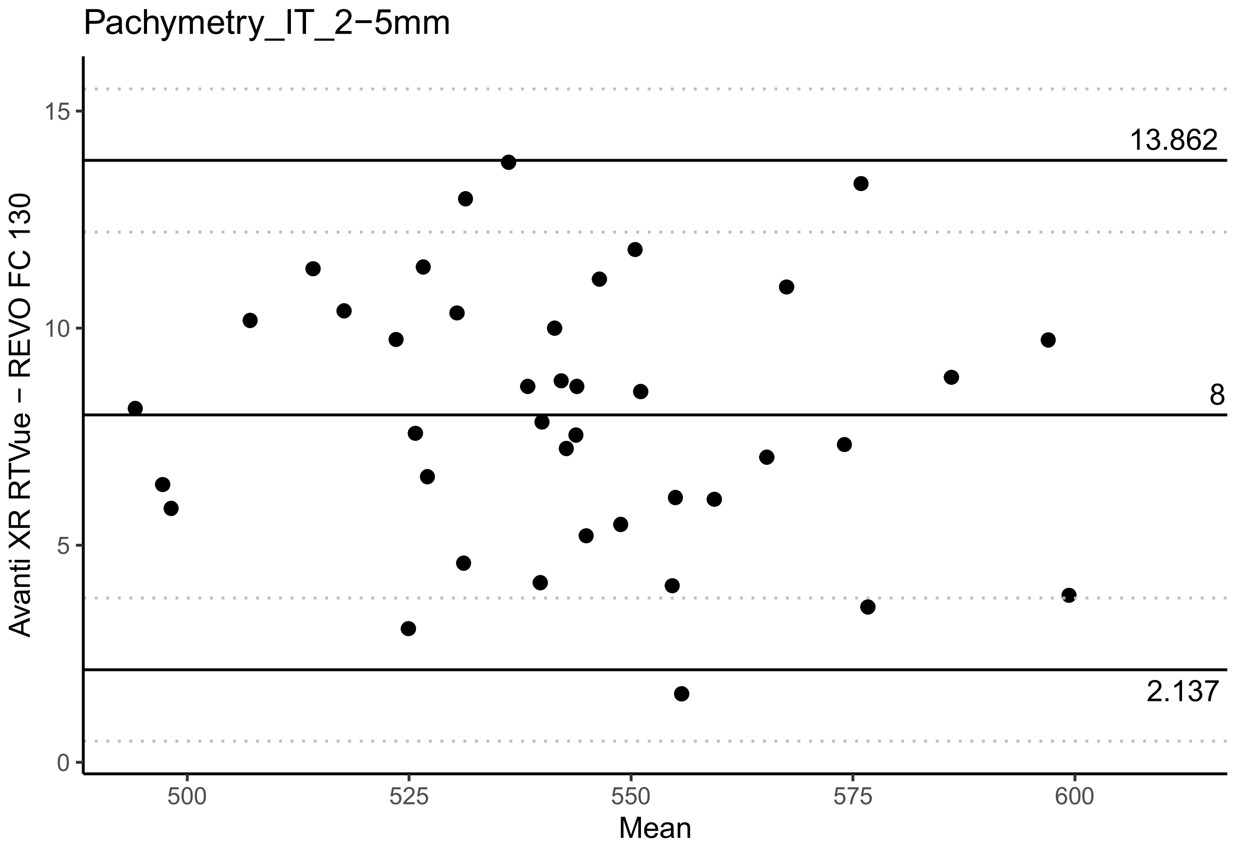

Table 33: Shapiro-Wilk Test Results

Test	W_statistic	p_value
Avanti RTVue	0.9745113	0.4937183
REVO FC 130	0.9823876	0.7770067
Differences	0.9874442	0.9298009

Table 34: Paired T-test results

	Test	t_statistic	p_value
t	Paired t-test for mean differences	16.91516	0

Table 35: Bland Altman Statistics

Sector	Mean_Diff	SD	upperLOA	lower_CI	upper_CI	lowerLOA	lower_CI_	upper_CI_
Pachymetry_IT_2-5mm	7.9998	2.9911	13.8623	12.2137	15.5109	2.1372	0.4886	3.7858

Table 36: Basic Statistics

Mean_Avanti	SD1	Min1	Max1	Mean_REVO	SD2	Min2	Max2
547.1498	24.8931	498.23	601.87	539.15	25.2311	490.08	597.41

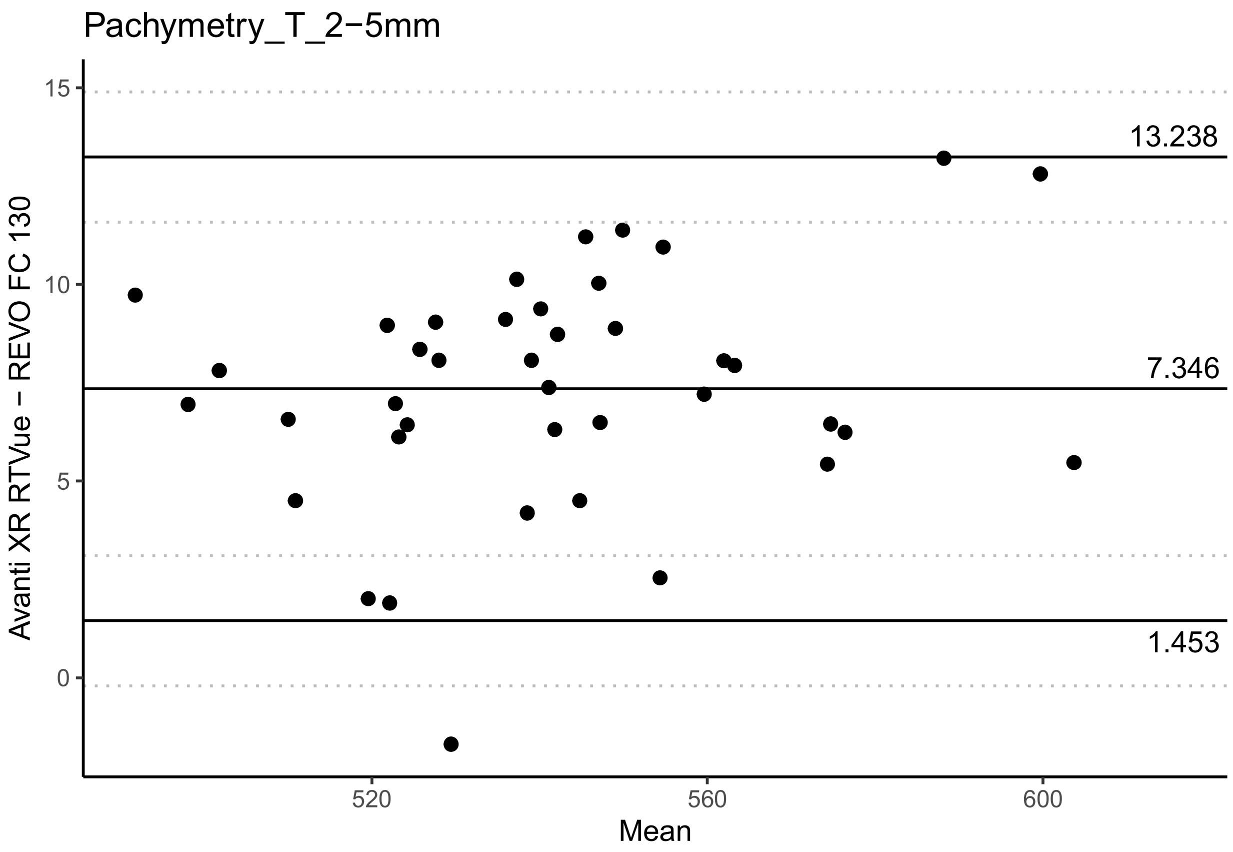

Table 37: Shapiro-Wilk Test Results

Test	W_statistic	p_value
Avanti RTVue	0.9705486	0.3745401
REVO FC 130	0.9747240	0.5007530
Differences	0.9683070	0.3176705

Table 38: Paired T-test results

	Test	t_statistic	p_value
t	Paired t-test for mean differences	15.45221	0

Table 39: Bland Altman Statistics

Sector	Mean_Diff	SD	upperLOA	lower_CI	upper_CI	lowerLOA	lower_CI_	upper_CI_
Pachymetry_T_2-5mm	7.3455	3.0065	13.2382	11.5812	14.8953	1.4528	-0.2043	3.1098

Table 40: Basic Statistics

Mean_Avanti	SD1	Min1	Max1	Mean_REVO	SD2	Min2	Max2
545.366	25.9621	496.68	606.43	538.0205	25.274	486.95	600.96

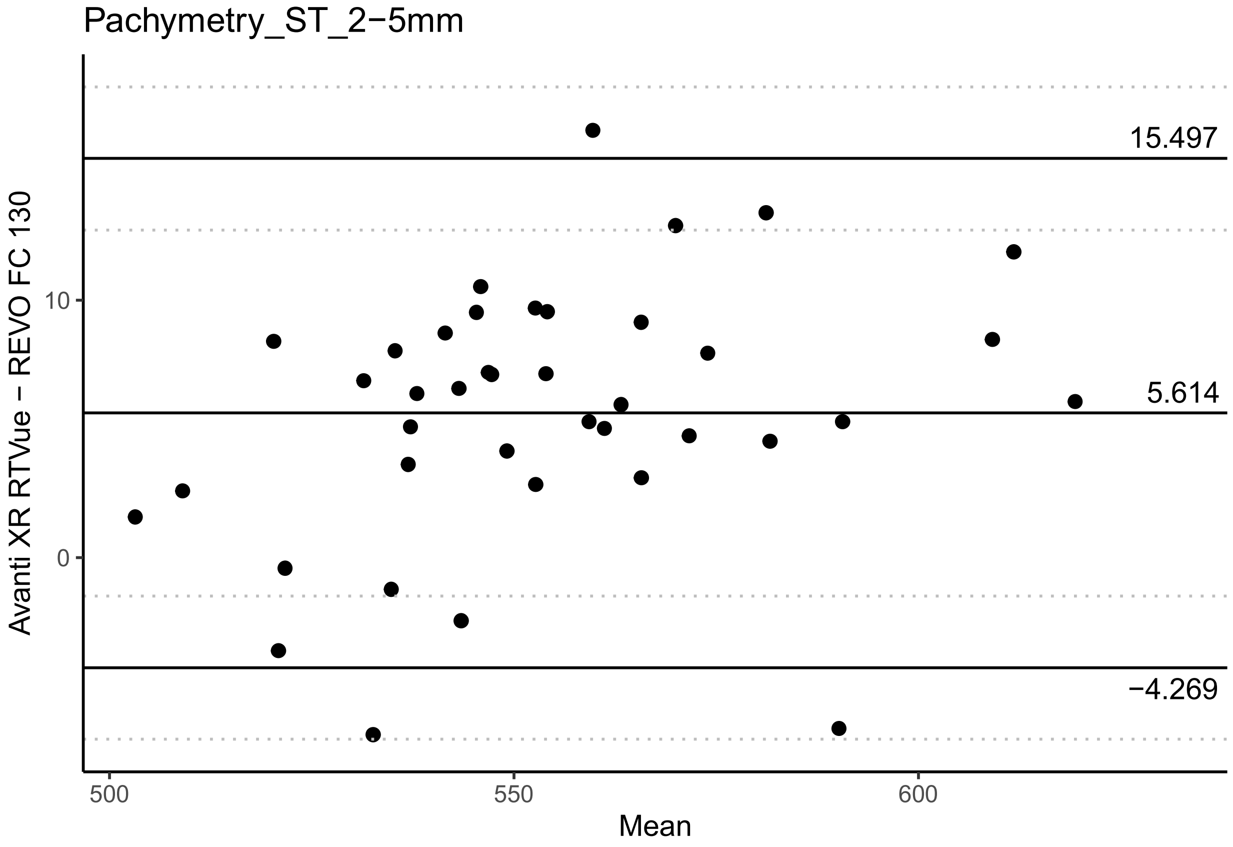

Table 41: Shapiro-Wilk Test Results

Test	W_statistic	p_value
Avanti RTVue	0.9744381	0.4913120
REVO FC 130	0.9603316	0.1719262
Differences	0.9592387	0.1577434

Table 42: Paired T-test results

	Test	t_statistic	p_value
t	Paired t-test for mean differences	7.041425	0

Table 43: Bland Altman Statistics

Sector	Mean_Diff	SD	upperLOA	lower_CI	upper_CI	lowerLOA	lower_CI_	upper_CI_
Pachymetry_ST_2-5mm	5.6138	5.0422	15.4965	12.7175	18.2756	-4.269	-7.0481	-1.49

Table 44: Basic Statistics

Mean_Avanti	SD1	Min1	Max1	Mean_REVO	SD2	Min2	Max2
557.1095	27.304	503.98	622.41	551.4958	25.8151	502.4	616.35

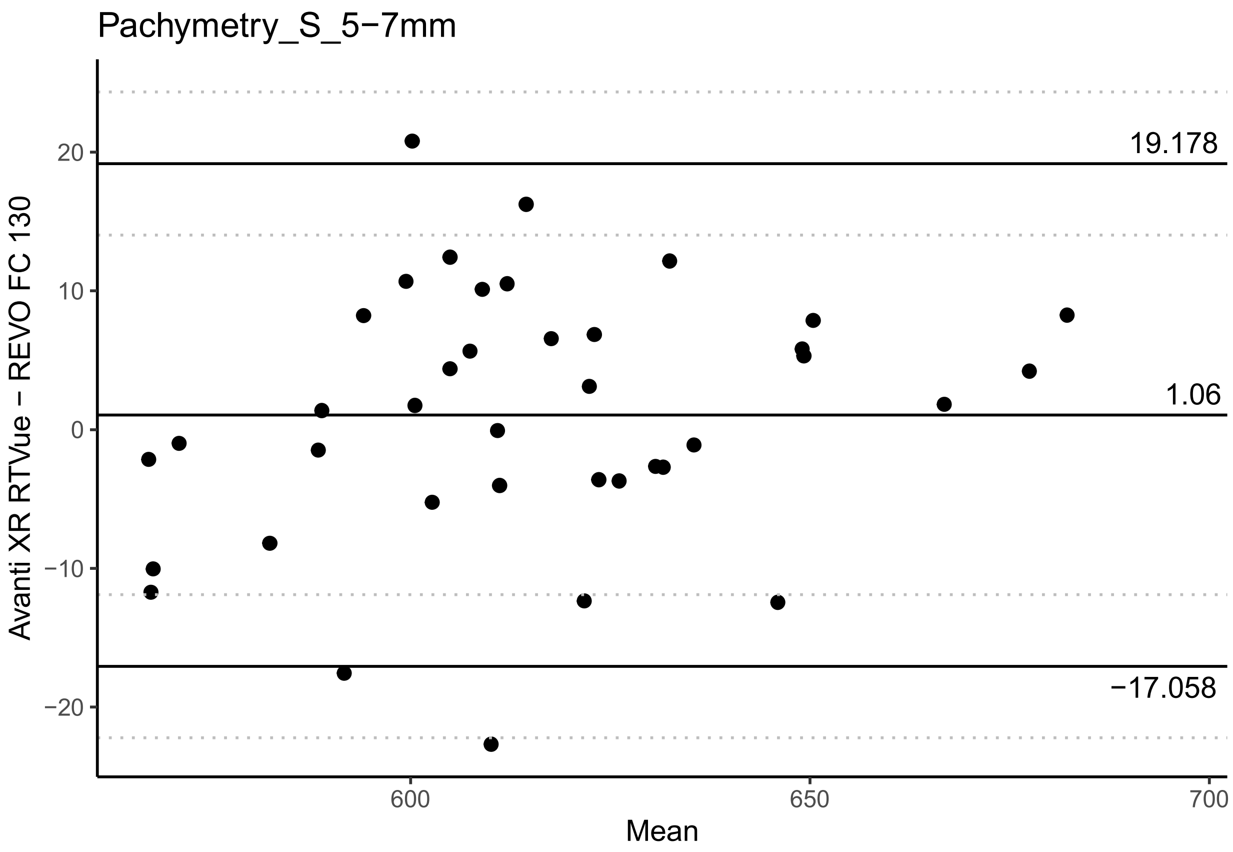

Table 45: Shapiro-Wilk Test Results

Test	W_statistic	p_value
Avanti RTVue	0.9741658	0.4824190
REVO FC 130	0.9690920	0.3520116
Differences	0.9841122	0.8457524

Table 46: Paired T-test results

	Test	t_statistic	p_value
t	Paired t-test for mean differences	0.7162958	0.4781879

Table 47: Bland Altman Statistics

Sector	Mean_Diff	SD	upperLOA	lower_CI	upper_CI	lowerLOA	lower_CI_	upper_CI_
Pachymetry_S_5-7mm	1.0603	9.2438	19.1781	14.0129	24.3433	-17.0576	-22.2228	-11.8924

Table 48: Basic Statistics

Mean_Avanti	SD1	Min1	Max1	Mean_REVO	SD2	Min2	Max2
615.7295	29.7711	561.63	686.32	614.6692	27.6781	568.28	678.07

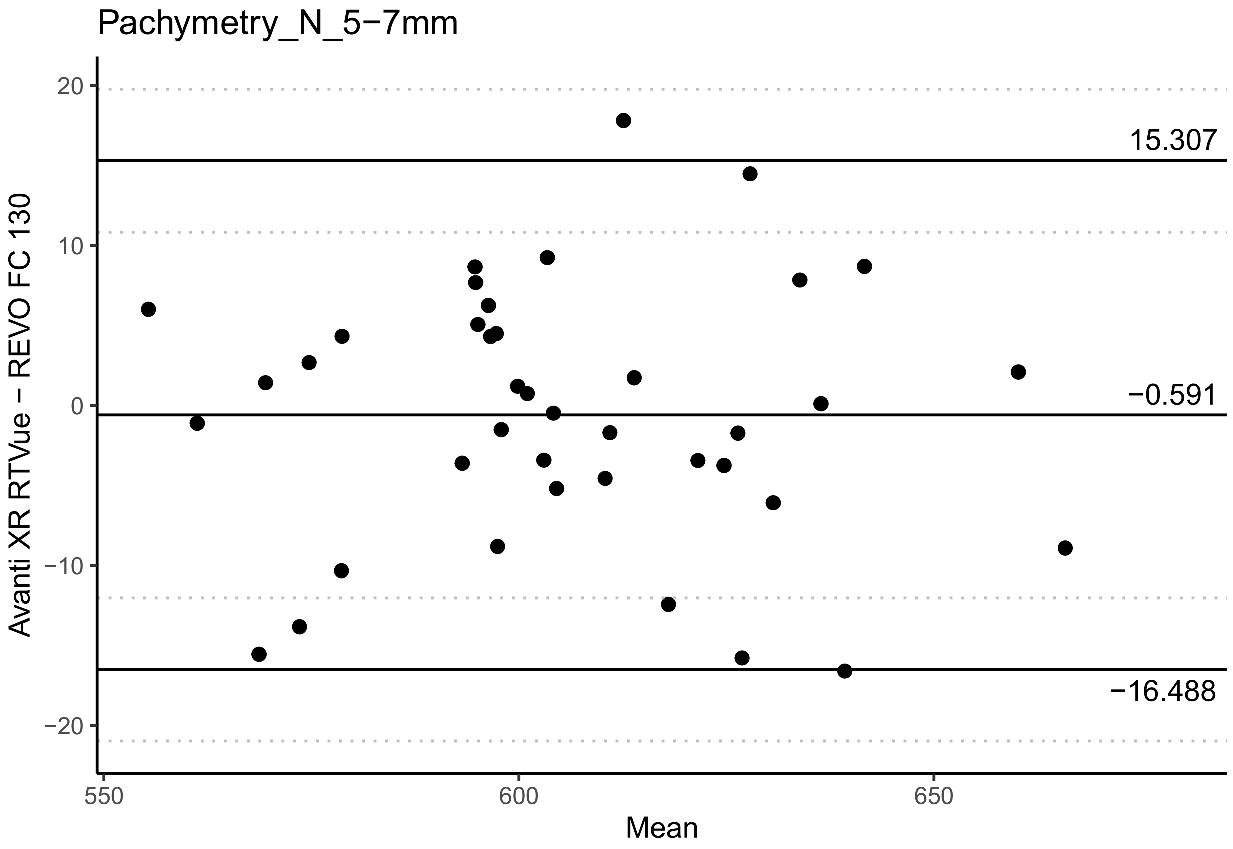

Table 49: Shapiro-Wilk Test Results

Test	W_statistic	p_value
Avanti RTVue	0.9708569	0.3829633
REVO FC 130	0.9864077	0.9044585
Differences	0.9799894	0.6891596

Table 50: Paired T-test results

	Test	t_statistic	p_value
t	Paired t-test for mean differences	-0.4606442	0.6476123

Table 51: Bland Altman Statistics

Sector	Mean_Diff	SD	upperLOA	lower_CI	upper_CI	lowerLOA	lower_CI_	upper_CI_
Pachymetry_N_5-7mm	-0.5907	8.1109	15.3066	10.8362	19.777	-16.4881	-20.9585	-12.0177

Table 52: Basic Statistics

Mean_Avanti	SD1	Min1	Max1	Mean_REVO	SD2	Min2	Max2
605.705	25.6303	558.36	661.35	606.2958	25.8965	552.34	670.25

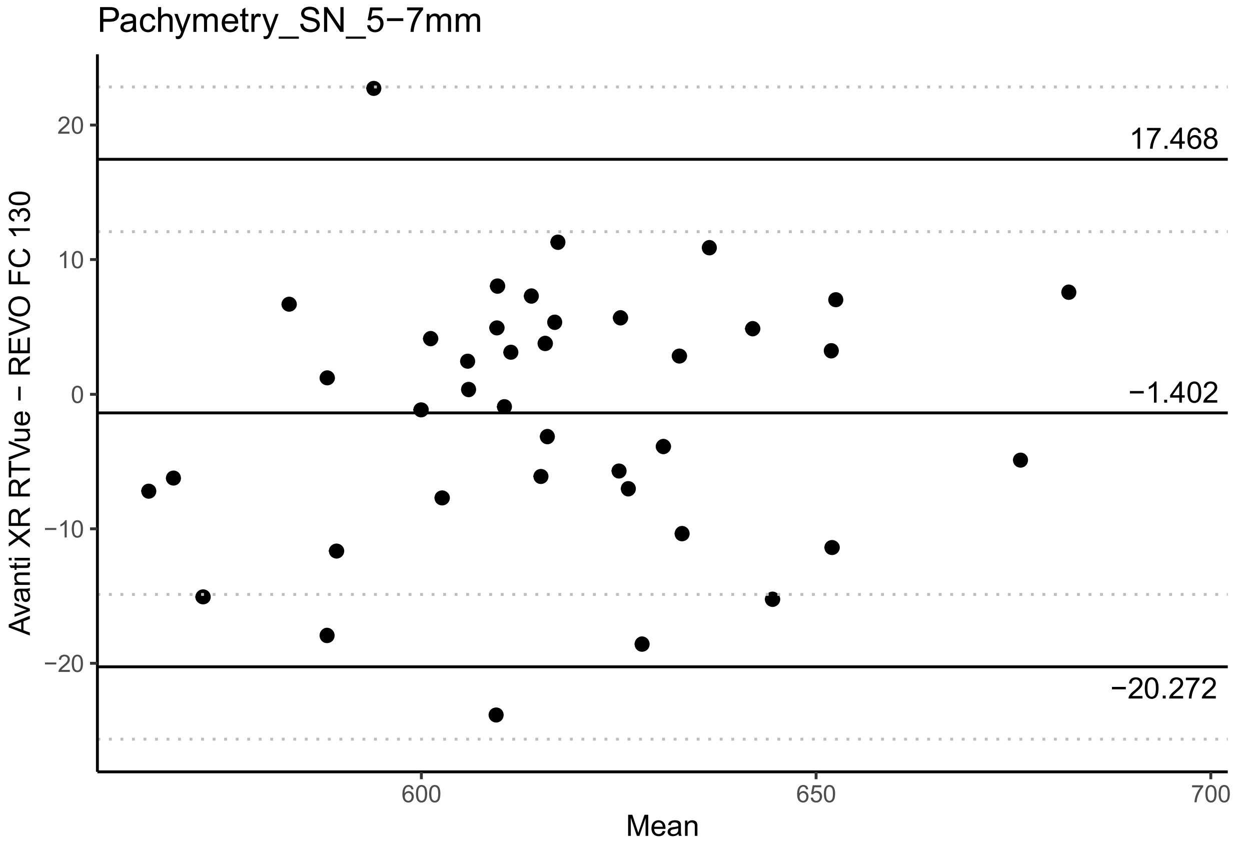

Table 53: Shapiro-Wilk Test Results

Test	W_statistic	p_value
Avanti RTVue	0.9765536	0.5635656
REVO FC 130	0.9761162	0.5642807
Differences	0.9740154	0.4938906

Table 54: Paired T-test results

	Test	t_statistic	p_value
t	Paired t-test for mean differences	-0.9092912	0.3689279

Table 55: Bland Altman Statistics

Sector	Mean_Diff	SD	upperLOA	lower_CI	upper_CI	lowerLOA	lower_CI_	upper_CI_
Pachymetry_SN_5-7mm	-1.4018	9.6275	17.4681	12.0885	22.8477	-20.2717	-25.6513	-14.8921

Table 56: Basic Statistics

Mean_Avanti	SD1	Min1	Max1	Mean_REVO	SD2	Min2	Max2
615.9049	27.4037	561.84	685.79	617.3067	26.3062	569.05	678.33

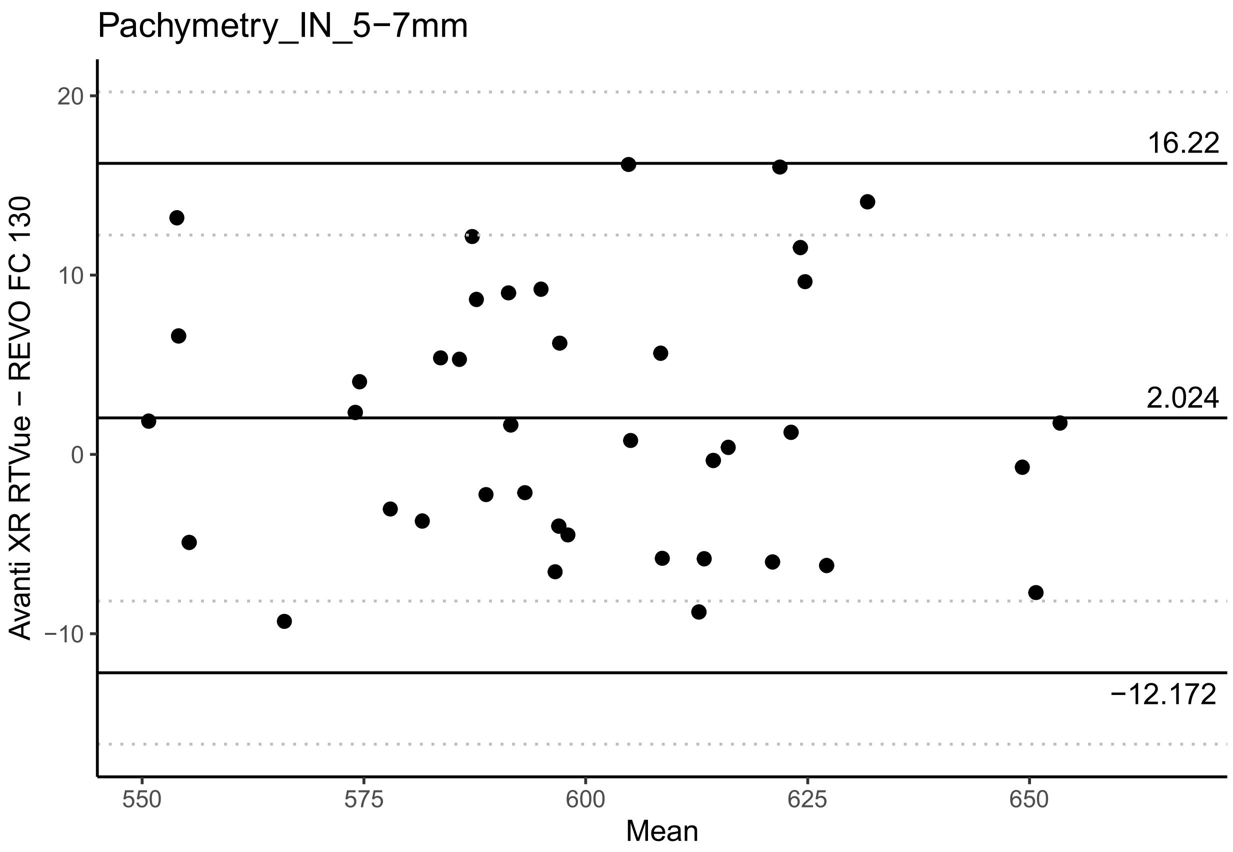

Table 57: Shapiro-Wilk Test Results

Test	W_statistic	p_value
Avanti RTVue	0.9798127	0.6825958
REVO FC 130	0.9769021	0.5759424
Differences	0.9564486	0.1264967

Table 58: Paired T-test results

	Test	t_statistic	p_value
t	Paired t-test for mean differences	1.76736	0.0849924

Table 59: Bland Altman Statistics

Sector	Mean_Diff	SD	upperLOA	lower_CI	upper_CI	lowerLOA	lower_CI_	upper_CI_
Pachymetry_IN_5-7mm	2.024	7.243	16.2202	12.2282	20.2122	-12.1722	-16.1642	-8.1802

Table 60: Basic Statistics

Mean_Avanti	SD1	Min1	Max1	Mean_REVO	SD2	Min2	Max2
600.7958	26.0721	551.66	654.32	598.7718	26.4329	547.33	654.59

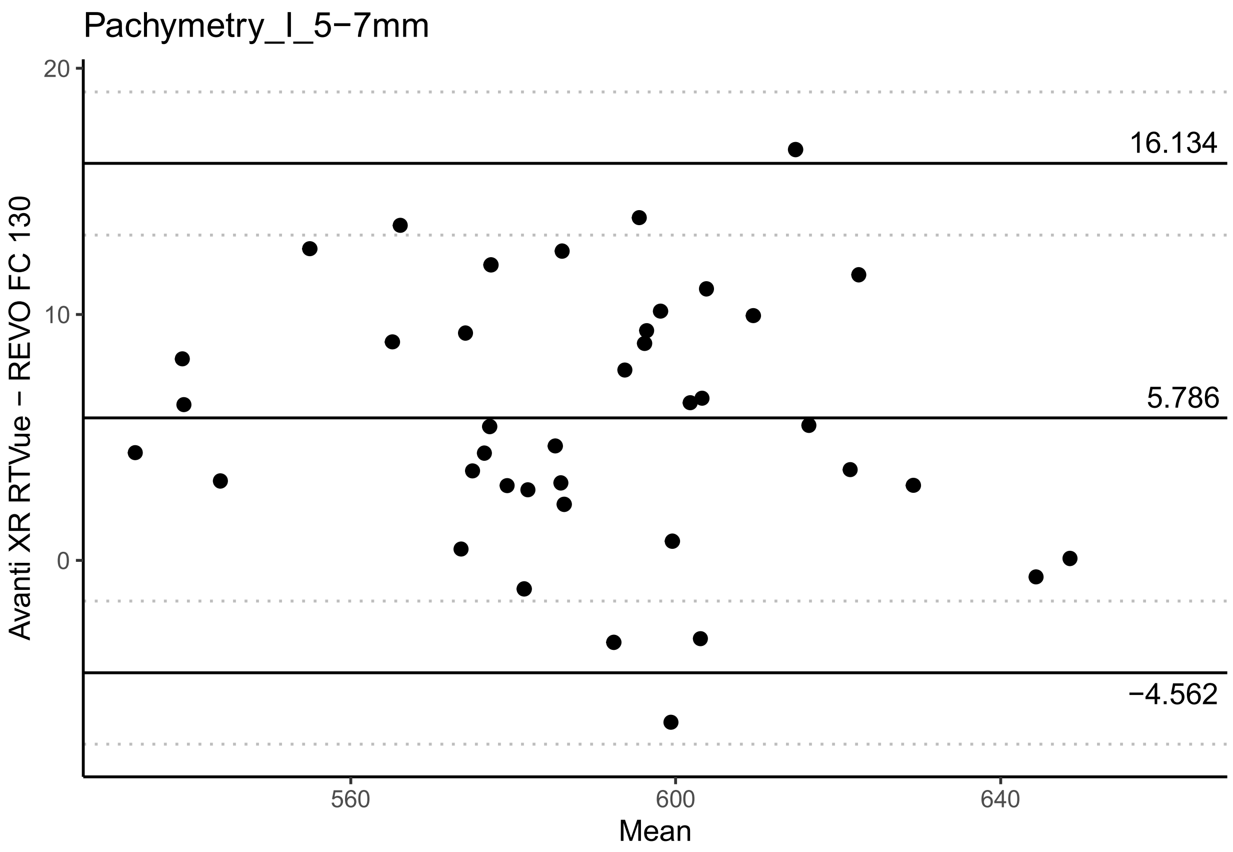

Table 61: Shapiro-Wilk Test Results

Test	W_statistic	p_value
Avanti RTVue	0.9783478	0.6283731
REVO FC 130	0.9760090	0.5444661
Differences	0.9890069	0.9602863

Table 62: Paired T-test results

	Test	t_statistic	p_value
t	Paired t-test for mean differences	6.931185	0

Table 63: Bland Altman Statistics

Sector	Mean_Diff	SD	upperLOA	lower_CI	upper_CI	lowerLOA	lower_CI_	upper_CI_
Pachymetry_I_5-7mm	5.786	5.2796	16.134	13.2241	19.0439	-4.562	-7.4719	-1.6521

Table 64: Basic Statistics

Mean_Avanti	SD1	Min1	Max1	Mean_REVO	SD2	Min2	Max2
592.1912	26.1426	535.69	648.56	586.4052	26.8594	531.31	648.48

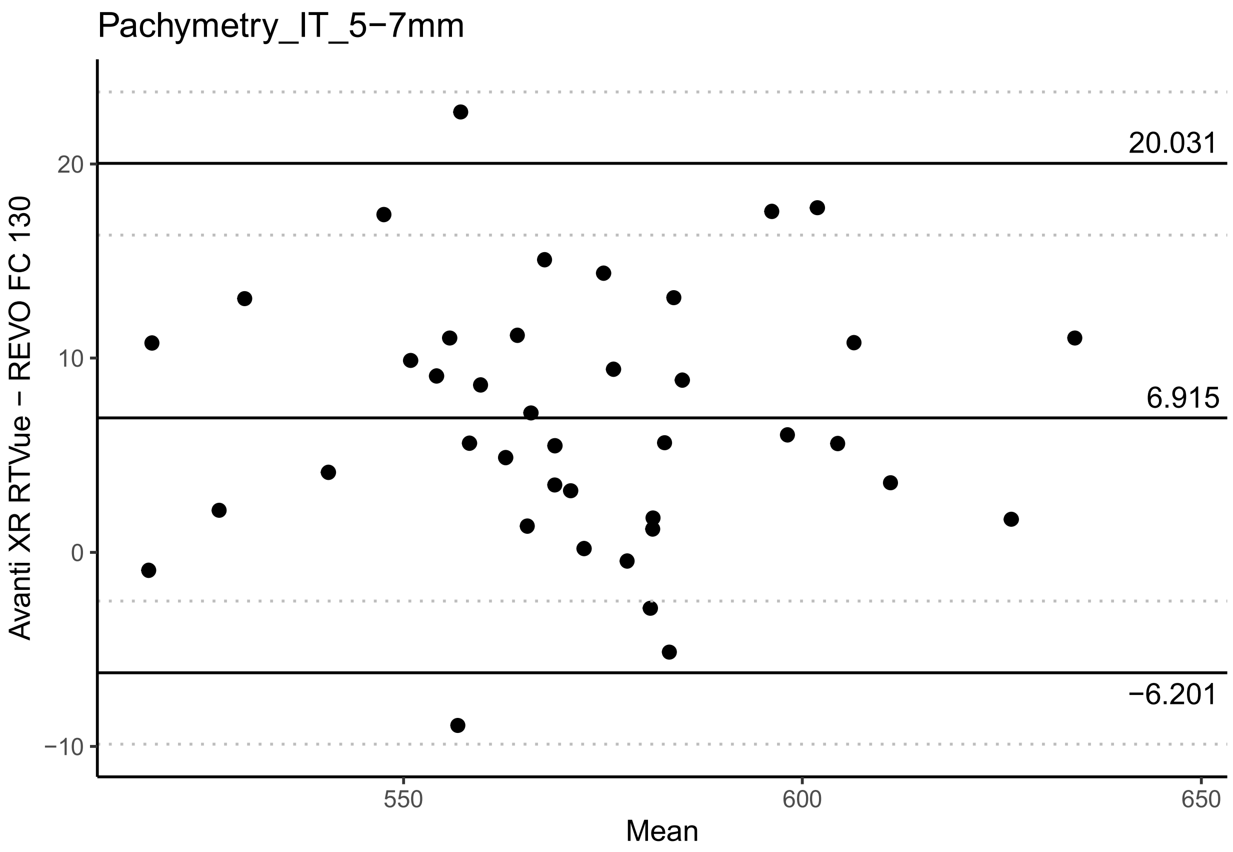

Table 65: Shapiro-Wilk Test Results

Test	W_statistic	p_value
Avanti RTVue	0.9776209	0.6018178
REVO FC 130	0.9851714	0.8695323
Differences	0.9924395	0.9944853

Table 66: Paired T-test results

	Test	t_statistic	p_value
t	Paired t-test for mean differences	6.535617	1e-07

Table 67: Bland Altman Statistics

Sector	Mean_Diff	SD	upperLOA	lower_CI	upper_CI	lowerLOA	lower_CI_	upper_CI_
Pachymetry_IT_5-7mm	6.915	6.6917	20.0307	16.3425	23.7189	-6.2007	-9.8889	-2.5125

Table 68: Basic Statistics

Mean_Avanti	SD1	Min1	Max1	Mean_REVO	SD2	Min2	Max2
575.1668	26.4688	517.6	639.65	568.2518	26.3887	513.09	628.61

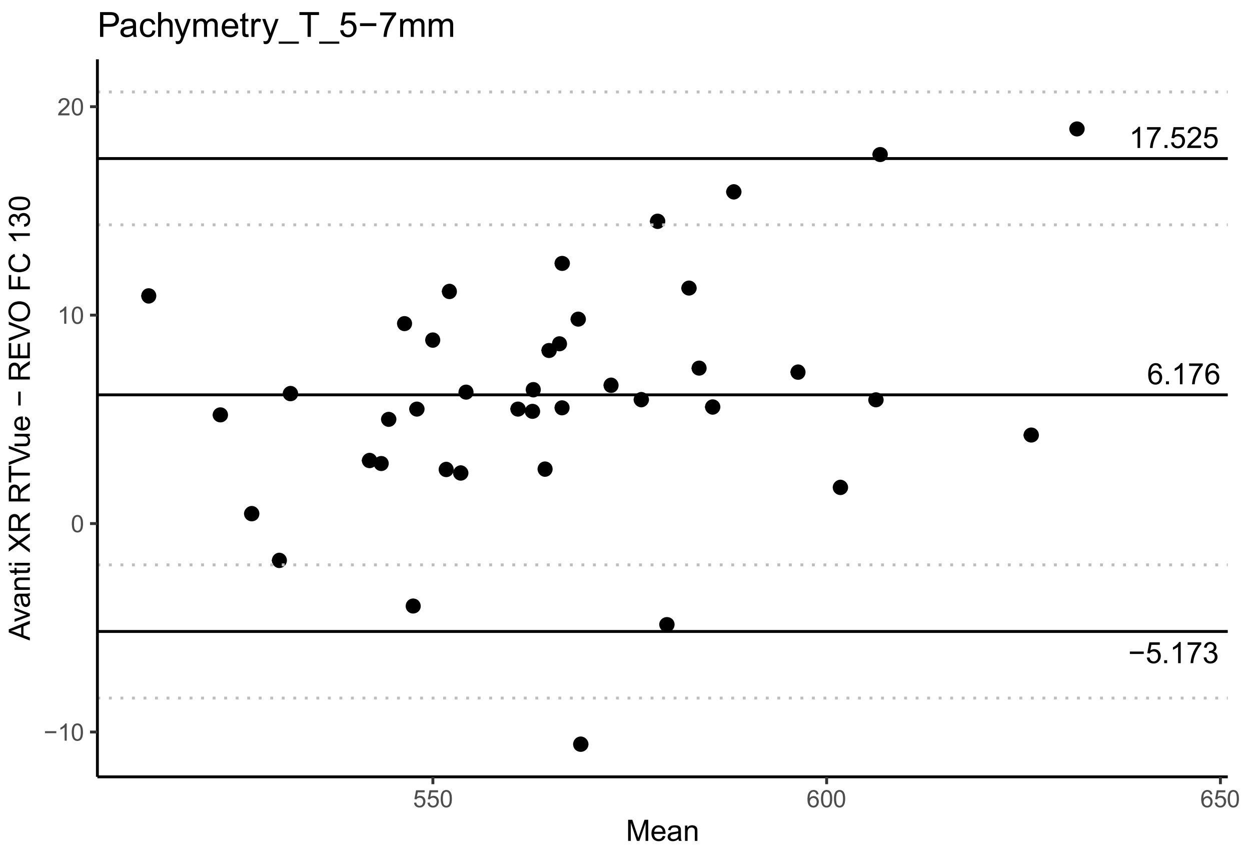

Table 69: Shapiro-Wilk Test Results

Test	W_statistic	p_value
Avanti RTVue	0.9750559	0.5118472
REVO FC 130	0.9788244	0.6459407
Differences	0.9632216	0.2155259

Table 70: Paired T-test results

	Test	t_statistic	p_value
t	Paired t-test for mean differences	6.745619	0

Table 71: Bland Altman Statistics

Sector	Mean_Diff	SD	upperLOA	lower_CI	upper_CI	lowerLOA	lower_CI_	upper_CI_
Pachymetry_T_5-7mm	6.1757	5.7903	17.5247	14.3333	20.716	-5.1732	-8.3645	-1.9818

Table 72: Basic Statistics

Mean_Avanti	SD1	Min1	Max1	Mean_REVO	SD2	Min2	Max2
568.764	27.6034	519.37	641.26	562.5882	25.7899	508.44	623.86

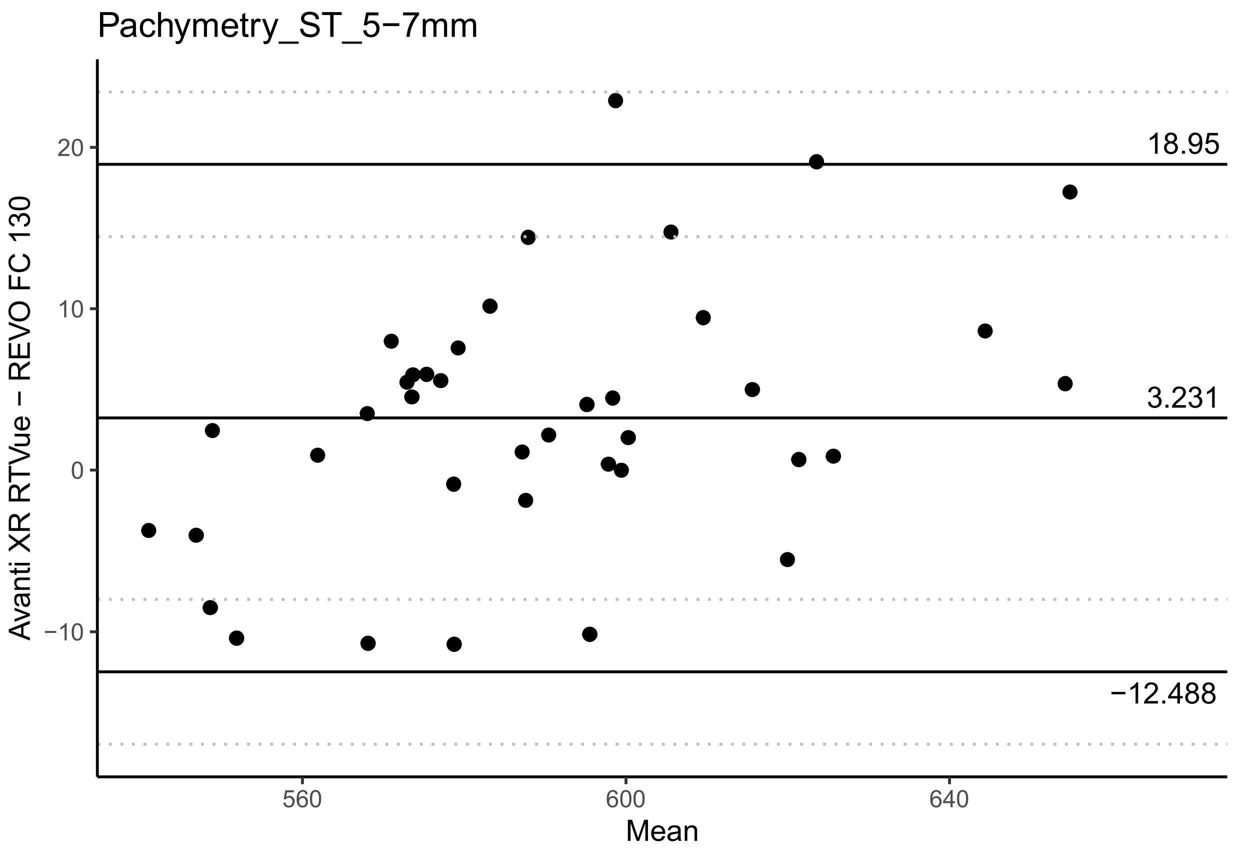

Table 73: Shapiro-Wilk Test Results

Test	W_statistic	p_value
Avanti RTVue	0.9683745	0.3192731
REVO FC 130	0.9671329	0.3053658
Differences	0.9665347	0.2922135

Table 74: Paired T-test results

	Test	t_statistic	p_value
t	Paired t-test for mean differences	2.516169	0.0162071

Table 75: Bland Altman Statistics

Sector	Mean_Diff	SD	upperLOA	lower_CI	upper_CI	lowerLOA	lower_CI_	upper_CI_
Pachymetry_ST_5-7mm	3.2313	8.0199	18.9502	14.469	23.4315	-12.4877	-16.9689	-8.0064

Table 76: Basic Statistics

Mean_Avanti	SD1	Min1	Max1	Mean_REVO	SD2	Min2	Max2
591.6828	30.1583	539.13	663.54	588.4515	26.7016	542.86	651.65

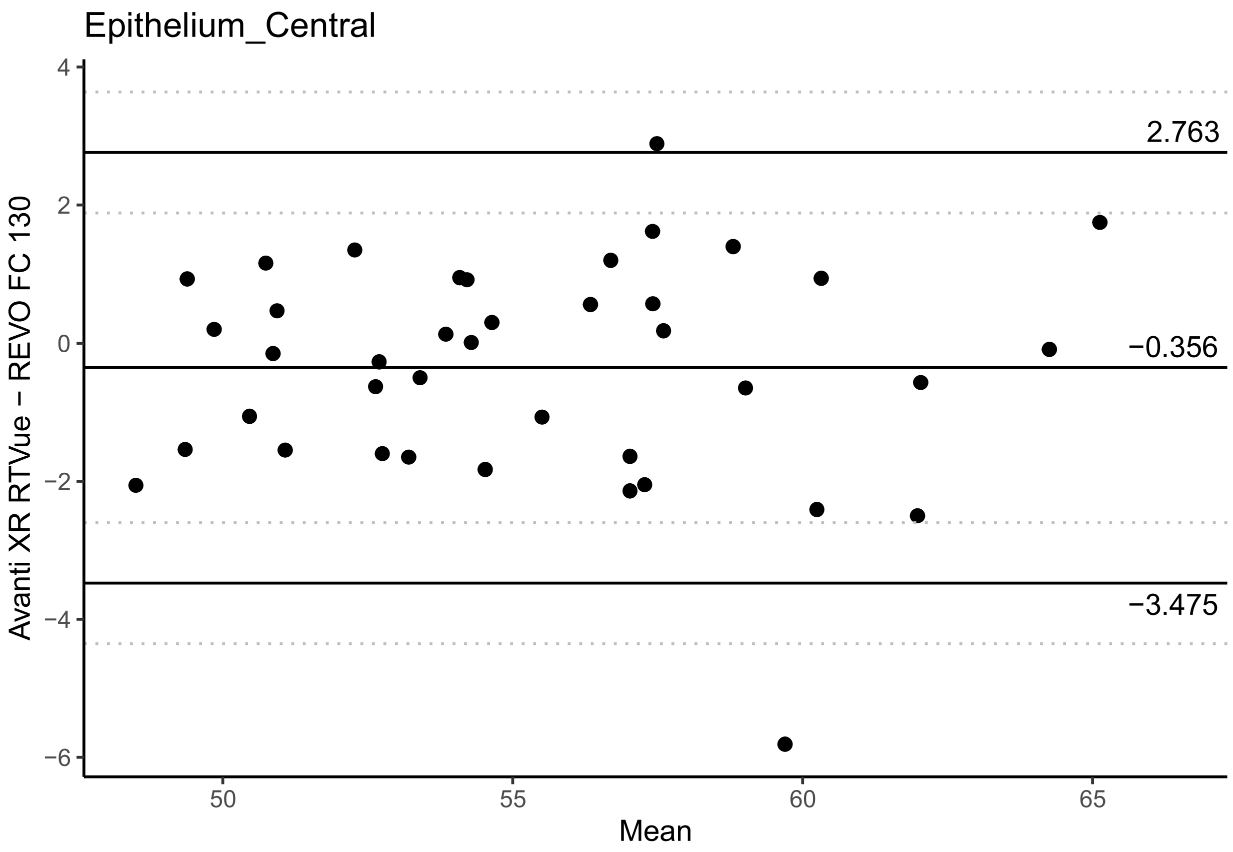

Table 77: Shapiro-Wilk Test Results

Test	W_statistic	p_value
Avanti RTVue	0.9803964	0.7042655
REVO FC 130	0.9541339	0.1052695
Differences	0.9455417	0.0533250

Table 78: Paired T-test results

	Test	t_statistic	p_value
t	Paired t-test for mean differences	-1.414778	0.1650686

Table 79: Bland Altman Statistics

Sector	Mean_Diff	SD	upperLOA	lower_CI	upper_CI	lowerLOA	lower_CI_	upper_CI_
Epithelium_Central	-0.356	1.5914	2.7632	1.8861	3.6404	-3.4752	-4.3524	-2.5981

Table 80: Basic Statistics

Mean_Avanti	SD1	Min1	Max1	Mean_REVO	SD2	Min2	Max2
55.1955	4.2556	47.47	66	55.5515	4.2754	48.92	64.3

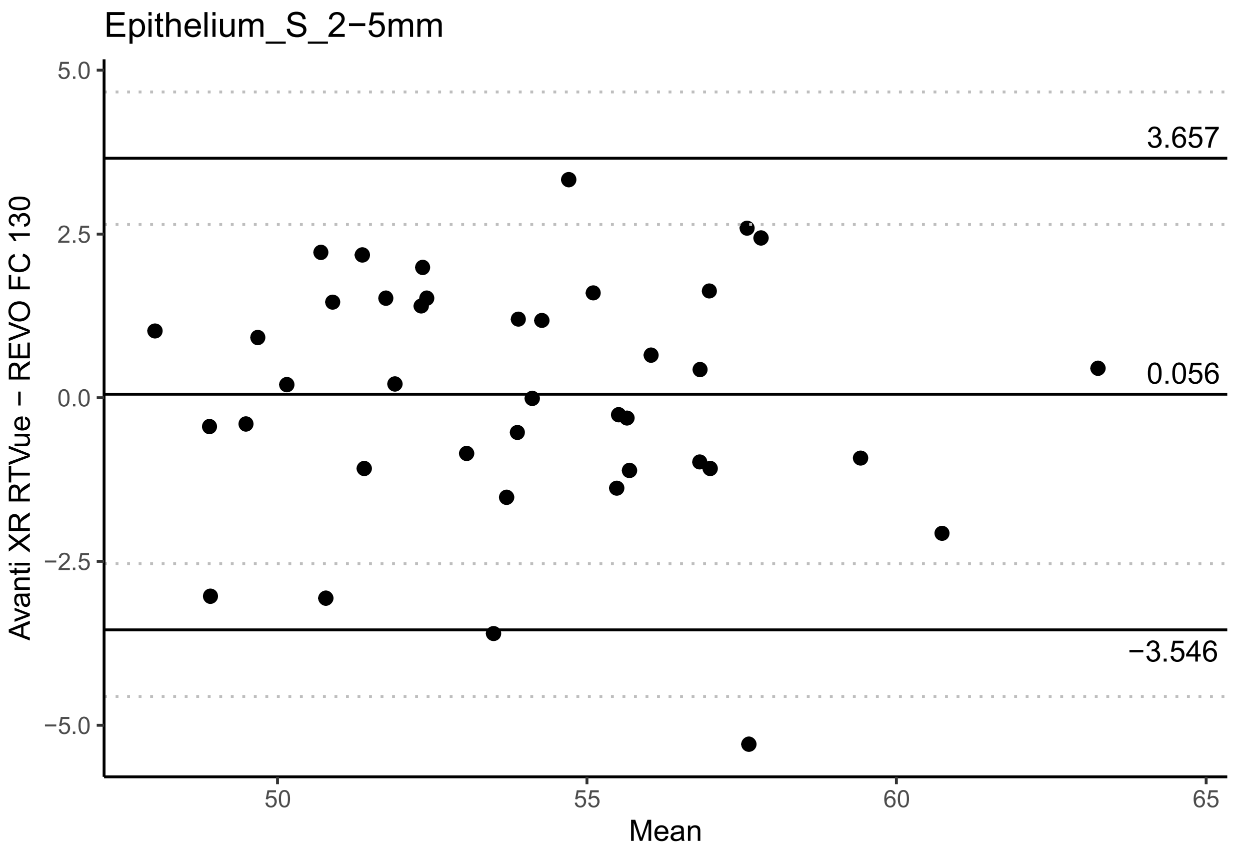

Table 81: Shapiro-Wilk Test Results

Test	W_statistic	p_value
Avanti RTVue	0.9845851	0.8514276
REVO FC 130	0.9658014	0.2629284
Differences	0.9618688	0.1939561

Table 82: Paired T-test results

	Test	t_statistic	p_value
t	Paired t-test for mean differences	0.1910189	0.8495018

Table 83: Bland Altman Statistics

Sector	Mean_Diff	SD	upperLOA	lower_CI	upper_CI	lowerLOA	lower_CI_	upper_CI_
Epithelium_S_2-5mm	0.0555	1.8376	3.6572	2.6444	4.67	-3.5462	-4.559	-2.5334

Table 84: Basic Statistics

Mean_Avanti	SD1	Min1	Max1	Mean_REVO	SD2	Min2	Max2
54.0178	3.4672	47.4	63.48	53.9622	3.6085	47.51	63.03

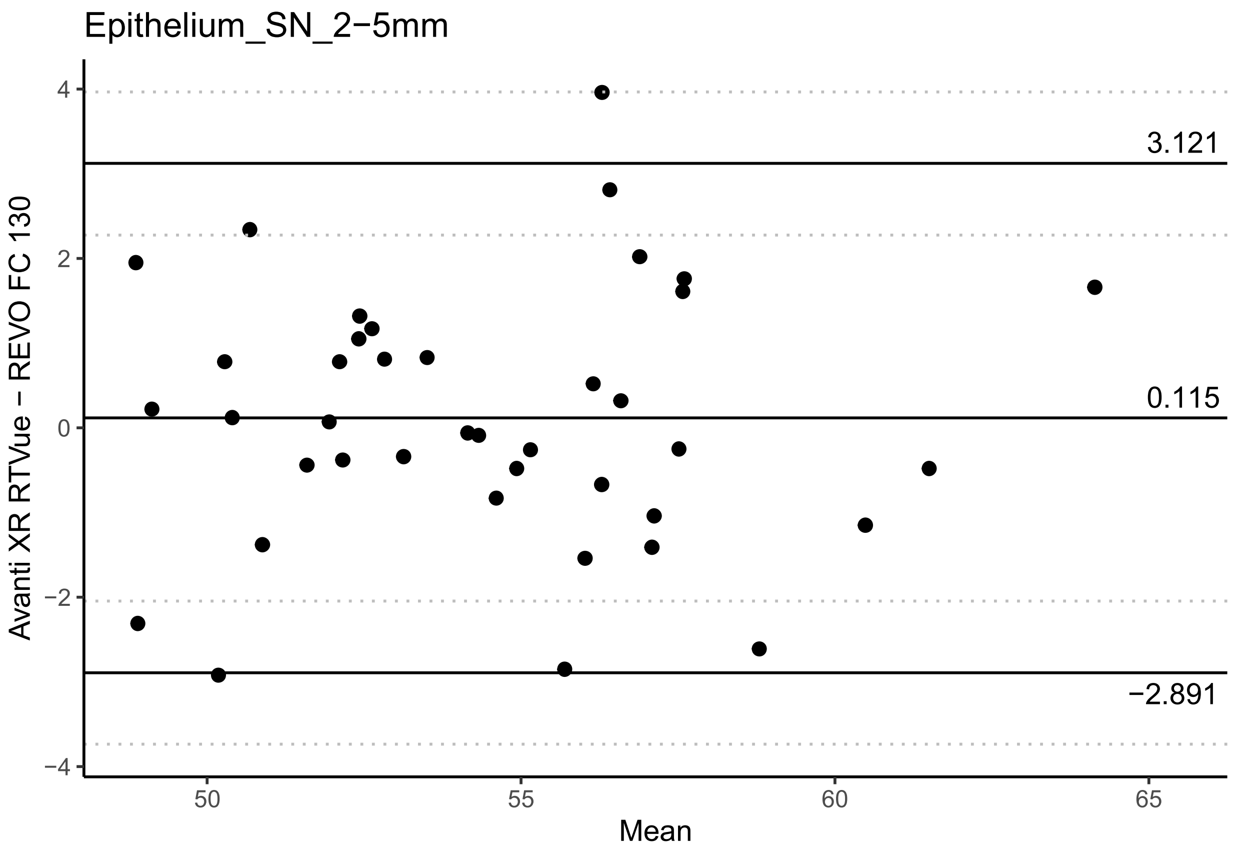

Table 85: Shapiro-Wilk Test Results

Test	W_statistic	p_value
Avanti RTVue	0.9804908	0.7077681
REVO FC 130	0.9698374	0.3556657
Differences	0.9871544	0.9231058

Table 86: Paired T-test results

	Test	t_statistic	p_value
t	Paired t-test for mean differences	0.4752892	0.6372321

Table 87: Bland Altman Statistics

Sector	Mean_Diff	SD	upperLOA	lower_CI	upper_CI	lowerLOA	lower_CI_	upper_CI_
Epithelium_SN_2-5mm	0.1152	1.5336	3.1211	2.2759	3.9664	-2.8906	-3.7359	-2.0454

Table 88: Basic Statistics

Mean_Avanti	SD1	Min1	Max1	Mean_REVO	SD2	Min2	Max2
54.5412	3.6219	47.74	64.97	54.426	3.5769	47.89	63.31

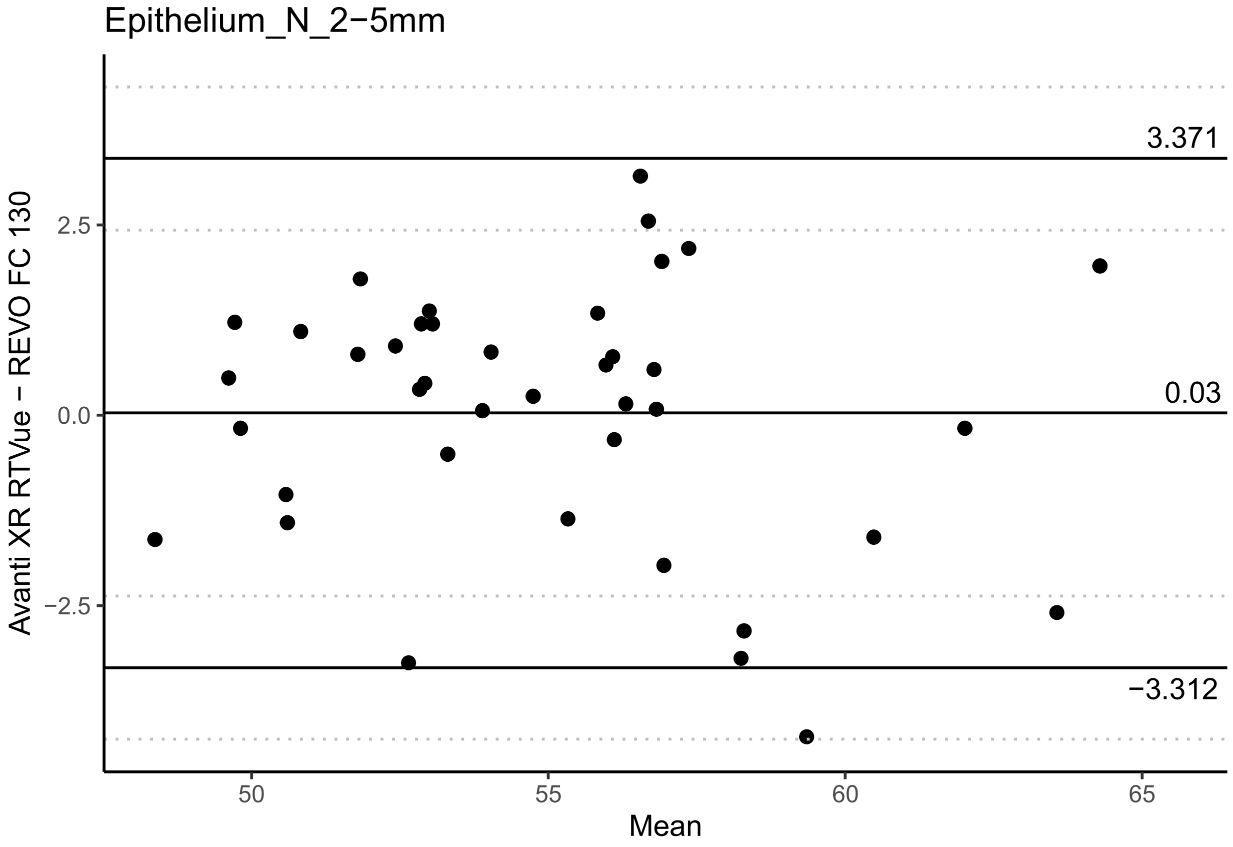

Table 89: Shapiro-Wilk Test Results

Test	W_statistic	p_value
Avanti RTVue	0.9733426	0.4561690
REVO FC 130	0.9478115	0.0637738
Differences	0.9564595	0.1266064

Table 90: Paired T-test results

	Test	t_statistic	p_value
t	Paired t-test for mean differences	0.1094276	0.9134245

Table 91: Bland Altman Statistics

Sector	Mean_Diff	SD	upperLOA	lower_CI	upper_CI	lowerLOA	lower_CI_	upper_CI_
Epithelium_N_2-5mm	0.0295	1.705	3.3713	2.4316	4.311	-3.3123	-4.252	-2.3726

Table 92: Basic Statistics

Mean_Avanti	SD1	Min1	Max1	Mean_REVO	SD2	Min2	Max2
54.9845	3.7321	47.56	65.27	54.955	3.9835	49.11	64.86

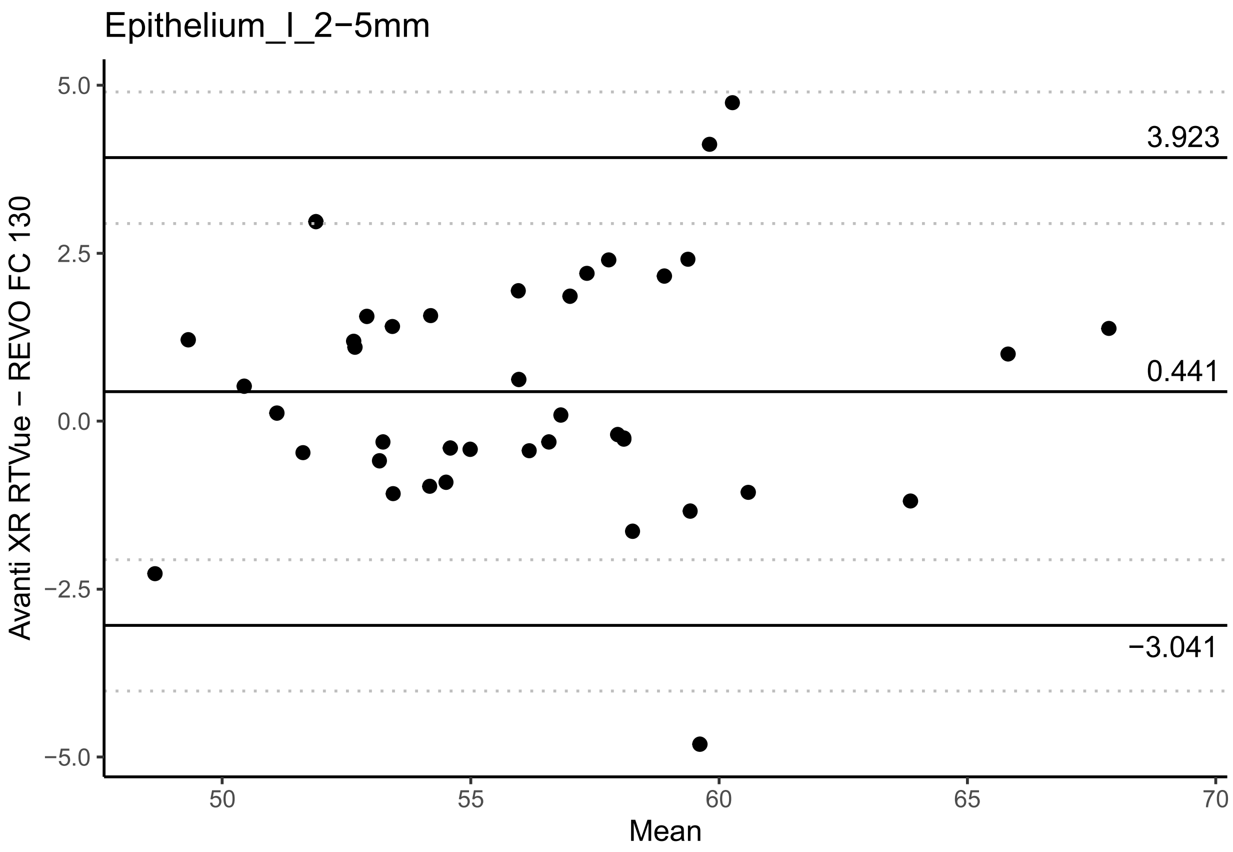

Table 93: Shapiro-Wilk Test Results

Test	W_statistic	p_value
Avanti RTVue	0.9704909	0.3729816
REVO FC 130	0.9617982	0.1928880
Differences	0.9677133	0.3038783

Table 94: Paired T-test results

	Test	t_statistic	p_value
t	Paired t-test for mean differences	1.570187	0.1244505

Table 95: Bland Altman Statistics

Sector	Mean_Diff	SD	upperLOA	lower_CI	upper_CI	lowerLOA	lower_CI_	upper_CI_
Epithelium_I_2-5mm	0.441	1.7763	3.9226	2.9435	4.9016	-3.0406	-4.0196	-2.0615

Table 96: Basic Statistics

Mean_Avanti	SD1	Min1	Max1	Mean_REVO	SD2	Min2	Max2
56.4322	4.3517	47.51	68.54	55.9912	4.1979	48.71	67.16

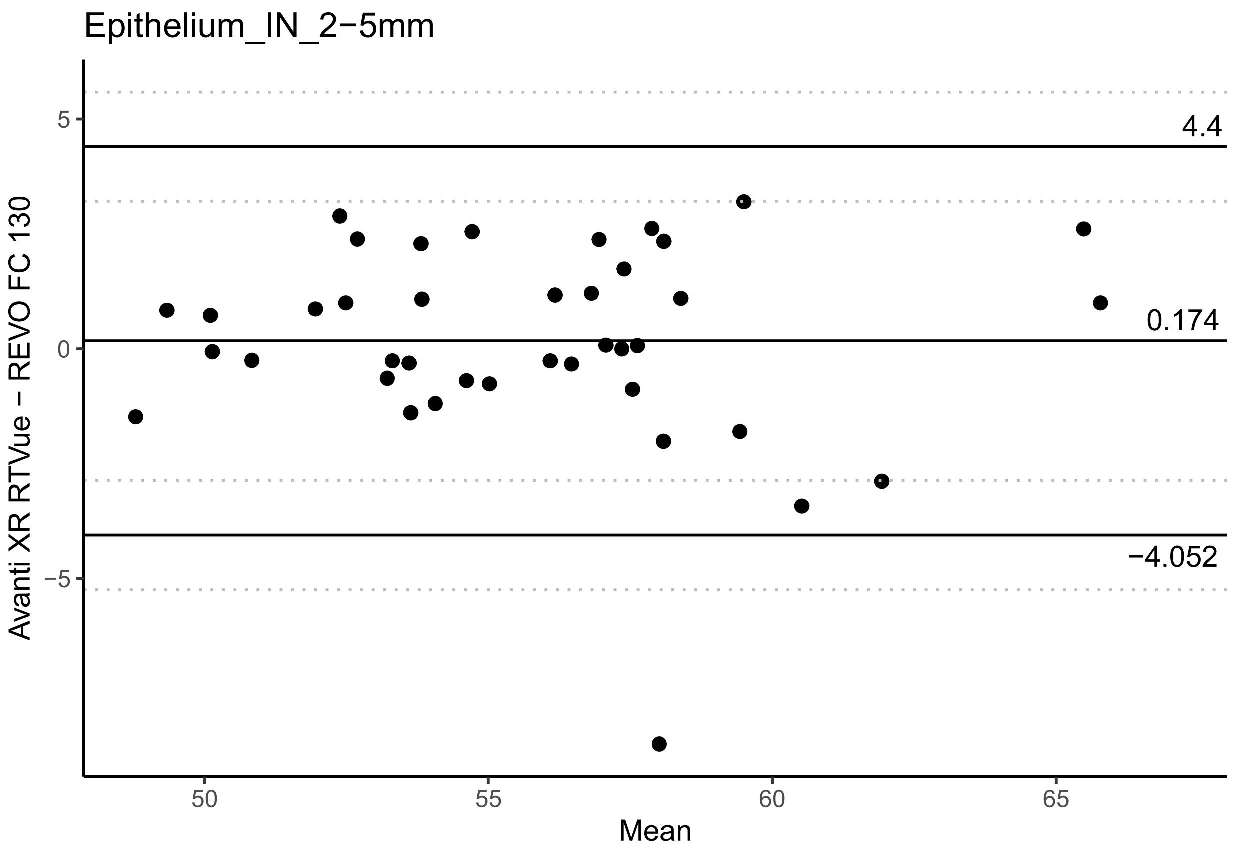

Table 97: Shapiro-Wilk Test Results

Test	W_statistic	p_value
Avanti RTVue	0.9567284	0.1293345
REVO FC 130	0.9627677	0.2080507
Differences	0.8707210	0.0002987

Table 98: Paired T-test results

	Test	t_statistic	p_value
t	Paired t-test for mean differences	0.5096452	0.6131721

Table 99: Bland Altman Statistics

Sector	Mean_Diff	SD	upperLOA	lower_CI	upper_CI	lowerLOA	lower_CI_	upper_CI_
Epithelium_IN_2-5mm	0.1737	2.1562	4.3999	3.2115	5.5883	-4.0524	-5.2408	-2.864

Table 100: Basic Statistics

Mean_Avanti	SD1	Min1	Max1	Mean_REVO	SD2	Min2	Max2
55.8653	3.9619	48.05	66.79	55.6915	4.083	48.92	65.28

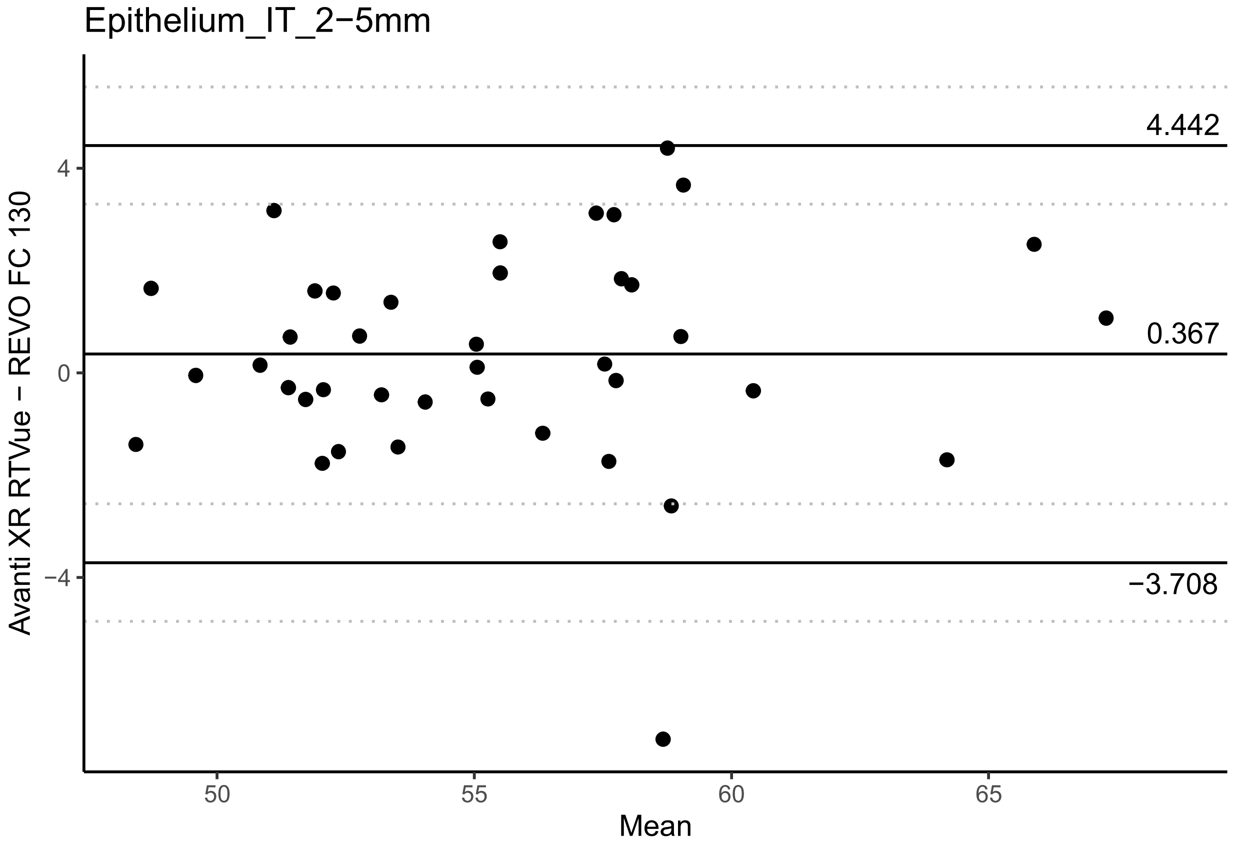

Table 101: Shapiro-Wilk Test Results

Test	W_statistic	p_value
Avanti RTVue	0.9517405	0.0870528
REVO FC 130	0.9541206	0.1051589
Differences	0.9353398	0.0241462

Table 102: Paired T-test results

	Test	t_statistic	p_value
t	Paired t-test for mean differences	1.1156	0.2714202

Table 103: Bland Altman Statistics

Sector	Mean_Diff	SD	upperLOA	lower_CI	upper_CI	lowerLOA	lower_CI_	upper_CI_
Epithelium_IT_2-5mm	0.3668	2.0792	4.4419	3.296	5.5879	-3.7084	-4.8544	-2.5625

Table 104: Basic Statistics

Mean_Avanti	SD1	Min1	Max1	Mean_REVO	SD2	Min2	Max2
55.6695	4.542	47.72	67.82	55.3028	4.4087	47.89	66.75

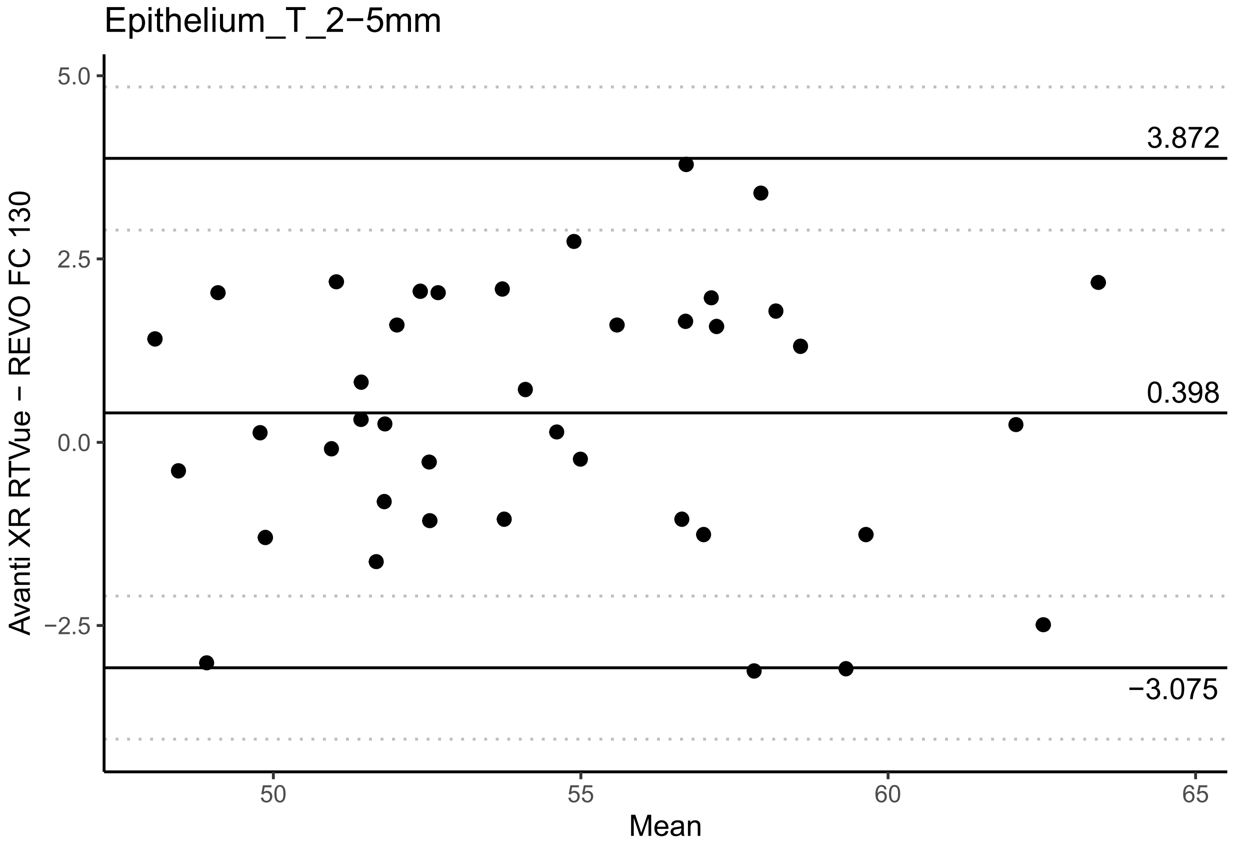

Table 105: Shapiro-Wilk Test Results

Test	W_statistic	p_value
Avanti RTVue	0.9799281	0.6868823
REVO FC 130	0.9635616	0.2212897
Differences	0.9631305	0.2140062

Table 106: Paired T-test results

	Test	t_statistic	p_value
t	Paired t-test for mean differences	1.421187	0.1632097

Table 107: Bland Altman Statistics

Sector	Mean_Diff	SD	upperLOA	lower_CI	upper_CI	lowerLOA	lower_CI_	upper_CI_
Epithelium_T_2-5mm	0.3982	1.7723	3.8719	2.8951	4.8487	-3.0754	-4.0522	-2.0986

Table 108: Basic Statistics

Mean_Avanti	SD1	Min1	Max1	Mean_REVO	SD2	Min2	Max2
54.6748	4.0619	47.41	64.51	54.2765	4.0516	47.37	63.77

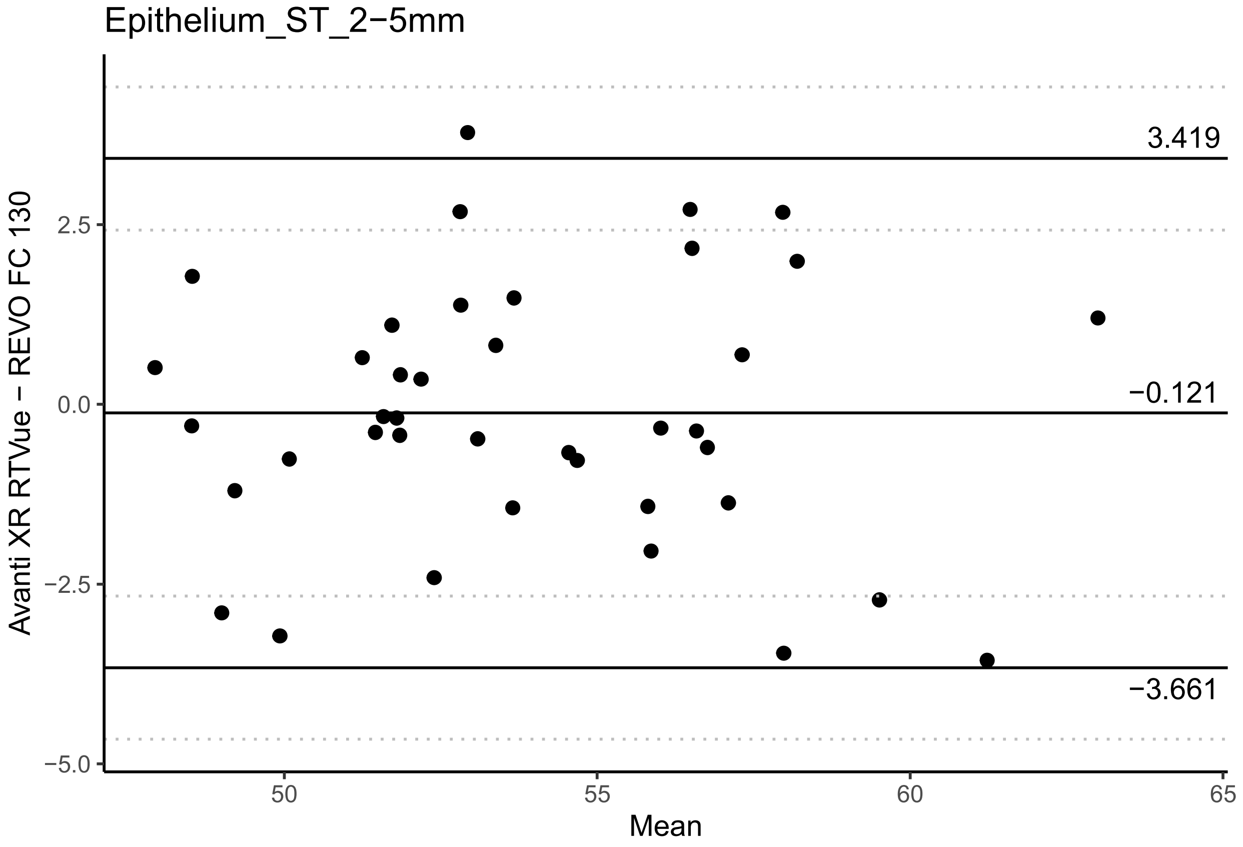

Table 109: Shapiro-Wilk Test Results

Test	W_statistic	p_value
Avanti RTVue	0.9769180	0.5765106
REVO FC 130	0.9563744	0.1257552
Differences	0.9807406	0.7170130

Table 110: Paired T-test results

	Test	t_statistic	p_value
t	Paired t-test for mean differences	-0.423705	0.6741088

Table 111: Bland Altman Statistics

Sector	Mean_Diff	SD	upperLOA	lower_CI	upper_CI	lowerLOA	lower_CI_	upper_CI_
Epithelium_ST_2-5mm	-0.121	1.8061	3.419	2.4236	4.4145	-3.661	-4.6565	-2.6656

Table 112: Basic Statistics

Mean_Avanti	SD1	Min1	Max1	Mean_REVO	SD2	Min2	Max2
53.869	3.6586	47.55	63.6	53.99	3.7168	47.64	63.01

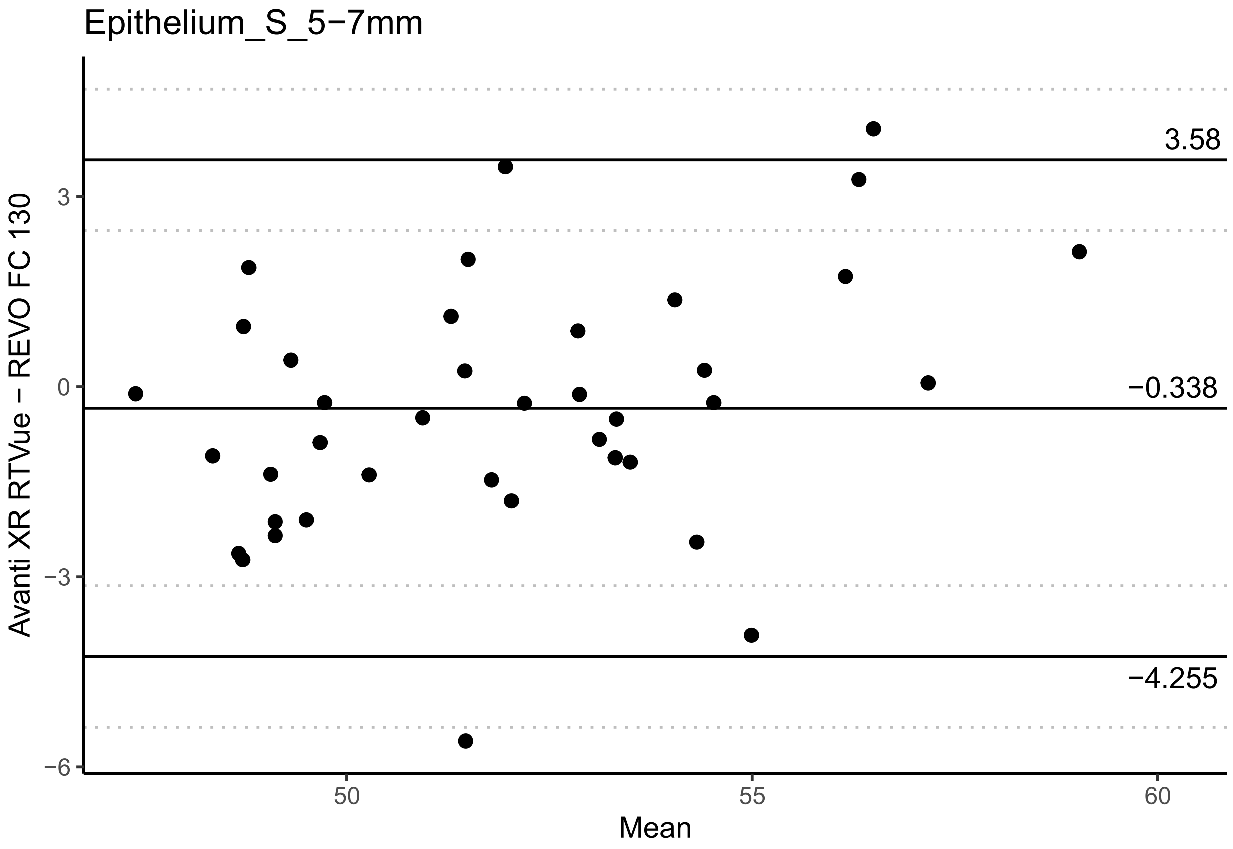

Table 113: Shapiro-Wilk Test Results

Test	W_statistic	p_value
Avanti RTVue	0.9476849	0.0631397
REVO FC 130	0.9693046	0.3574082
Differences	0.9863278	0.9088928

Table 114: Paired T-test results

	Test	t_statistic	p_value
t	Paired t-test for mean differences	-1.055065	0.2980578

Table 115: Bland Altman Statistics

Sector	Mean_Diff	SD	upperLOA	lower_CI	upper_CI	lowerLOA	lower_CI_	upper_CI_
Epithelium_S_5-7mm	-0.3377	1.9988	3.58	2.4631	4.6969	-4.2554	-5.3723	-3.1385

Table 116: Basic Statistics

Mean_Avanti	SD1	Min1	Max1	Mean_REVO	SD2	Min2	Max2
51.8162	3.3186	47.34	60.1	52.1538	2.6407	47.45	57.97

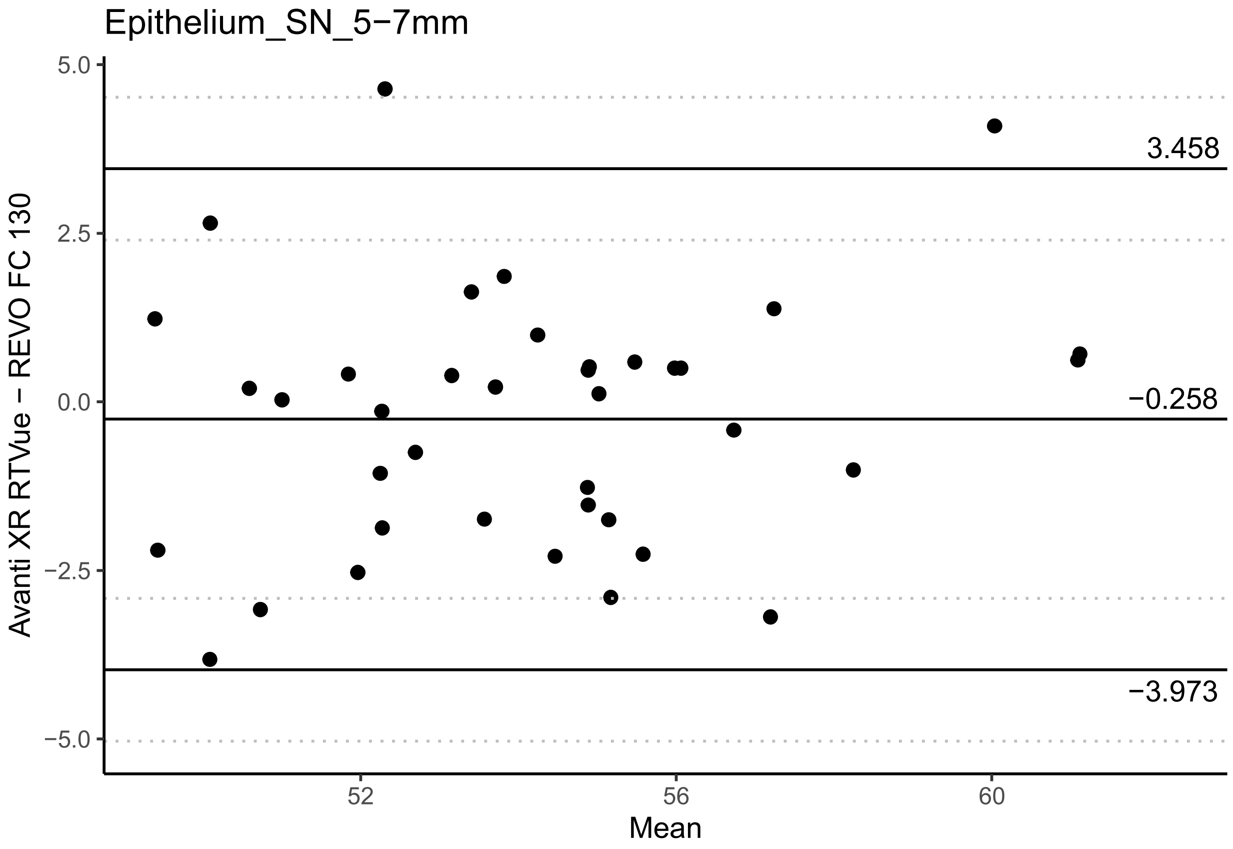

Table 117: Shapiro-Wilk Test Results

Test	W_statistic	p_value
Avanti RTVue	0.9598135	0.1650560
REVO FC 130	0.9812422	0.7483989
Differences	0.9661282	0.2835598

Table 118: Paired T-test results

	Test	t_statistic	p_value
t	Paired t-test for mean differences	-0.8497855	0.4007668

Table 119: Bland Altman Statistics

Sector	Mean_Diff	SD	upperLOA	lower_CI	upper_CI	lowerLOA	lower_CI_	upper_CI_
Epithelium_SN_5-7mm	-0.2579	1.8956	3.4575	2.3983	4.5167	-3.9734	-5.0326	-2.9142

Table 120: Basic Statistics

Mean_Avanti	SD1	Min1	Max1	Mean_REVO	SD2	Min2	Max2
54.049	3.2464	48.18	62.08	54.3069	2.936	48.77	60.78

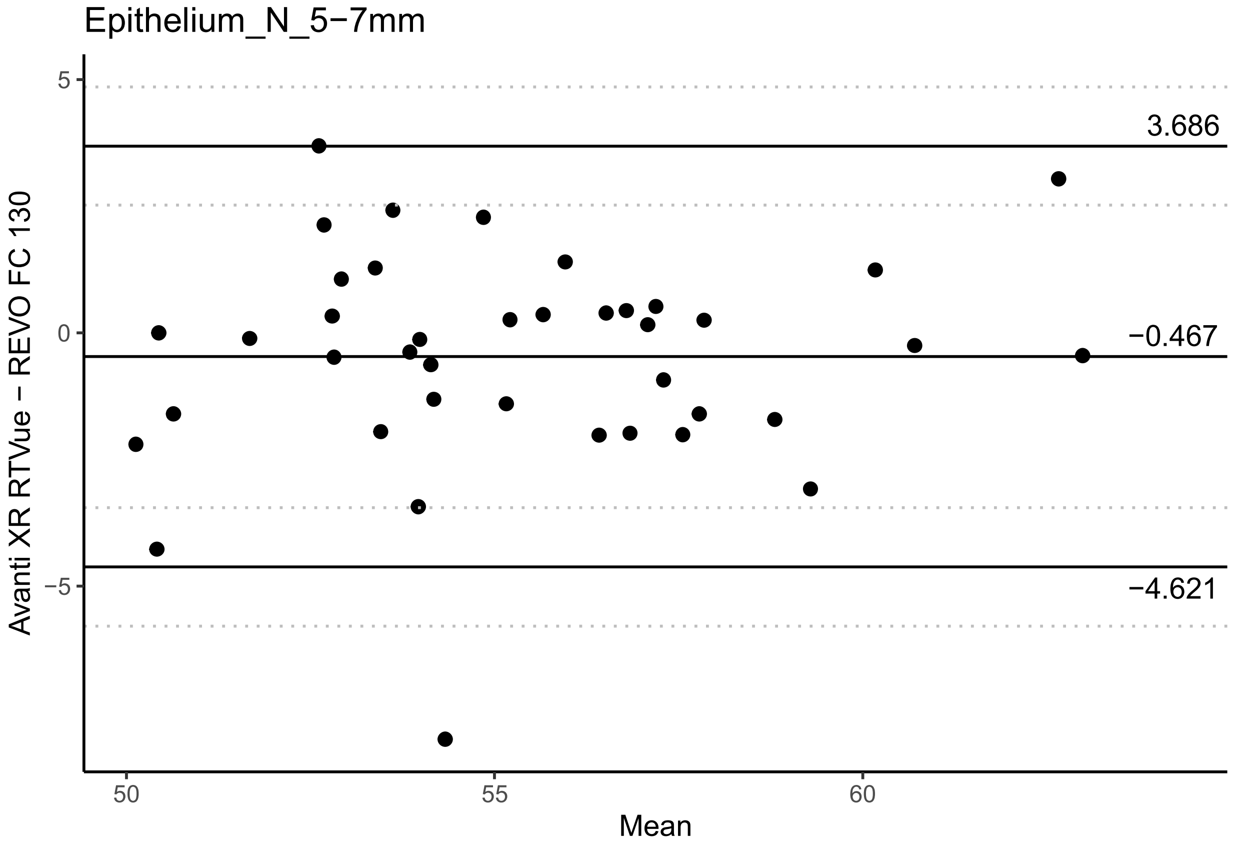

Table 121: Shapiro-Wilk Test Results

Test	W_statistic	p_value
Avanti RTVue	0.9743613	0.4887931
REVO FC 130	0.9706689	0.3778100
Differences	0.9386942	0.0312535

Table 122: Paired T-test results

	Test	t_statistic	p_value
t	Paired t-test for mean differences	-1.39456	0.171042

Table 123: Bland Altman Statistics

Sector	Mean_Diff	SD	upperLOA	lower_CI	upper_CI	lowerLOA	lower_CI_	upper_CI_
Epithelium_N_5-7mm	-0.4672	2.1191	3.6861	2.5182	4.854	-4.6206	-5.7885	-3.4527

Table 124: Basic Statistics

Mean_Avanti	SD1	Min1	Max1	Mean_REVO	SD2	Min2	Max2
55.1362	3.4485	48.28	64.18	55.6035	3.2257	50.44	63.21

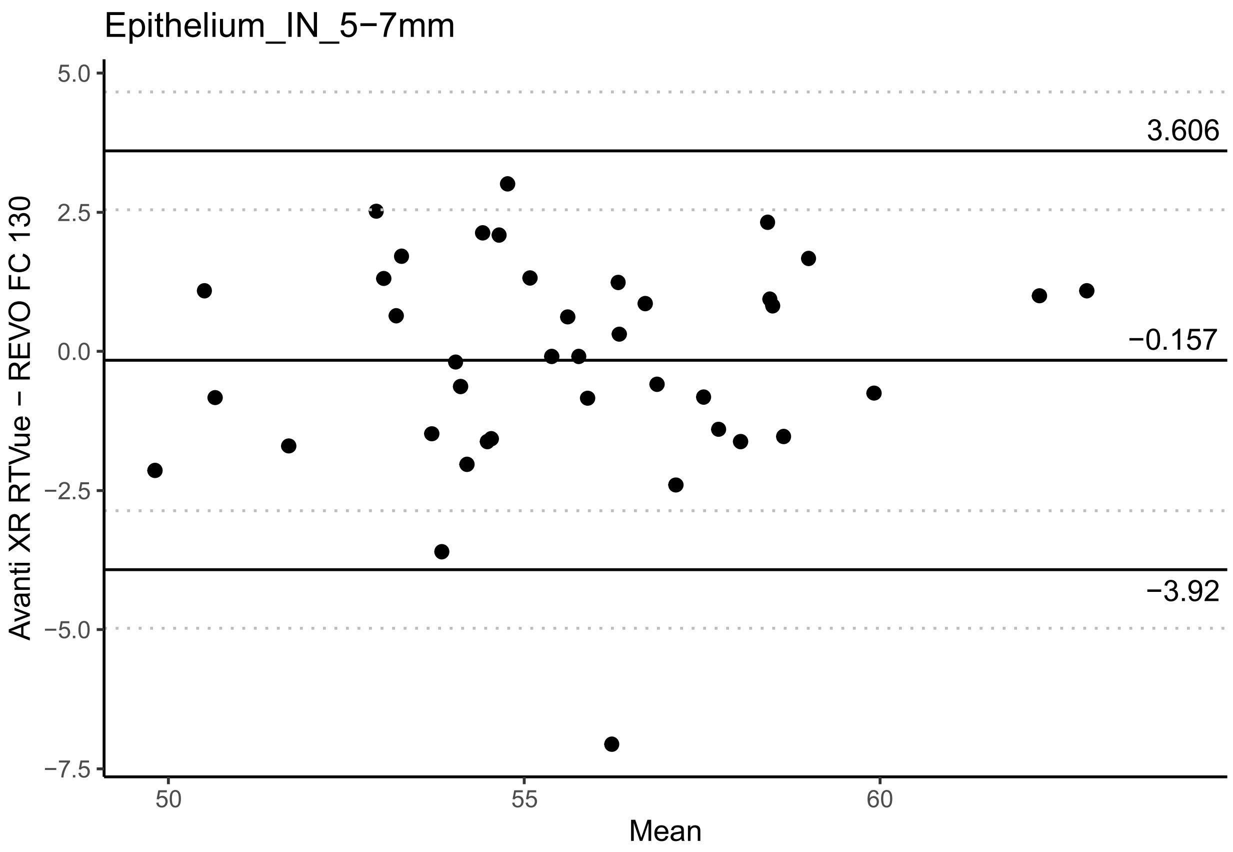

Table 125: Shapiro-Wilk Test Results

Test	W_statistic	p_value
Avanti RTVue	0.9804043	0.7045606
REVO FC 130	0.9870263	0.9200486
Differences	0.9261439	0.0120785

Table 126: Paired T-test results

	Test	t_statistic	p_value
t	Paired t-test for mean differences	-0.5179949	0.6073881

Table 127: Bland Altman Statistics

Sector	Mean_Diff	SD	upperLOA	lower_CI	upper_CI	lowerLOA	lower_CI_	upper_CI_
Epithelium_IN_5-7mm	-0.1572	1.92	3.6059	2.5477	4.6641	-3.9204	-4.9786	-2.8622

Table 128: Basic Statistics

Mean_Avanti	SD1	Min1	Max1	Mean_REVO	SD2	Min2	Max2
55.5827	3.1257	48.74	63.45	55.74	2.9591	49.96	62.36

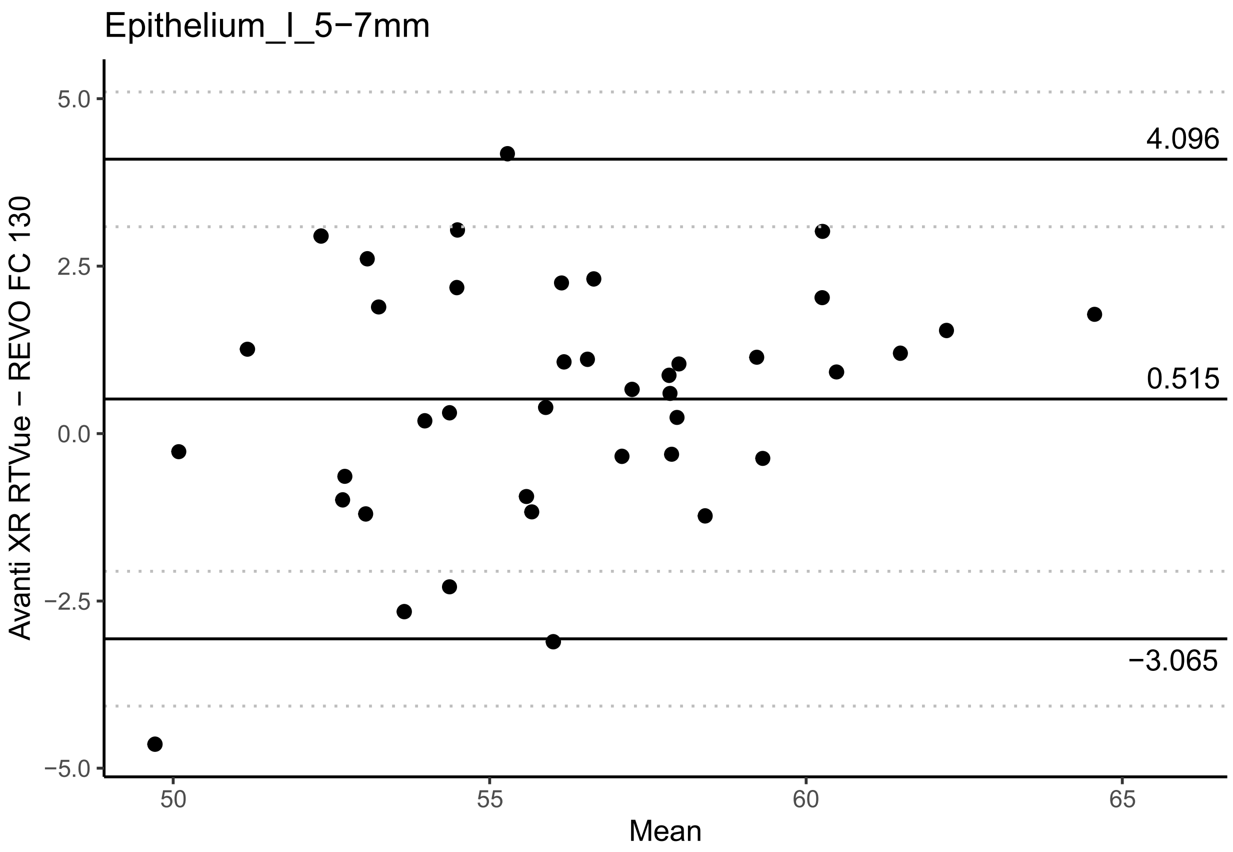

Table 129: Shapiro-Wilk Test Results

Test	W_statistic	p_value
Avanti RTVue	0.9894007	0.9664335
REVO FC 130	0.9855015	0.8793130
Differences	0.9757233	0.5345744

Table 130: Paired T-test results

	Test	t_statistic	p_value
t	Paired t-test for mean differences	1.784592	0.0821083

Table 131: Bland Altman Statistics

Sector	Mean_Diff	SD	upperLOA	lower_CI	upper_CI	lowerLOA	lower_CI_	upper_CI_
Epithelium_I_5-7mm	0.5155	1.8269	4.0963	3.0893	5.1032	-3.0653	-4.0722	-2.0583

Table 132: Basic Statistics

Mean_Avanti	SD1	Min1	Max1	Mean_REVO	SD2	Min2	Max2
56.441	3.6734	47.39	65.45	55.9255	3.1525	50.22	63.67

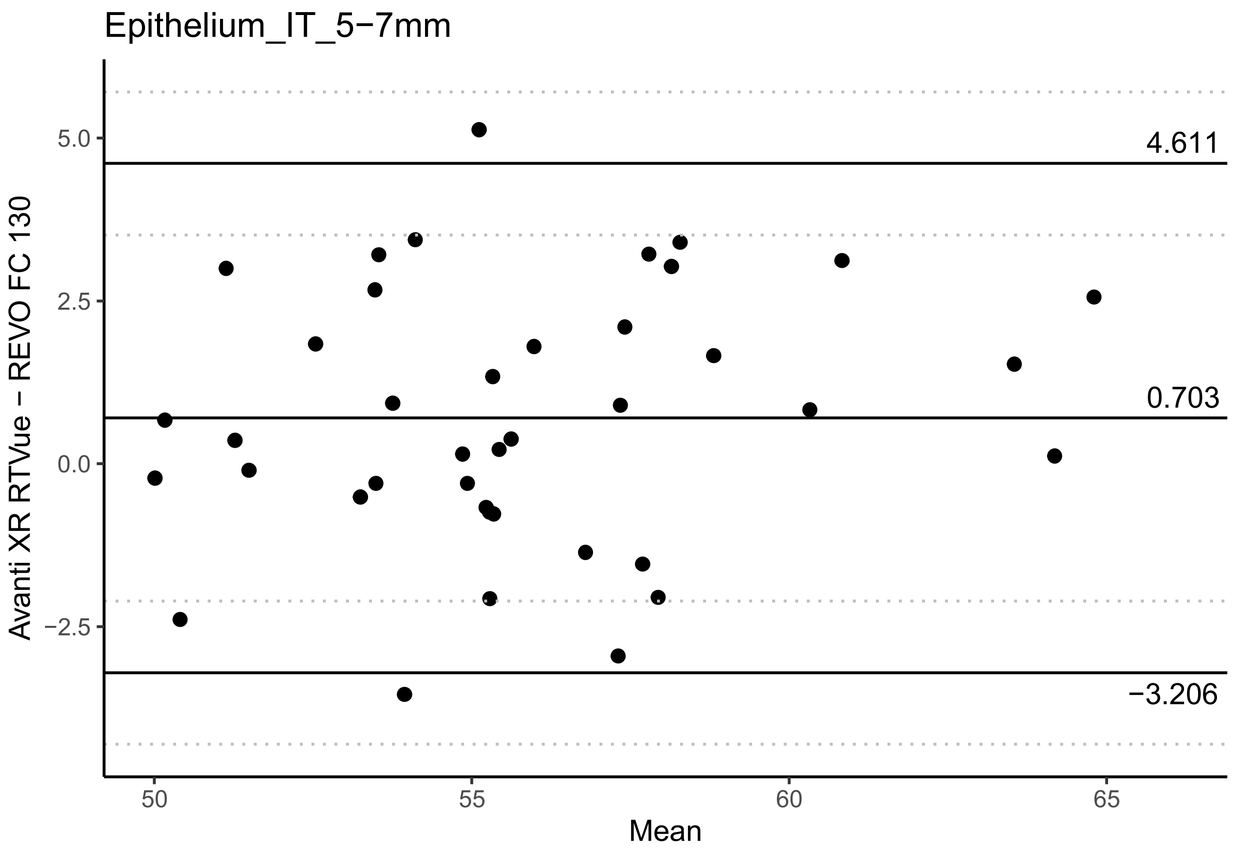

Table 133: Shapiro-Wilk Test Results

Test	W_statistic	p_value
Avanti RTVue	0.9600128	0.1676675
REVO FC 130	0.9599460	0.1667869
Differences	0.9836434	0.8206364

Table 134: Paired T-test results

	Test	t_statistic	p_value
t	Paired t-test for mean differences	2.228059	0.0317145

Table 135: Bland Altman Statistics

Sector	Mean_Diff	SD	upperLOA	lower_CI	upper_CI	lowerLOA	lower_CI_	upper_CI_
Epithelium_IT_5-7mm	0.7025	1.9941	4.611	3.5119	5.71	-3.206	-4.305	-2.1069

Table 136: Basic Statistics

Mean_Avanti	SD1	Min1	Max1	Mean_REVO	SD2	Min2	Max2
56.1545	3.8829	49.21	66.08	55.452	3.546	49.63	64.12

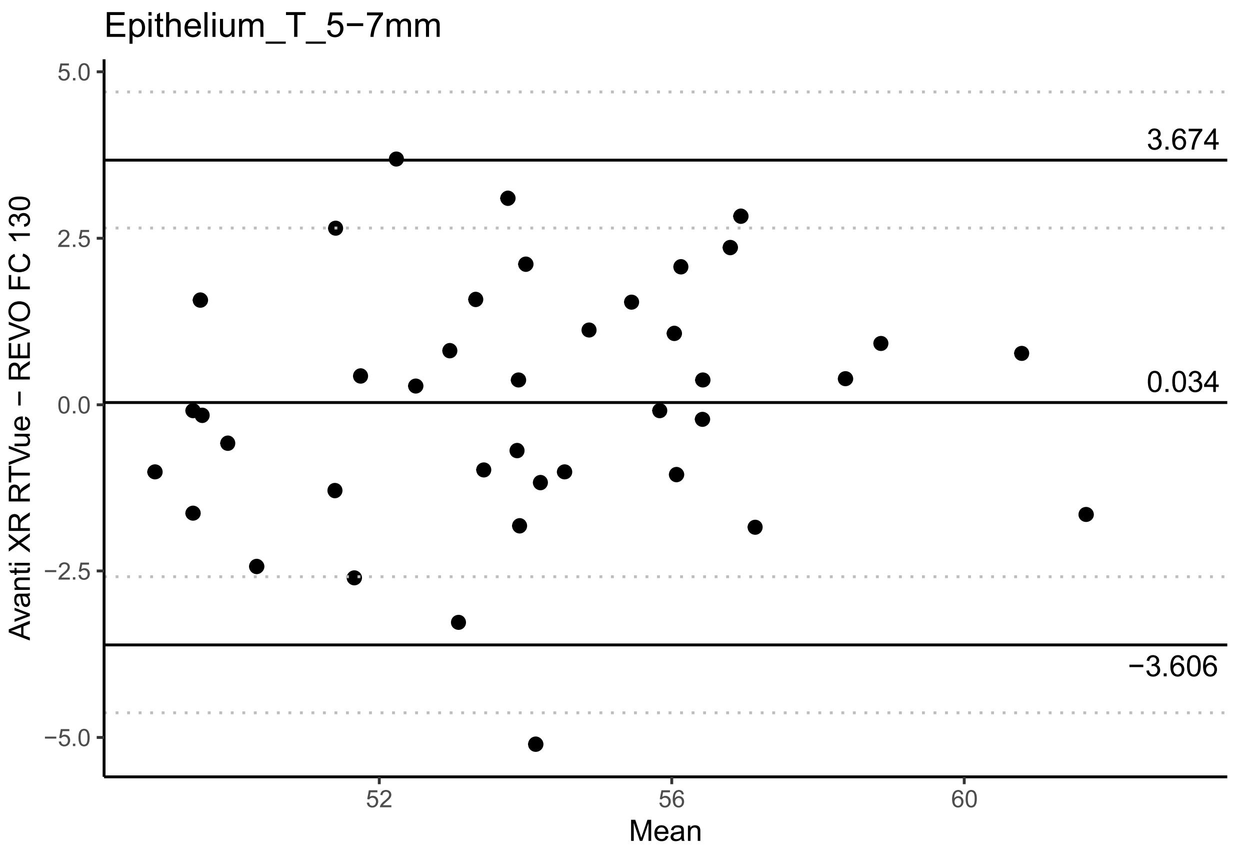

Table 137: Shapiro-Wilk Test Results

Test	W_statistic	p_value
Avanti RTVue	0.9758101	0.5375690
REVO FC 130	0.9707666	0.3804812
Differences	0.9872144	0.9245189

Table 138: Paired T-test results

	Test	t_statistic	p_value
t	Paired t-test for mean differences	0.1149327	0.9090882

Table 139: Bland Altman Statistics

Sector	Mean_Diff	SD	upperLOA	lower_CI	upper_CI	lowerLOA	lower_CI_	upper_CI_
Epithelium_T_5-7mm	0.0337	1.8572	3.6739	2.6503	4.6975	-3.6064	-4.63	-2.5828

Table 140: Basic Statistics

Mean_Avanti	SD1	Min1	Max1	Mean_REVO	SD2	Min2	Max2
54.045	3.3406	48.43	61.17	54.0112	3.119	48.77	62.49

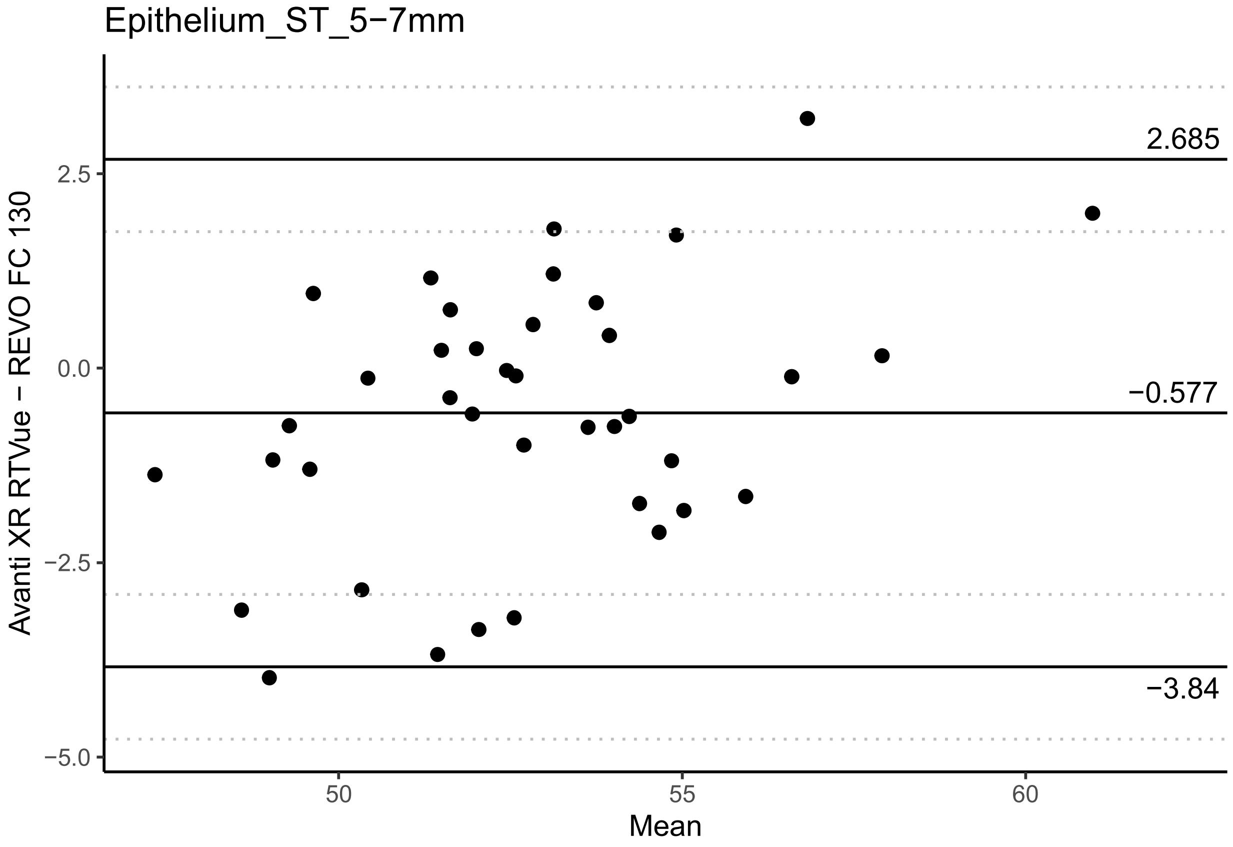

Table 141: Shapiro-Wilk Test Results

Test	W_statistic	p_value
Avanti RTVue	0.9636414	0.2226620
REVO FC 130	0.9869339	0.9235318
Differences	0.9837208	0.8332907

Table 142: Paired T-test results

	Test	t_statistic	p_value
t	Paired t-test for mean differences	-2.166264	0.0366303

Table 143: Bland Altman Statistics

Sector	Mean_Diff	SD	upperLOA	lower_CI	upper_CI	lowerLOA	lower_CI_	upper_CI_
Epithelium_ST_5-7mm	-0.5774	1.6647	2.6853	1.7551	3.6155	-3.8402	-4.7703	-2.91

Table 144: Basic Statistics

Mean_Avanti	SD1	Min1	Max1	Mean_REVO	SD2	Min2	Max2
52.4726	3.1989	46.64	61.97	53.05	2.5436	48.01	59.98

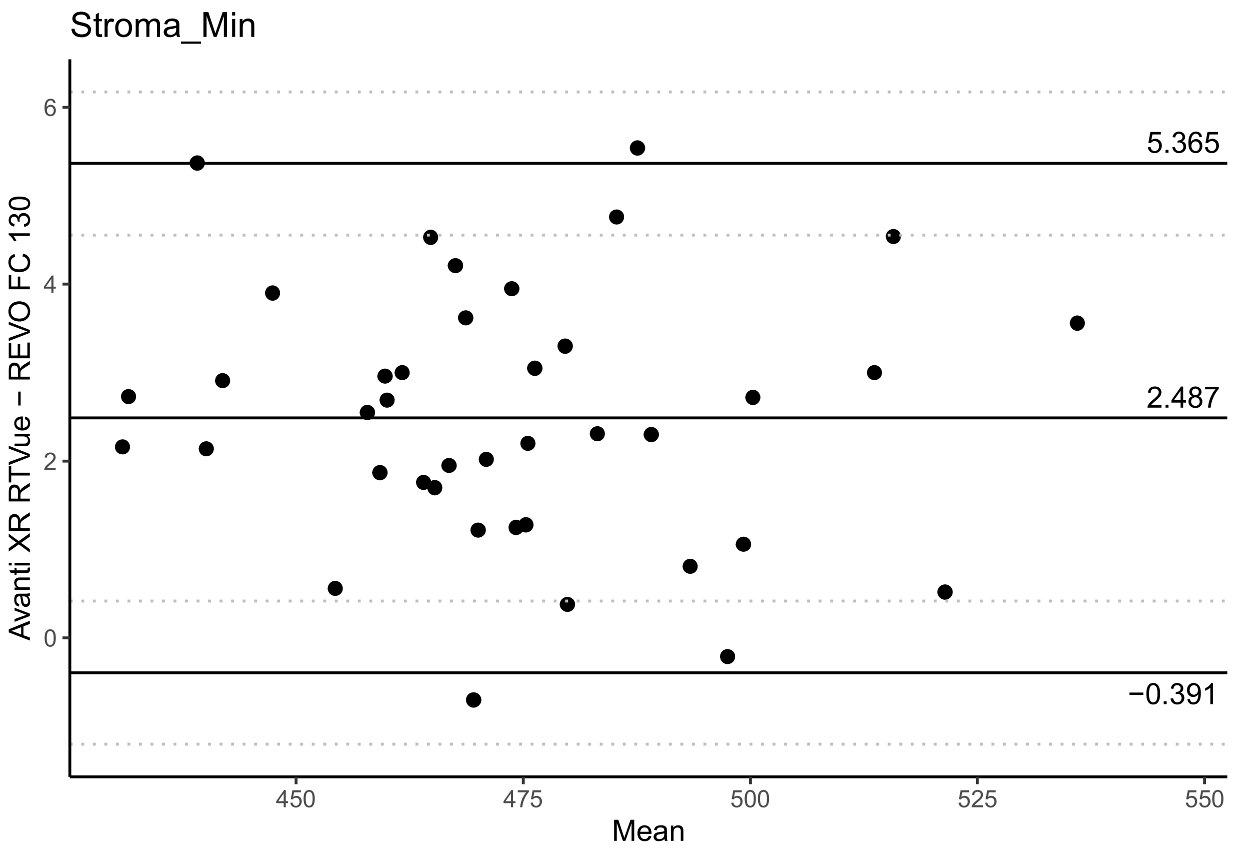

Table 145: Shapiro-Wilk Test Results

Test	W_statistic	p_value
Avanti RTVue	0.9701934	0.3650173
REVO FC 130	0.9702604	0.3667974
Differences	0.9906451	0.9818523

Table 146: Paired T-test results

	Test	t_statistic	p_value
t	Paired t-test for mean differences	10.71027	0

Table 147: Bland Altman Statistics

Sector	Mean_Diff	SD	upperLOA	lower_CI	upper_CI	lowerLOA	lower_CI_	upper_CI_
Stroma_Min	2.4867	1.4685	5.3649	4.5556	6.1743	-0.3914	-1.2008	0.4179

Table 148: Basic Statistics

Mean_Avanti	SD1	Min1	Max1	Mean_REVO	SD2	Min2	Max2
474.9612	23.6863	431.99	537.77	472.4745	23.7903	429.83	534.21

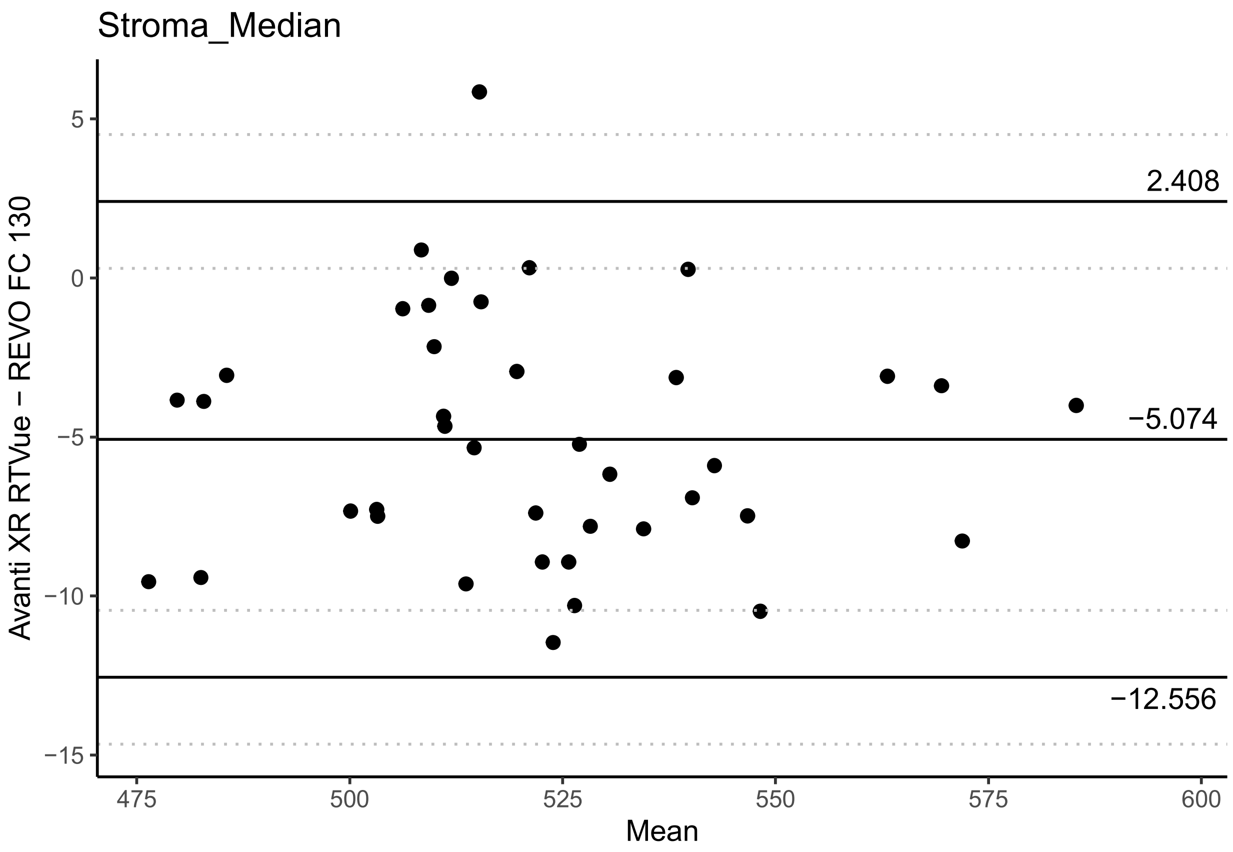

Table 149: Shapiro-Wilk Test Results

Test	W_statistic	p_value
Avanti RTVue	0.9611766	0.1837229
REVO FC 130	0.9663703	0.2745731
Differences	0.9641740	0.2320291

Table 150: Paired T-test results

	Test	t_statistic	p_value
t	Paired t-test for mean differences	-8.406107	0

Table 151: Bland Altman Statistics

Sector	Mean_Diff	SD	upperLOA	lower_CI	upper_CI	lowerLOA	lower_CI_	upper_CI_
Stroma_Median	-5.074	3.8176	2.4084	0.3043	4.5125	-12.5564	-14.6605	-10.4523

Table 152: Basic Statistics

Mean_Avanti	SD1	Min1	Max1	Mean_REVO	SD2	Min2	Max2
519.1572	24.8758	471.61	583.31	524.2313	25.0816	481.16	587.32

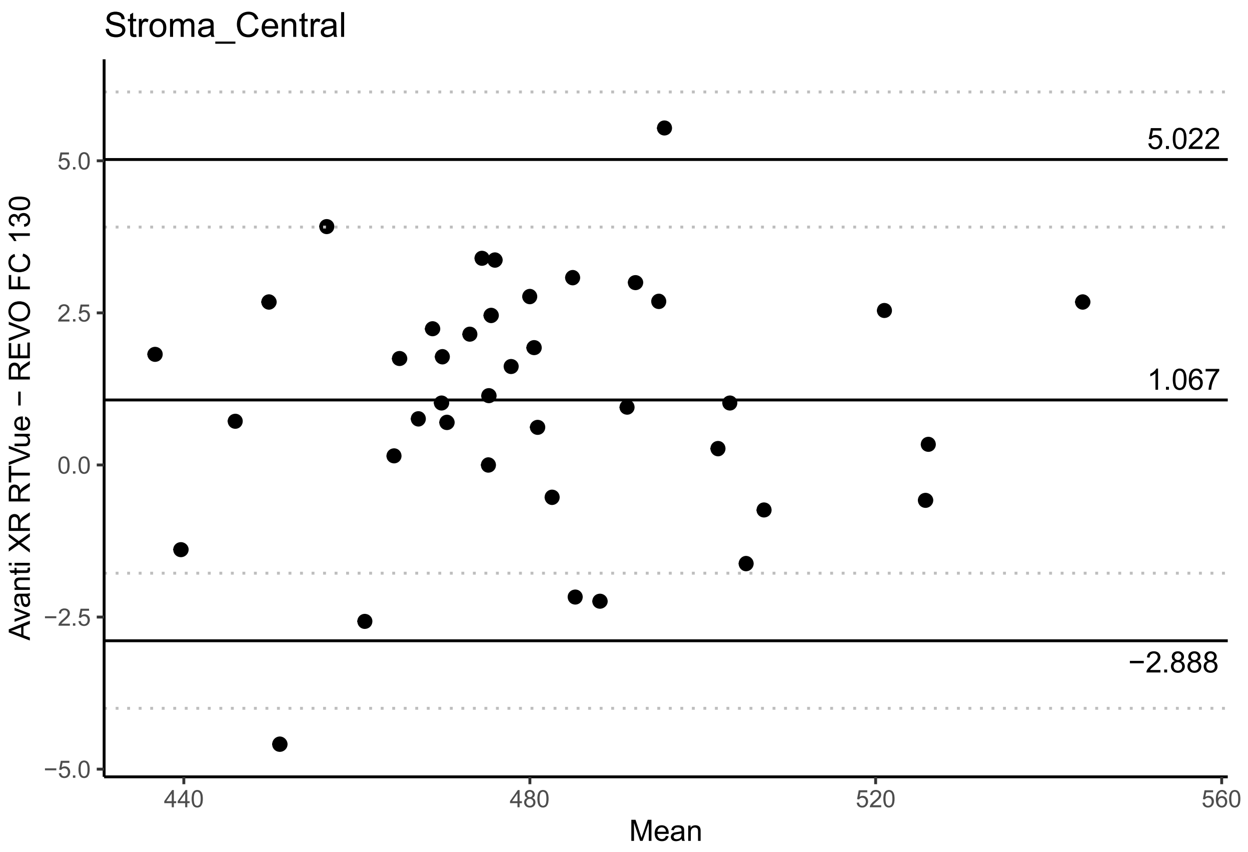

Table 153: Shapiro-Wilk Test Results

Test	W_statistic	p_value
Avanti RTVue	0.9695596	0.3485065
REVO FC 130	0.9692532	0.3407443
Differences	0.9728976	0.4423827

Table 154: Paired T-test results

	Test	t_statistic	p_value
t	Paired t-test for mean differences	3.344096	0.0018328

Table 155: Bland Altman Statistics

Sector	Mean_Diff	SD	upperLOA	lower_CI	upper_CI	lowerLOA	lower_CI_	upper_CI_
Stroma_Central	1.067	2.018	5.0222	3.91	6.1345	-2.8882	-4.0005	-1.776

Table 156: Basic Statistics

Mean_Avanti	SD1	Min1	Max1	Mean_REVO	SD2	Min2	Max2
481.3608	23.7221	437.57	545.28	480.2938	23.5559	435.75	542.6

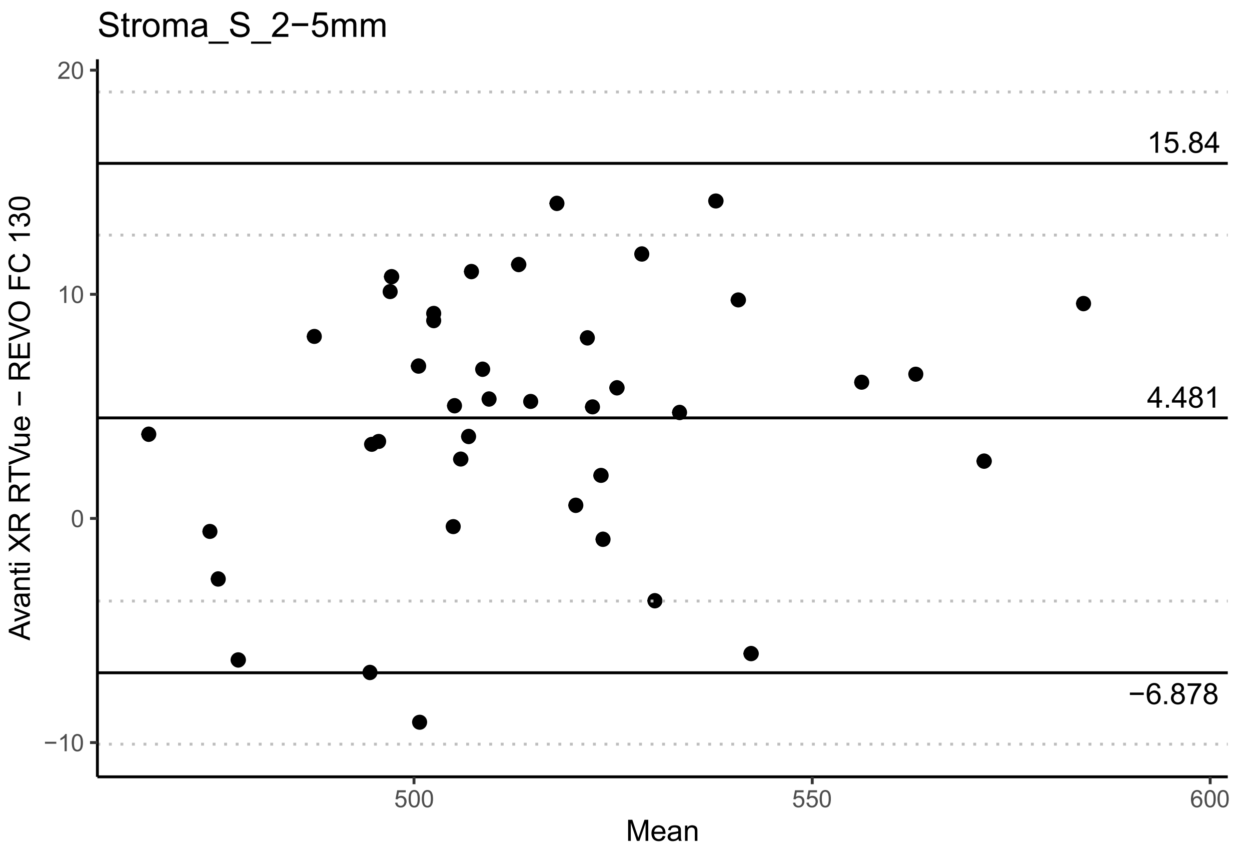

Table 157: Shapiro-Wilk Test Results

Test	W_statistic	p_value
Avanti RTVue	0.9646346	0.2404214
REVO FC 130	0.9580559	0.1436703
Differences	0.9634327	0.2190874

Table 158: Paired T-test results

	Test	t_statistic	p_value
t	Paired t-test for mean differences	4.889942	1.77e-05

Table 159: Bland Altman Statistics

Sector	Mean_Diff	SD	upperLOA	lower_CI	upper_CI	lowerLOA	lower_CI_	upper_CI_
Stroma_S_2-5mm	4.481	5.7956	15.8404	12.6461	19.0348	-6.8784	-10.0728	-3.6841

Table 160: Basic Statistics

Mean_Avanti	SD1	Min1	Max1	Mean_REVO	SD2	Min2	Max2
516.8797	26.5598	468.53	588.89	512.3987	25.2178	464.77	579.3

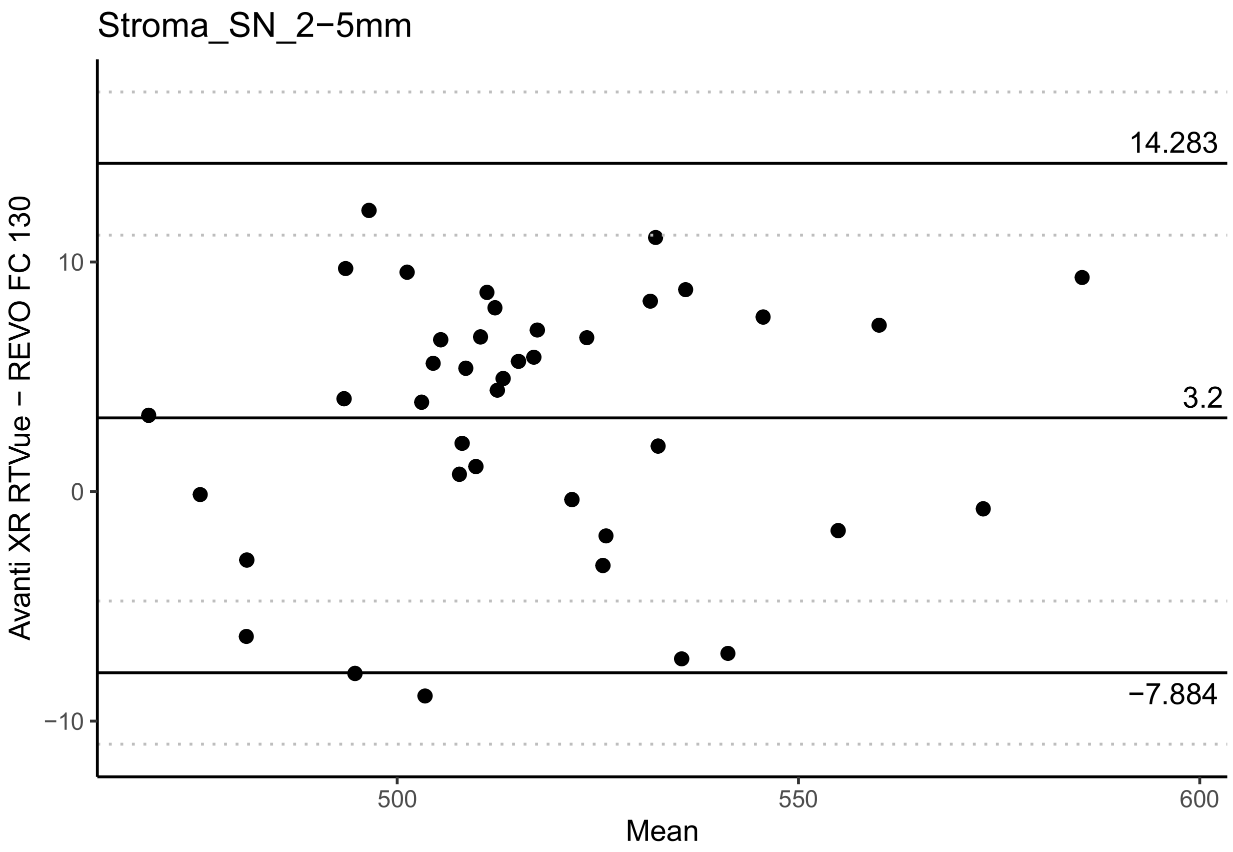

Table 161: Shapiro-Wilk Test Results

Test	W_statistic	p_value
Avanti RTVue	0.9650349	0.2479377
REVO FC 130	0.9602714	0.1711143
Differences	0.9399601	0.0344729

Table 162: Paired T-test results

	Test	t_statistic	p_value
t	Paired t-test for mean differences	3.578634	0.0009428

Table 163: Bland Altman Statistics

Sector	Mean_Diff	SD	upperLOA	lower_CI	upper_CI	lowerLOA	lower_CI_	upper_CI_
Stroma_SN_2-5mm	3.1998	5.655	14.2835	11.1667	17.4002	-7.884	-11.0007	-4.7672

Table 164: Basic Statistics

Mean_Avanti	SD1	Min1	Max1	Mean_REVO	SD2	Min2	Max2
518.3595	25.2928	470.66	590.01	515.1598	24.7195	467.34	580.69

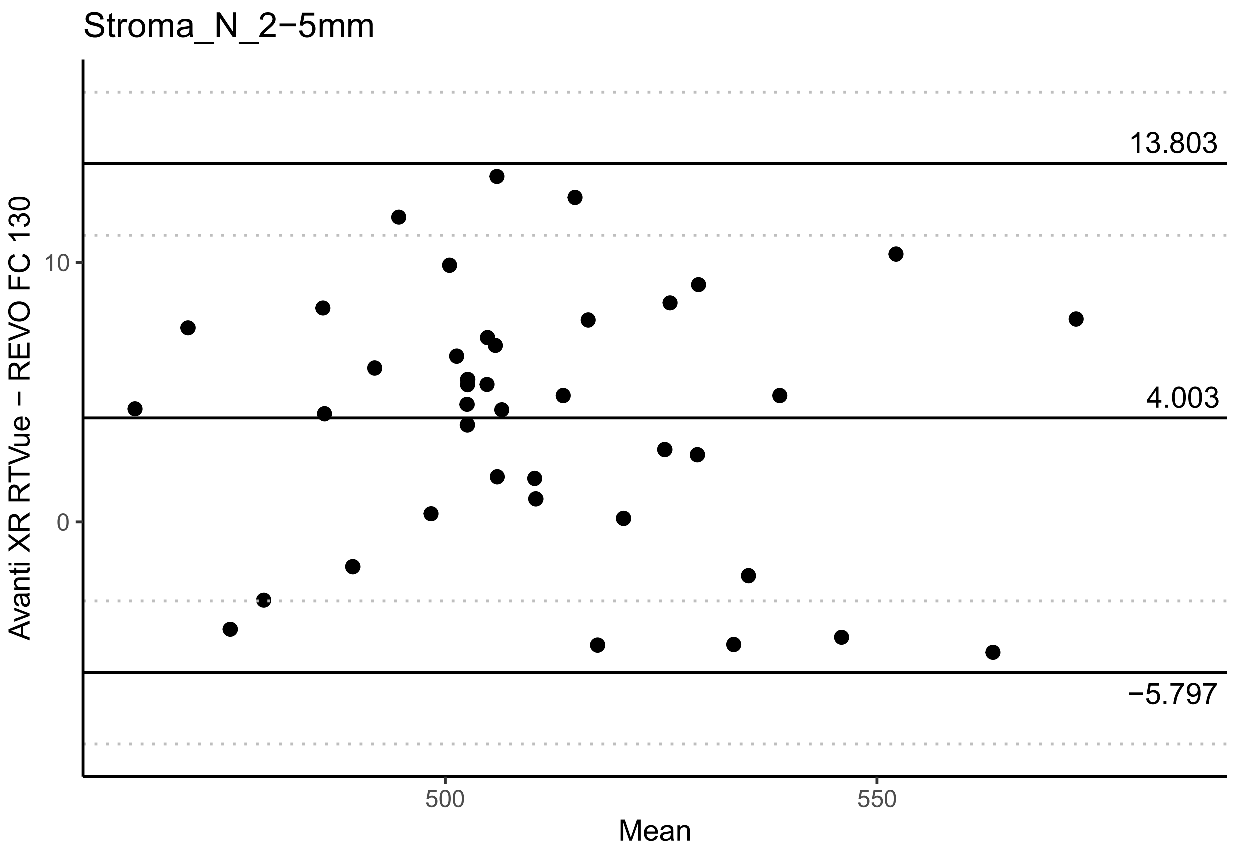

Table 165: Shapiro-Wilk Test Results

Test	W_statistic	p_value
Avanti RTVue	0.9696049	0.3496658
REVO FC 130	0.9654105	0.2551838
Differences	0.9616205	0.1902249

Table 166: Paired T-test results

	Test	t_statistic	p_value
t	Paired t-test for mean differences	5.063359	1.03e-05

Table 167: Bland Altman Statistics

Sector	Mean_Diff	SD	upperLOA	lower_CI	upper_CI	lowerLOA	lower_CI_	upper_CI_
Stroma_N_2-5mm	4.003	5.0001	13.8032	11.0473	16.559	-5.7972	-8.553	-3.0413

Table 168: Basic Statistics

Mean_Avanti	SD1	Min1	Max1	Mean_REVO	SD2	Min2	Max2
512.919	23.6523	466.27	576.95	508.916	24.1246	461.91	569.13

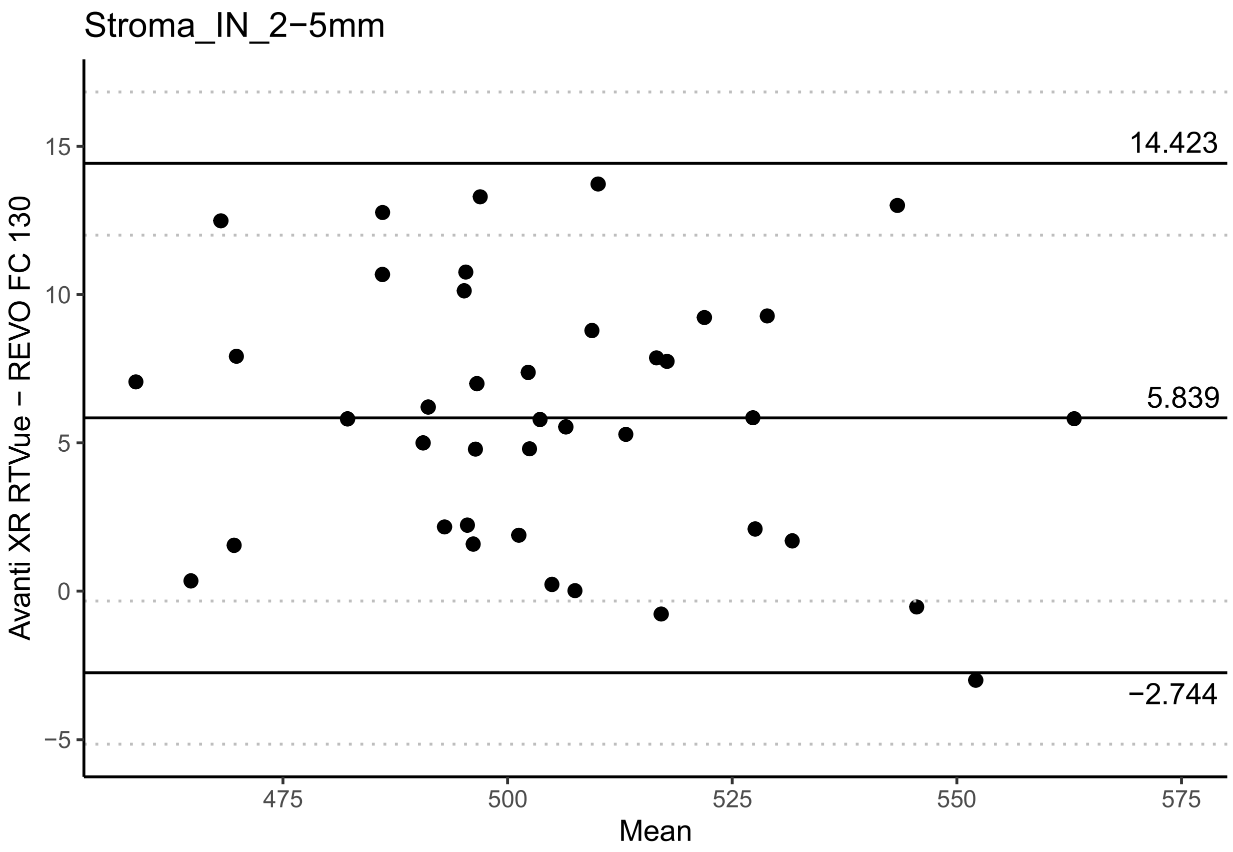

Table 169: Shapiro-Wilk Test Results

Test	W_statistic	p_value
Avanti RTVue	0.9764619	0.5603252
REVO FC 130	0.9773174	0.5908421
Differences	0.9697216	0.3526663

Table 170: Paired T-test results

	Test	t_statistic	p_value
t	Paired t-test for mean differences	8.433409	0

Table 171: Bland Altman Statistics

Sector	Mean_Diff	SD	upperLOA	lower_CI	upper_CI	lowerLOA	lower_CI_	upper_CI_
Stroma_IN_2-5mm	5.8395	4.3793	14.4229	12.0092	16.8366	-2.7439	-5.1576	-0.3302

Table 172: Basic Statistics

Mean_Avanti	SD1	Min1	Max1	Mean_REVO	SD2	Min2	Max2
507.5642	23.3908	462.15	565.97	501.7247	24.1381	455.09	560.15

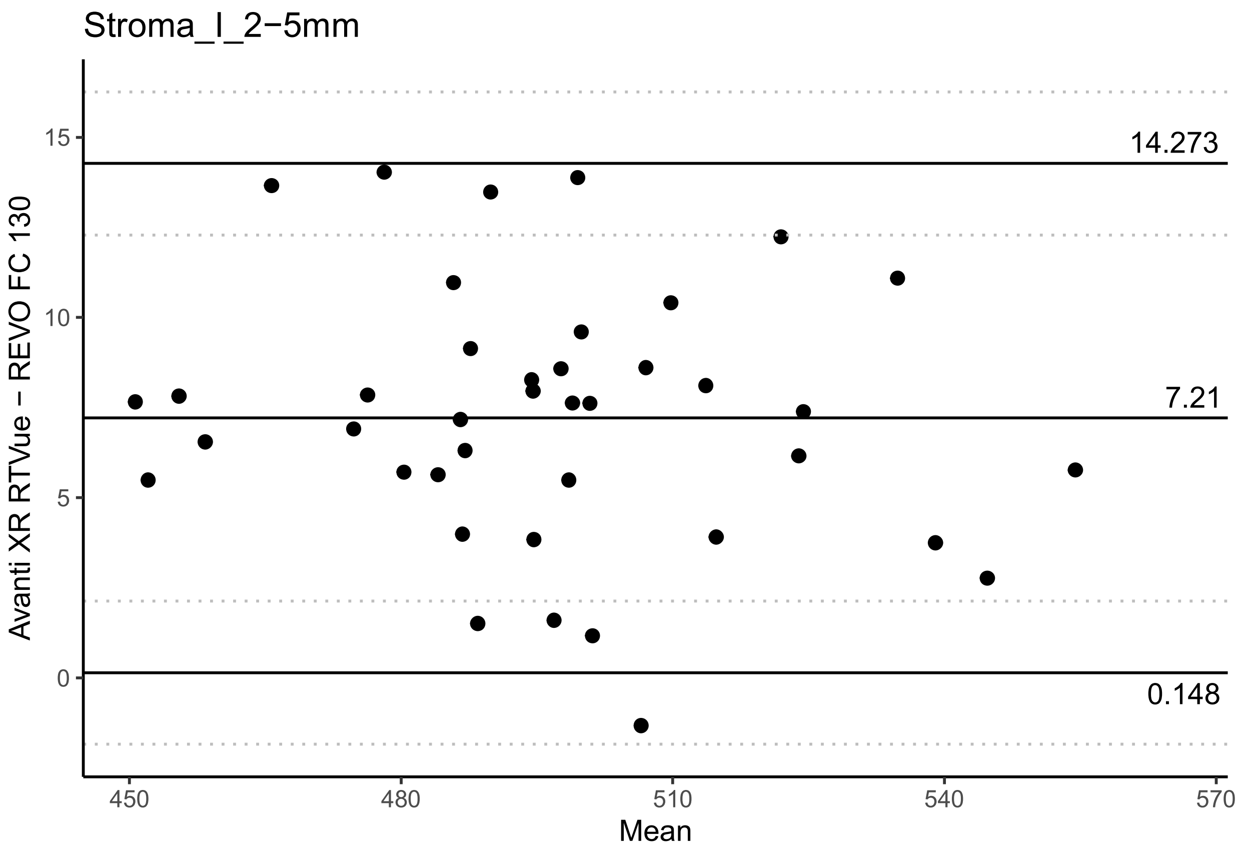

Table 173: Shapiro-Wilk Test Results

Test	W_statistic	p_value
Avanti RTVue	0.9724074	0.4275357
REVO FC 130	0.9746261	0.4975071
Differences	0.9752826	0.5195073

Table 174: Paired T-test results

	Test	t_statistic	p_value
t	Paired t-test for mean differences	12.65516	0

Table 175: Bland Altman Statistics

Sector	Mean_Diff	SD	upperLOA	lower_CI	upper_CI	lowerLOA	lower_CI_	upper_CI_
Stroma_I_2-5mm	7.2105	3.6035	14.2734	12.2873	16.2595	0.1476	-1.8385	2.1337

Table 176: Basic Statistics

Mean_Avanti	SD1	Min1	Max1	Mean_REVO	SD2	Min2	Max2
500.11	23.8813	454.48	557.35	492.8995	24.4192	446.82	551.58

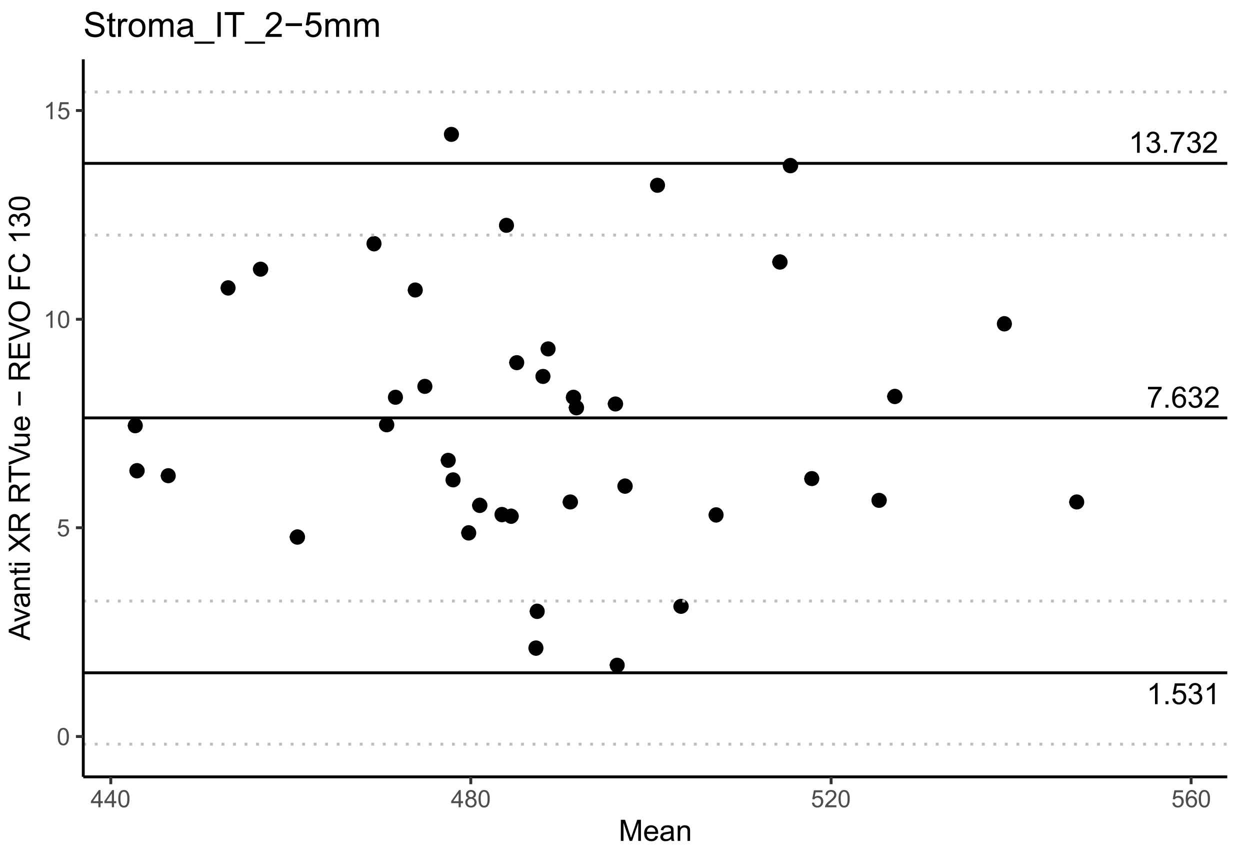

Table 177: Shapiro-Wilk Test Results

Test	W_statistic	p_value
Avanti RTVue	0.9684147	0.3202306
REVO FC 130	0.9764624	0.5603430
Differences	0.9717917	0.4093958

Table 178: Paired T-test results

	Test	t_statistic	p_value
t	Paired t-test for mean differences	15.5079	0

Table 179: Bland Altman Statistics

Sector	Mean_Diff	SD	upperLOA	lower_CI	upper_CI	lowerLOA	lower_CI_	upper_CI_
Stroma_IT_2-5mm	7.6318	3.1124	13.7321	12.0167	15.4476	1.5314	-0.1841	3.2468

Table 180: Basic Statistics

Mean_Avanti	SD1	Min1	Max1	Mean_REVO	SD2	Min2	Max2
491.4798	24.2577	446.11	550.1	483.848	24.3657	439.01	544.48

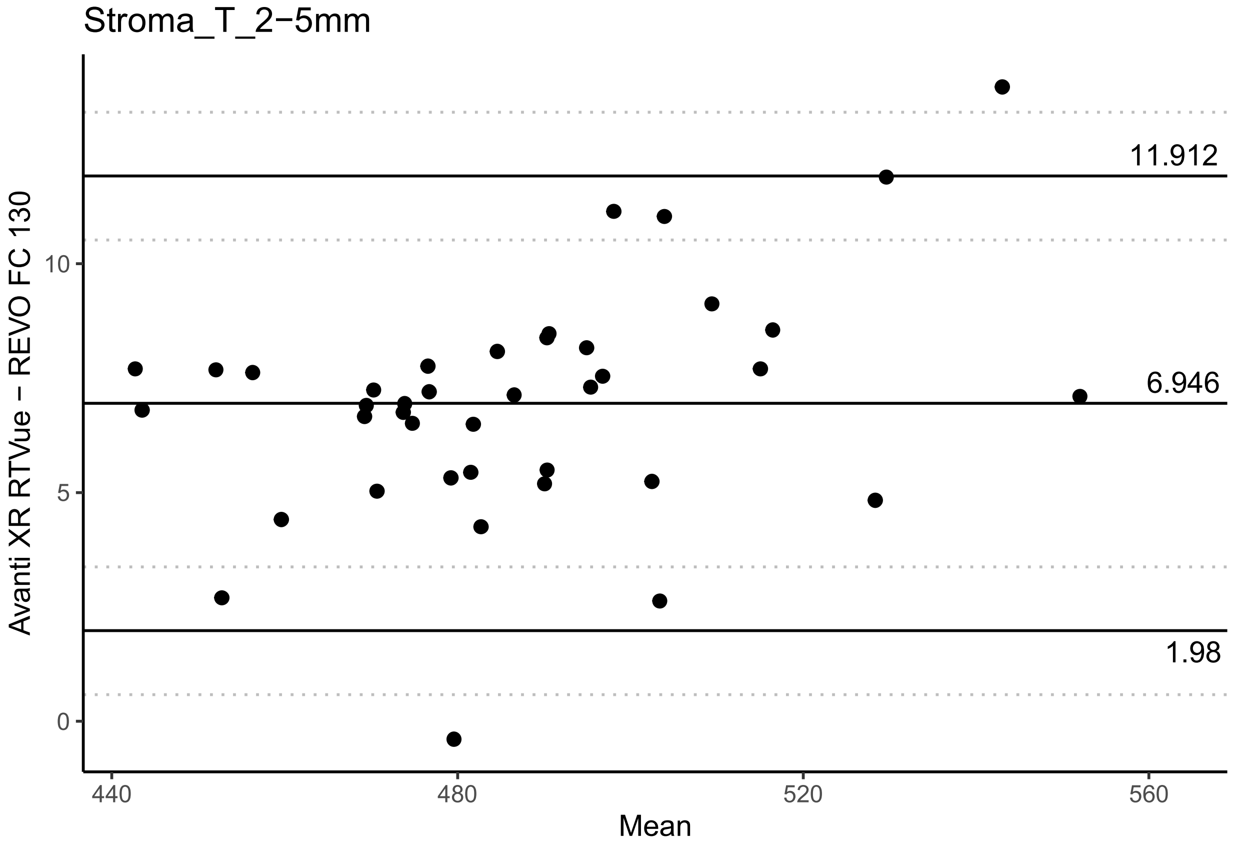

Table 181: Shapiro-Wilk Test Results

Test	W_statistic	p_value
Avanti RTVue	0.9649880	0.2470463
REVO FC 130	0.9724176	0.4278411
Differences	0.9473103	0.0612996

Table 182: Paired T-test results

	Test	t_statistic	p_value
t	Paired t-test for mean differences	17.33996	0

Table 183: Bland Altman Statistics

Sector	Mean_Diff	SD	upperLOA	lower_CI	upper_CI	lowerLOA	lower_CI_	upper_CI_
Stroma_T_2-5mm	6.946	2.5335	11.9116	10.5153	13.308	1.9804	0.584	3.3767

Table 184: Basic Statistics

Mean_Avanti	SD1	Min1	Max1	Mean_REVO	SD2	Min2	Max2
490.6902	25.4483	446.56	555.57	483.7443	24.4914	438.86	548.47

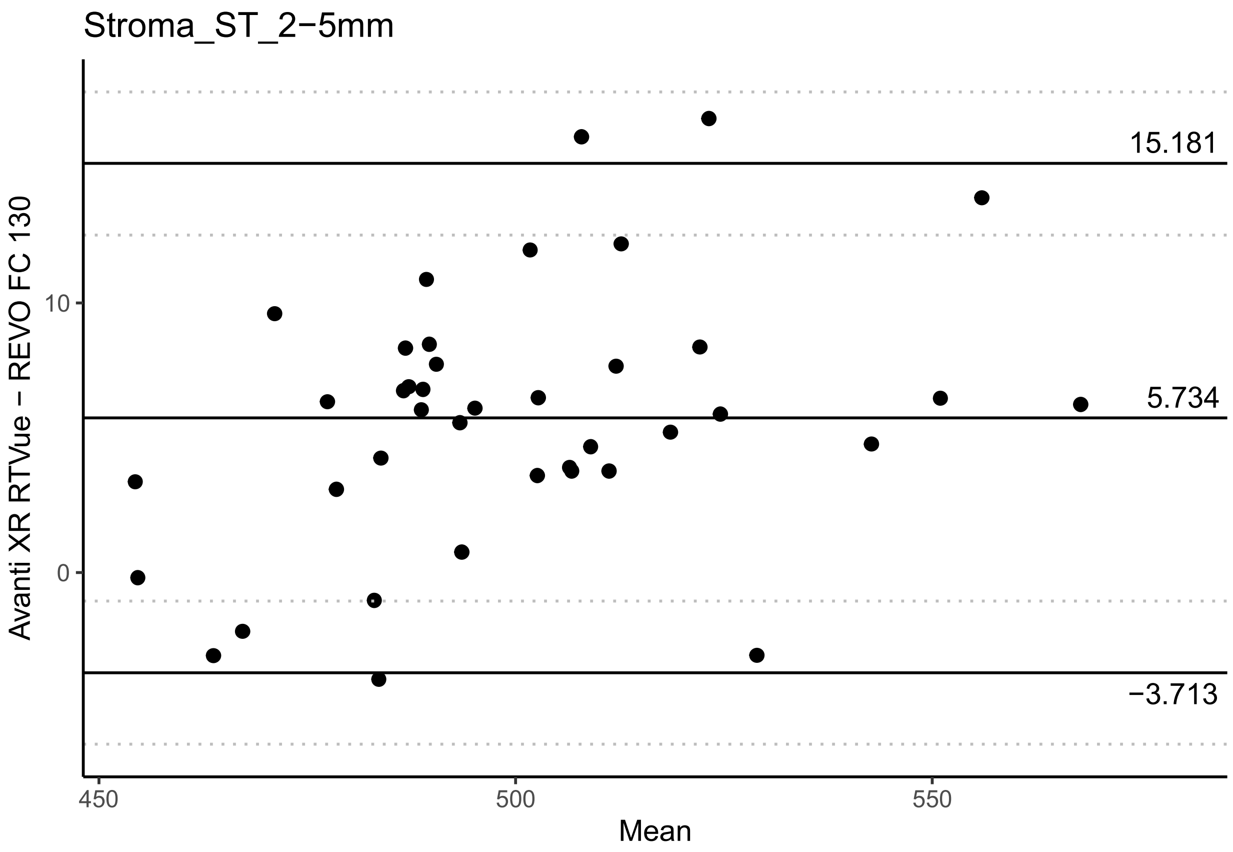

Table 185: Shapiro-Wilk Test Results

Test	W_statistic	p_value
Avanti RTVue	0.9677772	0.3053379
REVO FC 130	0.9553035	0.1155125
Differences	0.9629754	0.2114414

Table 186: Paired T-test results

	Test	t_statistic	p_value
t	Paired t-test for mean differences	7.52415	0

Table 187: Bland Altman Statistics

Sector	Mean_Diff	SD	upperLOA	lower_CI	upper_CI	lowerLOA	lower_CI_	upper_CI_
Stroma_ST_2-5mm	5.7342	4.82	15.1815	12.5249	17.8381	-3.713	-6.3696	-1.0564

Table 188: Basic Statistics

Mean_Avanti	SD1	Min1	Max1	Mean_REVO	SD2	Min2	Max2
503.2398	26.7564	454.57	570.92	497.5055	25.0577	452.68	564.68

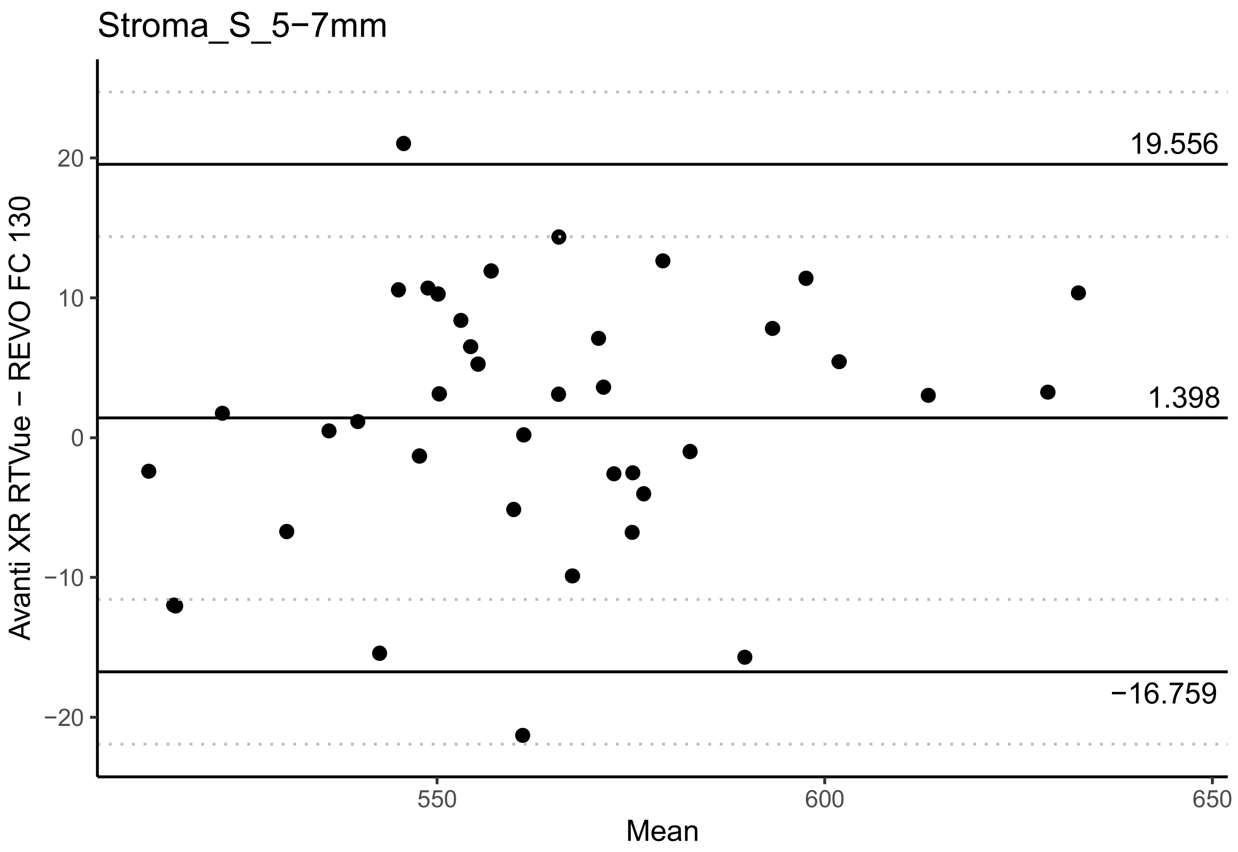

Table 189: Shapiro-Wilk Test Results

Test	W_statistic	p_value
Avanti RTVue	0.9625965	0.2052933
REVO FC 130	0.9690797	0.3517014
Differences	0.9791322	0.6720144

Table 190: Paired T-test results

	Test	t_statistic	p_value
t	Paired t-test for mean differences	0.9427024	0.3517883

Table 191: Bland Altman Statistics

Sector	Mean_Diff	SD	upperLOA	lower_CI	upper_CI	lowerLOA	lower_CI_	upper_CI_
Stroma_S_5-7mm	1.3985	9.2642	19.5563	14.3797	24.7329	-16.7594	-21.936	-11.5828

Table 192: Basic Statistics

Mean_Avanti	SD1	Min1	Max1	Mean_REVO	SD2	Min2	Max2
563.9138	29.6546	510.05	637.87	562.5154	27.6405	514	627.52

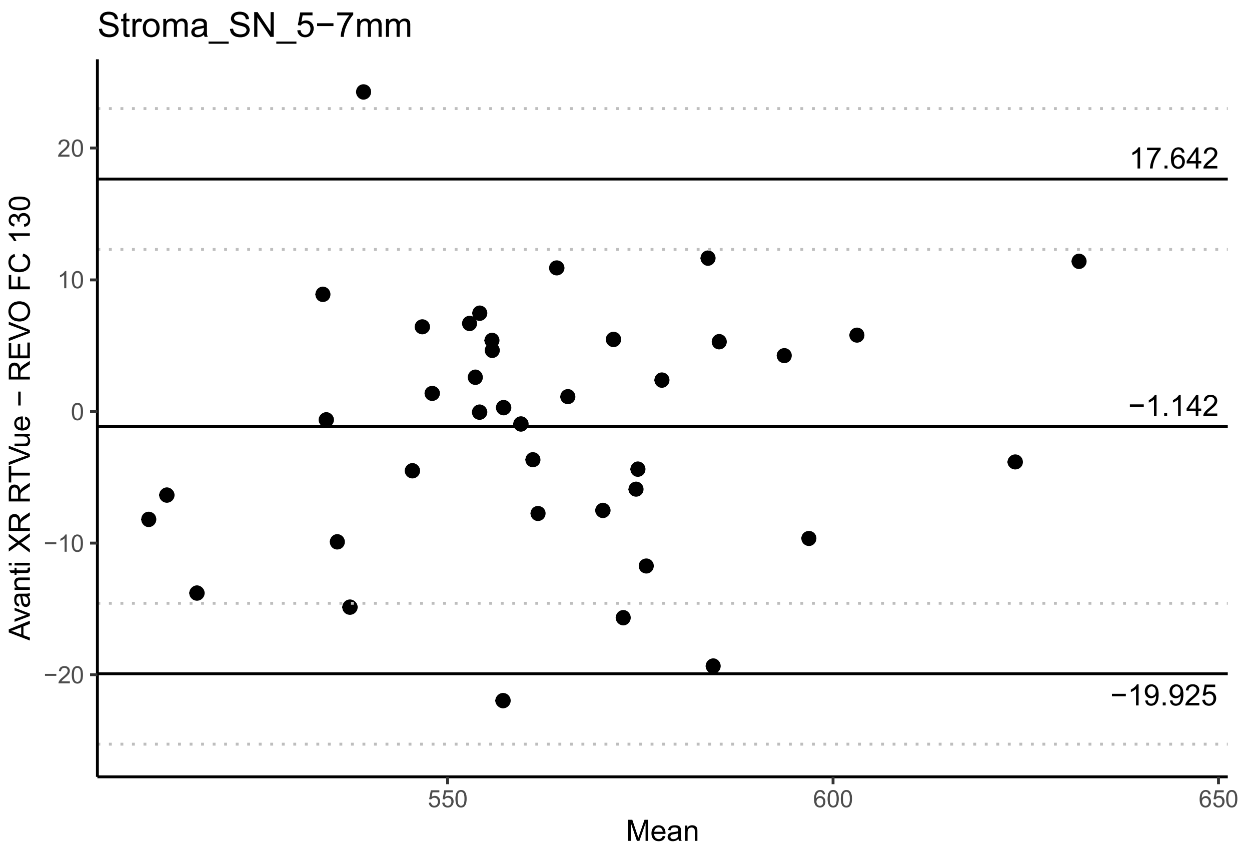

Table 193: Shapiro-Wilk Test Results

Test	W_statistic	p_value
Avanti RTVue	0.9602988	0.1714839
REVO FC 130	0.9751202	0.5302829
Differences	0.9833365	0.8207489

Table 194: Paired T-test results

	Test	t_statistic	p_value
t	Paired t-test for mean differences	-0.7438865	0.4615204

Table 195: Bland Altman Statistics

Sector	Mean_Diff	SD	upperLOA	lower_CI	upper_CI	lowerLOA	lower_CI_	upper_CI_
Stroma_SN_5-7mm	-1.1415	9.5833	17.6418	12.2869	22.9967	-19.9249	-25.2797	-14.57

Table 196: Basic Statistics

Mean_Avanti	SD1	Min1	Max1	Mean_REVO	SD2	Min2	Max2
561.8567	27.4519	507.1	637.61	562.9982	26.2782	515.3	626.21

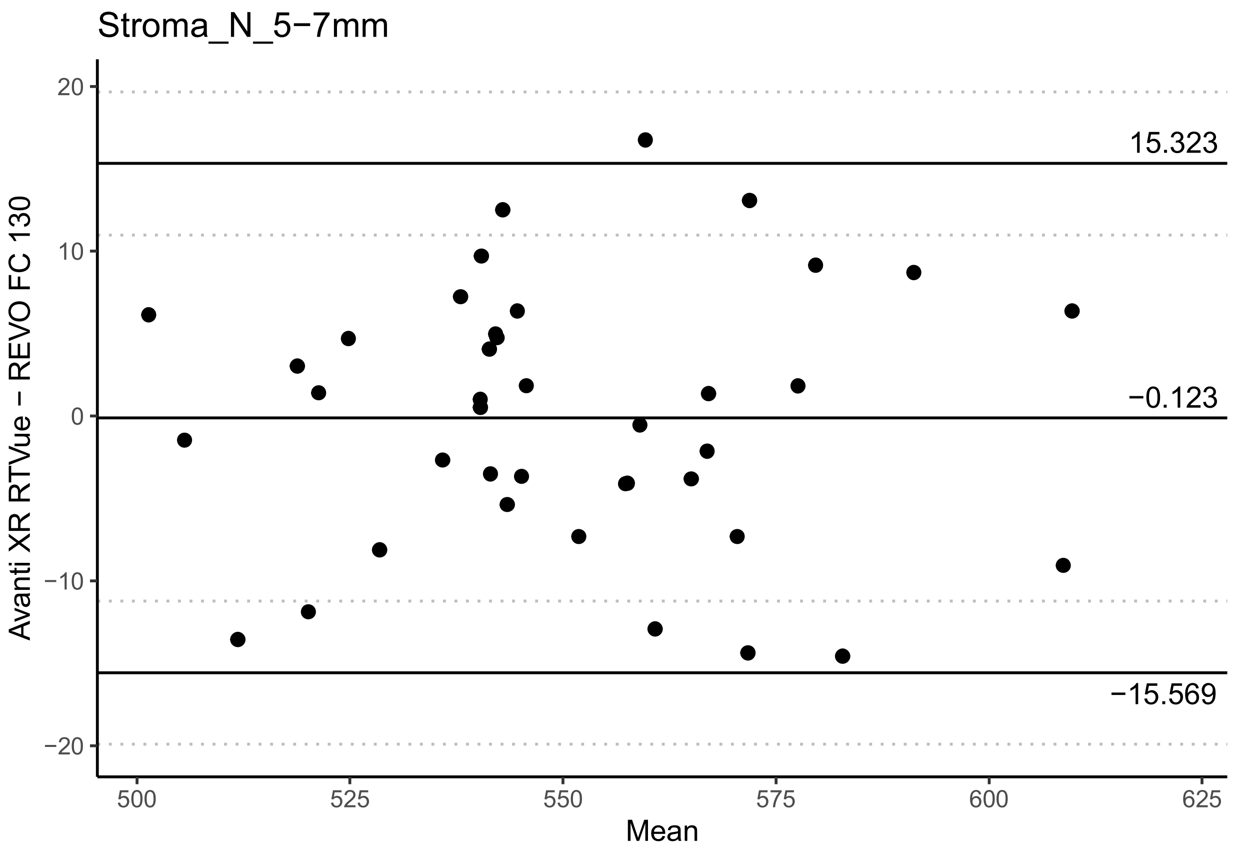

Table 197: Shapiro-Wilk Test Results

Test	W_statistic	p_value
Avanti RTVue	0.9733015	0.4548816
REVO FC 130	0.9803487	0.7024982
Differences	0.9816185	0.7492646

Table 198: Paired T-test results

	Test	t_statistic	p_value
t	Paired t-test for mean differences	-0.098715	0.9218702

Table 199: Bland Altman Statistics

Sector	Mean_Diff	SD	upperLOA	lower_CI	upper_CI	lowerLOA	lower_CI_	upper_CI_
Stroma_N_5-7mm	-0.123	7.8805	15.3227	10.9793	19.6661	-15.5687	-19.9121	-11.2253

Table 200: Basic Statistics

Mean_Avanti	SD1	Min1	Max1	Mean_REVO	SD2	Min2	Max2
550.5695	25.4339	504.44	612.93	550.6925	25.4382	498.3	613.25

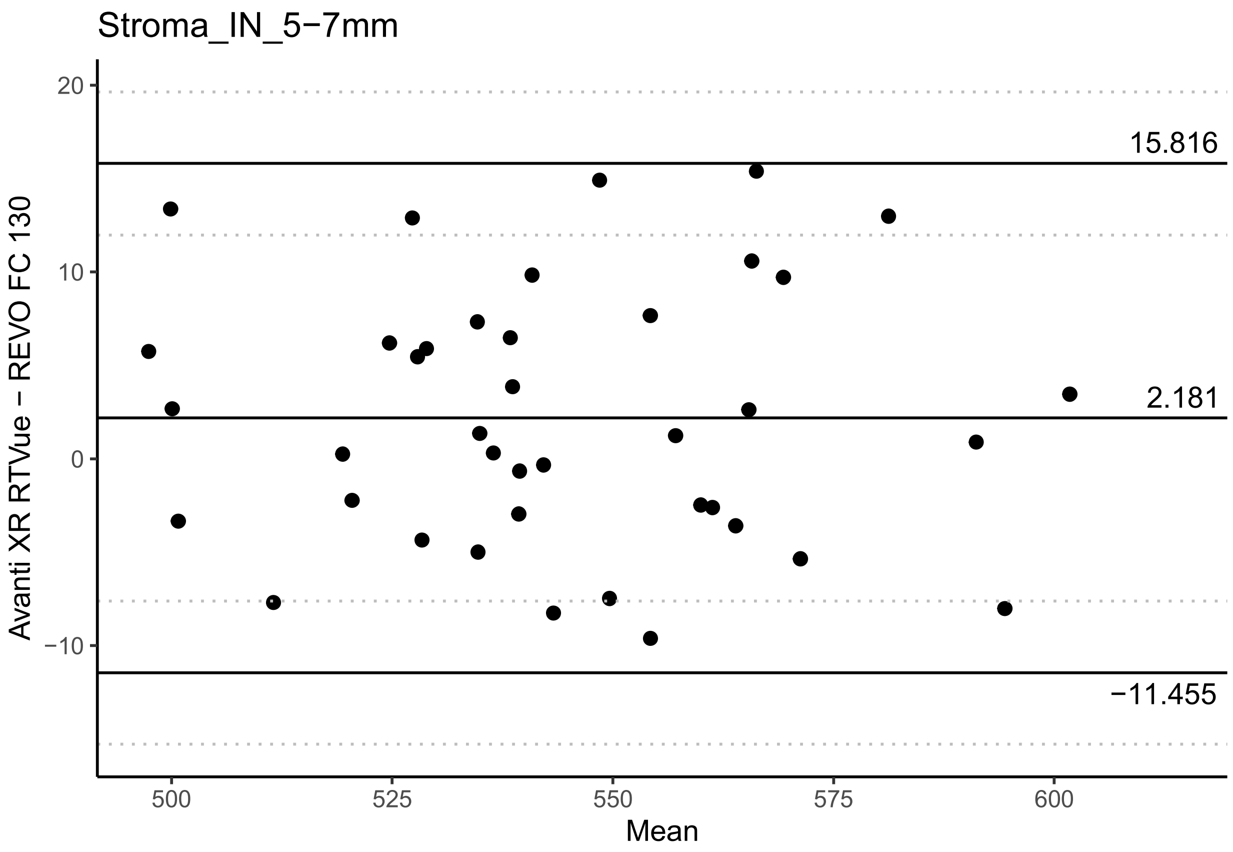

Table 201: Shapiro-Wilk Test Results

Test	W_statistic	p_value
Avanti RTVue	0.9760491	0.5458624
REVO FC 130	0.9774705	0.5963711
Differences	0.9673791	0.2963424

Table 202: Paired T-test results

	Test	t_statistic	p_value
t	Paired t-test for mean differences	1.982346	0.0545177

Table 203: Bland Altman Statistics

Sector	Mean_Diff	SD	upperLOA	lower_CI	upper_CI	lowerLOA	lower_CI_	upper_CI_
Stroma_IN_5-7mm	2.1805	6.9568	15.8157	11.9815	19.65	-11.4547	-15.289	-7.6205

Table 204: Basic Statistics

Mean_Avanti	SD1	Min1	Max1	Mean_REVO	SD2	Min2	Max2
545.2113	25.7789	499.1	603.49	543.0308	25.9535	493.2	600.03

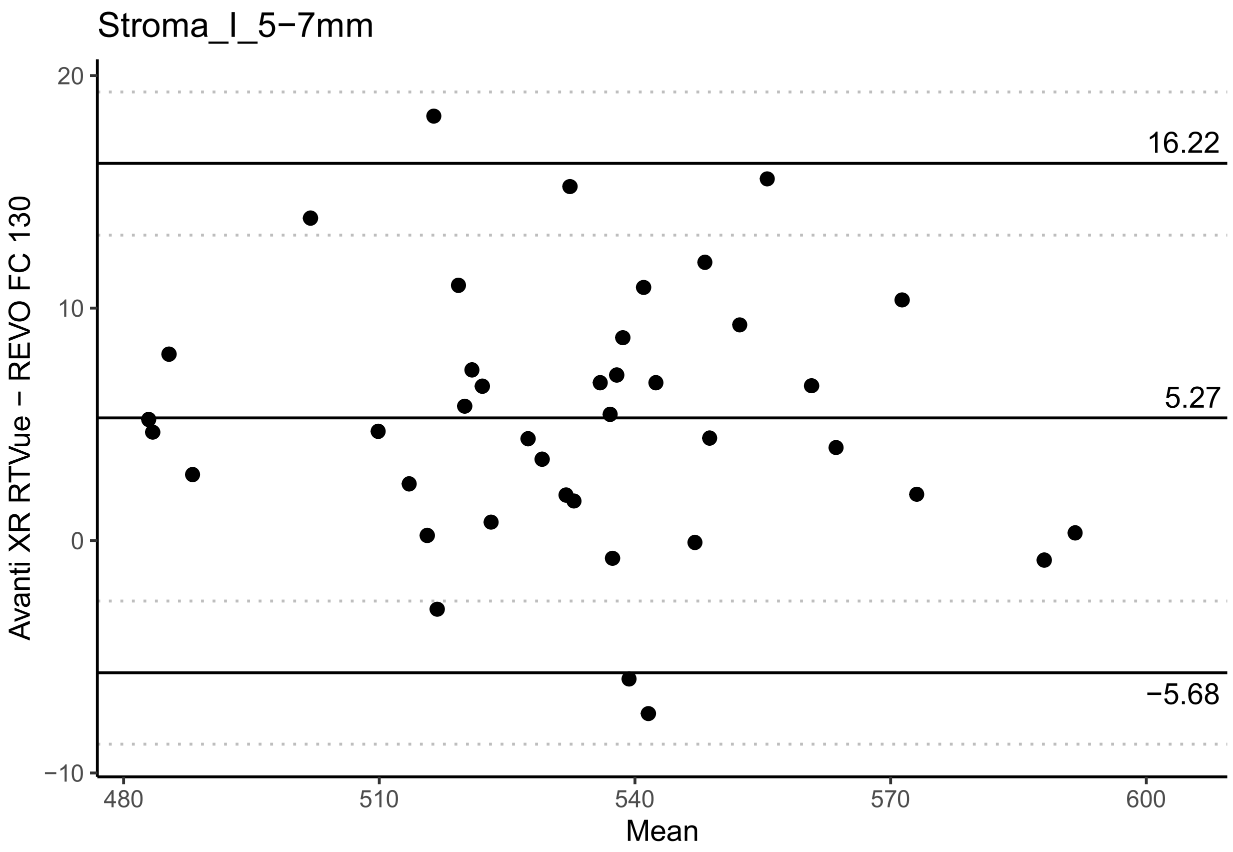

Table 205: Shapiro-Wilk Test Results

Test	W_statistic	p_value
Avanti RTVue	0.9754119	0.5239046
REVO FC 130	0.9719690	0.4145608
Differences	0.9887756	0.9563845

Table 206: Paired T-test results

	Test	t_statistic	p_value
t	Paired t-test for mean differences	5.965747	6e-07

Table 207: Bland Altman Statistics

Sector	Mean_Diff	SD	upperLOA	lower_CI	upper_CI	lowerLOA	lower_CI_	upper_CI_
Stroma_I_5-7mm	5.27	5.587	16.2204	13.1411	19.2997	-5.6804	-8.7597	-2.6011

Table 208: Basic Statistics

Mean_Avanti	SD1	Min1	Max1	Mean_REVO	SD2	Min2	Max2
535.7497	25.6287	485.54	591.83	530.4798	26.2291	480.33	591.5

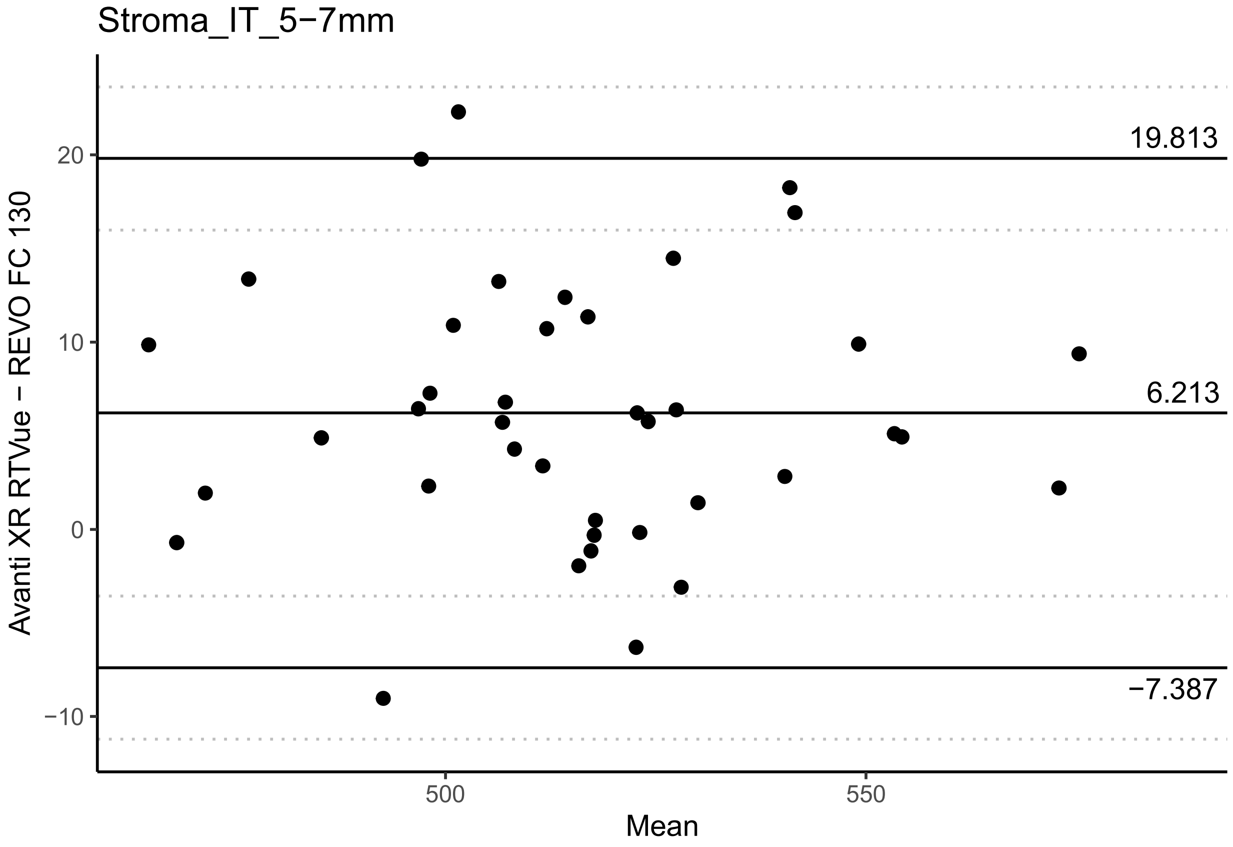

Table 209: Shapiro-Wilk Test Results

Test	W_statistic	p_value
Avanti RTVue	0.9714137	0.3985462
REVO FC 130	0.9825654	0.7833244
Differences	0.9890045	0.9602458

Table 210: Paired T-test results

	Test	t_statistic	p_value
t	Paired t-test for mean differences	5.663168	1.5e-06

Table 211: Bland Altman Statistics

Sector	Mean_Diff	SD	upperLOA	lower_CI	upper_CI	lowerLOA	lower_CI_	upper_CI_
Stroma_IT_5-7mm	6.2132	6.9389	19.8135	15.989	23.6379	-7.387	-11.2114	-3.5625

Table 212: Basic Statistics

Mean_Avanti	SD1	Min1	Max1	Mean_REVO	SD2	Min2	Max2
519.0128	25.8597	467.71	580.01	512.7995	25.7513	459.8	571.82

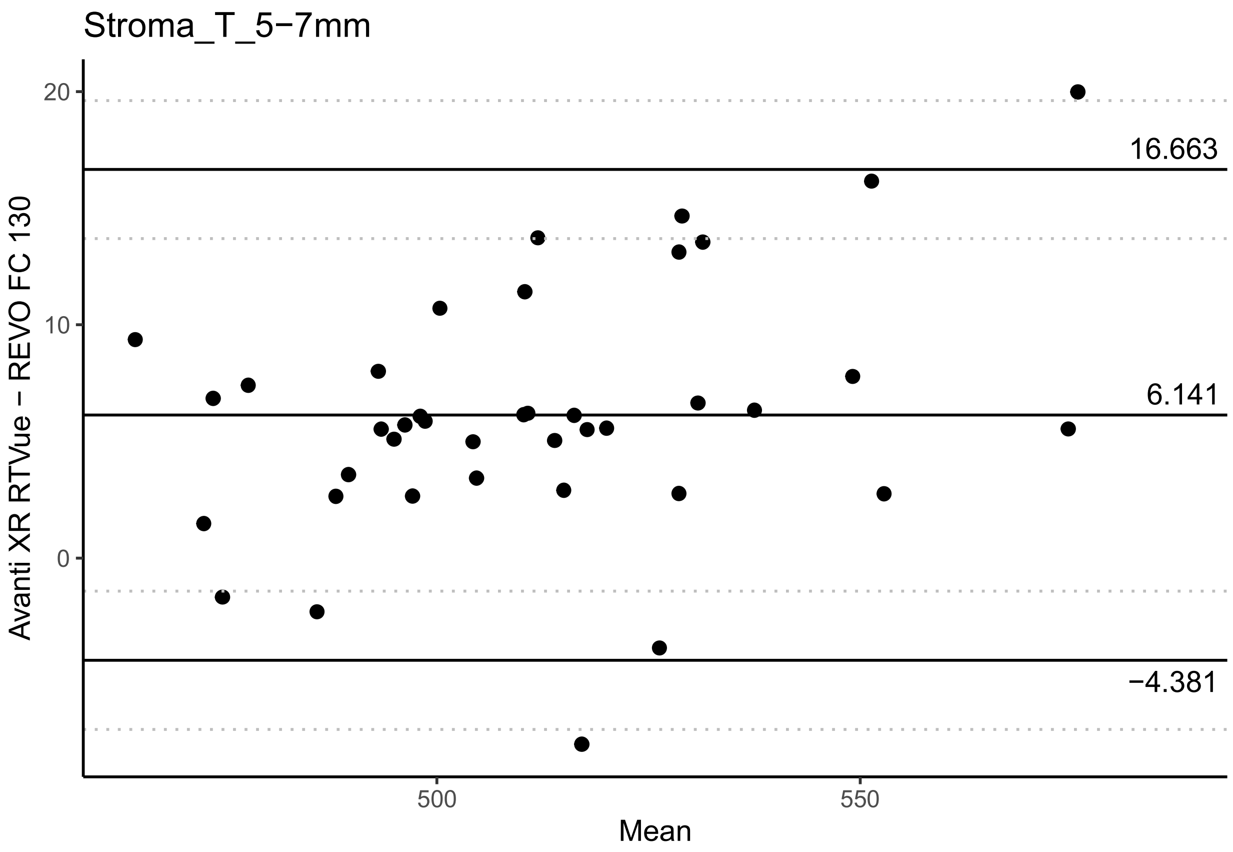

Table 213: Shapiro-Wilk Test Results

Test	W_statistic	p_value
Avanti RTVue	0.9673663	0.2960561
REVO FC 130	0.9768559	0.5742958
Differences	0.9545422	0.1087378

Table 214: Paired T-test results

	Test	t_statistic	p_value
t	Paired t-test for mean differences	7.234657	0

Table 215: Bland Altman Statistics

Sector	Mean_Diff	SD	upperLOA	lower_CI	upper_CI	lowerLOA	lower_CI_	upper_CI_
Stroma_T_5-7mm	6.1407	5.3683	16.6625	13.7038	19.6213	-4.381	-7.3398	-1.4223

Table 216: Basic Statistics

Mean_Avanti	SD1	Min1	Max1	Mean_REVO	SD2	Min2	Max2
514.718	27.3126	469.04	585.72	508.5772	25.4394	459.67	571.82

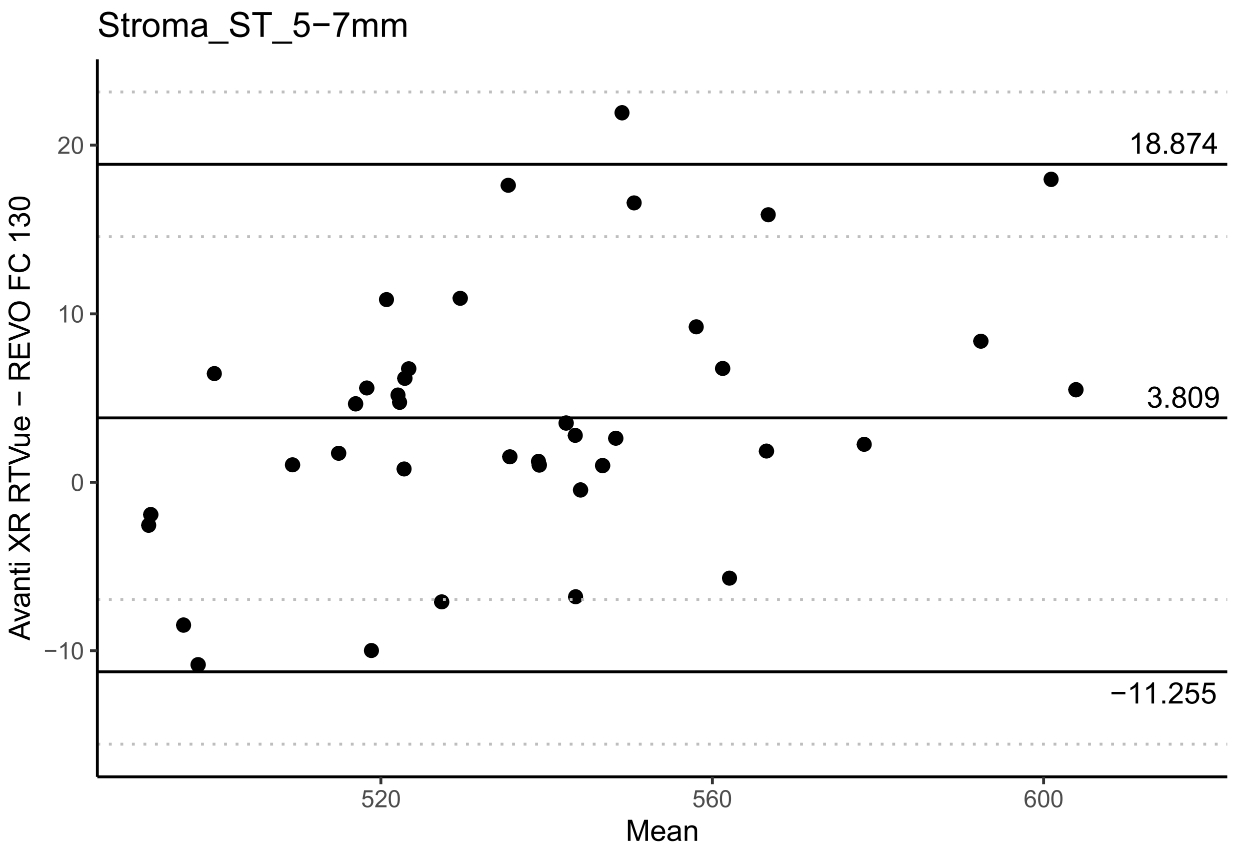

Table 217: Shapiro-Wilk Test Results

Test	W_statistic	p_value
Avanti RTVue	0.9604802	0.1739475
REVO FC 130	0.9606870	0.1879905
Differences	0.9660336	0.2815776

Table 218: Paired T-test results

	Test	t_statistic	p_value
t	Paired t-test for mean differences	3.095115	0.0036834

Table 219: Bland Altman Statistics

Sector	Mean_Diff	SD	upperLOA	lower_CI	upper_CI	lowerLOA	lower_CI_	upper_CI_
Stroma_ST_5-7mm	3.8092	7.6859	18.8735	14.5789	23.1682	-11.2551	-15.5497	-6.9604

Table 220: Basic Statistics

Mean_Avanti	SD1	Min1	Max1	Mean_REVO	SD2	Min2	Max2
539.2108	29.8328	490.68	609.9	535.4015	26.6813	493.17	601.16

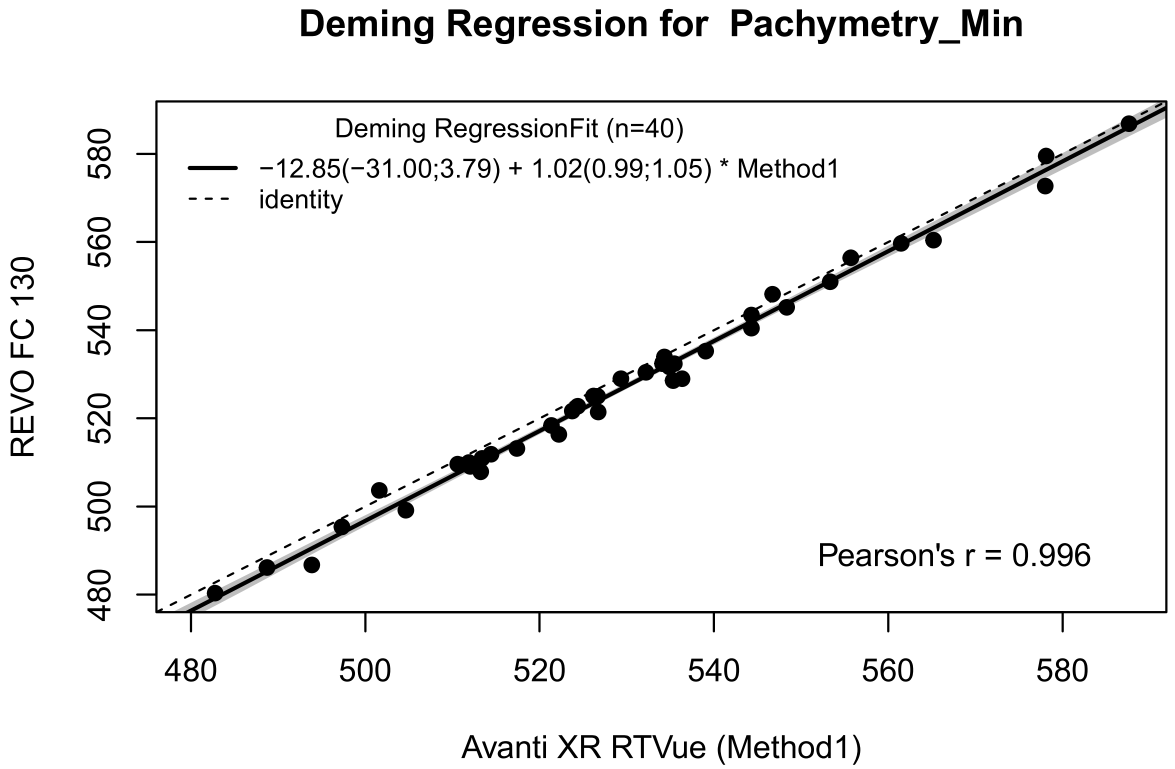

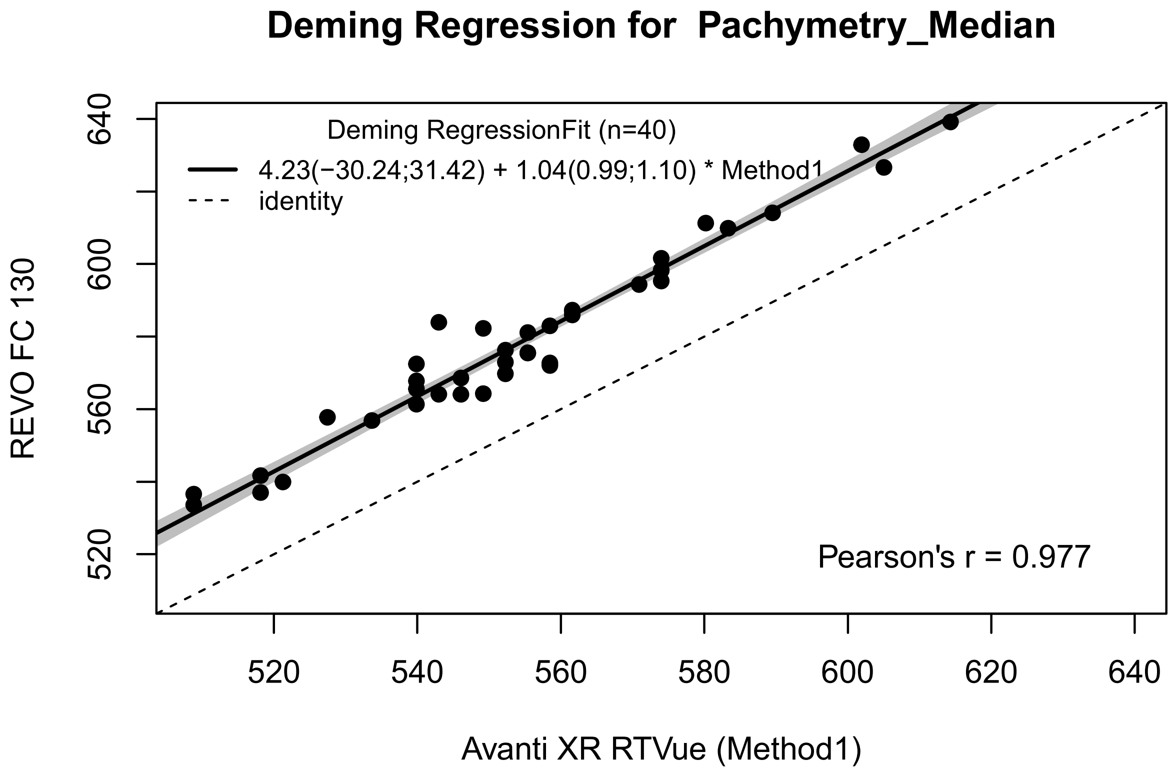
Deming Regression for Pachymetry_Median
Deming RegressionFit (n=40)
4.23(−30.24;31.42) + 1.04(0.99;1.10) * Method1
identity
Pearson's r = 0.977
REVO FC 130
520
560
600
640
520
540
560
580
600
620
640
Avanti XR RTVue (Method1)

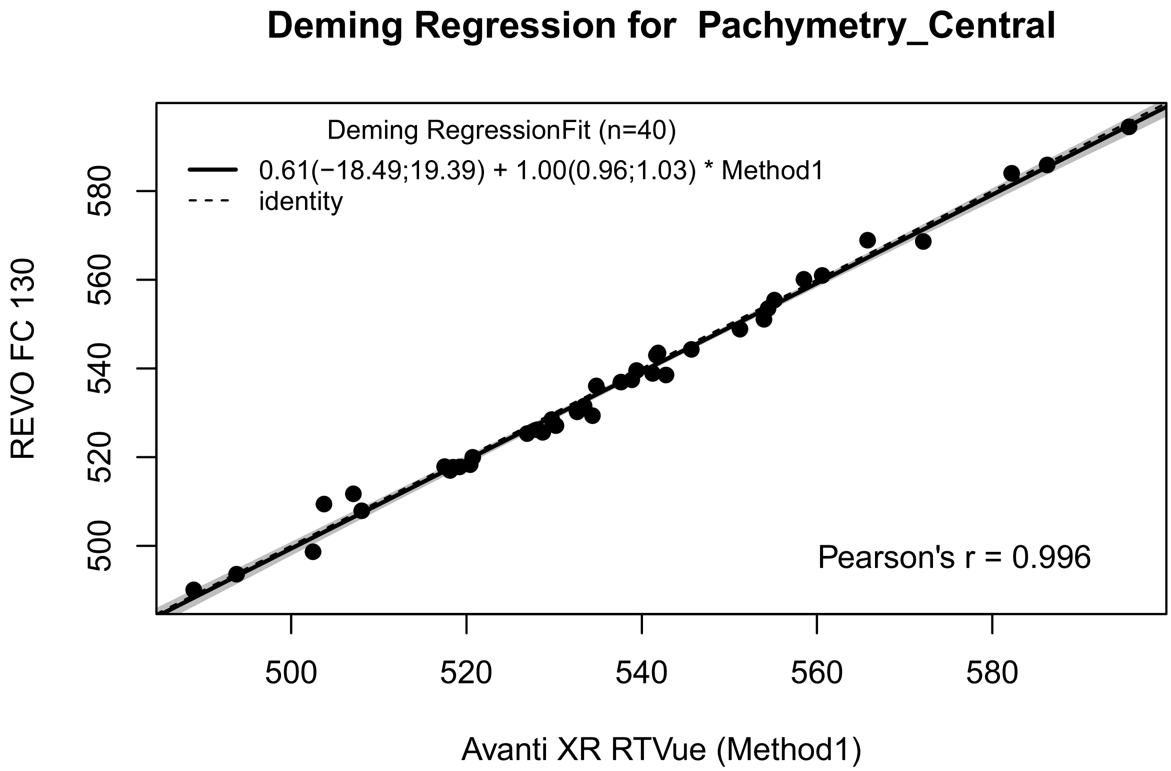
Deming Regression for Pachymetry_Central
Deming RegressionFit (n=40)
0.61(−18.49;19.39) + 1.00(0.96;1.03) * Method1
identity
Pearson's r = 0.996
REVO FC 130
Avanti XR RTVue (Method1)
500
520
540
560
580

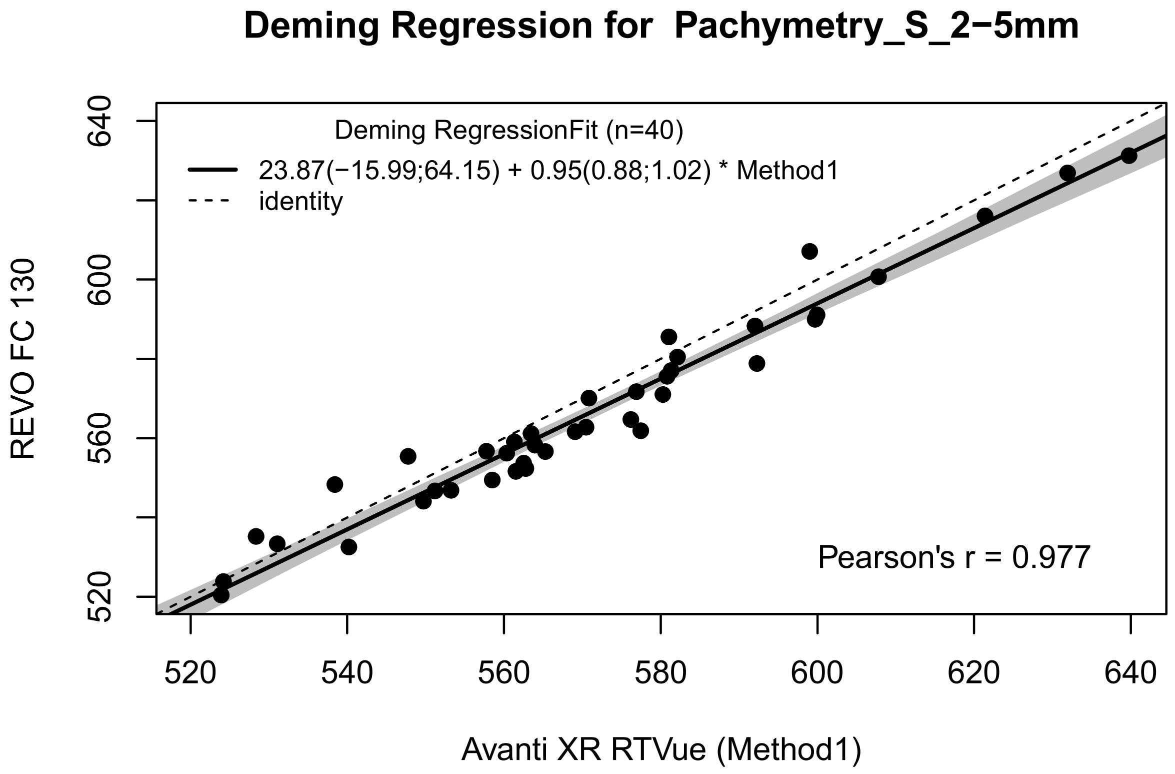

Deming Regression for Pachymetry_S_2−5mm
Deming RegressionFit (n=40)
23.87(−15.99;64.15) + 0.95(0.88;1.02) * Method1
identity
Pearson's r = 0.977
REVO FC 130
Avanti XR RTVue (Method1)
520
560
600
640
520
540
560
580
600
620
640

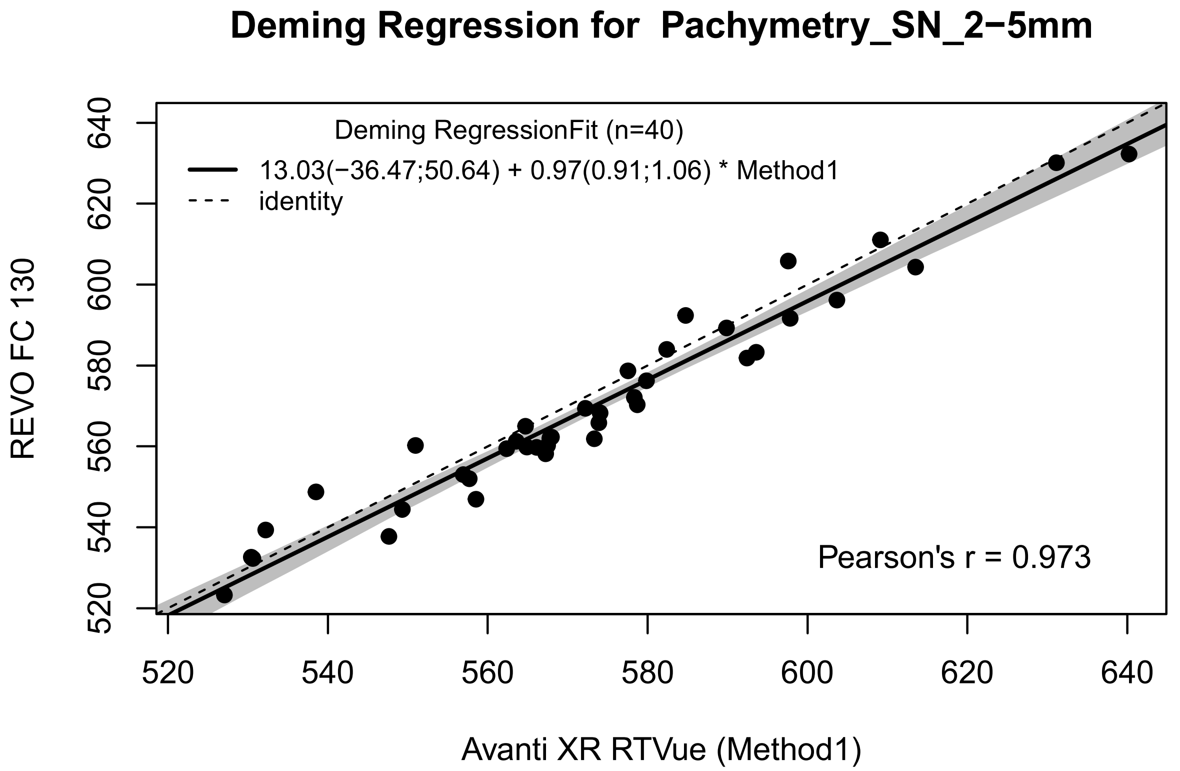
Deming Regression for Pachymetry_SN_2−5mm
Deming RegressionFit (n=40)
13.03(−36.47;50.64) + 0.97(0.91;1.06) * Method1
identity
Pearson's r = 0.973
REVO FC 130
Avanti XR RTVue (Method1)
520
540
560
580
600
620
640

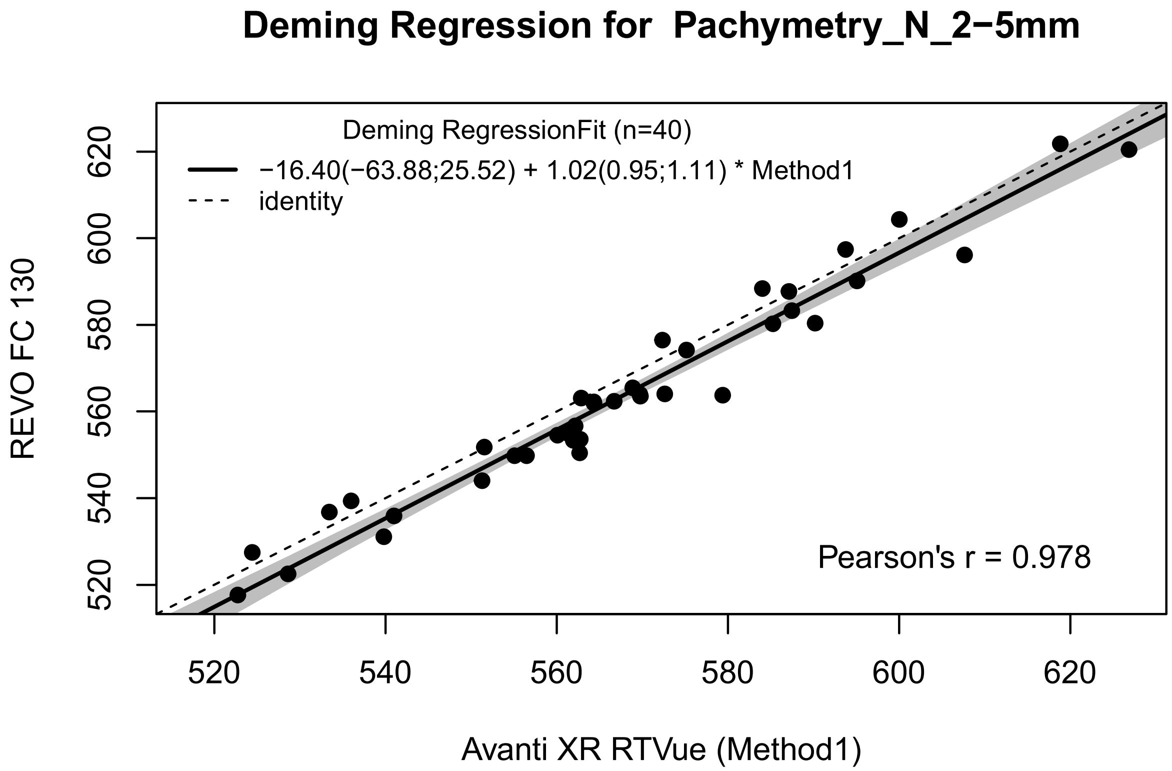
Deming Regression for Pachymetry_N_2−5mm
Deming RegressionFit (n=40)
−16.40(−63.88;25.52) + 1.02(0.95;1.11) * Method1
identity
Pearson's r = 0.978
REVO FC 130
520
540
560
580
600
620
Avanti XR RTVue (Method1)

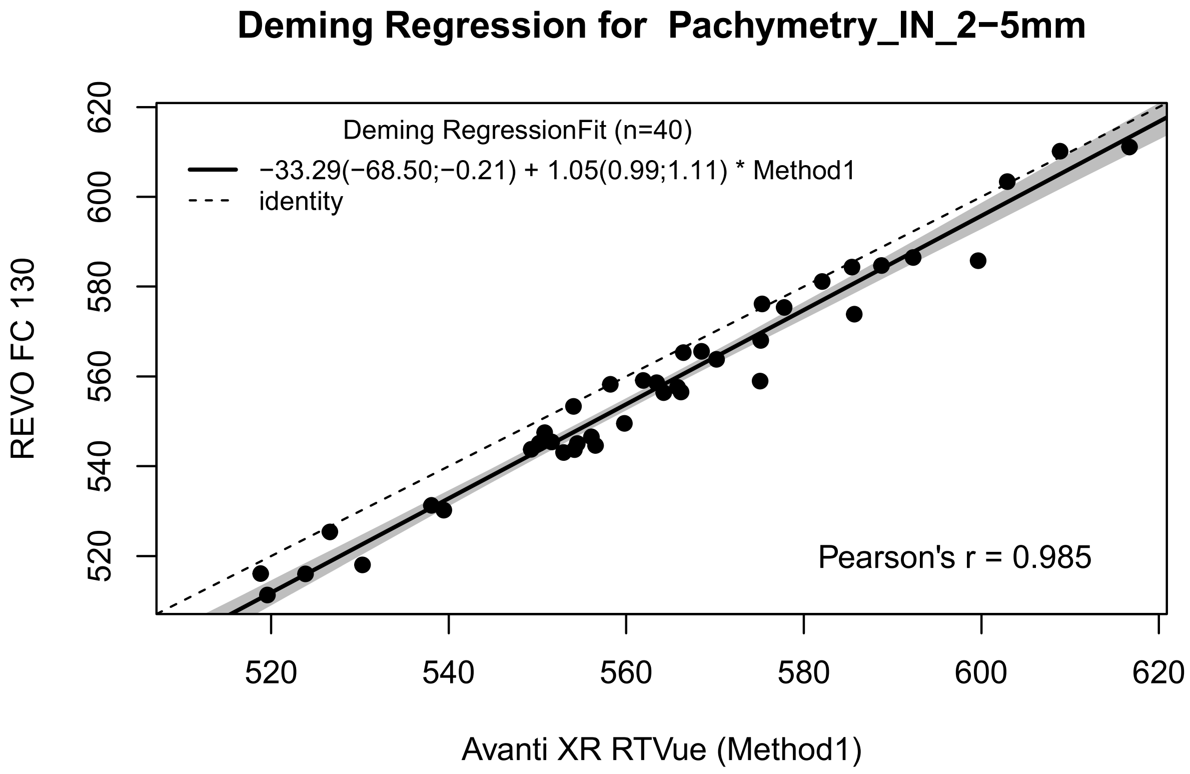

Deming Regression for Pachymetry_IN_2−5mm
Deming RegressionFit (n=40)
−33.29(−68.50;−0.21) + 1.05(0.99;1.11) * Method1
identity
Pearson's r = 0.985
REVO FC 130
Avanti XR RTVue (Method1)
520
540
560
580
600
620

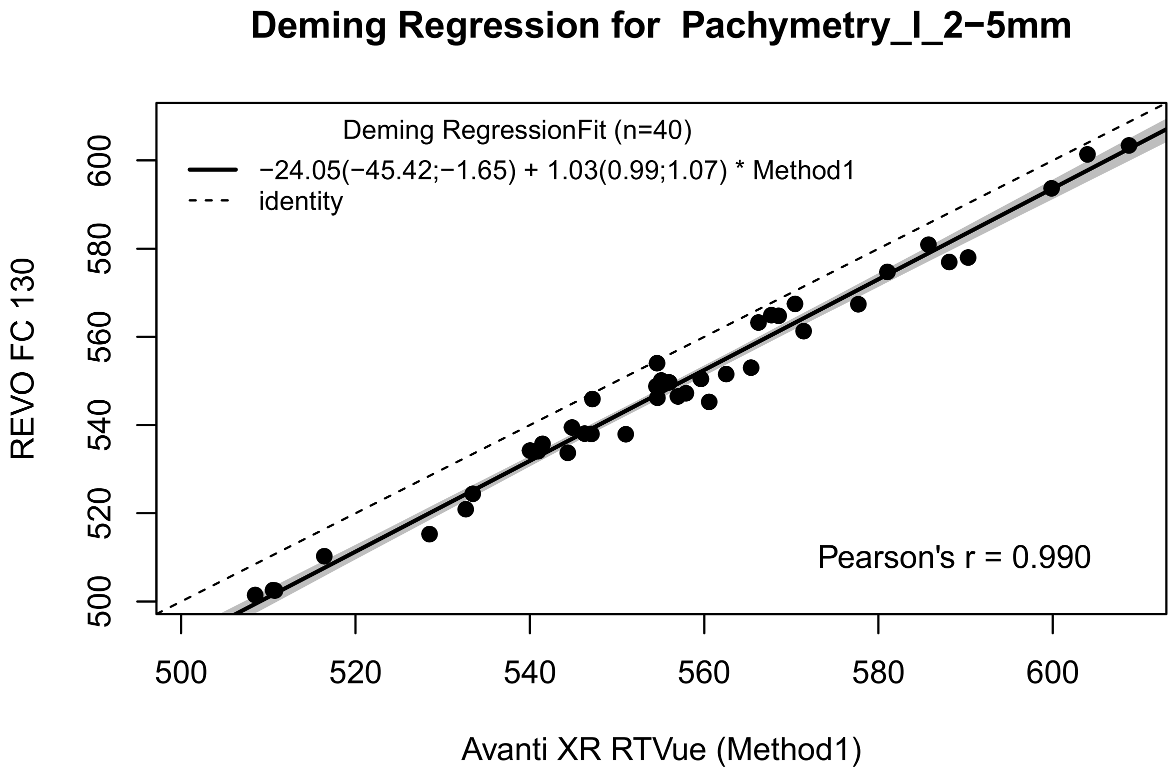
Deming Regression for Pachymetry_I_2−5mm
Deming RegressionFit (n=40)
−24.05(−45.42;−1.65) + 1.03(0.99;1.07) * Method1
identity
Pearson's r = 0.990
REVO FC 130
Avanti XR RTVue (Method1)
500
520
540
560
580
600

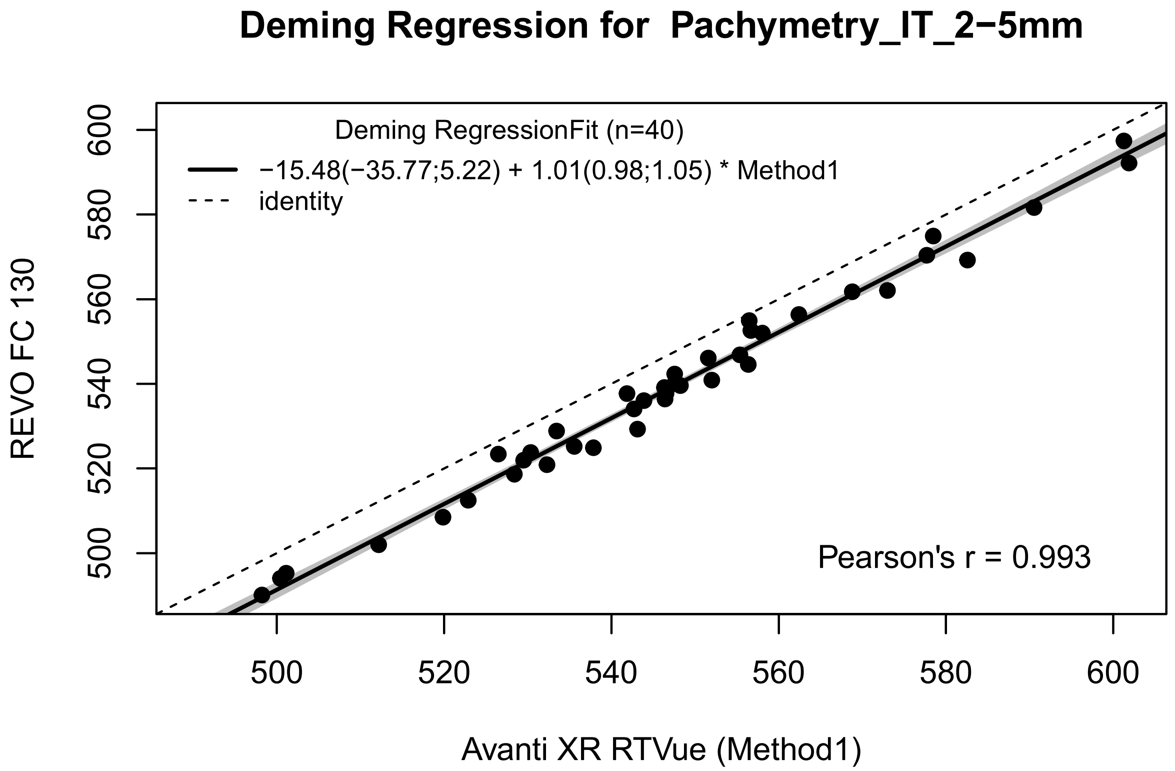

Deming Regression for Pachymetry_IT_2−5mm
Deming RegressionFit (n=40)
−15.48(−35.77;5.22) + 1.01(0.98;1.05) * Method1
identity
Pearson's r = 0.993
REVO FC 130
Avanti XR RTVue (Method1)
500
520
540
560
580
600

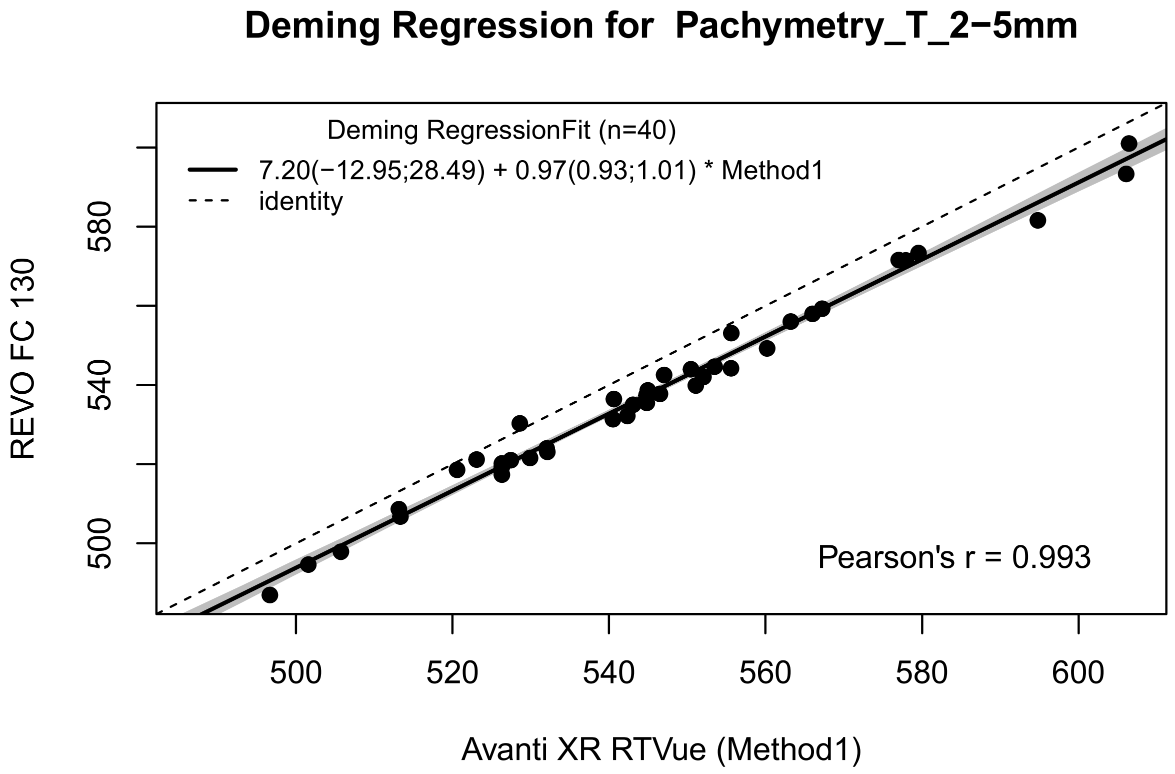
Deming Regression for Pachymetry_T_2−5mm
Deming RegressionFit (n=40)
7.20(−12.95;28.49) + 0.97(0.93;1.01) * Method1
identity
Pearson's r = 0.993
REVO FC 130
Avanti XR RTVue (Method1)
500
540
580
500
520
540
560
580
600

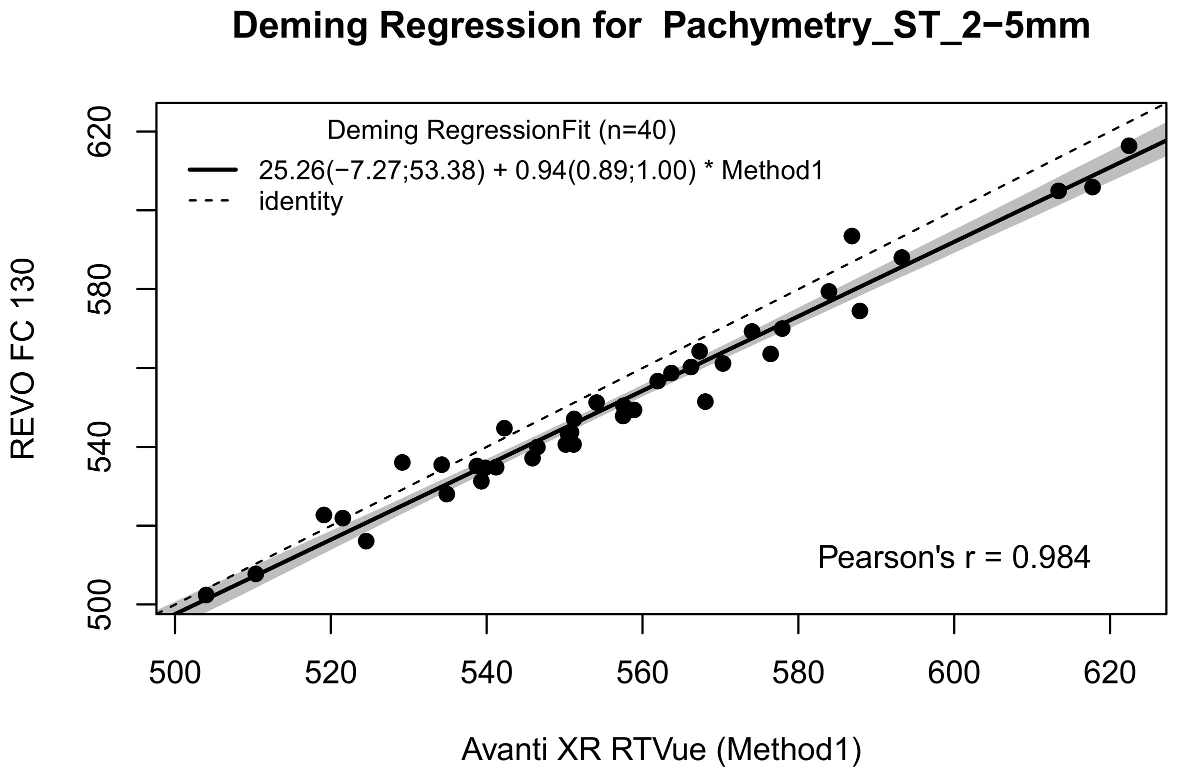
Deming Regression for Pachymetry_ST_2−5mm
Deming RegressionFit (n=40)
25.26(−7.27;53.38) + 0.94(0.89;1.00) * Method1
identity
Pearson's r = 0.984
REVO FC 130
620
580
540
500
500
520
540
560
580
600
620
Avanti XR RTVue (Method1)

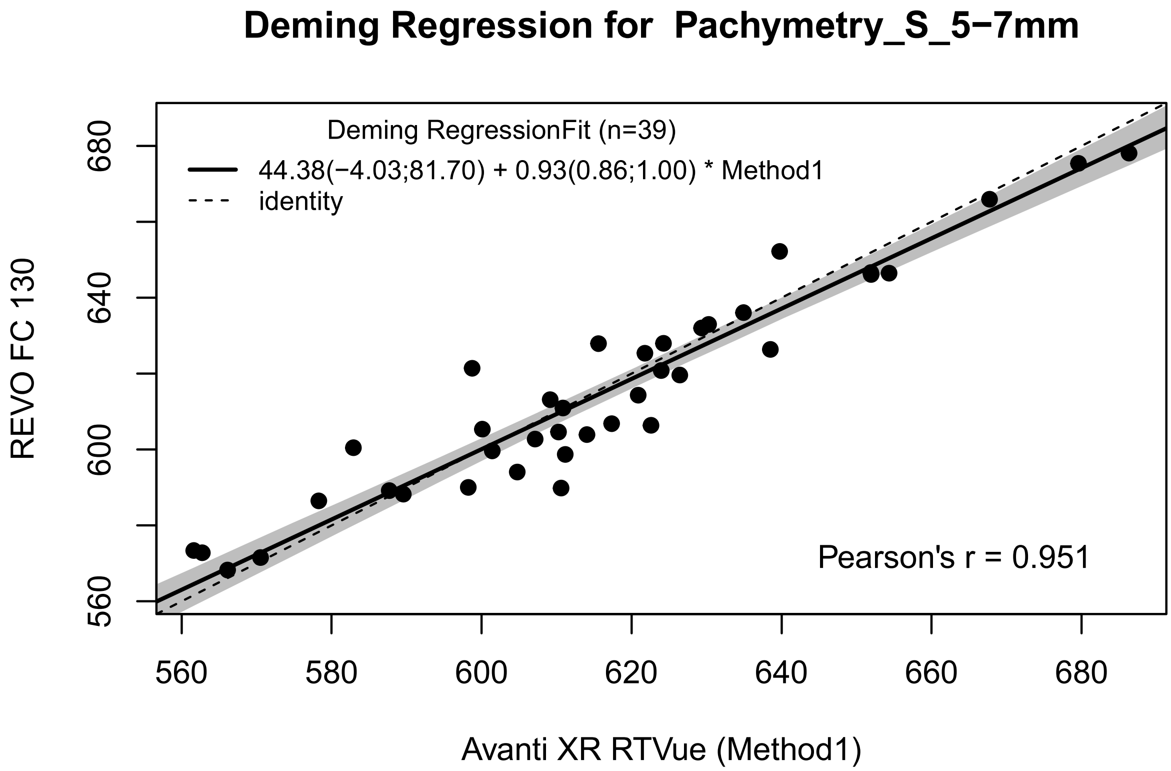

Deming Regression for Pachymetry_S_5−7mm
Deming RegressionFit (n=39)
44.38(−4.03;81.70) + 0.93(0.86;1.00) * Method1
identity
Pearson's r = 0.951
REVO FC 130
Avanti XR RTVue (Method1)
560
580
600
620
640
660
680

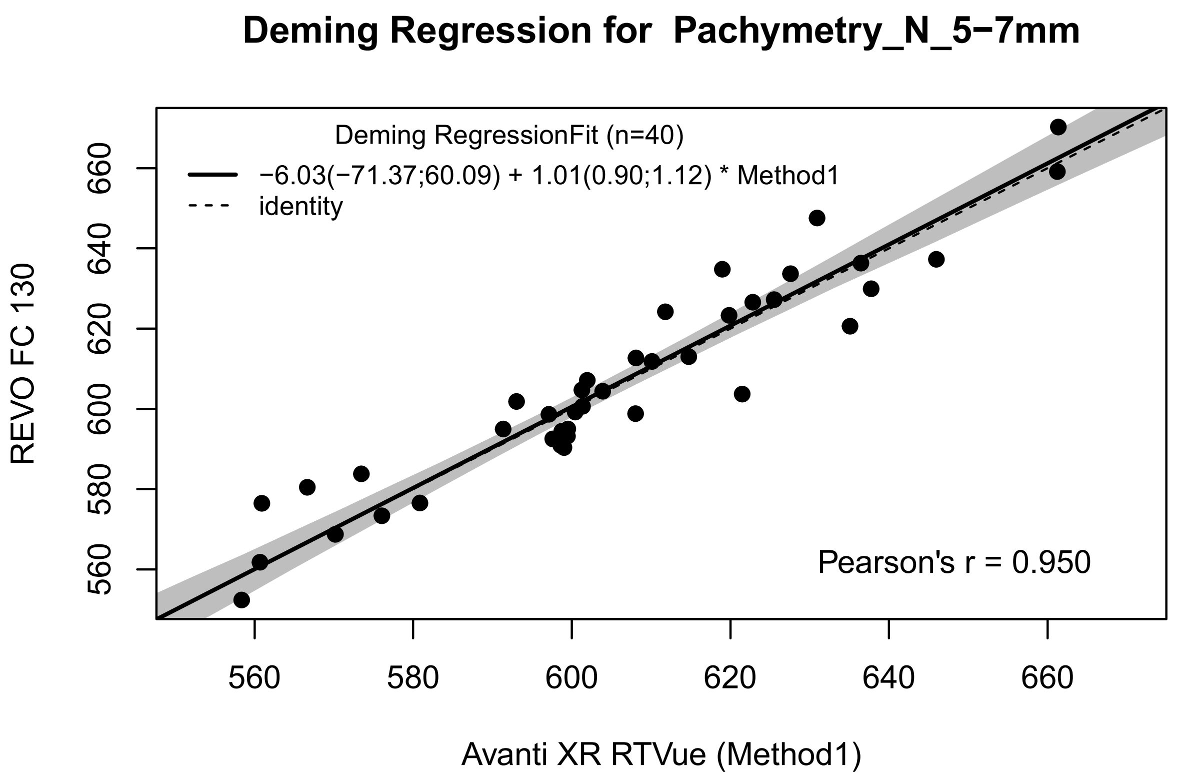
Deming Regression for Pachymetry_N_5−7mm
Deming RegressionFit (n=40)
−6.03(−71.37;60.09) + 1.01(0.90;1.12) * Method1
identity
Pearson's r = 0.950
REVO FC 130
Avanti XR RTVue (Method1)
560
580
600
620
640
660

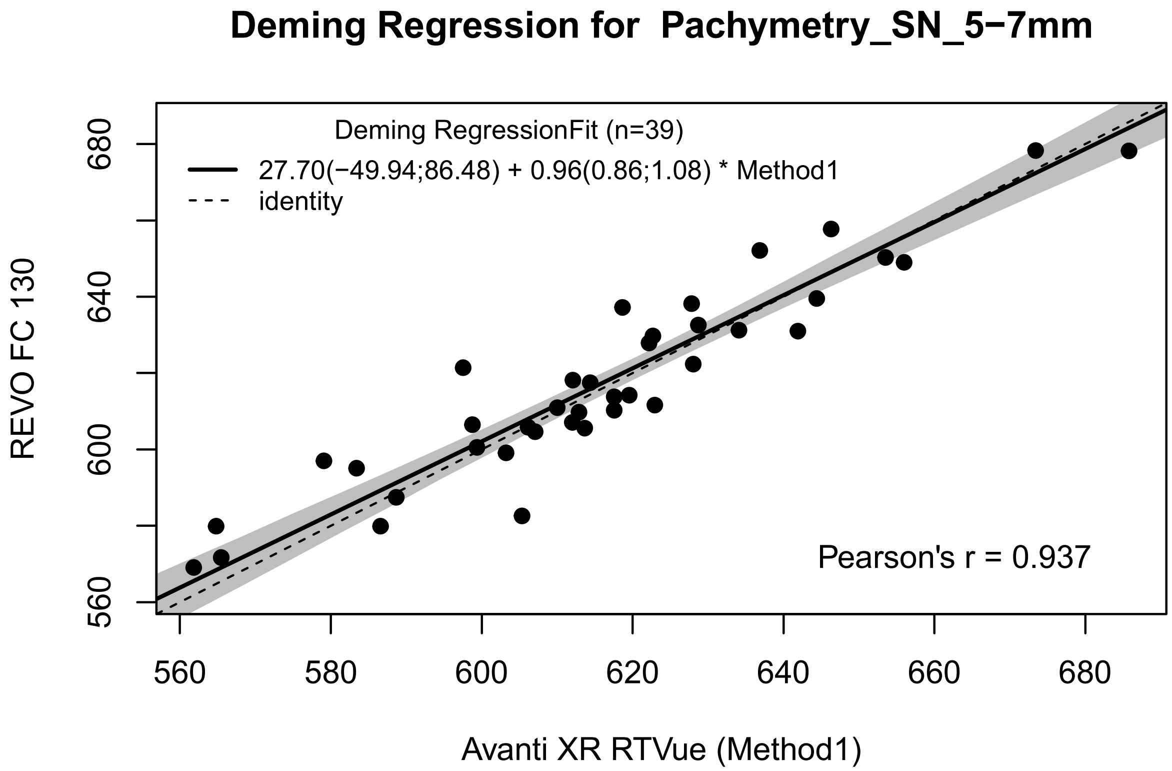
Deming Regression for Pachymetry_SN_5−7mm
Deming RegressionFit (n=39)
27.70(−49.94;86.48) + 0.96(0.86;1.08) * Method1
identity
Pearson's r = 0.937
REVO FC 130
Avanti XR RTVue (Method1)
560
600
640
680
560
580
600
620
640
660
680

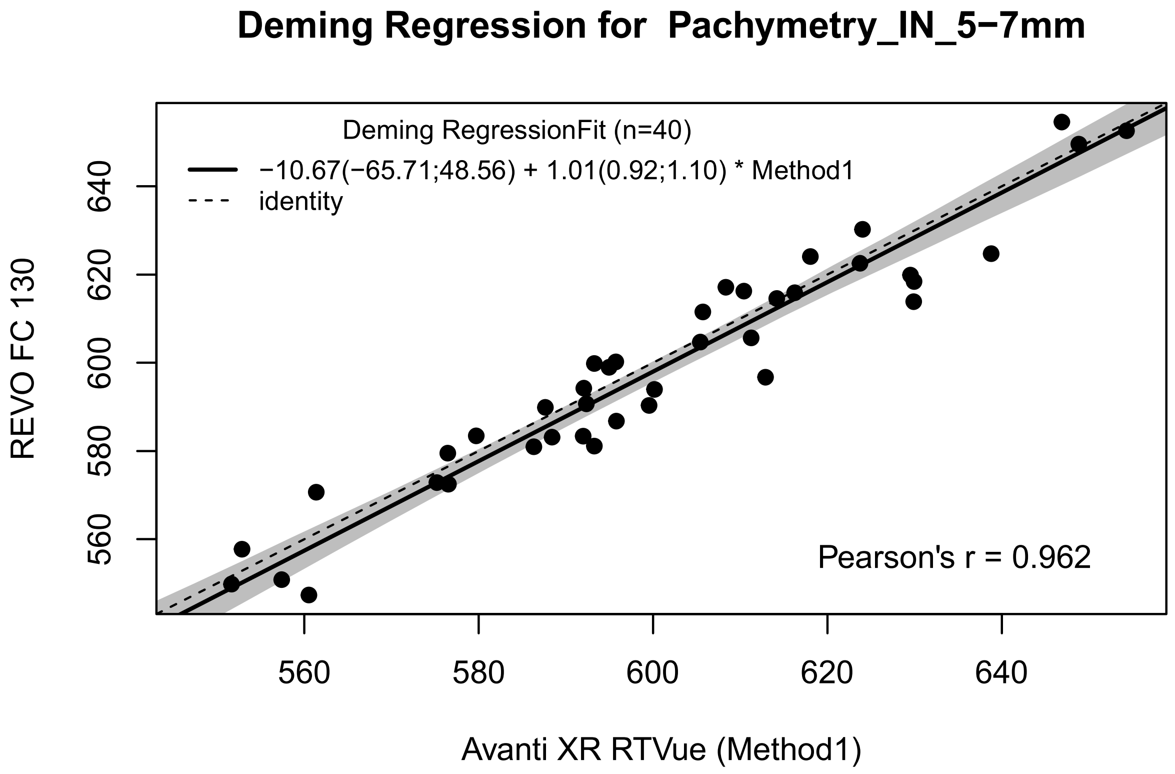
Deming Regression for Pachymetry_IN_5−7mm
Deming RegressionFit (n=40)
−10.67(−65.71;48.56) + 1.01(0.92;1.10) * Method1
identity
Pearson's r = 0.962
REVO FC 130
Avanti XR RTVue (Method1)
560
580
600
620
640
560
580
600
620
640

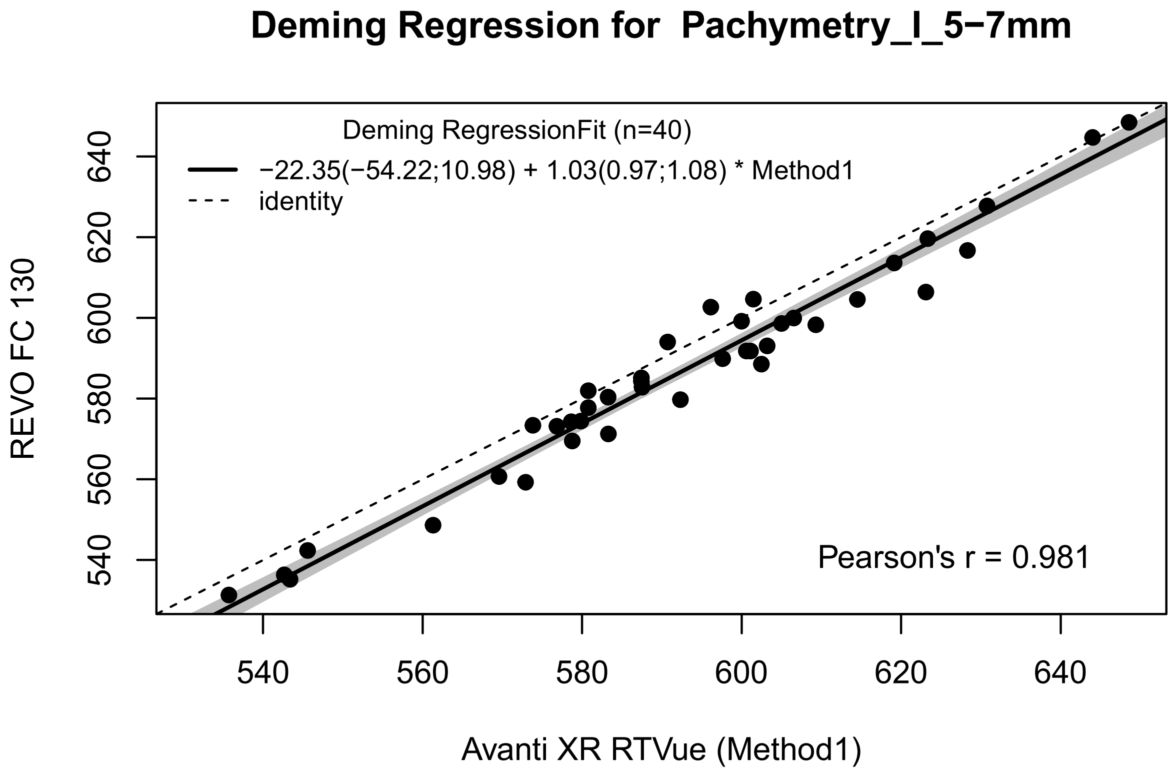

Deming Regression for Pachymetry_I_5−7mm
Deming RegressionFit (n=40)
−22.35(−54.22;10.98) + 1.03(0.97;1.08) * Method1
identity
Pearson's r = 0.981
REVO FC 130
Avanti XR RTVue (Method1)
540
560
580
600
620
640

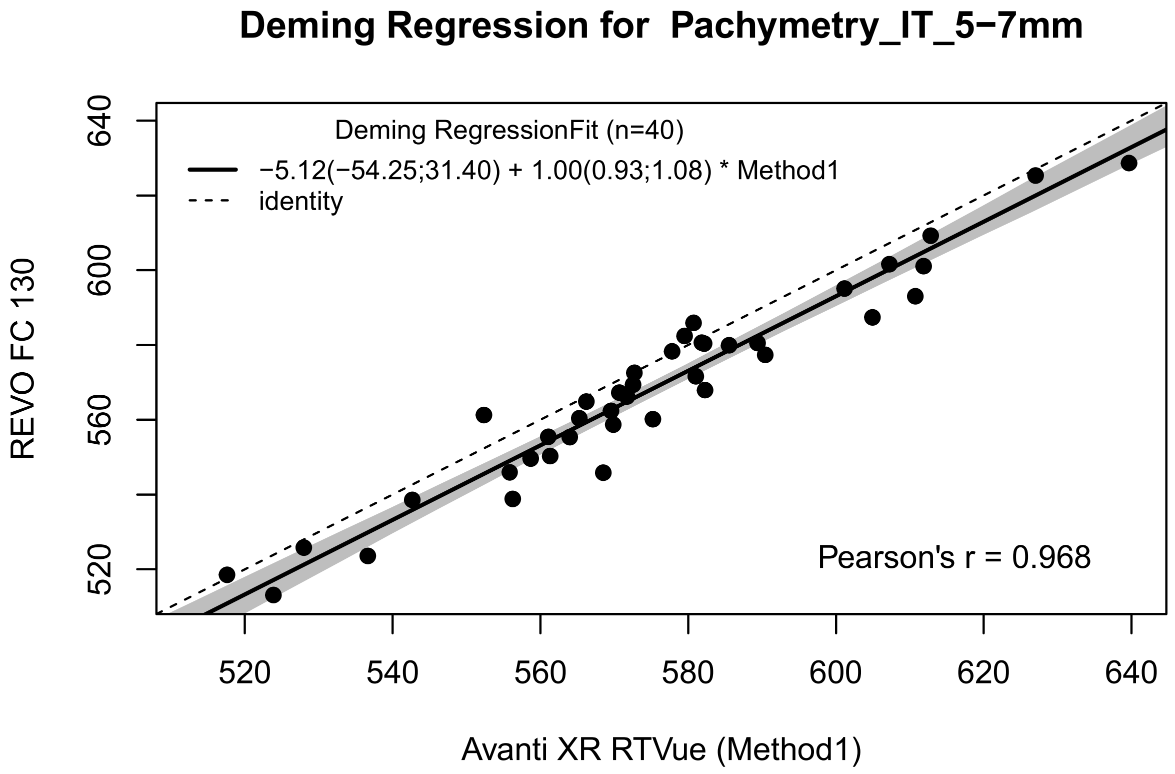

Deming Regression for Pachymetry_IT_5−7mm
Deming RegressionFit (n=40)
−5.12(−54.25;31.40) + 1.00(0.93;1.08) * Method1
identity
Pearson's r = 0.968
REVO FC 130
Avanti XR RTVue (Method1)
520
540
560
580
600
620
640

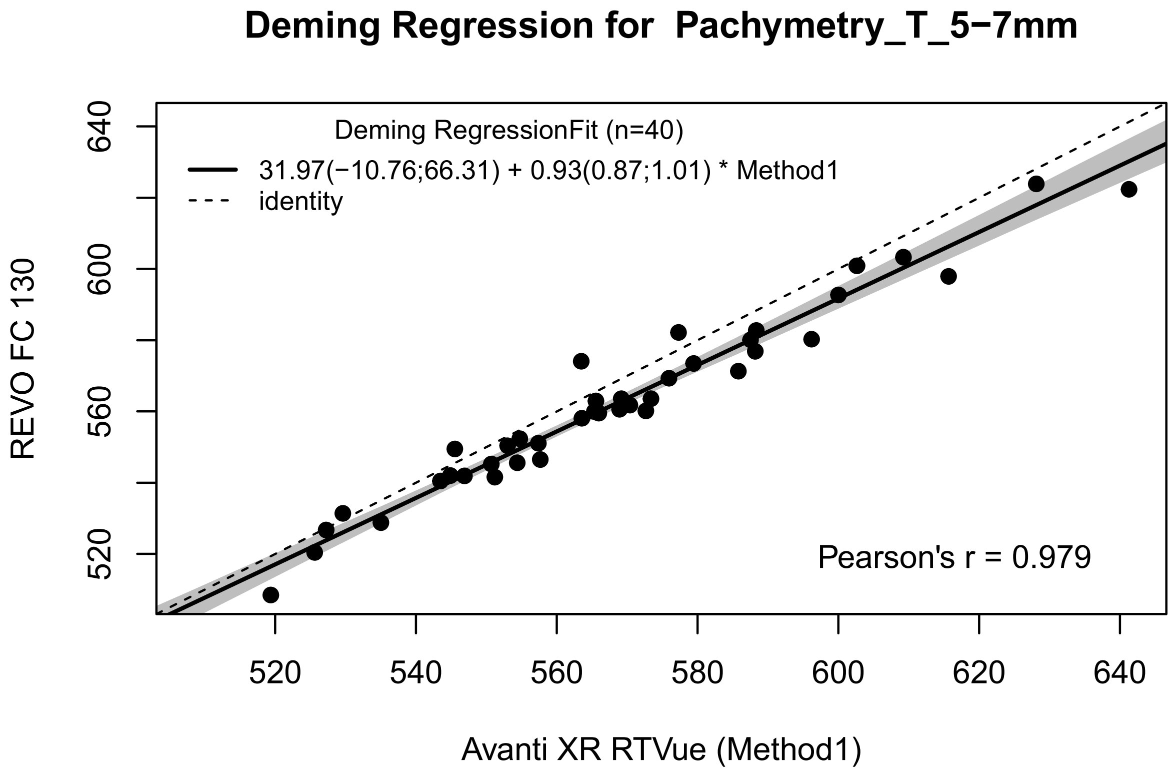
Deming Regression for Pachymetry_T_5−7mm
Deming RegressionFit (n=40)
31.97(−10.76;66.31) + 0.93(0.87;1.01) * Method1
identity
Pearson's r = 0.979
REVO FC 130
Avanti XR RTVue (Method1)
520
540
560
580
600
620
640

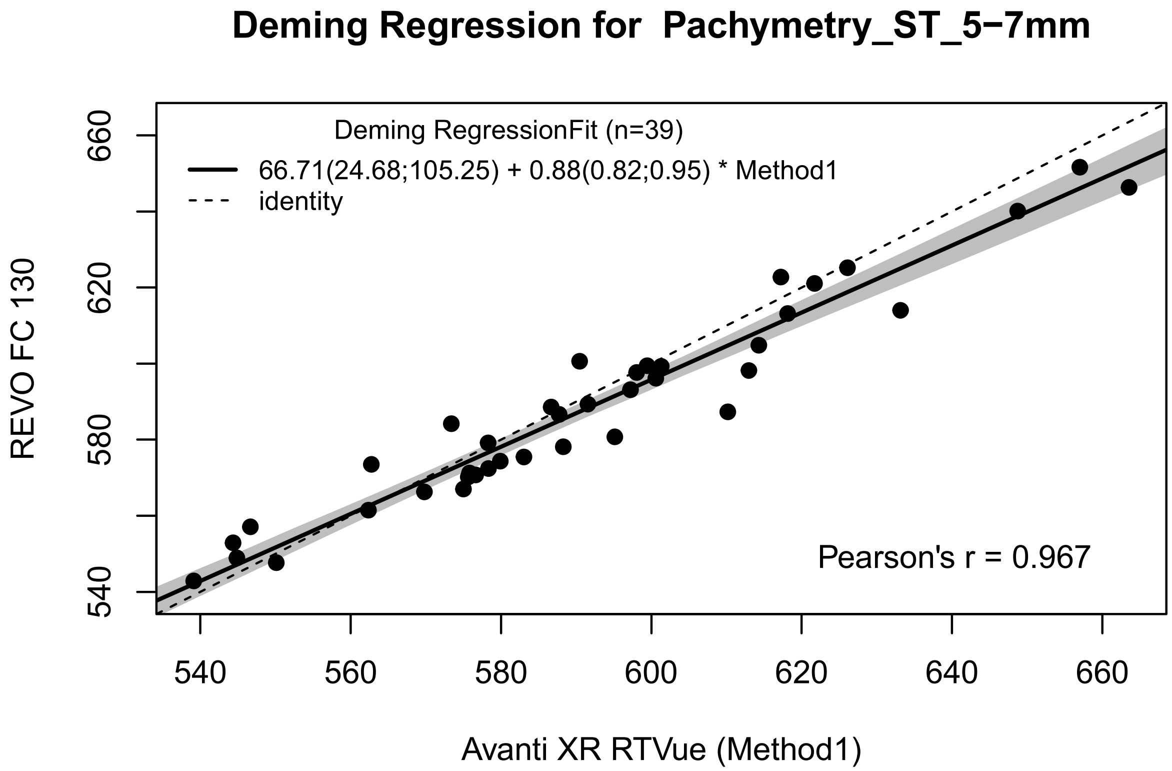
Deming Regression for Pachymetry_ST_5−7mm
Deming RegressionFit (n=39)
66.71(24.68;105.25) + 0.88(0.82;0.95) * Method1
identity
Pearson's r = 0.967
REVO FC 130
Avanti XR RTVue (Method1)
540
560
580
600
620
640
660

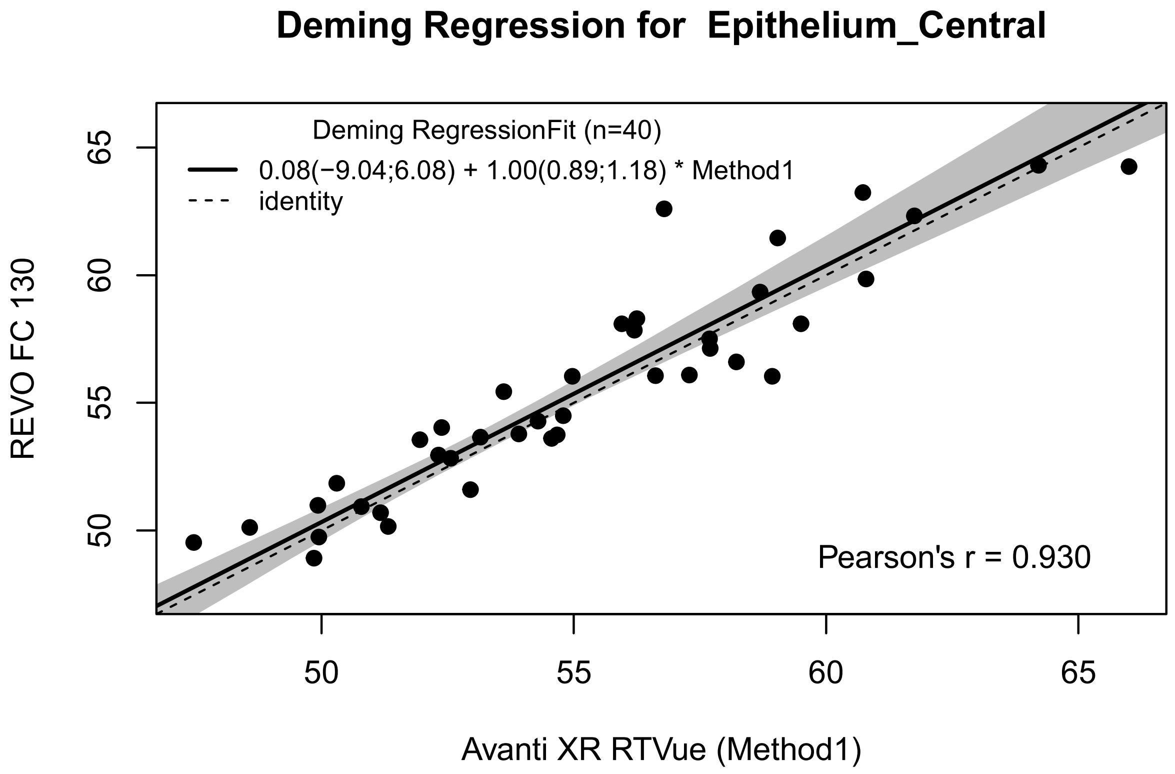

Deming Regression for Epithelium_Central
Deming RegressionFit (n=40)
0.08(−9.04;6.08) + 1.00(0.89;1.18) * Method1
identity
Pearson's r = 0.930
REVO FC 130
Avanti XR RTVue (Method1)
50
55
60
65
50
55
60
65

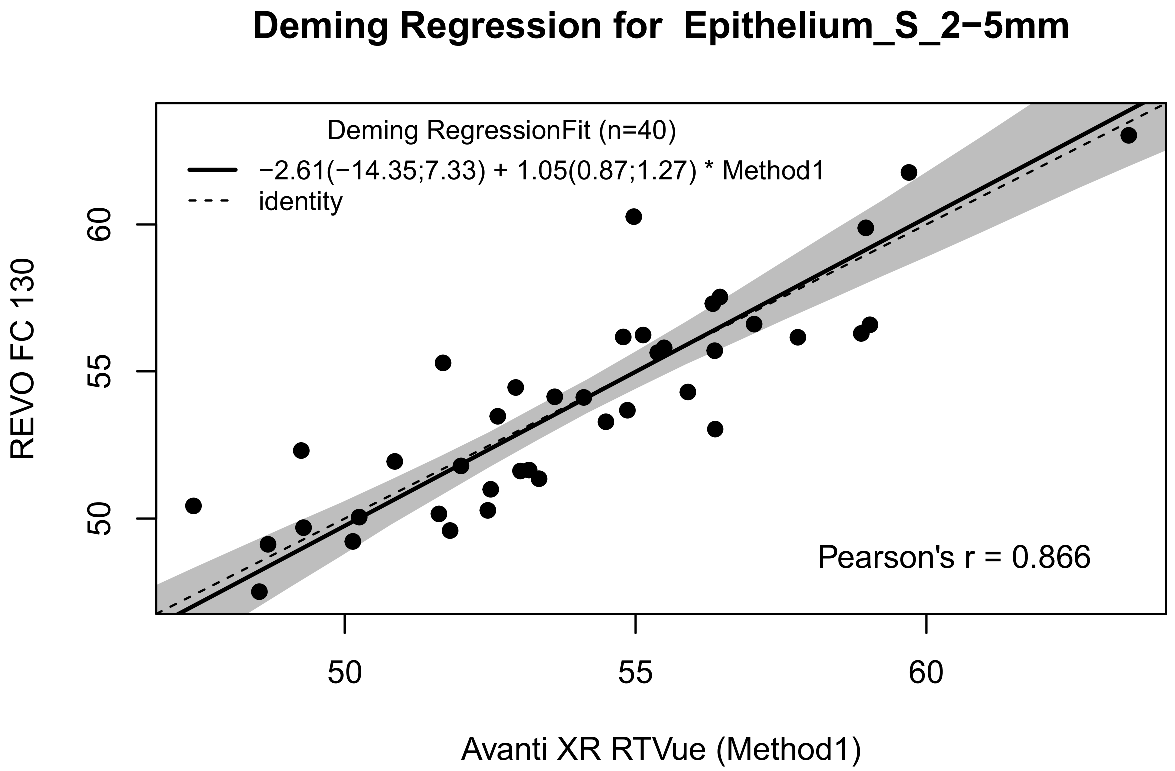

Deming Regression for Epithelium_S_2−5mm
Deming RegressionFit (n=40)
−2.61(−14.35;7.33) + 1.05(0.87;1.27) * Method1
identity
Pearson's r = 0.866
REVO FC 130
Avanti XR RTVue (Method1)
50
55
60
50
55
60

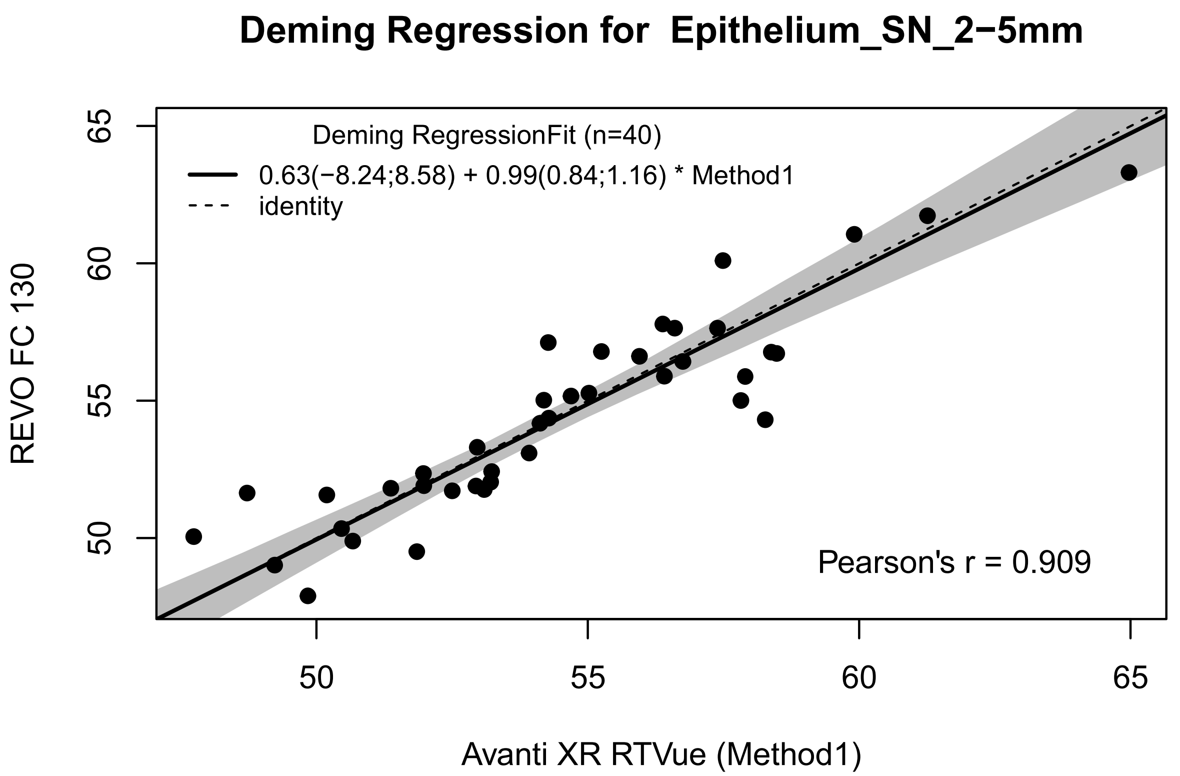

Deming Regression for Epithelium_SN_2−5mm
Deming RegressionFit (n=40)
0.63(−8.24;8.58) + 0.99(0.84;1.16) * Method1
identity
Pearson's r = 0.909
REVO FC 130
Avanti XR RTVue (Method1)
50
55
60
65
50
55
60
65

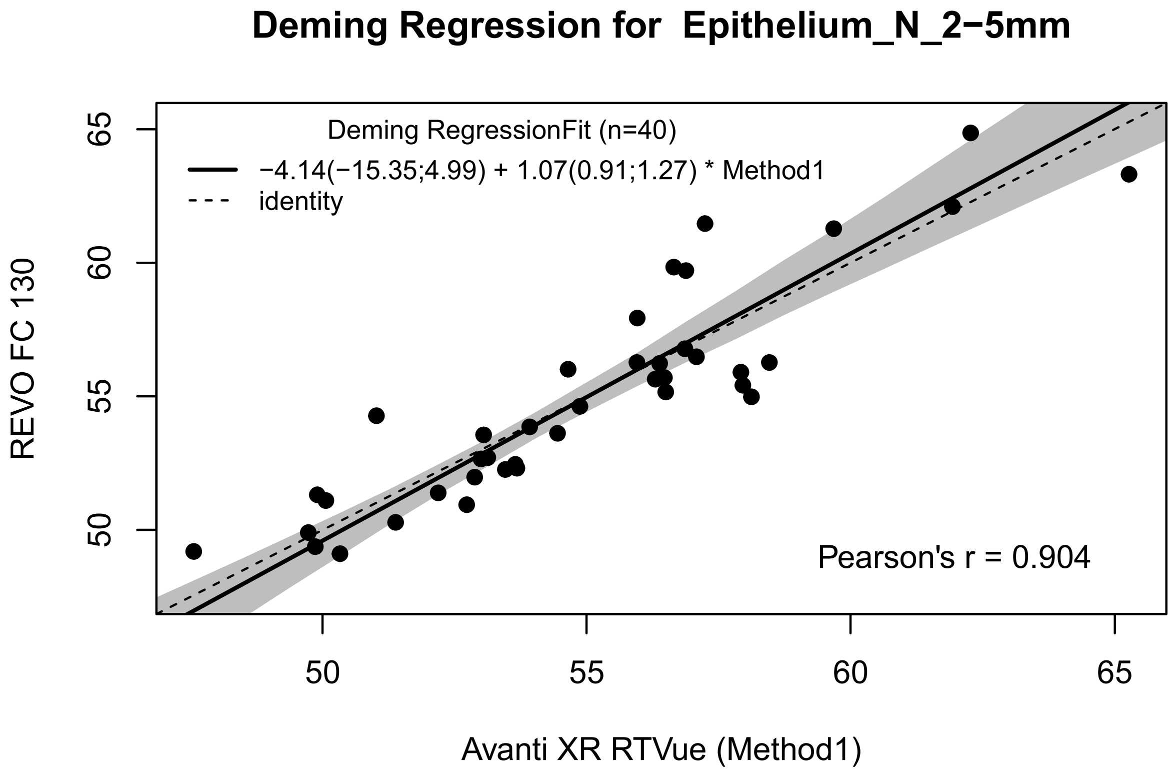

Deming Regression for Epithelium_N_2−5mm
Deming RegressionFit (n=40)
−4.14(−15.35;4.99) + 1.07(0.91;1.27) * Method1
identity
Pearson's r = 0.904
REVO FC 130
Avanti XR RTVue (Method1)
50
55
60
65
50
55
60
65

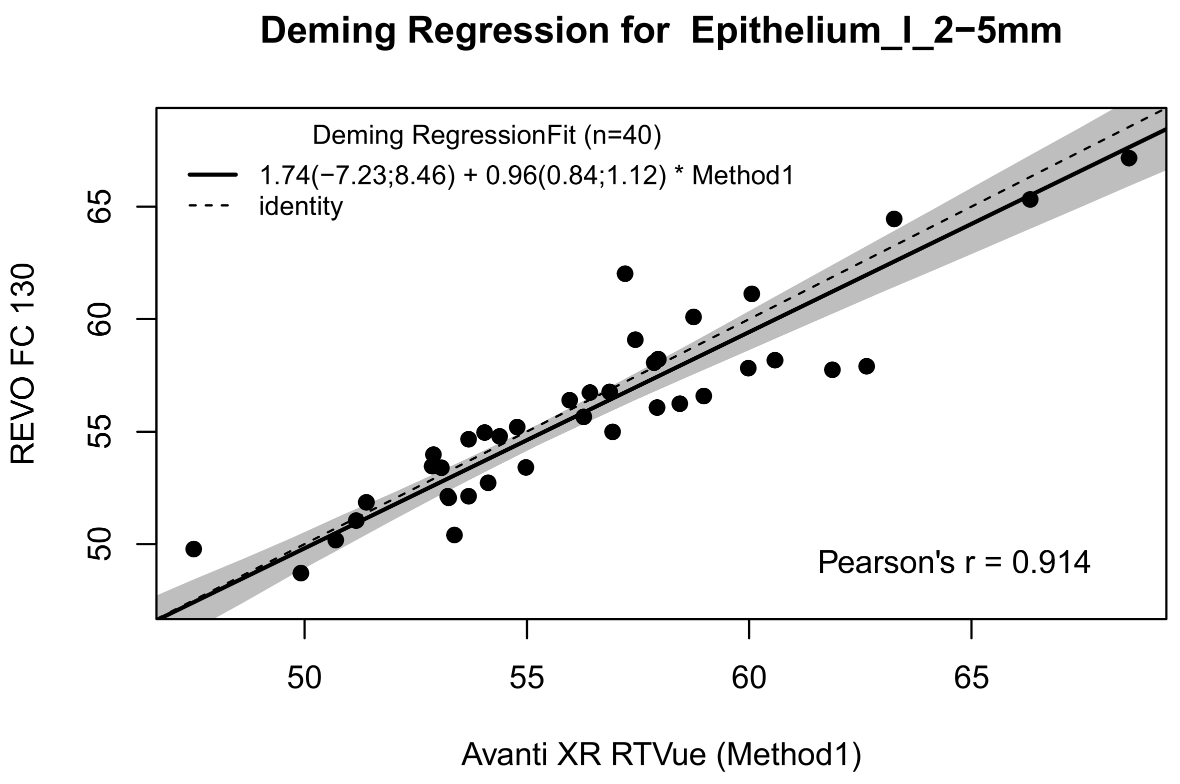

Deming Regression for Epithelium_I_2−5mm
Deming RegressionFit (n=40)
1.74(−7.23;8.46) + 0.96(0.84;1.12) * Method1
identity
Pearson's r = 0.914
REVO FC 130
Avanti XR RTVue (Method1)
50
55
60
65

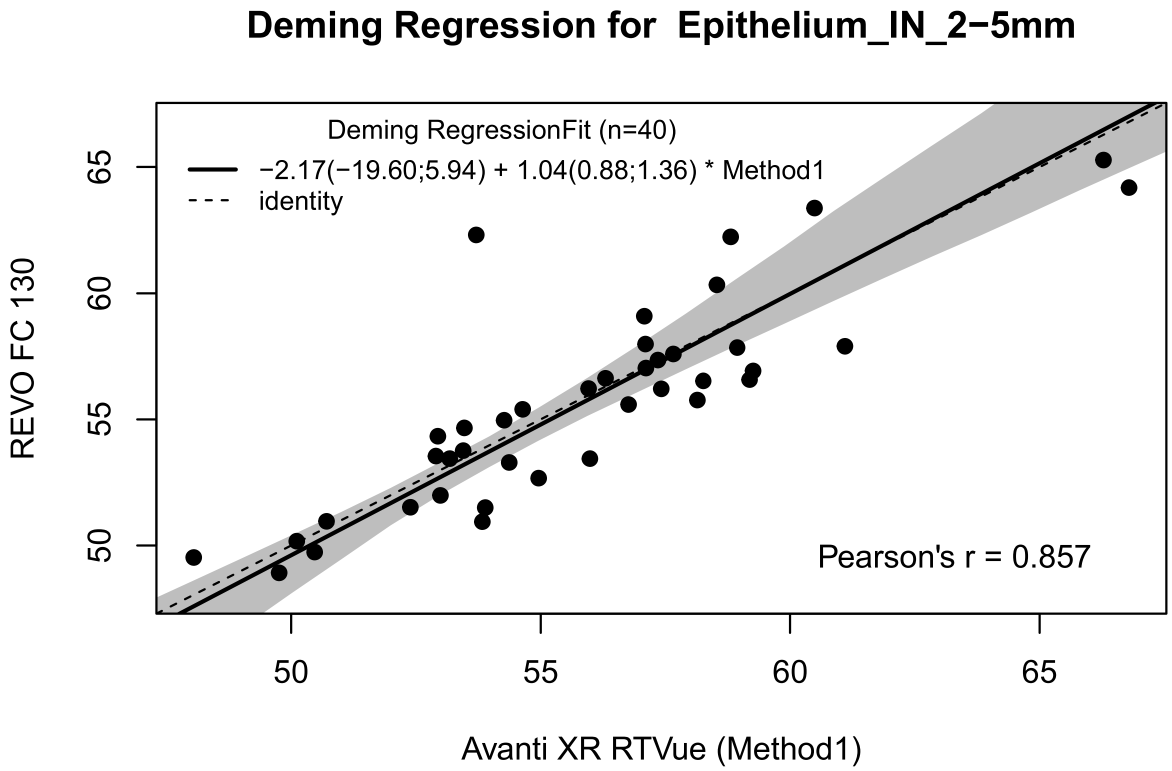
Deming Regression for Epithelium_IN_2−5mm
Deming RegressionFit (n=40)
−2.17(−19.60;5.94) + 1.04(0.88;1.36) * Method1
identity
Pearson's r = 0.857
REVO FC 130
Avanti XR RTVue (Method1)
50
55
60
65
50
55
60
65

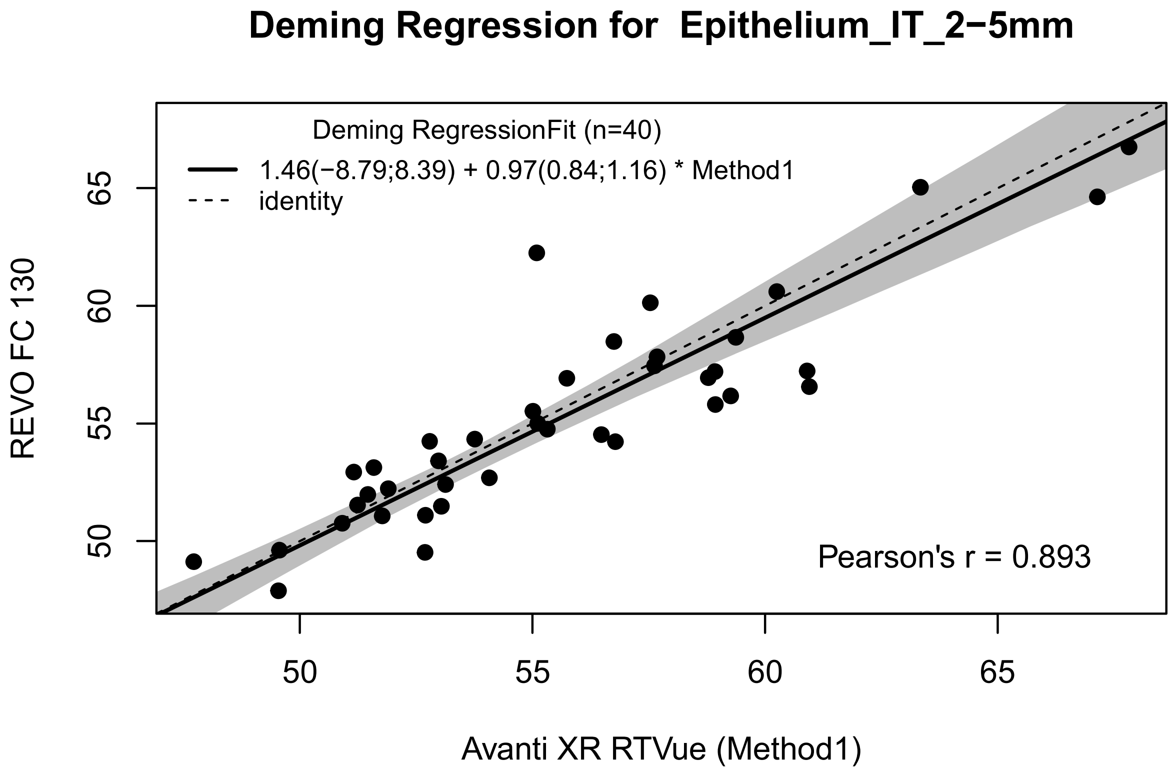
Deming Regression for Epithelium_IT_2−5mm
Deming RegressionFit (n=40)
1.46(−8.79;8.39) + 0.97(0.84;1.16) * Method1
identity
Pearson's r = 0.893
REVO FC 130
Avanti XR RTVue (Method1)
50
55
60
65

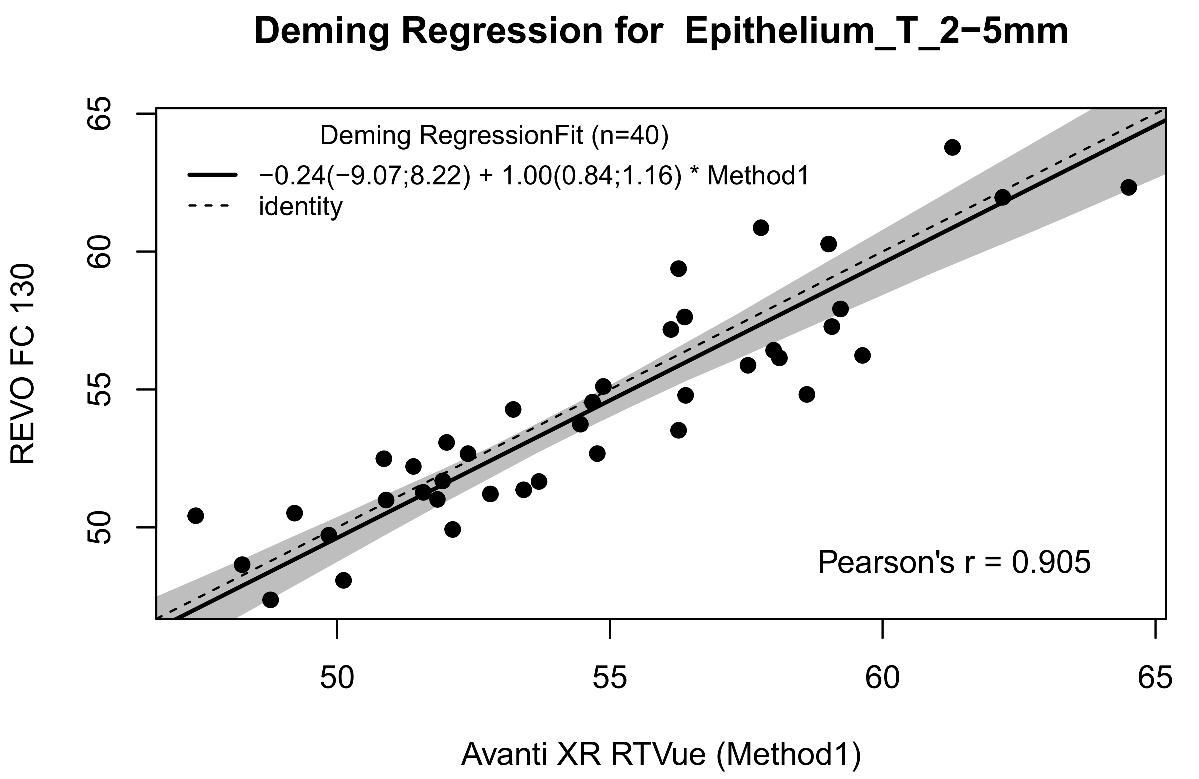

Deming Regression for Epithelium_T_2−5mm
Deming RegressionFit (n=40)
−0.24(−9.07;8.22) + 1.00(0.84;1.16) * Method1
identity
Pearson's r = 0.905
REVO FC 130
Avanti XR RTVue (Method1)
50
55
60
65

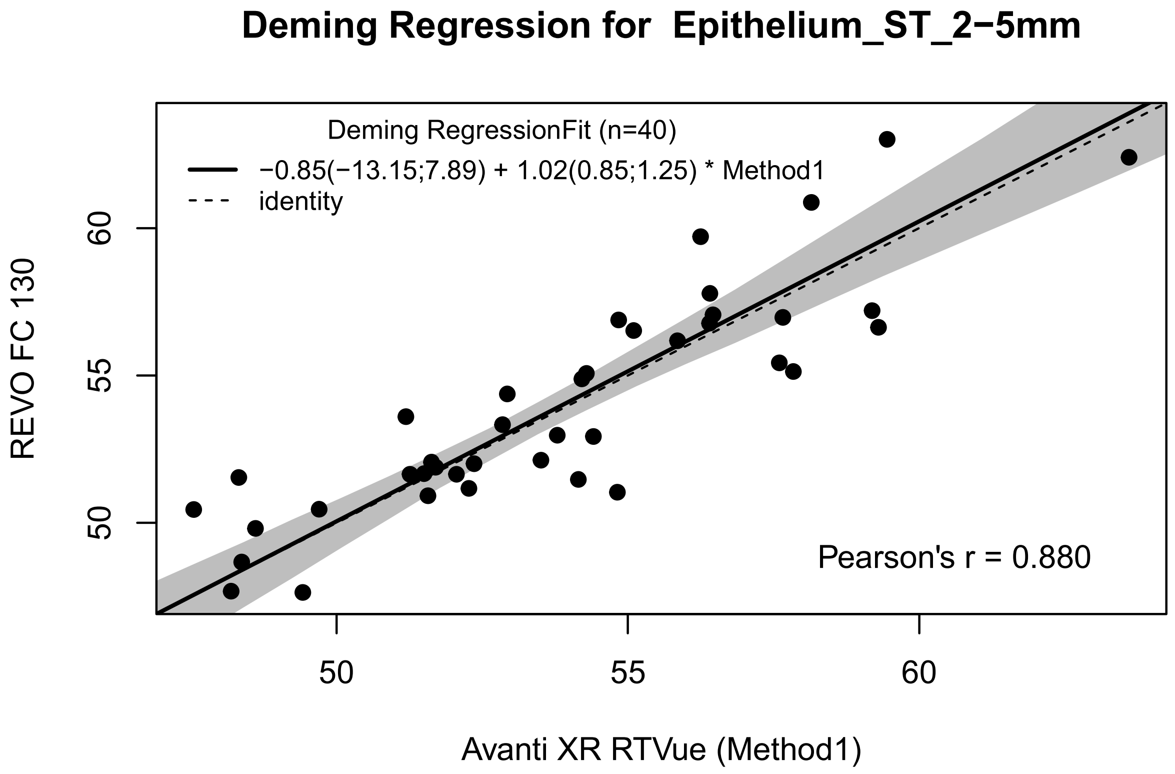

Deming Regression for Epithelium_ST_2−5mm
Deming RegressionFit (n=40)
−0.85(−13.15;7.89) + 1.02(0.85;1.25) * Method1
identity
Pearson's r = 0.880
REVO FC 130
Avanti XR RTVue (Method1)
50
55
60

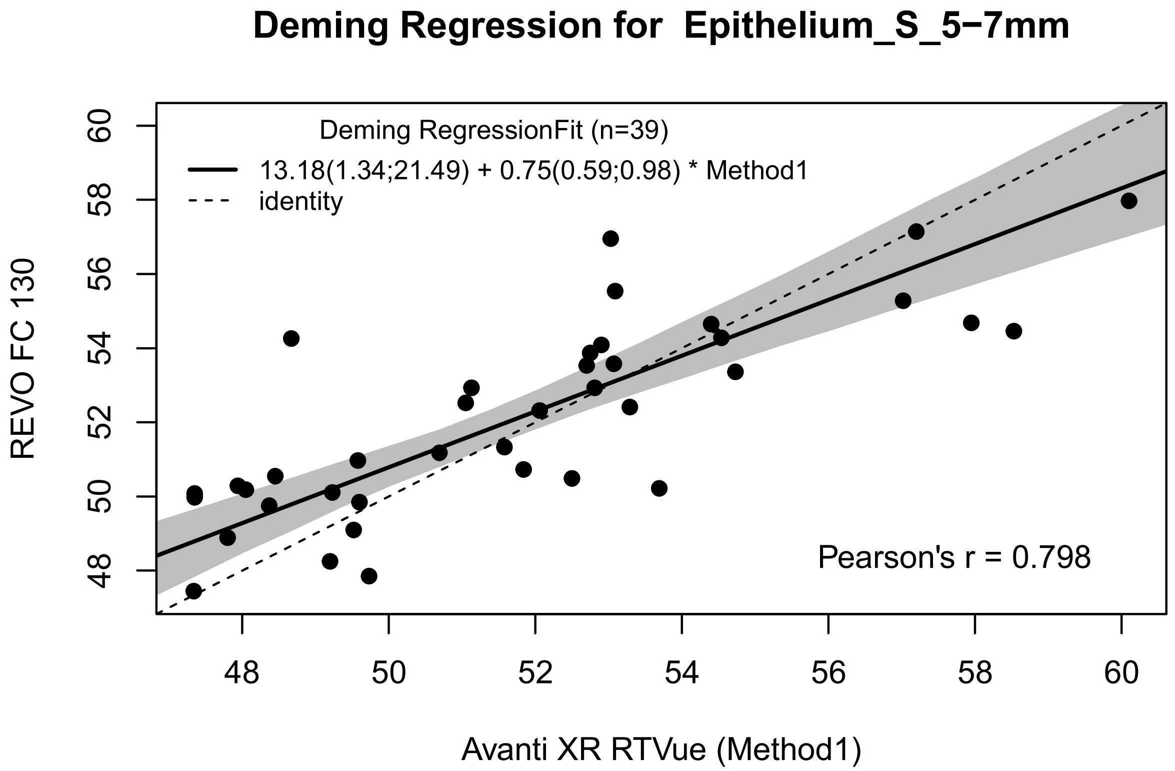
Deming Regression for Epithelium_S_5−7mm
Deming RegressionFit (n=39)
13.18(1.34;21.49) + 0.75(0.59;0.98) * Method1
identity
Pearson's r = 0.798
REVO FC 130
Avanti XR RTVue (Method1)
48
50
52
54
56
58
60

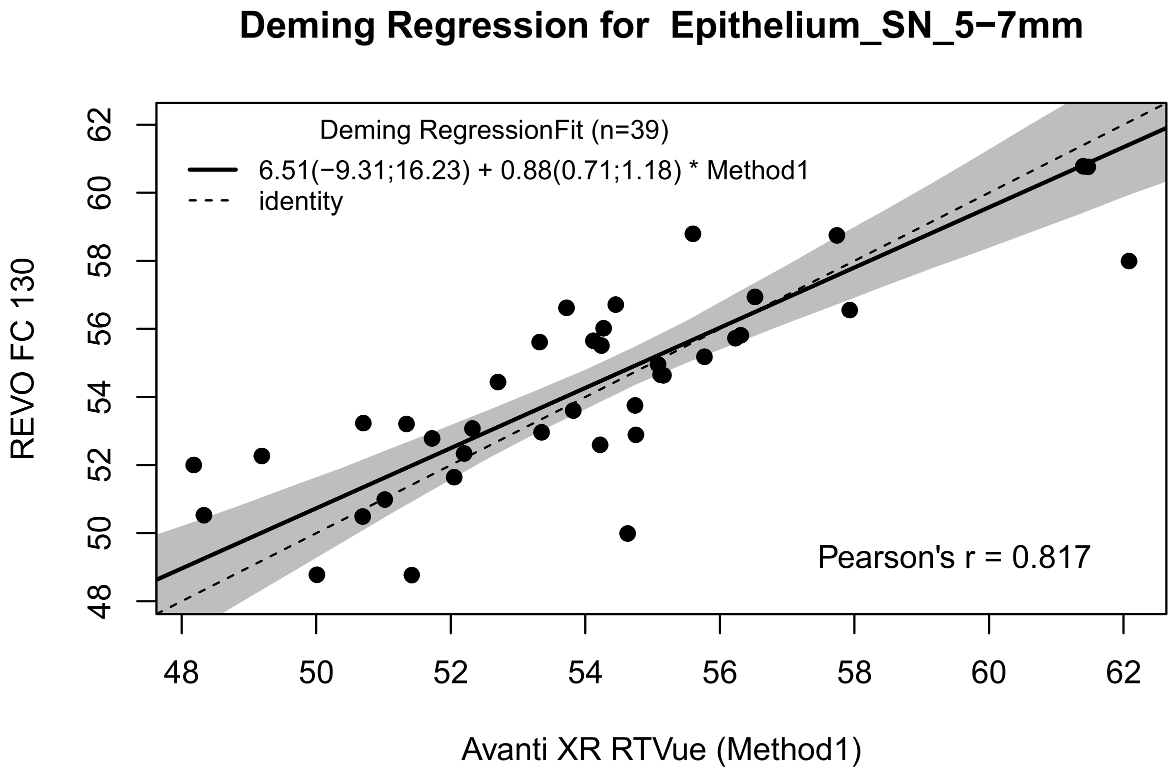
Deming Regression for Epithelium_SN_5−7mm
Deming RegressionFit (n=39)
6.51(−9.31;16.23) + 0.88(0.71;1.18) * Method1
identity
Pearson's r = 0.817
REVO FC 130
Avanti XR RTVue (Method1)
48
50
52
54
56
58
60
62

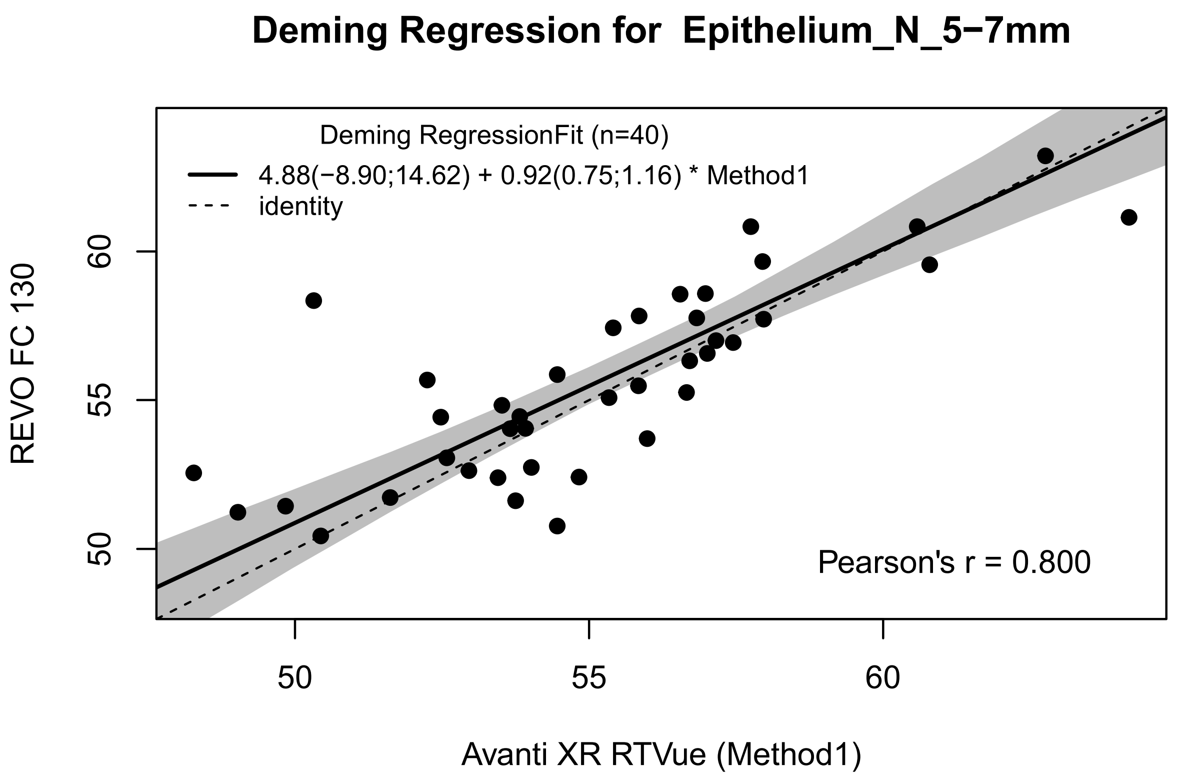
Deming Regression for Epithelium_N_5−7mm
Deming RegressionFit (n=40)
4.88(−8.90;14.62) + 0.92(0.75;1.16) * Method1
identity
Pearson's r = 0.800
REVO FC 130
Avanti XR RTVue (Method1)
50
55
60
50
55
60

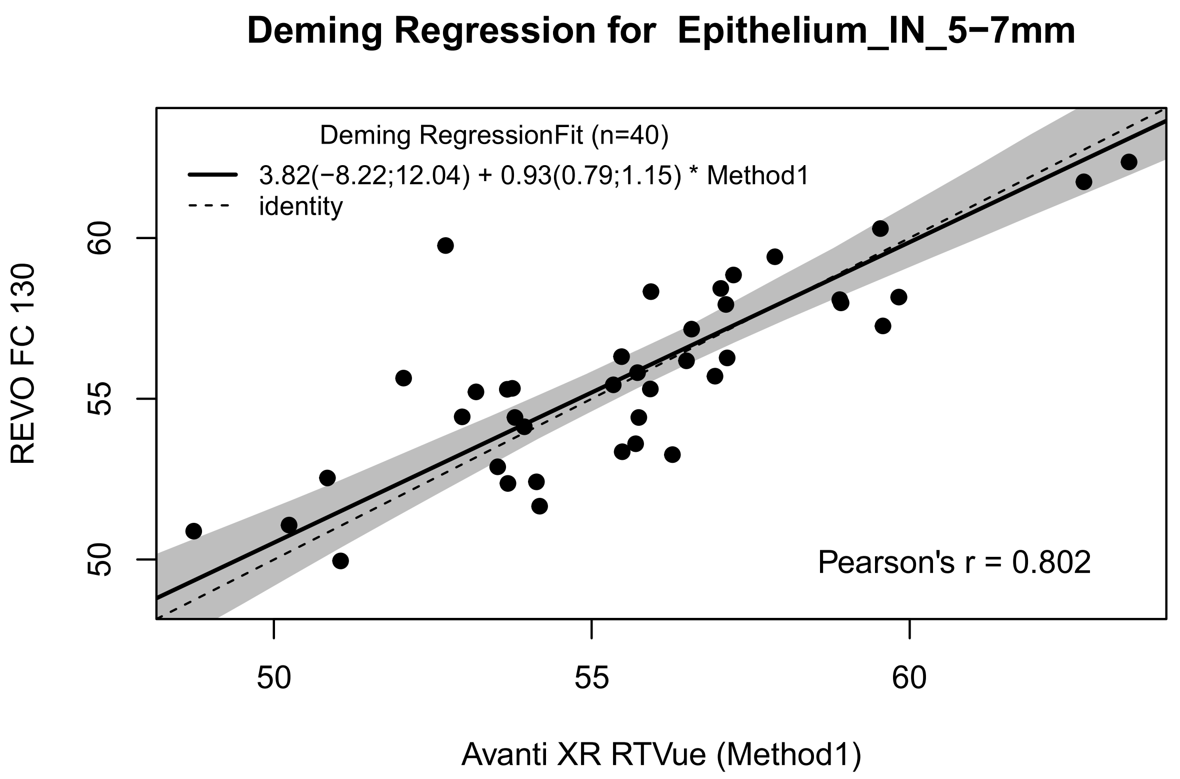

Deming Regression for Epithelium_IN_5−7mm
Deming RegressionFit (n=40)
3.82(−8.22;12.04) + 0.93(0.79;1.15) * Method1
identity
Pearson's r = 0.802
REVO FC 130
Avanti XR RTVue (Method1)
50
55
60
50
55
60

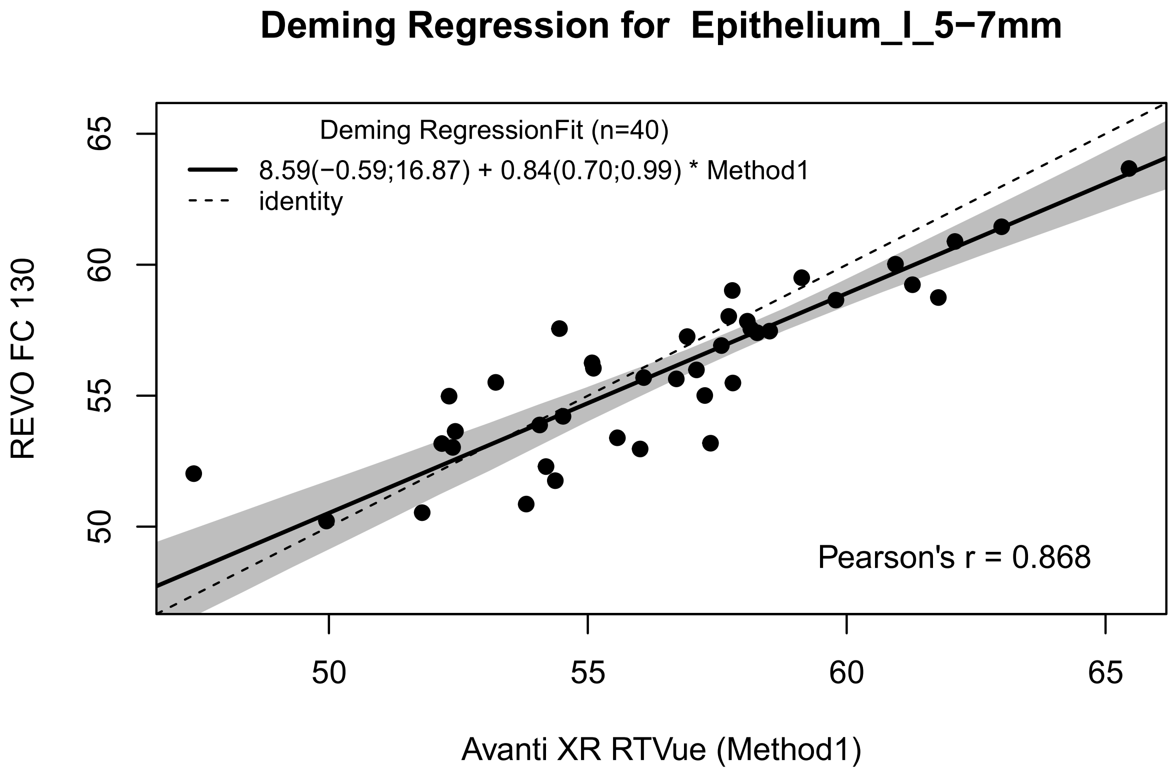

Deming Regression for Epithelium_I_5−7mm
Deming RegressionFit (n=40)
8.59(−0.59;16.87) + 0.84(0.70;0.99) * Method1
identity
Pearson's r = 0.868
REVO FC 130
Avanti XR RTVue (Method1)
50
55
60
65

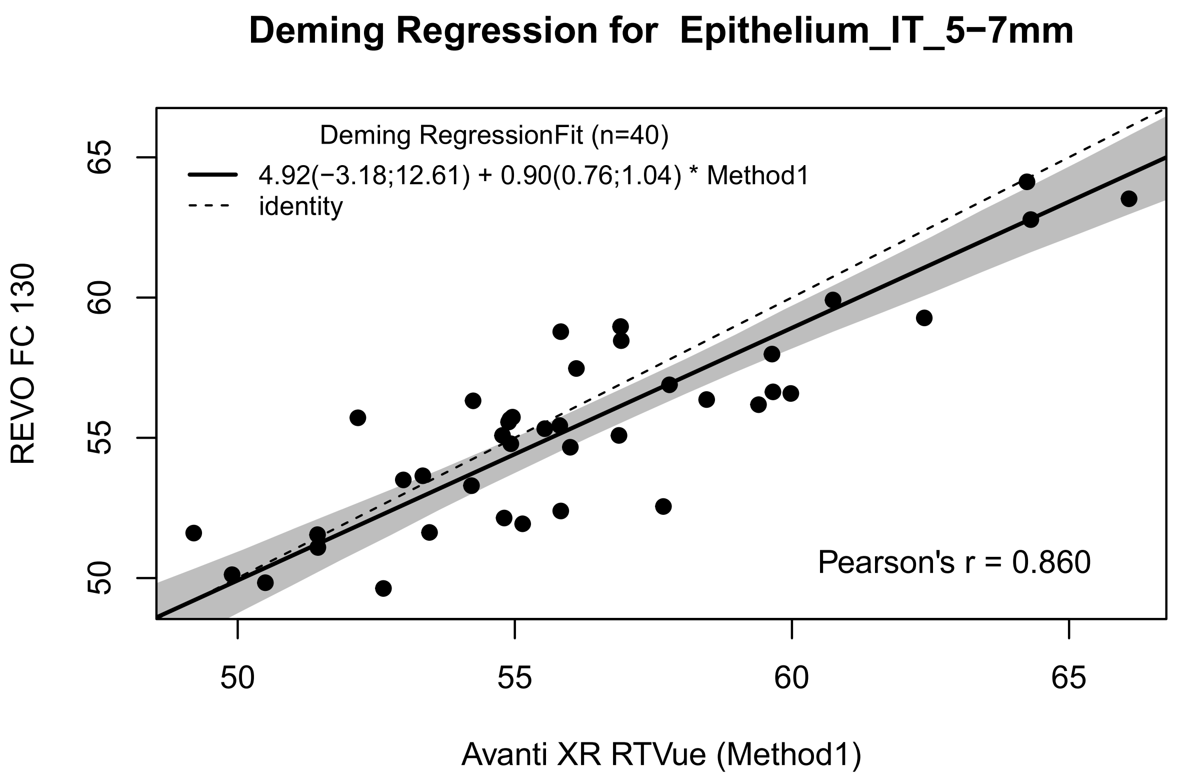
Deming Regression for Epithelium_IT_5−7mm
Deming RegressionFit (n=40)
4.92(−3.18;12.61) + 0.90(0.76;1.04) * Method1
identity
Pearson's r = 0.860
REVO FC 130
Avanti XR RTVue (Method1)
50
55
60
65
50
55
60
65

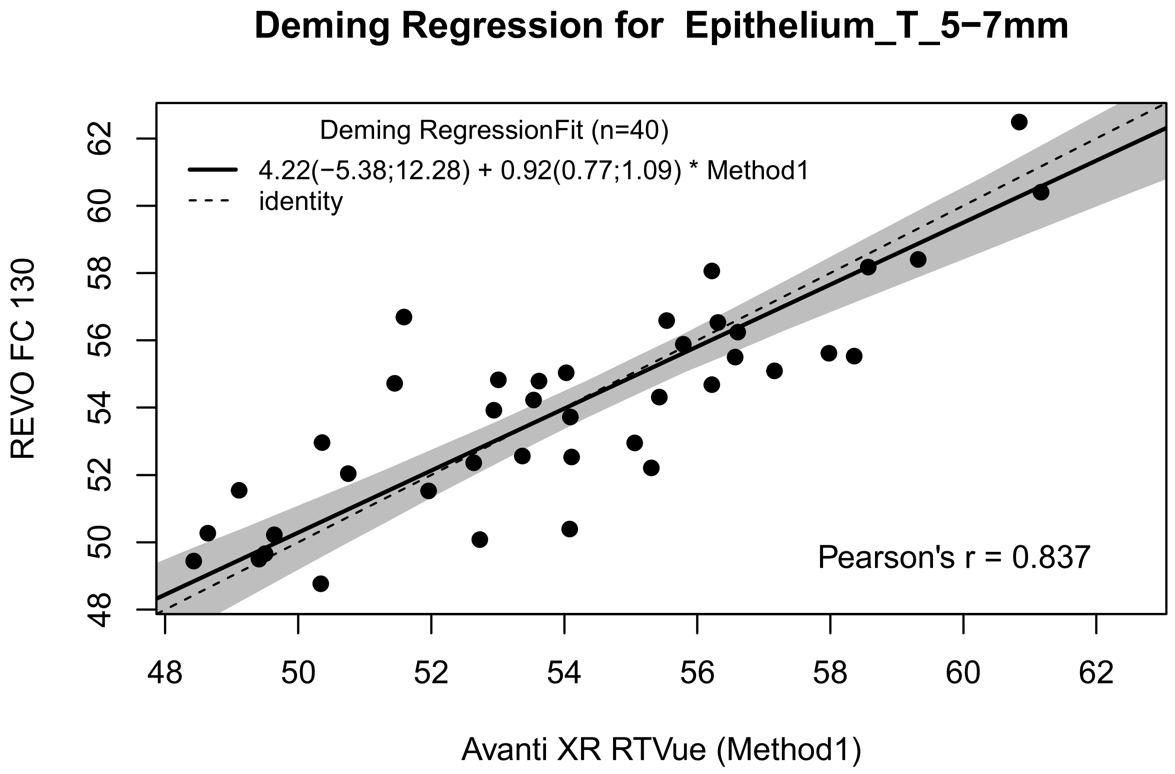
Deming Regression for Epithelium_T_5−7mm
Deming RegressionFit (n=40)
4.22(−5.38;12.28) + 0.92(0.77;1.09) * Method1
identity
Pearson's r = 0.837
REVO FC 130
Avanti XR RTVue (Method1)
48
50
52
54
56
58
60
62

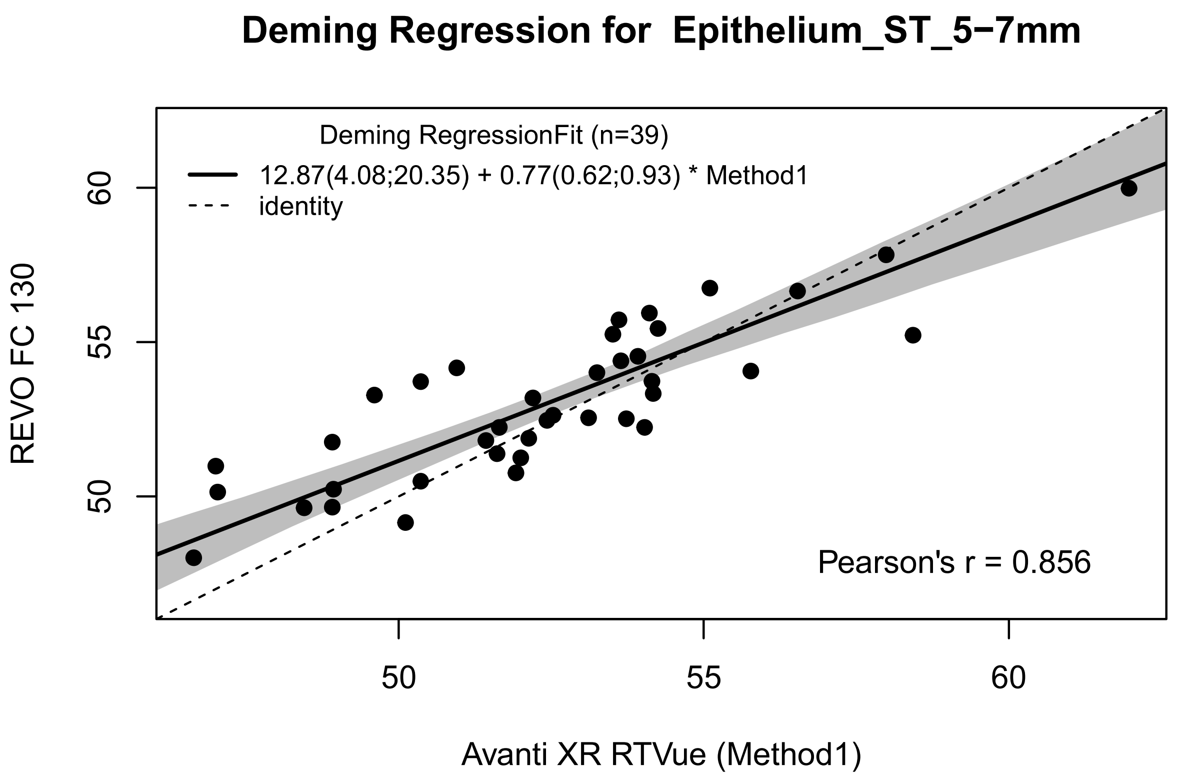
Deming Regression for Epithelium_ST_5−7mm
Deming RegressionFit (n=39)
12.87(4.08;20.35) + 0.77(0.62;0.93) * Method1
identity
Pearson's r = 0.856
REVO FC 130
Avanti XR RTVue (Method1)
50
55
60
50
55
60

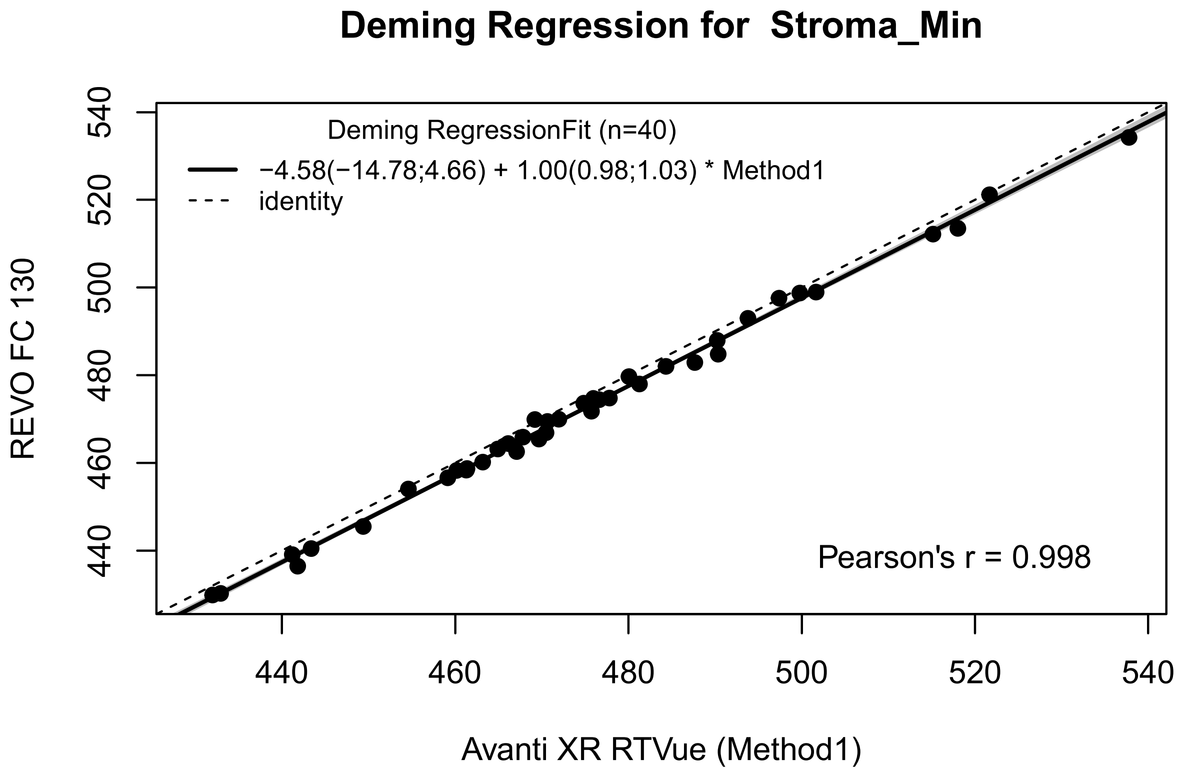
Deming Regression for Stroma_Min
Deming RegressionFit (n=40)
−4.58(−14.78;4.66) + 1.00(0.98;1.03) * Method1
identity
Pearson's r = 0.998
REVO FC 130
Avanti XR RTVue (Method1)
440
460
480
500
520
540

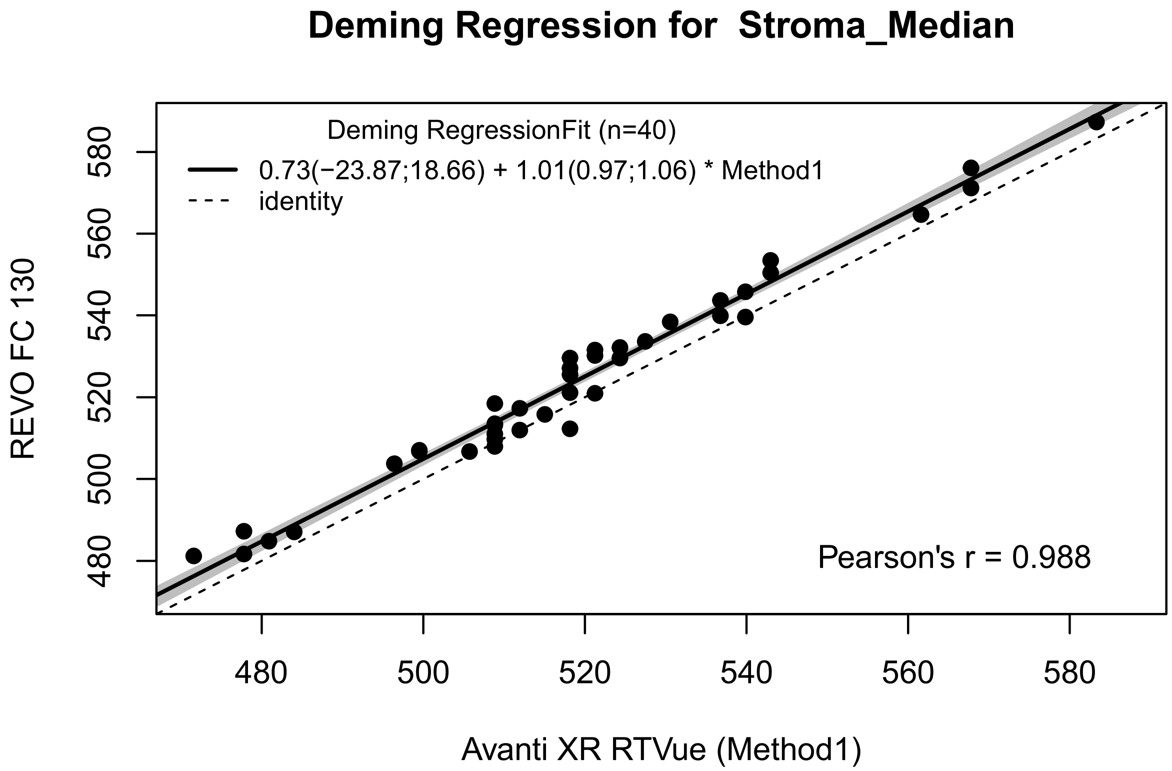

Deming Regression for Stroma_Median
Deming RegressionFit (n=40)
0.73(−23.87;18.66) + 1.01(0.97;1.06) * Method1
identity
Pearson's r = 0.988
REVO FC 130
480
500
520
540
560
580
Avanti XR RTVue (Method1)

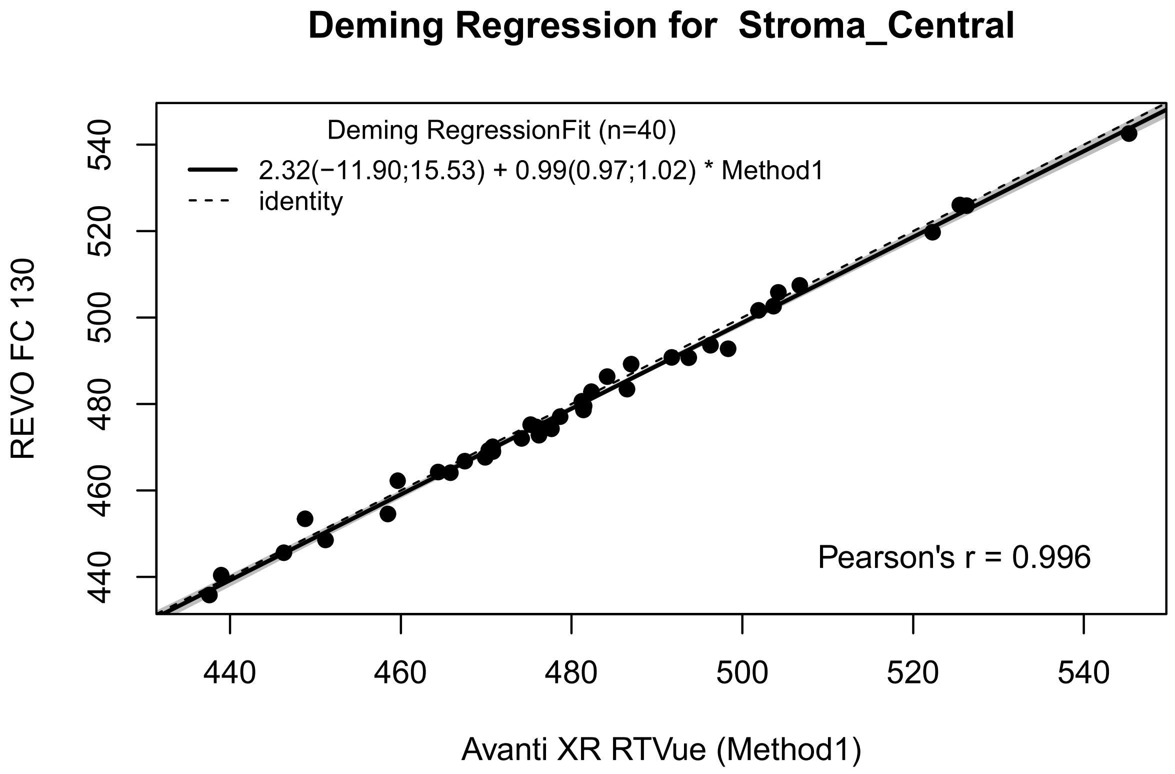
Deming Regression for Stroma_Central
Deming RegressionFit (n=40)
2.32(−11.90;15.53) + 0.99(0.97;1.02) * Method1
identity
Pearson's r = 0.996
REVO FC 130
Avanti XR RTVue (Method1)
440
460
480
500
520
540

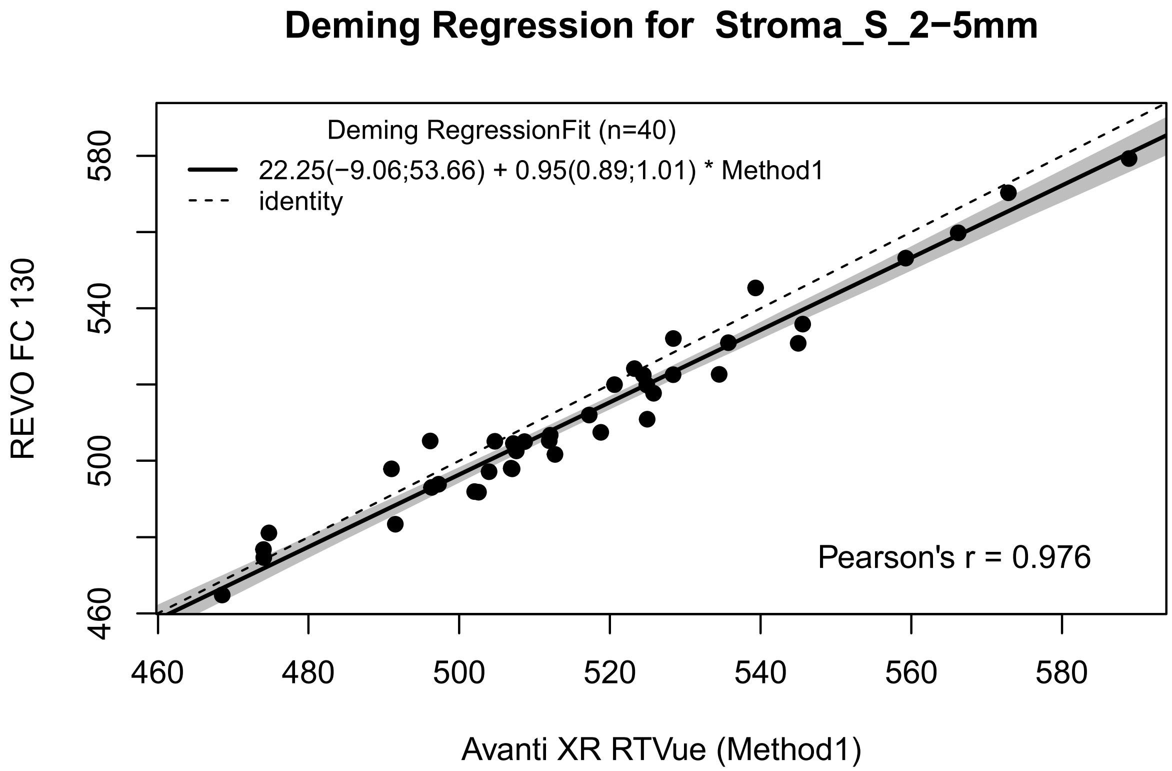

Deming Regression for Stroma_S_2−5mm
Deming RegressionFit (n=40)
22.25(−9.06;53.66) + 0.95(0.89;1.01) * Method1
identity
Pearson's r = 0.976
REVO FC 130
460
500
540
580
460
480
500
520
540
560
580
Avanti XR RTVue (Method1)

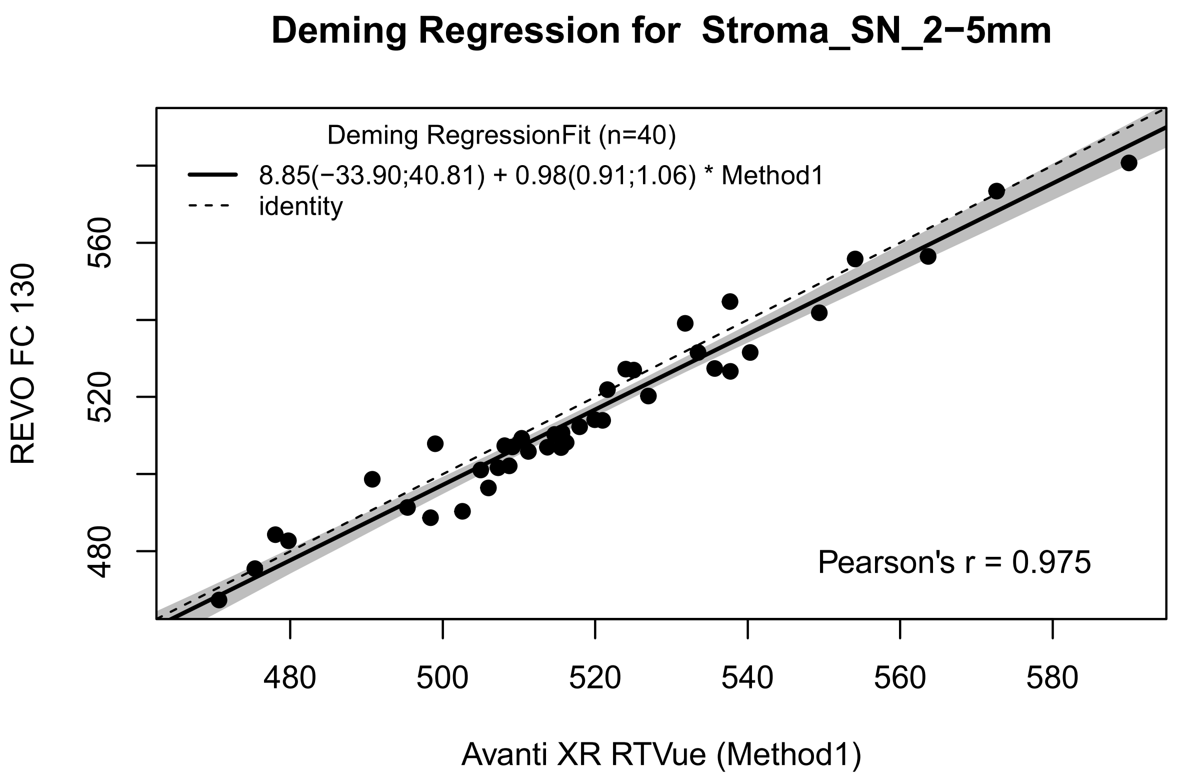
Deming Regression for Stroma_SN_2−5mm
Deming RegressionFit (n=40)
8.85(−33.90;40.81) + 0.98(0.91;1.06) * Method1
identity
Pearson's r = 0.975
REVO FC 130
480
520
560
480
500
520
540
560
580
Avanti XR RTVue (Method1)

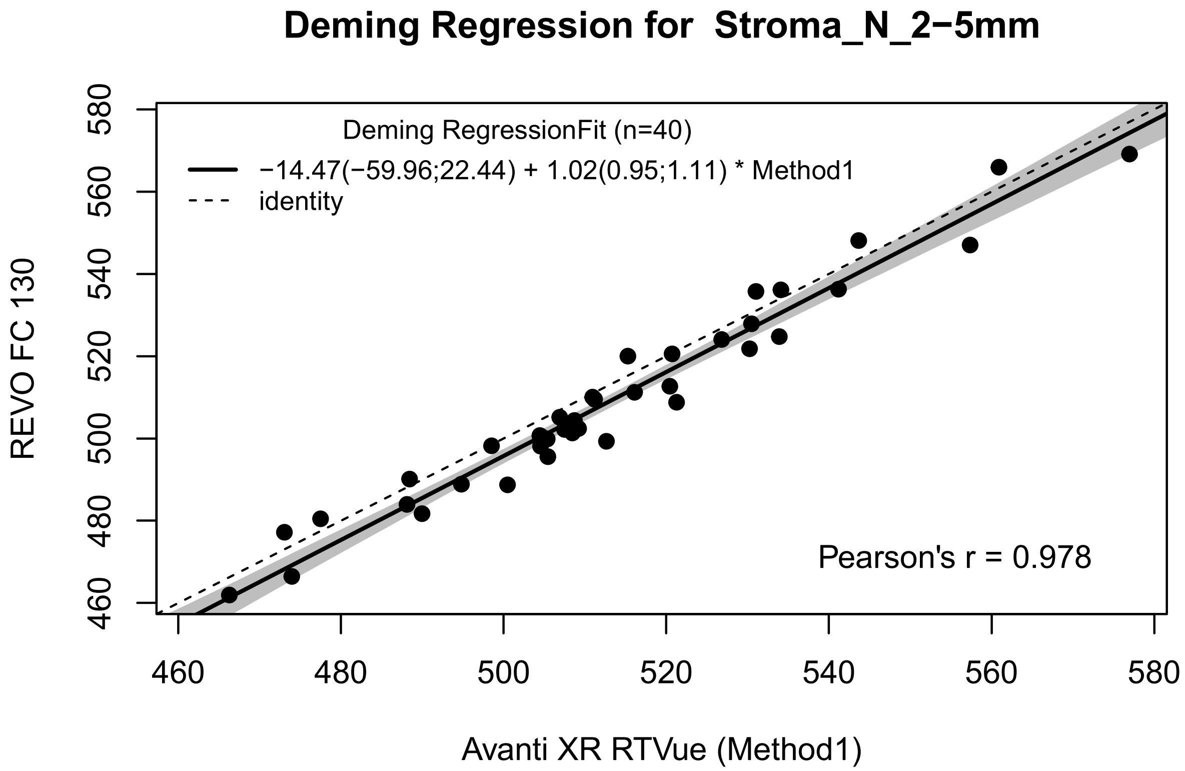
Deming Regression for Stroma_N_2−5mm
Deming RegressionFit (n=40)
−14.47(−59.96;22.44) + 1.02(0.95;1.11) * Method1
identity
Pearson's r = 0.978
REVO FC 130
Avanti XR RTVue (Method1)
460
480
500
520
540
560
580

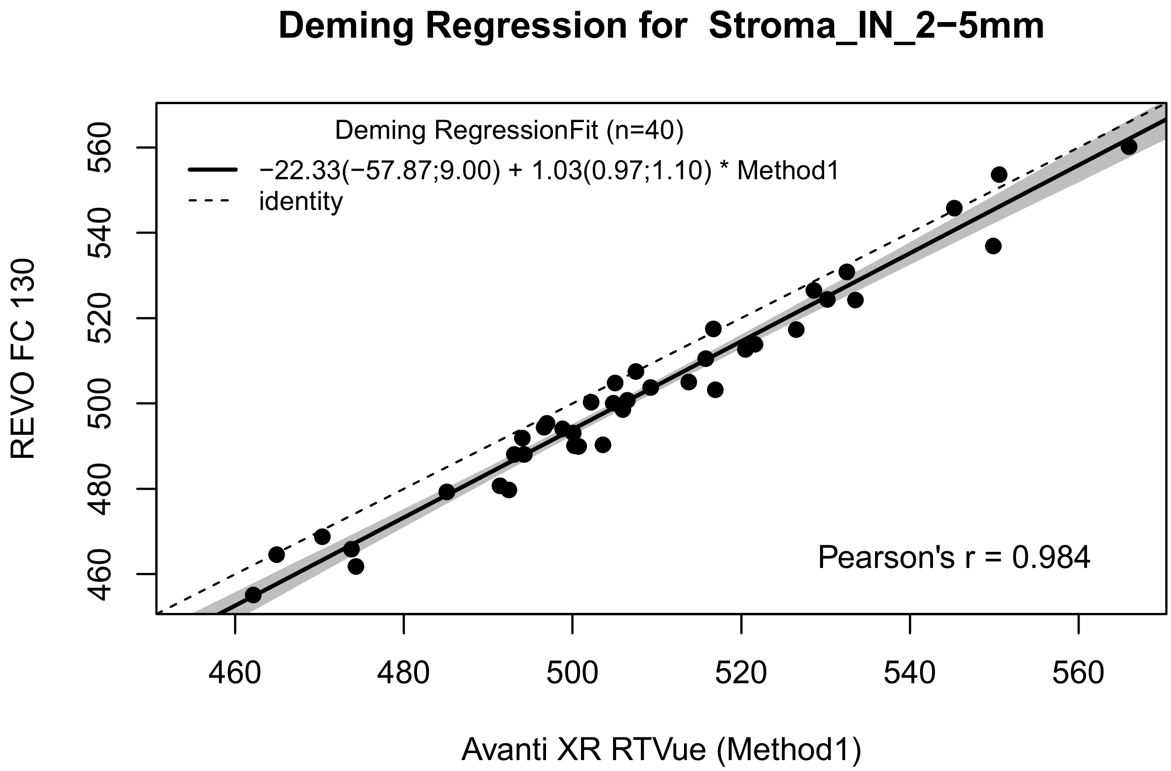

Deming Regression for Stroma_IN_2−5mm
Deming RegressionFit (n=40)
−22.33(−57.87;9.00) + 1.03(0.97;1.10) * Method1
identity
Pearson's r = 0.984
REVO FC 130
Avanti XR RTVue (Method1)
460
480
500
520
540
560

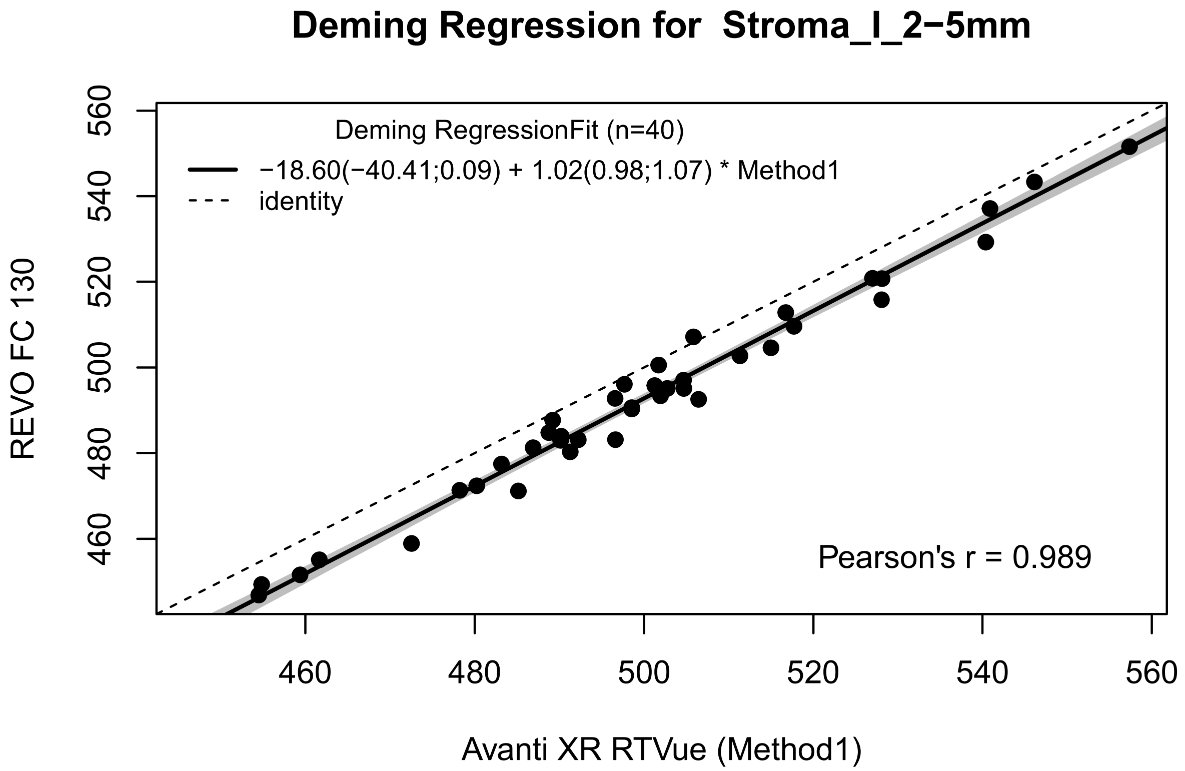
Deming Regression for Stroma_I_2−5mm
Deming RegressionFit (n=40)
−18.60(−40.41;0.09) + 1.02(0.98;1.07) * Method1
identity
Pearson's r = 0.989
REVO FC 130
Avanti XR RTVue (Method1)
460
480
500
520
540
560

Deming Regression for Stroma_IT_2−5mm

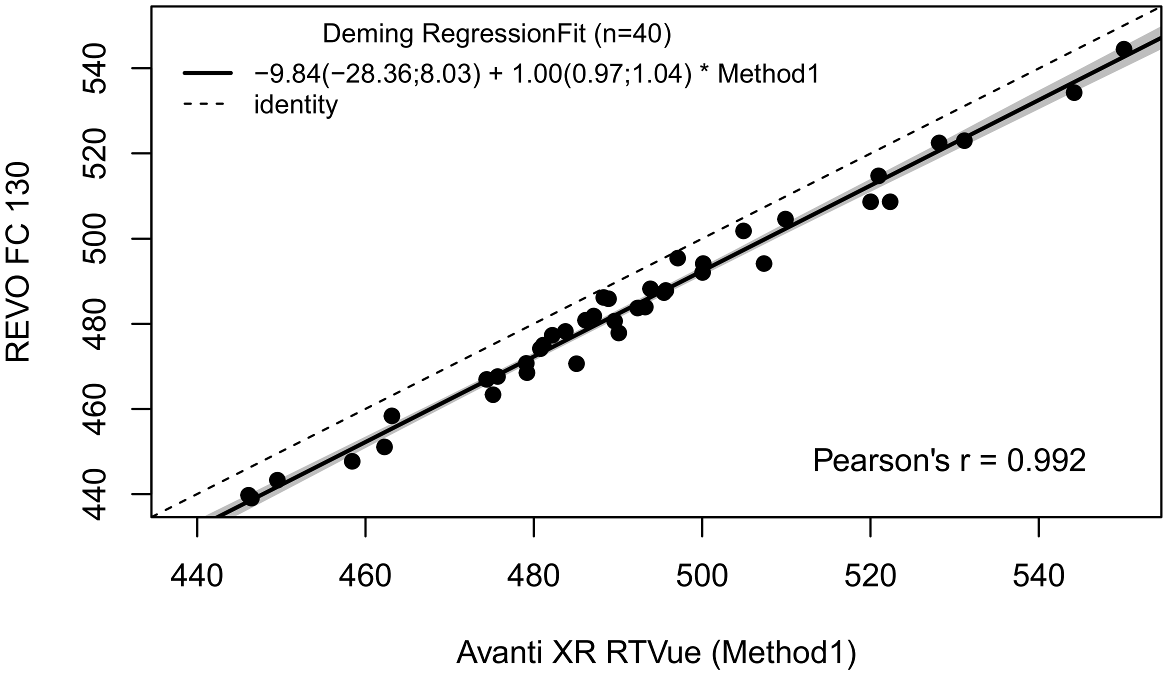

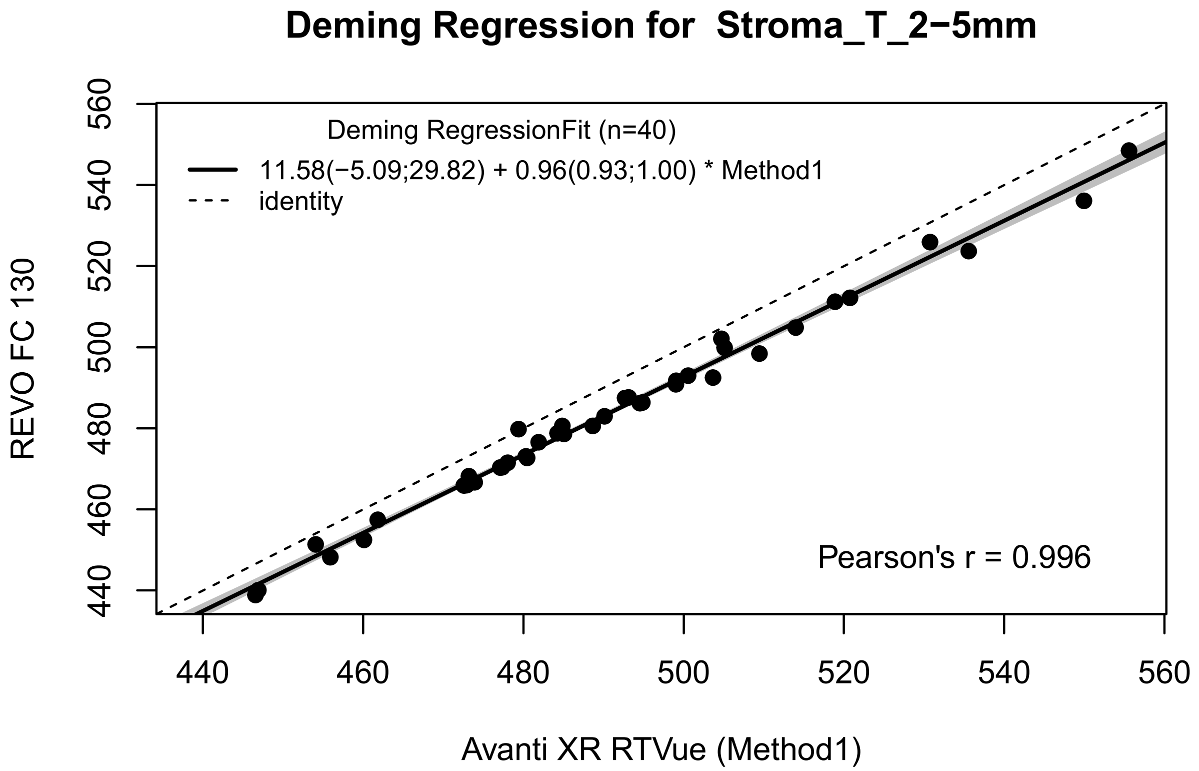
Deming Regression for Stroma_T_2−5mm
Deming RegressionFit (n=40)
11.58(−5.09;29.82) + 0.96(0.93;1.00) * Method1
identity
Pearson's r = 0.996
REVO FC 130
Avanti XR RTVue (Method1)
440
460
480
500
520
540
560

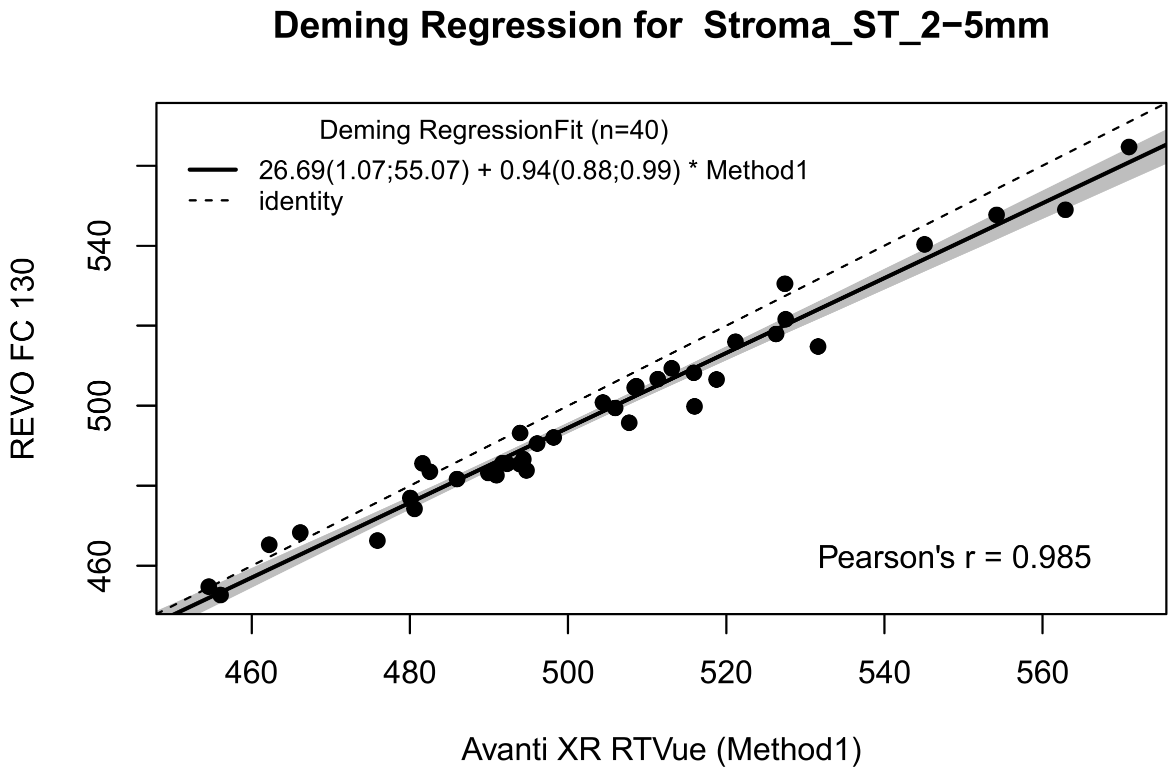

Deming Regression for Stroma_ST_2−5mm
Deming RegressionFit (n=40)
26.69(1.07;55.07) + 0.94(0.88;0.99) * Method1
identity
Pearson's r = 0.985
REVO FC 130
460
500
540
460
480
500
520
540
560
Avanti XR RTVue (Method1)

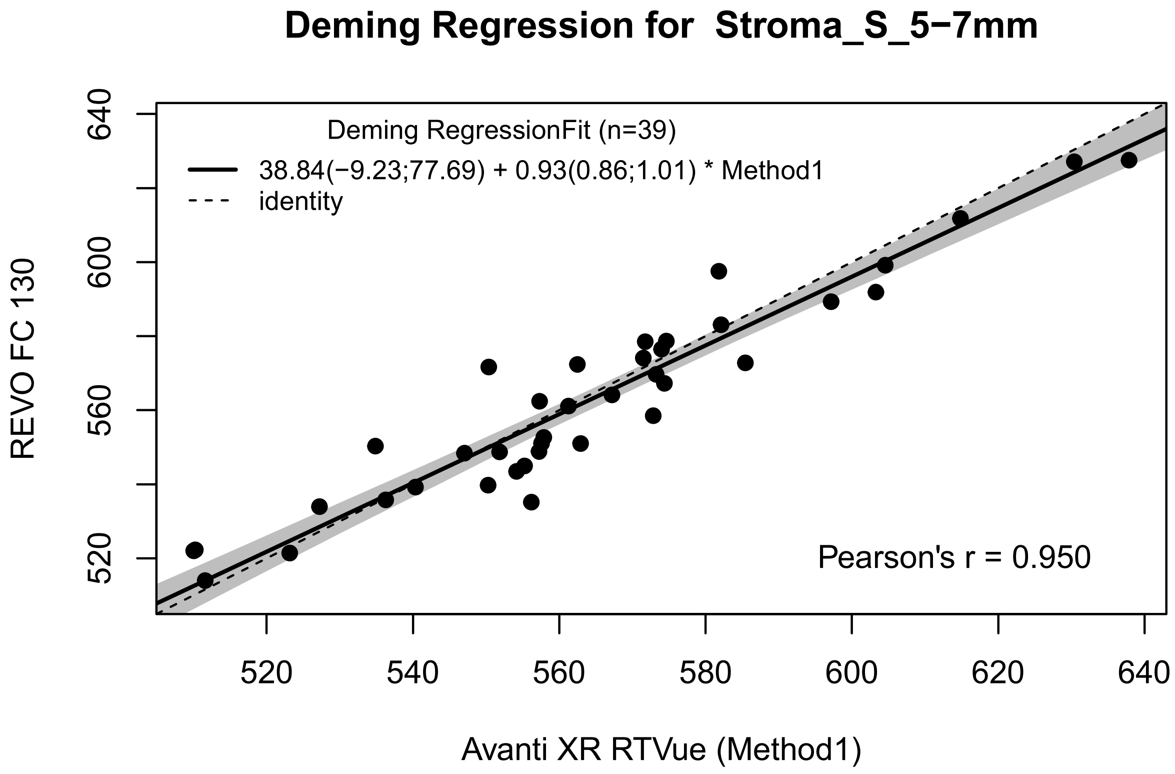
Deming Regression for Stroma_S_5−7mm
Deming RegressionFit (n=39)
38.84(−9.23;77.69) + 0.93(0.86;1.01) * Method1
identity
Pearson's r = 0.950
REVO FC 130
Avanti XR RTVue (Method1)
520
540
560
580
600
620
640

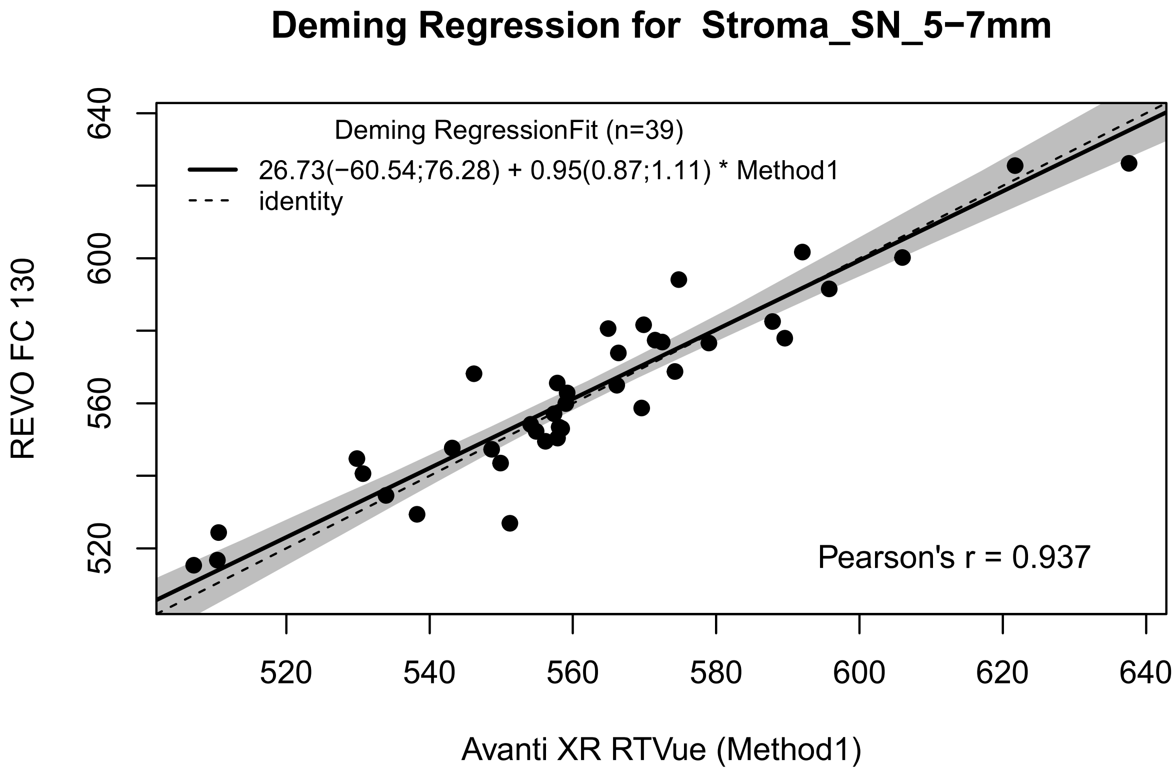
Deming Regression for Stroma_SN_5−7mm
Deming RegressionFit (n=39)
26.73(−60.54;76.28) + 0.95(0.87;1.11) * Method1
identity
Pearson's r = 0.937
REVO FC 130
Avanti XR RTVue (Method1)
520
540
560
580
600
620
640

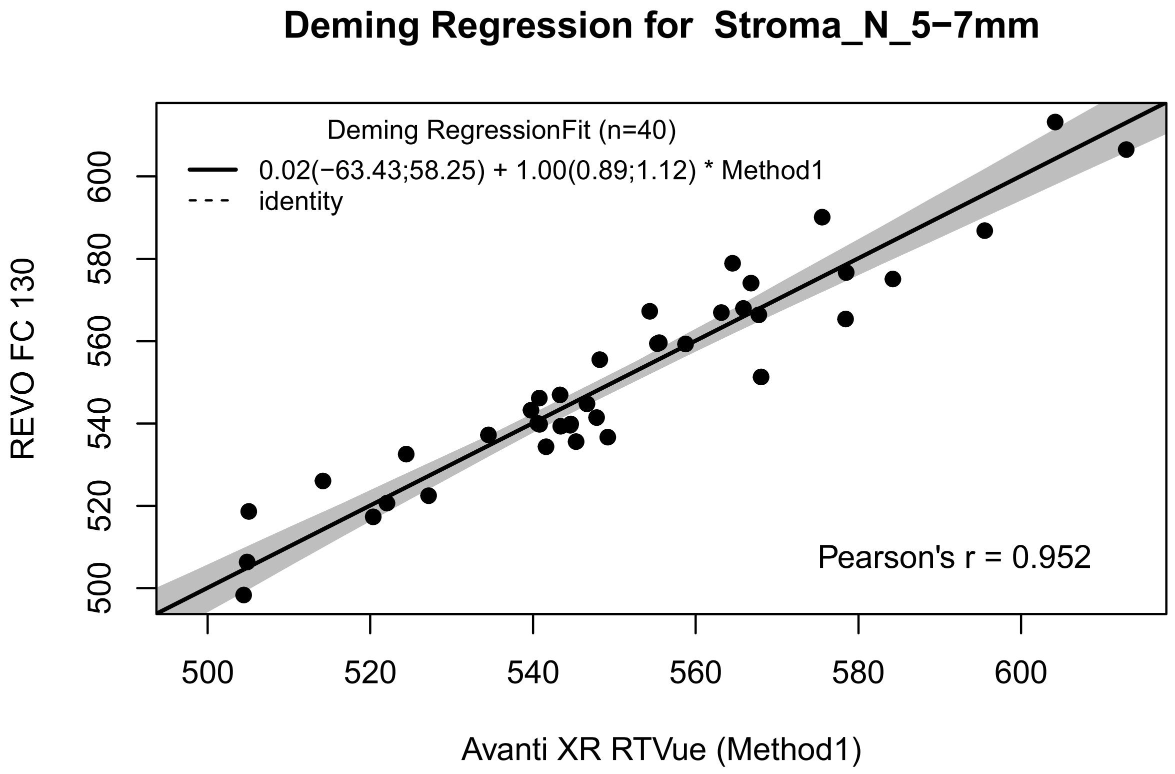
Deming Regression for Stroma_N_5−7mm
Deming RegressionFit (n=40)
0.02(−63.43;58.25) + 1.00(0.89;1.12) * Method1
identity
Pearson's r = 0.952
REVO FC 130
Avanti XR RTVue (Method1)
500
520
540
560
580
600

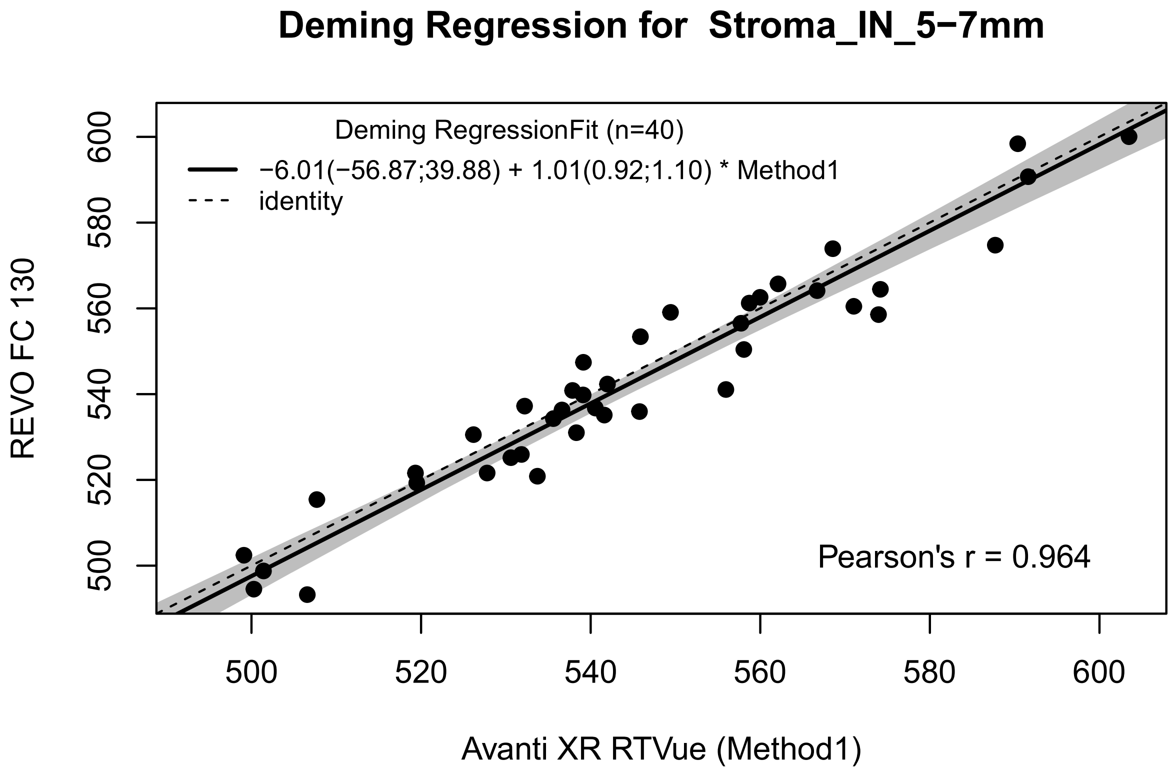
Deming Regression for Stroma_IN_5−7mm
Deming RegressionFit (n=40)
−6.01(−56.87;39.88) + 1.01(0.92;1.10) * Method1
identity
Pearson's r = 0.964
REVO FC 130
Avanti XR RTVue (Method1)
500
520
540
560
580
600

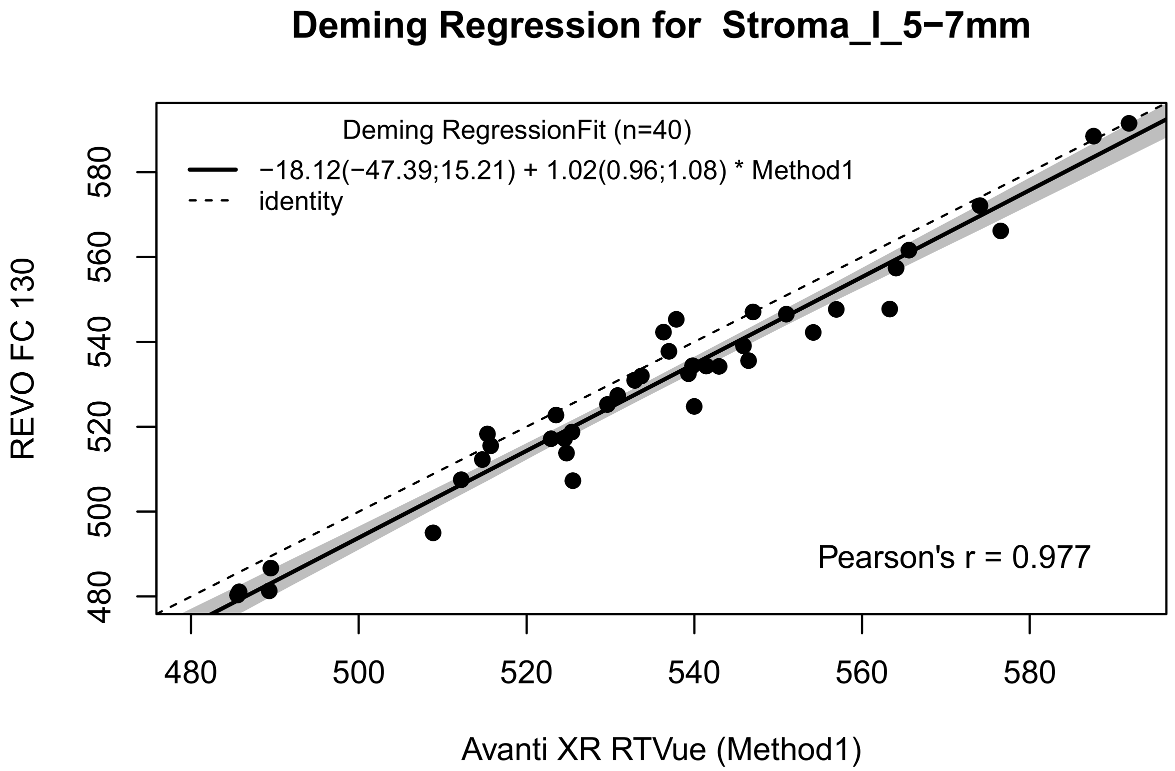

Deming Regression for Stroma_I_5−7mm
Deming RegressionFit (n=40)
−18.12(−47.39;15.21) + 1.02(0.96;1.08) * Method1
identity
Pearson's r = 0.977
REVO FC 130
480
500
520
540
560
580
Avanti XR RTVue (Method1)

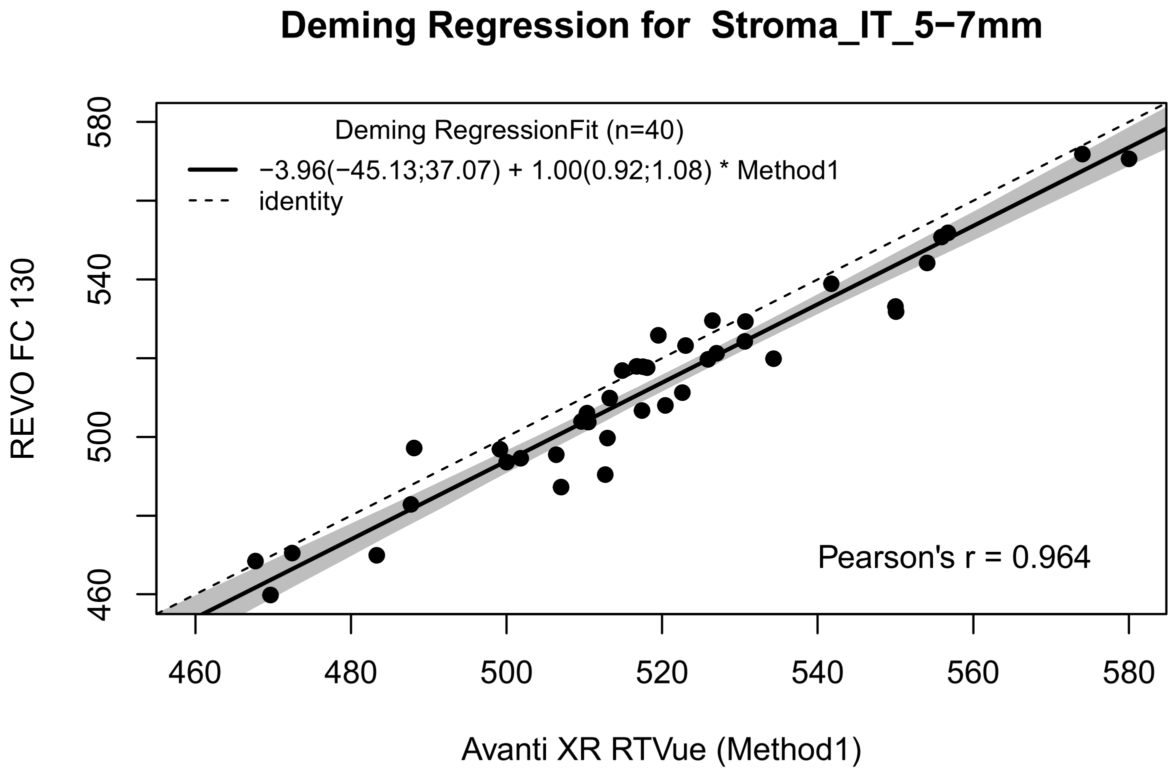

Deming Regression for Stroma_IT_5−7mm
Deming RegressionFit (n=40)
−3.96(−45.13;37.07) + 1.00(0.92;1.08) * Method1
identity
Pearson's r = 0.964
REVO FC 130
Avanti XR RTVue (Method1)
460
480
500
520
540
560
580

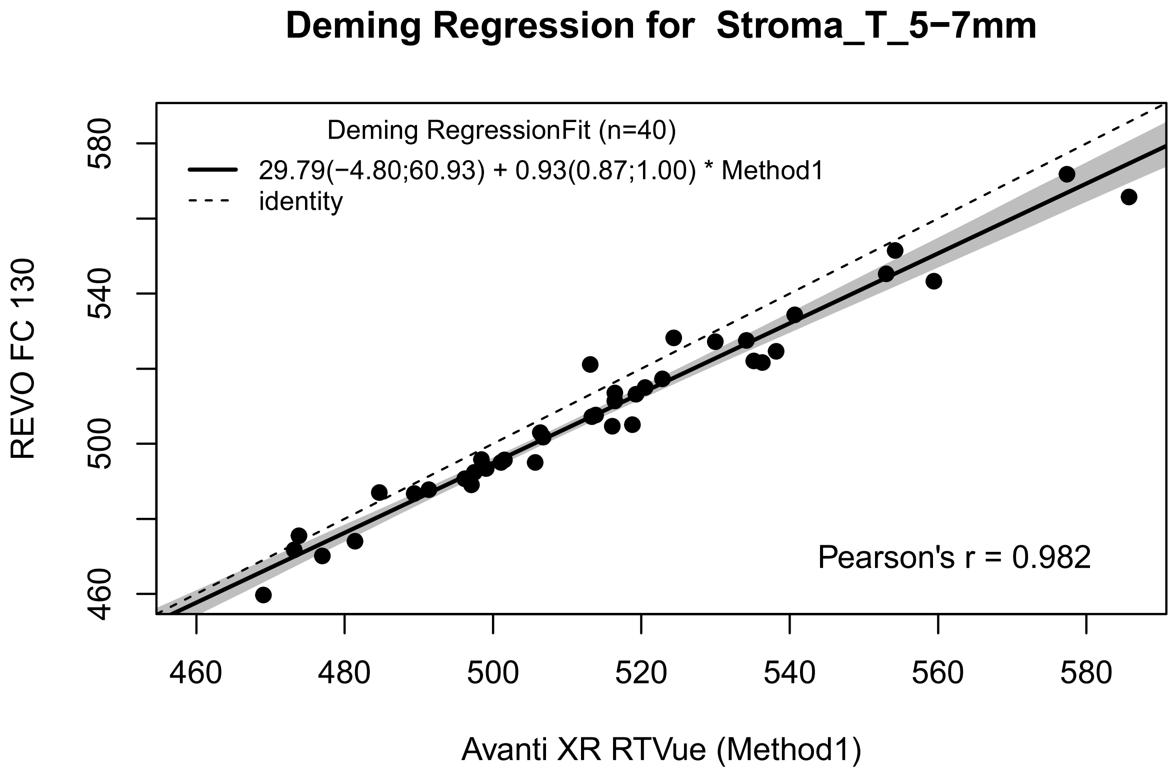

Deming Regression for Stroma_T_5−7mm
Deming RegressionFit (n=40)
29.79(−4.80;60.93) + 0.93(0.87;1.00) * Method1
identity
Pearson's r = 0.982
REVO FC 130
460
500
540
580
460
480
500
520
540
560
580
Avanti XR RTVue (Method1)

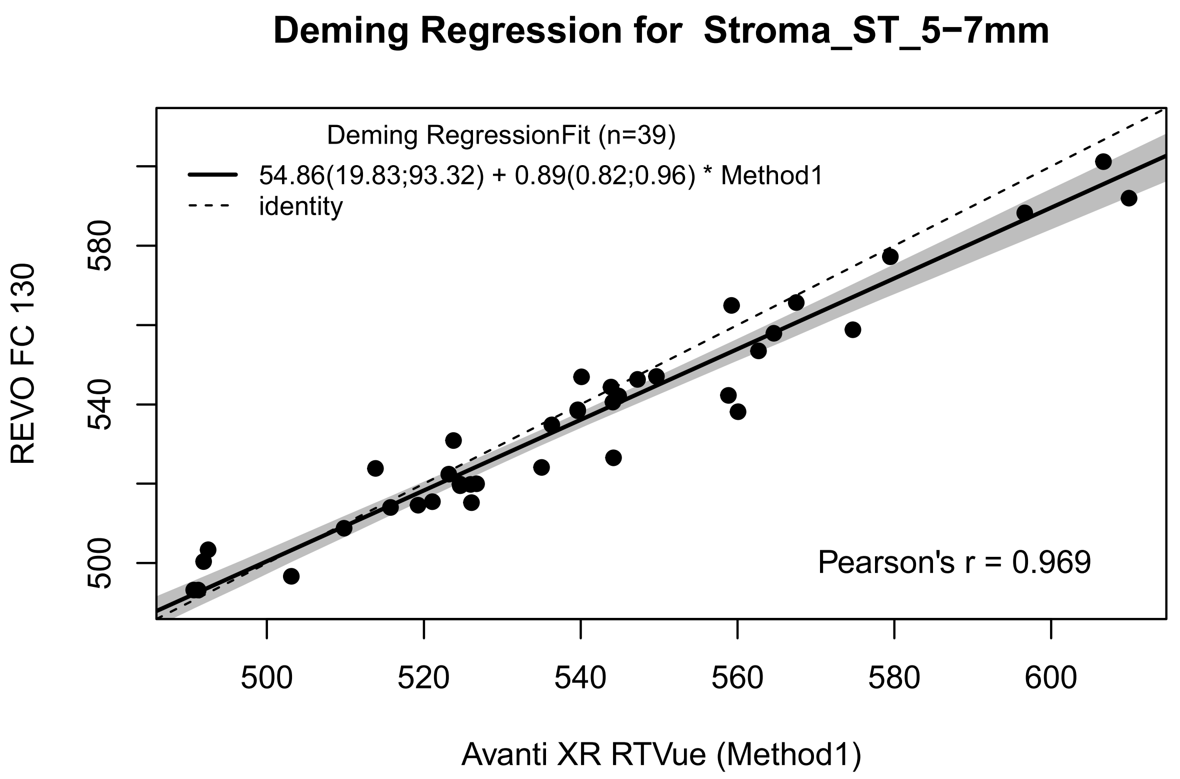

Deming Regression for Stroma_ST_5−7mm
Deming RegressionFit (n=39)
54.86(19.83;93.32) + 0.89(0.82;0.96) * Method1
identity
Pearson's r = 0.969
REVO FC 130
500
540
580
500
520
540
560
580
600
Avanti XR RTVue (Method1)
